# White matter micro- and macrostructure brain charts for the human lifespan

**DOI:** 10.1038/s41586-026-10454-2

**Published:** 2026-05-13

**Authors:** Michael E. Kim, Chenyu Gao, Karthik Ramadass, Nancy R. Newlin, Praitayini Kanakaraj, Sam Bogdanov, Gaurav Rudravaram, Derek Archer, Timothy J. Hohman, Angela L. Jefferson, Victoria L. Morgan, Alexandra Roche, Dario J. Englot, Susan M. Resnick, Lori L. Beason-Held, Laurie E. Cutting, Laura A. Barquero, Micah A. D’archangel, Tin Q. Nguyen, Kathryn L. Humphreys, Yanbin Niu, Sophia Vinci-Booher, Carissa J. Cascio, The HABS-HD Study Team, Sid O’Bryant, L. Taylor Davis, Zhiyuan Li, Simon N. Vandekar, Panpan Zhang, John C. Gore, Bennett A. Landman, Kurt G. Schilling

**Affiliations:** 1 Department of Computer Science, Vanderbilt University, Nashville, TN USA; 2 Department of Electrical and Computer Engineering, Vanderbilt University, Nashville, TN USA; 3 Medical Scientist Training Program, Vanderbilt University, Nashville, TN USA; 4 Vanderbilt Memory and Alzheimer’s Center, Vanderbilt University Medical Center, Nashville, TN USA; 5 Vanderbilt Genetics Institute, Vanderbilt University Medical Center, Nashville, TN USA; 6 Vanderbilt Brain Institute, Vanderbilt University, Nashville, TN USA; 7 Department of Medicine, Vanderbilt University Medical Center, Nashville, TN USA; 8 Department of Neurology, Vanderbilt University Medical Center, Nashville, TN USA; 9 Department of Psychology and Human Development, Vanderbilt University, Nashville, TN USA; 10 Department of Psychiatry and Behavioral Sciences, Vanderbilt University Medical Center, Nashville, TN USA; 11 Department of Radiology and Radiological Sciences, Vanderbilt University Medical Center, Nashville, TN USA; 12 Vanderbilt University Institute of Imaging Science, Nashville, TN USA; 13 Department of Biomedical Engineering, Vanderbilt University, Nashville, TN USA; 14 Department of Neurological Surgery, Vanderbilt University Medical Center, Nashville, TN USA; 15 Laboratory of Behavioral Neuroscience, National Institute on Aging, National Institutes of Health, Baltimore, MD USA; 16 Department of Special Education, Peabody College of Education and Human Development, Nashville, TN USA; 17 Life Span Institute and Department of Psychology, University of Kansas, Lawrence, KS USA; 18 Department of Computer Science, Park University, Parkville, MO USA; 19 Department of Biostatistics, Vanderbilt University Medical Center, Nashville, TN USA; 20 Department of Family Medicine, University of North Texas Health Science Center, Fort Worth, TX USA; 21 Laboratory of Neuro Imaging, Department of Neurology, University of California School of Medicine, Los Angeles, CA USA; 22 Department of Neurology, Johns Hopkins University, Baltimore, MD USA

**Keywords:** Brain imaging, Data processing

## Abstract

The human brain relies on a complex network of connections to function, with white matter acting as the primary communication highway between different brain regions^1,2^. Disruptions in these critical communication pathways are linked to several neurological, psychiatric and developmental disorders^3,4^. Although clinicians have long used standard growth charts to track physical development^5^, with more recent work translating these to whole-brain and grey matter measurements^6–9^, there has been no equivalent reference standard for white matter. Establishing a readily available normative reference is an imperative first step if we hope to utilize these white matter structural biomarkers clinically. Here we present lifespan reference charts for human brain white matter. By processing and standardizing 35,120 brain scans from diverse global studies, we mapped the typical growth, maturation and age-related decline of specific brain pathways from birth to 100 years of age. These reference charts establish a fundamental benchmark for healthy brain development and ageing, allowing researchers and clinicians to quantify how an individual’s brain deviates from typical patterns and highlighting disorder-related alterations. Furthermore, the accompanying open access charts enable the scientific and clinical communities to evaluate new patient and research data against these normative baselines, facilitating future clinical and neuroscience studies.

## Abstract (web summary)

Integration of data representing 35,120 brain scans from diverse global studies enables construction of reference charts that define normative microstructural and macrostructural properties across the human lifespan for research and clinical diagnosis.

## Main

The human brain is an intricately organized network, with white matter (WM) forming the backbone of large-scale neural communication^1,2^. Although much focus in neuroscience has been placed on grey matter morphology and its association with cognition, behaviour and disease, WM comprises nearly half of total brain volume and serves as the fundamental conduit for information transfer between cortical and subcortical regions. Disruptions in WM integrity are implicated across a spectrum of neurodevelopmental, neuropsychiatric and neurodegenerative disorders^3,4^. However, despite these crucial roles of WM in brain functions, no standardized normative reference data have previously been derived for characterizing individual variability, developmental milestones or pathological deviations in WM structure. These may now be derived using appropriate image analysis tools by leveraging the large array of imaging datasets that are publicly available.

The concept of normative brain charts—analogous to paediatric growth charts—has recently been introduced^6^ to benchmark some neuroanatomical trajectories across the lifespan, including grey matter features such as cortical thickness and volume^6–9^. These efforts have revealed fundamental principles of neurodevelopment and ageing, enabling robust comparisons across individuals and clinical populations. However, in the context of WM, these previous efforts were predominantly limited to global volumetric analyses and did not provide the tract-specific, pathway-level phenotypes that are needed to localize deviations and to relate WM architecture to circuit function and dysfunction. This constraint is largely due to methodological challenges of diffusion MRI (dMRI), the only non-invasive imaging modality that allows for studying directionality and organization of WM pathways in the human brain. These challenges include complexities in fibre tractography, inter-site variability and historically limited large-scale, harmonized datasets^10^. Given the increasing recognition of the role of WM in normal brain functions and in disease, establishing a rigorous, data-driven, normative reference for WM is a pressing scientific need. Just as with paediatric growth charts, defining this standard of healthy maturation is a necessary prerequisite for identifying and quantifying atypical development and decline^11,12^.

dMRI provides a rich array of information on WM structure, including microstructural indices that reflect axonal density and dispersion^13^, as well as macrostructural properties such as WM tract volume, length and shape^14^. By integrating methodological advances in tract segmentation, data harmonization and statistical modelling that charts the full population distribution (for example, location, scale and skew) of inter-individual variability, we have established age- and sex-stratified trajectories of WM organization from early childhood to advanced ageing. By establishing standardized benchmarks for WM maturation and degeneration, we provide the foundational data to support discovery in fundamental neuroscience and for clinical translation, facilitating individualized assessments of brain health. These reference charts, along with openly accessible processing tools and harmonization pipelines, create a robust foundation for future research, large-scale collaborations and personalized approaches to studying WM alterations in health and disease.

We report our development of WM brain charts, a large-scale normative framework that documents changes in WM microstructure and macrostructure across the human lifespan. By integrating and analysing dMRI data spanning 50 different population studies, we have systematically characterized axonal density and dispersion, as well as tract volume, length and shape, in functionally relevant WM pathways, from early life to mature adulthood. These WM brain charts may be used in several important applications including: (1) defining normative trajectories of microstructural and macrostructural features of specific WM pathways; (2) revealing previously uncharacterized developmental milestones, refining our understanding of critical periods of WM growth and decline; (3) enabling sensitive detection of deviations from normative pathways, with implications for identifying early markers of neurodevelopmental and neurodegenerative disorders; and (4) enabling standardized centile scoring of new datasets to quantify WM alterations in clinical populations.

## Mapping normative WM growth

**Fig. 1:**
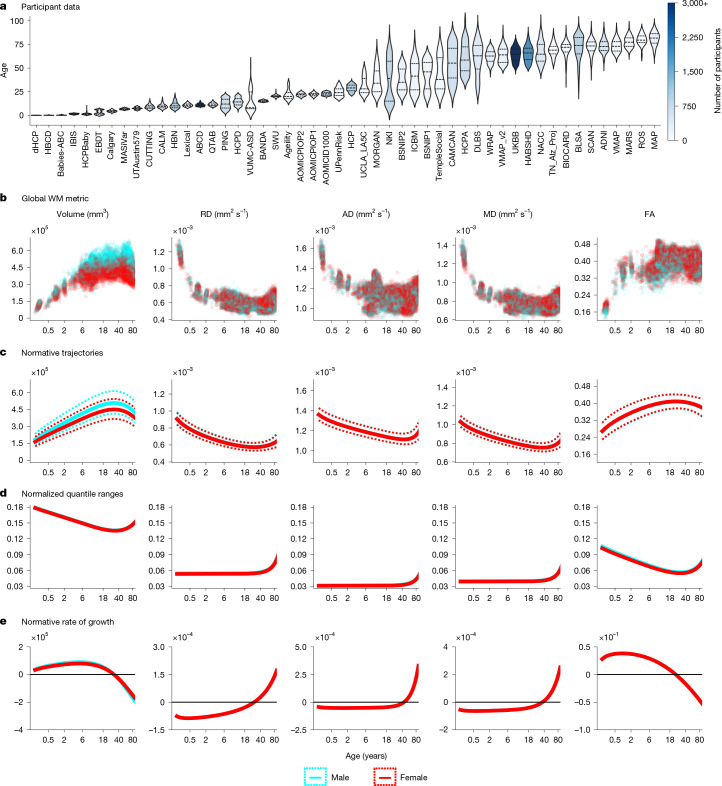
Global WM brain charts across the human lifespan. **a**, Data were aggregated from 50 imaging studies, comprising *n* = 35,120 scans from typically developing participants aged 0 to 100 years, forming the basis for the lifespan WM brain charts. Violin plots show the age distribution for each study coloured by the number of typically developing or ageing participants. **b**, Raw data points illustrate the distribution of global WM metrics (volume, FA, MD, AD and RD) across the lifespan for male (cyan) and female (red) participants. **c**, Normative trajectories for each metric, modelled using GAMLSS, show the median (solid lines) and 2.5th and 97.5th percentiles (dotted lines). **d**, Normalized quantile ranges (difference between 25th and 75th percentiles divided by the median) depict changes in population variability across the lifespan for each global WM feature. **e**, The normative rate of change (d/d(log(age))) for the median centile visualizes the velocity of WM development with age, highlighting that growth and decline are most rapid early in life and that different WM features reach their peak or minimum values at distinct ages. The horizontal line (*y* = 0) denotes critical points at which the derivative of the trajectories change sign. Note that *x* axes are log-scaled to emphasize developmental and ageing periods.

We included and analysed dMRI data from a cohort of 35,120 individuals spanning 50 population studies and including 4,253,251 imaging volumes, representing typical development and ageing with no known neurological or psychiatric conditions. We modelled both global and tract-specific WM phenotypes—including 72 anatomically defined pathways—using generalized additive models for location, scale and shape (GAMLSS)^15^, a flexible statistical framework endorsed by the World Health Organization for modelling non-linear biological trajectories^16^. GAMLSS enabled simultaneous estimation of age-dependent changes in location, scaling and skewness, while accounting for study-level batch effects. Microstructural (fractional anisotropy (FA), mean diffusivity (MD), axial diffusivity (AD) and radial diffusivity (RD)) and macrostructural (tract volume, length and surface area) features were modelled across the full lifespan (Fig. 1 and Extended Data Table 1; demographic information is presented in Supplementary Table S2.T1 and Supplementary Table SDT.T9).

Lifespan trajectories of global WM features revealed distinct, feature-specific patterns of growth and decline (Fig. 1b,c). Cerebral WM volume increased rapidly during early development, peaking at 34 years, followed by gradual atrophy in later life. FA exhibited a similar, though slightly earlier, inflection—increasing during childhood and adolescence, peaking around age 24, then steadily declining. By contrast, diffusivity measures (MD, RD and AD) demonstrated inverted trajectories: decreasing steeply during early life, reaching their lowest points in middle adulthood (RD at approximately 32 years, MD at approximately 40 years and AD at approximately 46 years), followed by progressive increases into older age. These divergent trajectories delineate critical inflection points in WM maturation and degeneration.

GAMLSS-estimated population variability revealed distinct age-related trends across WM phenotypes (Fig. 1d). Variability in metrics of diffusivity remained low or declined during early life, followed by substantial increases during mid-to-late adulthood. FA variability peaked during early childhood, declined steadily through midlife, and increased again in older age—suggesting age-dependent fluctuations in structural heterogeneity. Rates of change (Fig. 1e) were steepest during early childhood, reflecting rapid developmental transitions, and gradually diminished through late childhood and early adolescence (ages 6–12), mirroring the extended maturation period of myelination and axonal remodelling described in histological and neuroimaging studies^17–19^.

## Tract-specific WM phenotypes

**Fig. 2:**
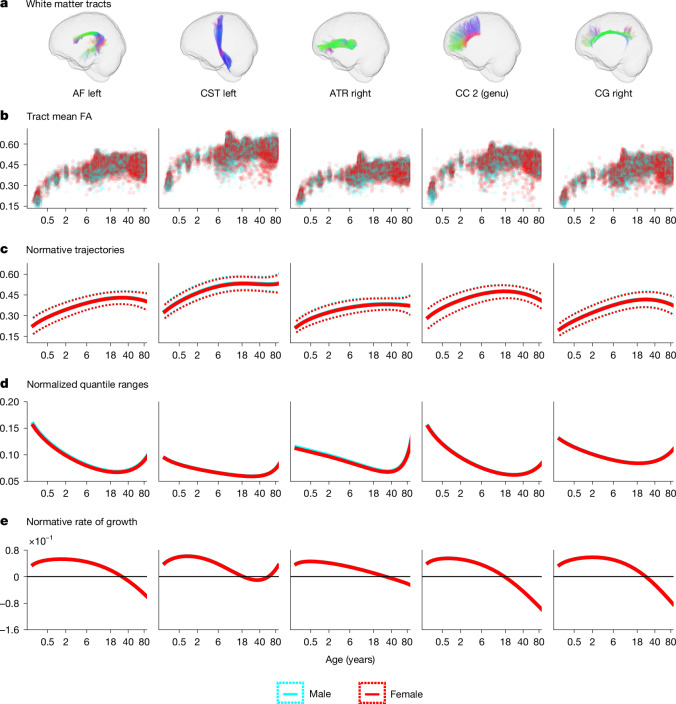
Tract-specific microstructural FA brain charts. Lifespan FA brain charts for WM microstructure (see Extended Data Figs. 1–3 for MD, AD and RD; see Supplementary Fig. S1.F1 for contralateral) reveal distinct developmental trajectories and variability across different WM pathways. **a**, Five exemplar tracts are shown: left arcuate fasciculus (AF left), left CST (CST left), right anterior thalamic radiation (ATR right), genu of the corpus callosum (CC 2 (genu)) and the right cingulum (CG right). Tracts are coloured by orientation, with blue indicating inferior–superior, red indicating left–right, and green indicating posterior–anterior. Graphs in **b**–**e** are aligned with the corresponding tracts. **b**, Raw FA data points for these tracts extend across the lifespan. **c**, Normative GAMLSS trajectories show FA increasing during development, plateauing in adulthood and declining in later life, where the timing and magnitude vary by tract. Median (solid lines) and 2.5th and 97.5th percentiles (dotted lines) are shown. **d**, Normalized quantile ranges indicate that FA variability is generally lowest in middle age and increases later in life, although developmental variability patterns differ between tracts. **e**, The normative rate of change (d/d(log(age))) for the median centile suggests that FA peaks at different ages and changes at different rates depending on the specific tract. Note that *x* axes are log-scaled to emphasize developmental and ageing periods.

**Fig. 3:**
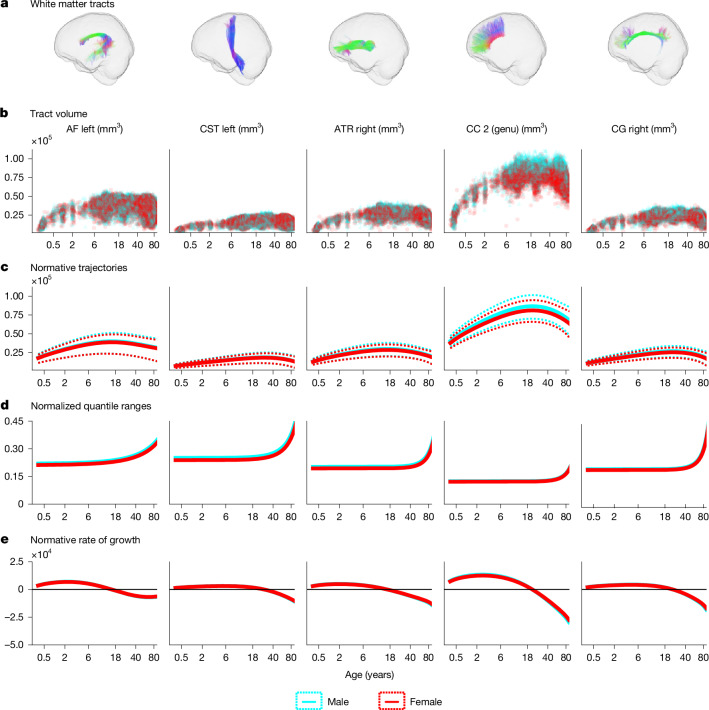
Tract-specific macrostructural volume brain charts. Lifespan brain charts of pathway volume for WM macrostructure (see Extended Data Figs. 4 and 5 for length and surface area and Supplementary Fig. S1.F2 for contralateral) reveal distinct developmental trajectories and variability across different WM pathways. **a**, Five exemplar tracts are shown: AF left, CST left, ATR right, CC 2 (genu) and CG right, as in Fig. 2a. Tracts are coloured by orientation, with blue indicating inferior–superior, red indicating left–right, and green indicating posterior–anterior. Graphs in **b**–**e** are aligned with the corresponding tracts. **b**, Raw tract volume data points for these tracts span the lifespan, illustrating differences in typical volume ranges between tracts. **c**, Normative GAMLSS trajectories show tract volume increasing during development, peaking in adolescence or early adulthood and declining in later life, with tract-specific timing and magnitude. Median (solid lines) and 2.5th/97.5th percentiles (dotted lines) are shown. **d**, Normalized quantile ranges indicate that volume variability increases later in life and differs between sexes, and is generally lowest in middle age. **e**, The normative rate of change (d/d(log(age))) for the median centile suggests that tract volume peaks at different ages and the rate of subsequent decline varies depending on the specific tract. Note that *x* axes are log-scaled to emphasize developmental and ageing periods.

**Extended Data Fig. 1:**
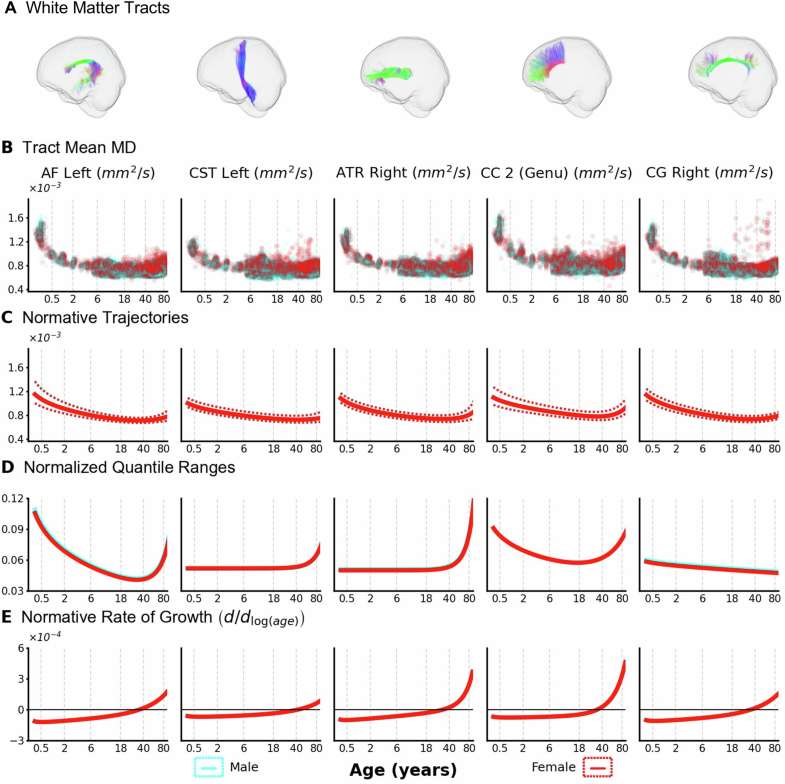
MD microstructural brain charts. Microstructural brain charts (MD) for five different WM tracts show tract-wise variability and varying developmental milestones across the human lifespan. A.) From left to right, left arcuate fasciculus; left corticospinal tract, right anterior thalamic radiation; genu of the corpus callosum; and the right cingulum. B.) Raw datapoints span the entirety of the lifespan at high density. C.) Most normative trajectories of MD for different pathways decrease in value during development, reach a minimum in adulthood, and then increase at different rates in aging. For all pathways represented, the male trajectories (blue) sit at nearly equal values to the female trajectories (red). D.) Normalized quantile differences indicate that microstructural variability in MD increases at the end of the human lifespan and is lowest in middle age. Variability in MD during development appears to be tract dependent, with some tracts showing lowest variability in infancy and others decreasing to a minimum in adulthood. E.) The normative rate of change ($d/{d}_{\log ({age})}$) of MD for the represented tracts. MD of tracts reach minima at different ages in the lifespan, indicating tract-specific developmental patterns.

**Extended Data Fig. 2:**
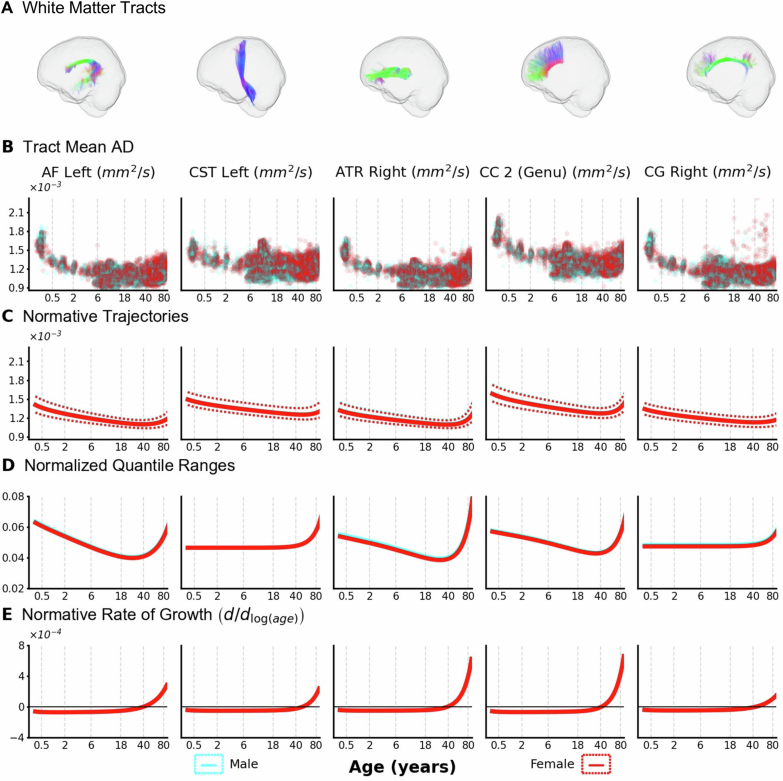
AD microstructural brain charts. Microstructural brain charts (AD) for five different WM tracts show tract-wise variability and varying developmental milestones across the human lifespan. A.) From left to right, left arcuate fasciculus; left corticospinal tract, right anterior thalamic radiation; genu of the corpus callosum; and the right cingulum. B.) Raw datapoints span the entirety of the lifespan at high density. C.) Normative trajectories of AD for different pathways decrease in value during development, reach a minimum in adulthood, and then increase at different rates in aging. For all pathways represented, the male trajectories (blue) sit slightly above the female trajectories (red). D.) Normalized quantile differences indicate that microstructural variability in AD increases at the end of the human lifespan and is lowest in middle age. Variability in AD during development appears to be tract dependent, with some tracts showing lowest variability in infancy and others decreasing to a minimum in adulthood. E.) The normative rate of change ($d/{d}_{\log ({age})}$) of AD for the represented tracts. AD of tracts reach minima at different ages in the lifespan, indicating tract-specific developmental patterns.

**Extended Data Fig. 3:**
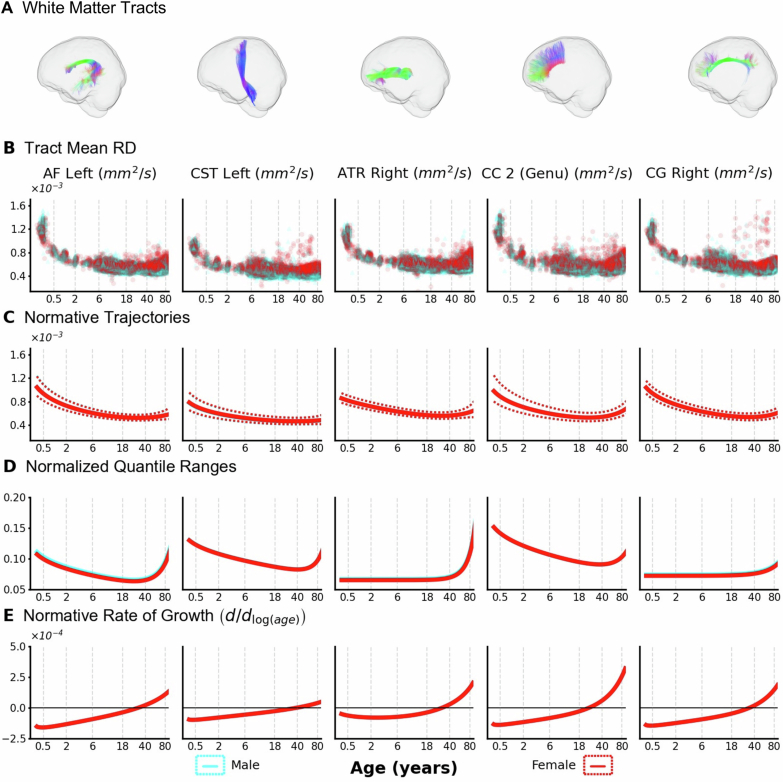
RD microstructural brain charts. Microstructural brain charts (RD) for five different WM tracts show tract-wise variability and varying developmental milestones across the human lifespan. A.) From left to right, left arcuate fasciculus; left corticospinal tract, right anterior thalamic radiation; genu of the corpus callosum; and the right cingulum. B.) Raw datapoints span the entirety of the lifespan at high density. C.) Normative trajectories of RD for different pathways typically decrease in value during development, reach a minimum in adulthood, and then increase at different rates in aging. For all pathways represented, the female trajectories (red) sit slightly above the male trajectories (blue). D.) Normalized quantile differences indicate that microstructural variability in RD increases at the end of the human lifespan and is lowest in middle age. RD variability appears to be tract dependent, with some tracts showing lowest variability in infancy and others increasing steadily across the lifespan. E.) The normative rate of change ($d/{d}_{\log ({age})}$) of RD for the represented tracts. RD of tracts reach minima at different ages in the lifespan, indicating tract-specific developmental patterns.

**Extended Data Fig. 4:**
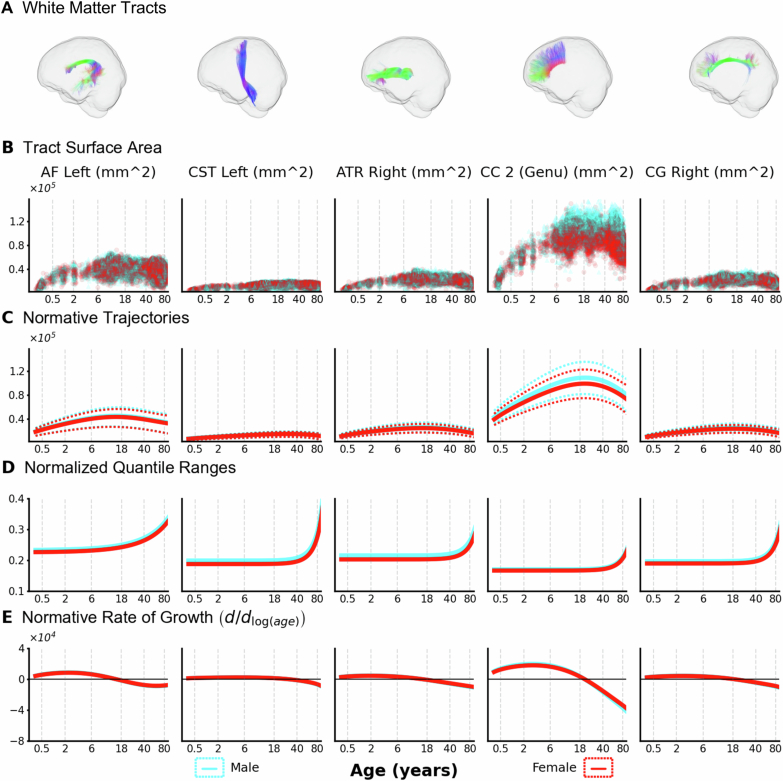
Surface area macrostructural brain charts. Macrostructural brain charts (surface area) for five different WM tracts show tract-wise variability and varying developmental milestones across the human lifespan. A.) From left to right, left arcuate fasciculus; left corticospinal tract, right anterior thalamic radiation; genu of the corpus callosum; and the right cingulum. B.) Raw datapoints span the entirety of the lifespan at high density. C.) Normative trajectories of surface area for different pathways increase in value during development, reach a maximum in adulthood, and then decrease at different rates in aging. For all pathways represented, the male trajectories (blue) sit slightly above the female trajectories (red). D.) Normalized quantile differences indicate that macrostructural variability in tract surface area increases at the end of the human lifespan and is lowest in middle age. Variability in the male trajectories is consistently greater than that of the female trajectories. E.) The normative rate of change ($d/{d}_{\log ({age})}$) of surface area for the represented tracts. Surface area of tracts reach maxima at different ages in the lifespan, indicating tract-specific developmental patterns.

**Extended Data Fig. 5:**
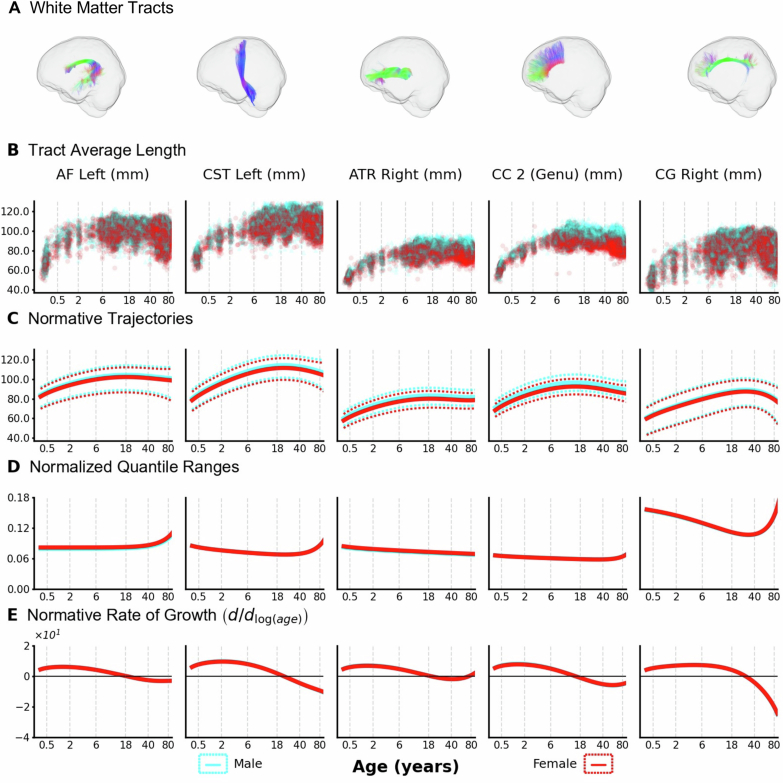
Average length macrostructural brain charts. Macrostructural brain charts (average length) for five different WM tracts show tract-wise variability and varying developmental milestones across the human lifespan. A.) From left to right, left arcuate fasciculus; left corticospinal tract, right anterior thalamic radiation; genu of the corpus callosum; and the right cingulum. B.) Raw datapoints span the entirety of the lifespan at high density. C.) Normative trajectories of average length for different represented pathways increase in value during development, reach a maximum in adulthood, and then decrease at different rates in aging. For all pathways represented, the male trajectories (blue) sit slightly above the female trajectories (red). D.) Normalized quantile differences indicate that macrostructural variability is very tract-dependent. Some tracts have the highest variability at the beginning of the lifespan and lowest at the end, whereas others have the opposite trend. The change in variability of average length across the lifespan also appears to be tract dependent, with some tracts decreasing somewhat consistently and others showing sharp increases. E.) The normative rate of change ($d/{d}_{\log ({age})}$) of average length for the represented tracts. Average length of tracts reach maxima at different ages in the lifespan, indicating tract-specific developmental patterns.

To move beyond global cerebral measures and characterize regional heterogeneity, we applied the GAMLSS framework to derive tract-specific normative trajectories for WM microstructural and macrostructural features across the lifespan. We estimated trajectories for 72 anatomically defined WM pathways, capturing the full spectrum of association, commissural, projection, thalamic and limbic systems (see Extended Data Table 2 for a list of tracts and abbreviations). Representative trajectories are shown for five exemplar tracts: the arcuate fasciculus and cingulum bundle (association), corticospinal tract and anterior thalamic radiation (projection), and genu of the corpus callosum (commissural) for microstructural phenotypes (Fig. 2 and Extended Data Figs. 1–3; Supplementary Fig. S1.F1 for contralateral) and macrostructural phenotypes (Fig. 3 and Extended Data Figs. 4 and 5; Supplementary Fig. S1.F2 for contralateral; Supplementary Figs. S1.F3–S1.F11 for trajectories of normalized measures). Selection of visualized tracts is based on prevalence of study in the literature^20^ (and when displayed in figures are coloured to highlight directionality, with red, green and blue indicating left–right, anterior–posterior and superior–inferior orientations, respectively)^21^.

Tract-wise trajectories of FA revealed consistent developmental phases but variable temporal profiles across pathways (Fig. 2). FA increased rapidly during infancy and childhood, reached a plateau in early adulthood, and declined gradually in later life. Yet the magnitude and timing of these inflection points varied substantially by tract. For instance, the corticospinal tract exhibited a prolonged plateau of high anisotropy into midlife, and the genu of the corpus callosum and cingulum bundle reached peak FA earlier, followed by earlier declines. Inter-individual variability in FA followed a similar pattern, remaining low and stable during early development, then increasing with age in a tract-dependent manner. First-derivative estimates highlighted that FA change rates were steepest in early development but differed in timing and slope across pathways.

Macrostructural trajectories of tract volume followed characteristic inverted U-shaped curves (Fig. 3), with rapid volumetric expansion during early development, peaks occurring in adolescence or early adulthood, and progressive atrophy in older age. As with microstructure, these patterns were highly tract-dependent, with marked differences in peak timing (Supplementary Fig. S4.F8) and slope of decline (Supplementary Table SDT.T9). Inter-individual variability in volume increased with age, and the steepest rates of volumetric change occurred during early childhood. However, the timing and magnitude of these dynamics varied across tracts, reflecting anatomical and developmental diversity in WM maturation and degeneration. Furthermore, although sex differences in microstructural measurements are less pronounced than in macrostructure, we still observe significant effects for both (Supplementary Fig. S4.F6).

Together, these tract-specific brain charts delineate the heterogeneity of WM development and ageing, capturing normative ranges, variability and rates of change across anatomically distinct pathways. This reference framework enables precise interpretation of age-related trajectories at the pathway level and establishes a foundation for identifying atypical patterns in both developmental and clinical contexts.

## WM developmental milestones

**Fig. 4:**
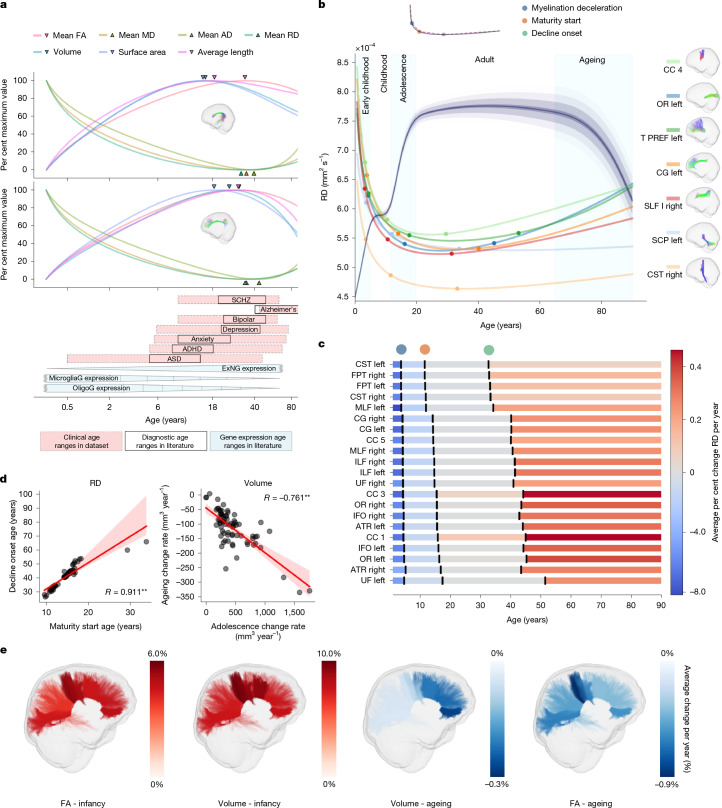
Tract-specific developmental milestones, neurobiological patterns and spatial gradients. Trajectories reveal developmental milestones, enabling investigation of neurobiological hypotheses and spatio-temporal microstructural dynamics. **a**, Median centile trajectories for all features of the left arcuate fasciculus (top) and the right cingulum (middle) show pathway-specific developmental maturation as peaks (downward triangles) or troughs (upward triangles). Bottom, a graphical summary situates these trajectories alongside non-MRI milestones. Pink boxes denote the age range for clinical disorders in the dataset; black boxes denote literature diagnostic ranges^22^; blue tapered bars indicate transcriptomic development windows^23^. ADHD, attention-deficit hyperactivity disorder; ASD, autism spectrum disorder; SCHZ, schizophrenia; ExNG, excitatory neuron gene; MicrogliaG, microglia gene; OligoG, oligodendrocyte gene. **b**, Median RD trajectories for exemplar tracts illustrate waves of development and late-life change (waves identified by piecewise segmentation of lifespan curves (top inset)). These in vivo patterns are consistent with proposed myelin development and ageing patterns^25^ based on foundational histological work from Yakovlev and Lecours^18^. Myelin changes are visualized qualitatively as the purple curve (adapted from ref. ^25^, Springer Nature). OR, optic radiation; SCP, superior cerebellar peduncle; SLF, superior longitudinal fascicle; T PREF, thalamic prefrontal. **c**, Relative timing and magnitude of change for RD trajectories across selected tracts. Vertical black lines represent the identified myelination time points (for example, rapid early myelin increase, maturity start and decline onset). Bar colour represents average annual per cent change during life epochs, revealing tract-specific variability in rates of change. IFO, inferior occipito-frontal fascicle; ILF, inferior longitudinal fascicle; MLF, middle longitudinal fascicle. **d**, Neurobiological hypothesis tests derived from tract milestone timings (left) and rates (right), with each datapoint representing one tract. Left, there is a positive relationship between age at feature maturity and age at decline onset for RD. Right, greater adolescent growth in macrostructure tract volume predicts steeper decline with ageing (that is, gain predicts loss; slope–slope relation). ** indicates significance of linear regression under Bonferroni correction (*P* < 0.0035; the shaded area represents 95% confidence intervals; additional feature regressions are shown in Supplementary Fig. S4.F9). **e**, Spatial organization. Anterior-to-posterior gradients emerge in rates of change across tracts (both microstructure and macrostructure), indicating regionally patterned maturation and decline.

We extracted tract-resolved milestones from lifespan centile trajectories by locating critical points (local extrema) for microstructural and macrostructural features (Fig. 4a). Examples from the left arcuate fasciculus and right cingulum illustrate two points that generalize: (1) feature-specific timing differs within a tract; and (2) timing differs across tracts. Across pathways, macrostructural metrics (length, surface area and volume) typically peak earlier than microstructural metrics (Supplementary Fig. S4.F8). The bottom context panel orients the trajectories within external timelines—marking cohort age coverage, literature-based diagnostic ages for the major disorders represented^22^ and developmental transcriptomic windows^23^—highlighting periods of pronounced gene-expression change and clinical vulnerability.

We then used the charts to investigate in vivo microstructural changes that reflect myelination. To relate milestones to myelination, we used RD^24^ as a myelin-sensitive proxy and segmented each RD trajectory piecewise (Methods, ‘WM lifespan milestones’). This captures the early life decrease (consistent with progressive myelination) and late-life increases in RD, with tract ordering that mirrors classical staging^18,25,26^: earlier for projection systems (for example, corticospinal tract (CST) and frontopontine tract (FPT)) and later for association pathways (for example, uncinate fasciculus (UF))^27,28^ (Fig. 4b,c). These patterns are in line with the established view that human myelination is protracted into mid-adulthood and proceeds in non-simultaneous waves across systems rather than as a single schedule^18,29^. We summarize the heterogeneity in timing and dynamics across pathways (Fig. 4c), reporting ages of key RD time points intended to reflect distinct waves of myelination^25^ (end of early rapid myelination, maturation throughout adolescence and onset of myelin decline) alongside the rates of change between each time point. We emphasize RD as a sensitive, but non-specific in vivo proxy that can reflect myelination as well as other microstructural factors^13^.

Because the charts yield tract-specific milestones, they also enable quantitative assessment of links between maturation and later life change (Fig. 4d). Under a retrogenesis framework^30^, we evaluated two tract-level hypotheses derived from centile trajectories. First (based on relative timings), the ‘last-in, first-out’ hypothesis predicts that later maturing pathways show earlier onset of decline^31^; operationally, we related the age of each tract at feature maturity to the age at which decline begins (illustrated for RD). We did not find evidence supporting the last-in, first-out hypothesis in these timing estimates; instead, later maturing pathways tended to show later onset of decline. Second (based on relative rates of change), the ‘gain-predicts-loss’ hypothesis suggests that pathways with larger developmental gains should show steeper ageing-related losses^17^; operationally, we related adolescent growth rates to later life decline rates (illustrated for tract volume). Macrostructural trajectories were consistent with this hypothesis, with faster adolescent volumetric expansion being associated with steeper volumetric decline in ageing (parallel regressions for additional WM features shown in Supplementary Fig. S4.F9). Together, these results indicate that centile trajectories capture systematic, feature-dependent couplings between maturation and later life change.

Finally, the charts localize spatial organization of WM change^32^ (Fig. 4e). We summarize the mean rate of change for FA and volume in infancy and ageing along the sections of the corpus callosum. During infancy, microstructural rates show an anterior–posterior ‘inside-out’ trend, with relatively slower change at the most anterior and posterior pathways and faster change in intermediate systems. During ageing, front-to-back volumetric differences emerge with stronger anterior declines. These gradients differ by feature and life stage and are not a mirror image of development, indicating that retrogenesis provides a partial, but incomplete account of the spatial organization of change.

These results collectively define a fine-grained atlas of WM developmental milestones. The timing, variability and rates of change in both microstructural and macrostructural features differ substantially across pathways, offering a comprehensive reference for interpreting normative WM maturation and age-related decline. This framework supports future efforts to investigate how developmental timing relates to functional specialization, vulnerability to disease and lifespan trajectories of brain structure.

## Individualized tract centile scores

**Fig. 5:**
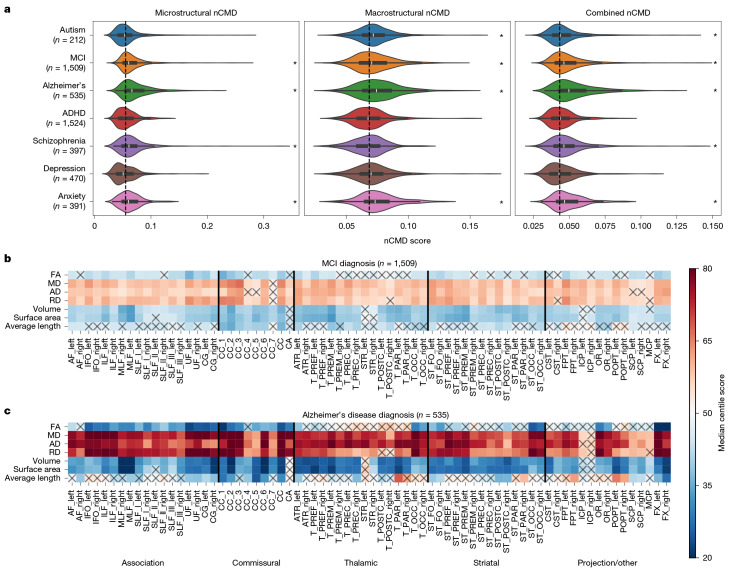
Case–control differences quantified by WM centile scores. Deviations from normative WM trajectories, measured using individualized centile scores (ranging 0–100, with 50 being the population median), reveal significant differences between typically developing controls and patient populations with diverse neurological conditions. **a**, Violin plots show distributions of the nCMD scores, an aggregate measure of deviation across all tracts, for microstructural, macrostructural and combined features in several diagnostic groups. Internal box plots show the median (central white line), with the bounds of the box representing 25th and 75th percentiles (interquartile range (IQR)) and whiskers extending to the maxima and minima within 1.5× IQR. Higher nCMD scores indicate greater overall deviation from the normative control population (compared against median nCMD for typically developing and ageing participants, shown as vertical dashed lines). Significant differences from controls (Bonferroni-corrected two-sided one-sample Wilcoxon signed-rank test) are marked with an asterisk, notably for MCI, Alzheimer’s disease and anxiety groups across all aggregated measures (significant at *P* < 7.4 × 10^−3^). **b**,**c**, Heat maps display the median centile score for each WM feature across all 72 tracts for individuals diagnosed with MCI (**b**) and Alzheimer’s disease (**c**). Colours represent the median centile relative to the median of the control (blue < 50, red > 50). Widespread deviations are evident, particularly lower FA and volume as well as higher diffusivities in MCI and Alzheimer’s disease. Crosses indicate features for which the median centile was not significantly different from 50 after Bonferroni correction under a two-sided one-sample Wilcoxon signed-rank test (significant at *P* < 9.92 × 10^−5^). CA, anterior commissure; FX, fornix; ICP, inferior cerebellar peduncle; MCP, middle cerebellar peduncle; POPT, parieto-occipito-temporal; STR, superior thalamic radiation; ST_FO, striato-fronto-orbital; ST_OCC, striato-occipital; ST_POSTC, striato-postcentral; ST_PREC, striato-precentral; ST_PREF, striato-prefrontal; ST_PREM, striato-premotor; ST_PAR, striato-parietal; T_OCC, thalamic-occipital; T_PAR, thalamic-parietal; T_POSTC, thalamic-postcentral; T_PREC, thalamic-precentral.

We computed individualized centile scores to benchmark WM measurements of each participant against normative age- and sex-stratified trajectories (Methods, ‘Centile scores and case–control differences’). These scores quantify the relative typicality or atypicality of microstructural and macrostructural features on a unified scale, with values near the extremes (0th or 100th percentile) reflecting substantial deviation from the normative population. Leveraging a clinically diverse dataset, we applied this framework to systematically examine case–control differences across several diagnostic groups, including neurodevelopmental, neurocognitive and neurodegenerative disorders (Fig. 5 and Extended Data Table 3).

To summarize deviations across multiple features and tracts, we computed a normalized centile Mahalanobis distance (nCMD) for each individual, capturing aggregate atypicality within microstructural, macrostructural and combined feature spaces (Methods, ‘Centile scores across cognitive groups’). Across diagnostic groups, nCMD values were consistently higher relative to controls (Fig. 5a; see Supplementary Table S3.T1 for effect sizes). This composite score enabled dimensionality reduction while preserving individual variation, revealing disorder-specific patterns—some of which were characterized primarily by microstructural deviation, whereas others were characterized by macrostructural or mixed profiles.

**Extended Data Fig. 6:**
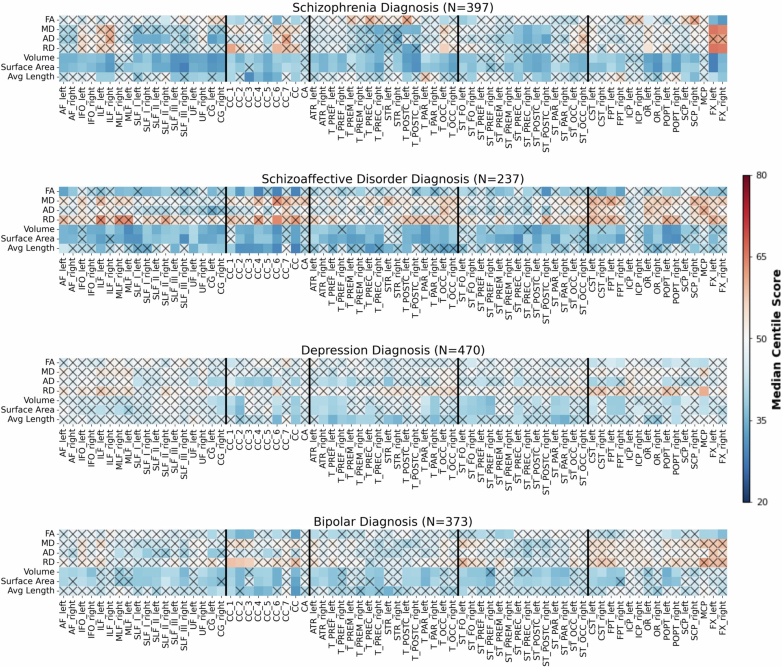
Centile score differences across major depressive and psychotic disorders. All major psychoses and major depressive disorder (top to bottom: schizophrenia, schizoaffective disorder, major depressive disorder, and bipolar disorder) showed localized reductions in tract macrostructure and microstructure under the one-sample two-sided Wilcoxon signed-rank test after Bonferroni correction for multiple comparisons. Gray “X” indicates features where the median centile was not significantly different from the 50^th^ normative centile (significant at $p < 9.92\times {10}^{-5}$).

**Extended Data Fig. 7:**
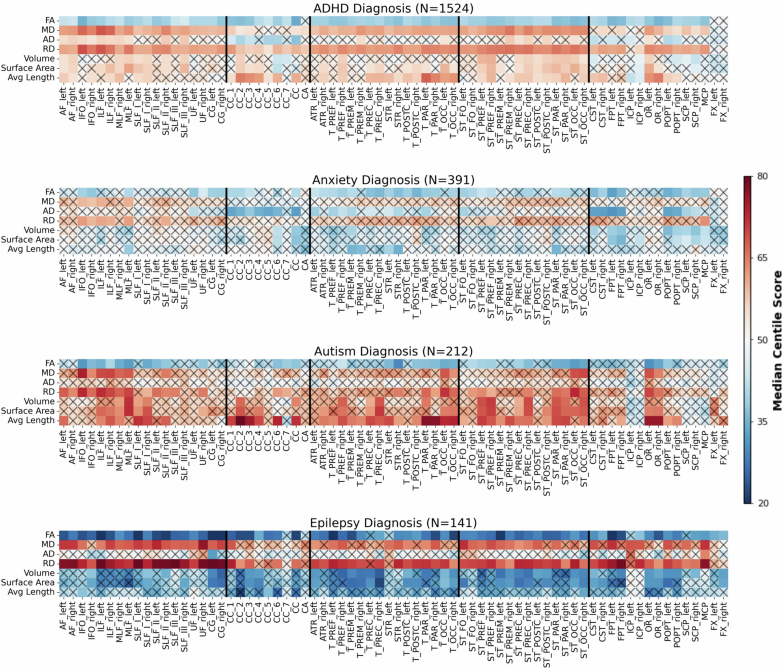
Centile score differences across other neurocognitive disorders with significant deviations. For many other clinical cohorts (top to bottom: attention-deficit hyperactivity disorder (ADHD), anxiety, autism spectrum disorder, and epilepsy), we observe either localized deviations in tract microstructure and macrostructure or consistent deviations across all tracts within specific macrostructural and microstructural features. Individuals with epilepsy and ADHD showed substantial deviations in FA and RD across the WM, suggesting diffuse microstructural abnormalities, alongside more localized reductions in volume, length, and surface area in select tracts. For individuals diagnosed with autism spectrum disorder, we observe increases in macrostructure across tracts, with a subset of tracts showing significant deviations. Finally, we observe sparsely significant deviations in microstructure and macrostructure for individuals diagnosed with anxiety. Gray “X” indicates features where the median centile was not significantly different from the 50^th^ normative centile under a two-sided one-sample Wilcoxon signed-rank test following Bonferroni correction for multiple comparisons (significant at $p < 9.92\times {10}^{-5}$).

Significant deviations from normative centile scores were also observed for specific tract microstructure and macrostructure measurements. Diagnostic groups of mild cognitive impairment (MCI) and Alzheimer’s disease showed significant deviations across most tracts and features compared with neurotypical controls (Fig. 5b,c; see Extended Data Figs. 6 and 7 and Supplementary Fig. S3.F1 for other diagnostic groups, Supplementary Figs. S3.F3–S3.F5 for alternative false discovery rate with Benjamini–Hochberg correction and Supplementary Figs. S3.F6–S3.F8 for effect sizes). MCI was associated with widespread increases in diffusivity, reductions in FA and lower tract volumes and surface areas—changes that were even more pronounced in Alzheimer’s disease.

These findings demonstrate that individualized centile scores offer a sensitive, interpretable framework for quantifying WM atypicality—capturing both global deviations and pathway-specific abnormalities across diverse neurological diagnostic groups.

## Centile scoring of new datasets

**Fig. 6:**
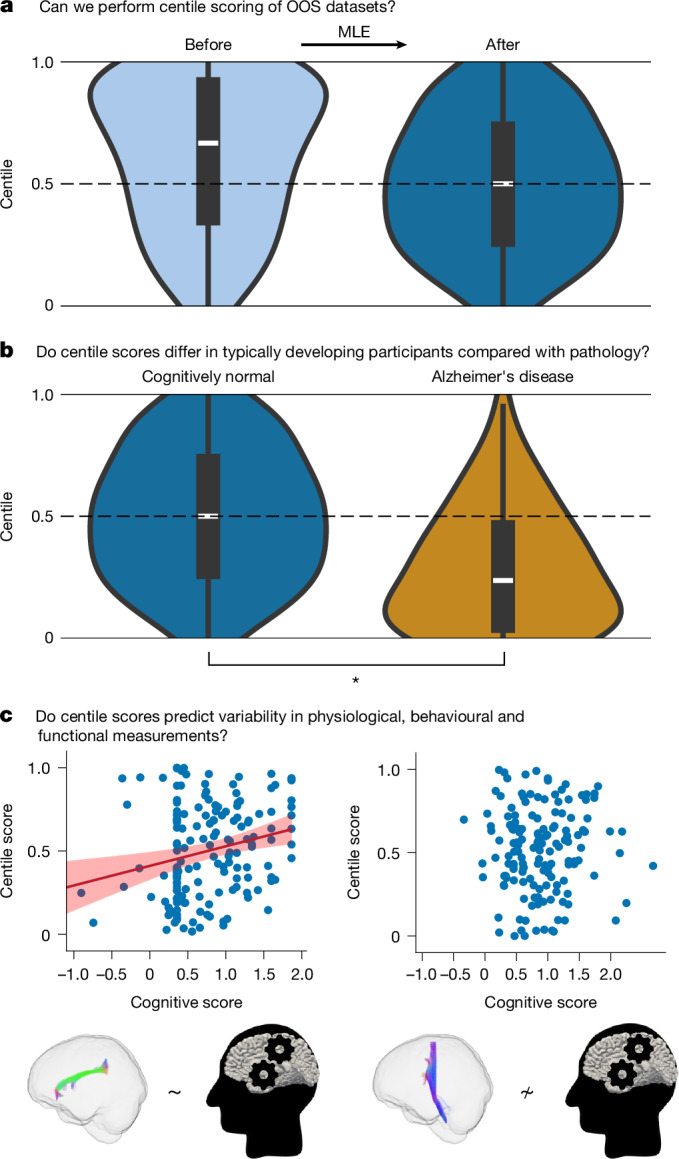
Applications of OOS centile scoring. Normative WM brain charts enable standardized centile scoring for new OOS datasets, facilitating harmonized analyses. Exemplar applications are demonstrated using the Alzheimer’s Disease Neuroimaging Initiative (ADNI) dataset (withheld from the models used for this figure for demonstration purposes). **a**, OOS datasets can be aligned to the reference trajectories using MLE on typically developing or ageing participants to estimate study-specific offsets. This process yields a correctly centred centile distribution for OOS controls (*n* = 591 for the mean FA of CC 1). Centile values before and after alignment are shown using a violin plot visualization with internal box plots showing the median (central white line), with the bounds of the box representing the IQR and whiskers extending to the maxima and minima within 1.5× IQR. The 50th centile is represented by a dotted black line. We recommend a sample size of *n* ≥ 100 for alignment based on empirical assessments (see Supplementary Fig. S8.F1). **b**, Aligned centile scores allow for standardized comparisons between diagnostic groups within the OOS dataset, illustrated here comparing typically developing and ageing individuals (cognitively normal, *n* = 591) and those with Alzheimer’s disease (*n* = 212). Asterisk denotes significance under a two-sided Mann-Whitney *U* test, with *P* < 0.05. **c**, Aligned centile scores serve as standardized metrics for investigating relationships with external variables; here we provide an illustrative cognition-specific example. Executive function (composite score^43^) is significantly correlated with aligned FA centiles in the right SLF III (left; *n* = 160), but not the right CST (right; *n* = 151) for cognitively normal participants, highlighting pathway-specific associations. Significance was assessed for the centile–executive function association using a least-squares linear regression. Significance (*P* < 6.94 × 10^−4^ following Bonferroni correction) of the association is indicated with a red regression line and 95% confidence intervals.

A central challenge in applying normative brain charts is estimating individualized centile scores for new, out-of-sample (OOS) datasets that are not included in the original reference cohort. To address this, we implemented a maximum likelihood estimation (MLE) framework to align external datasets to existing normative trajectories on a feature and pathway-specific basis (Fig. 6 and Methods, ‘OOS centile estimation’). This alignment enables accurate and interpretable centile scoring, thereby extending the utility of WM brain charts to new datasets for studying both typical and pathological brain structure.

As an initial validation, we estimated study-specific offsets using cognitively healthy controls from a held-out dataset, yielding a near-uniform centile distribution centred around the population median (50th centile; Fig. 6a). This enabled two key applications in a new Alzheimer’s disease cohort. First, aligned centile scores provided a consistent framework for evaluating case–control differences, revealing specific WM features and pathways that significantly diverged in Alzheimer’s disease relative to controls (Fig. 6b). Second, the centile framework enabled associations between WM structure and cognitive performance to be examined, identifying pathway-specific features that were strongly linked to clinical variability (Fig. 6c). In this way, the reference charts can be used to relate abnormality indices to a broad range of external clinical data.

Together, these findings demonstrate that centile scoring of OOS datasets provides a rigorous and generalizable framework for evaluating group differences, identifying pathway-specific deviations and exploring structure–function relationships. By grounding these analyses in normative WM trajectories, our approach enables biologically interpretable comparisons across diagnostic groups and datasets (see Supplementary Information, section 8 for how to perform alignment with an OOS dataset).

## Discussion

Here we present normative brain charts of WM microstructure and macrostructure across the human lifespan, derived from 35,120 dMRI scans across 50 harmonized cohorts. These charts establish normative references for both global and tract-specific WM properties, enabling age and sex-stratified benchmarking of WM development, ageing and pathology. Our analyses delineate normative trajectories, identify tract-specific growth milestones and demonstrate individualized quantification of WM deviations with applications to diagnostic groups. By addressing prior methodological and dataset limitations, this work marks a critical step toward standardized, lifespan quantification of WM organization.

First, our WM brain charts define robust, sex-stratified trajectories for both microstructural and macrostructural properties across 72 WM pathways. This includes canonical diffusion metrics (FA, MD, AD and RD) and tract-specific morphometric features such as volume, length and surface area. Prior lifespan studies have focused largely on grey matter, and established normative models are now available for cortical and subcortical volumes (including BrainChart^6^, NiChart^9^ or CentileBrain^7,8^ platforms). Whereas recent efforts have created normative models for WM microstructure based on regional diffusion metrics^33,34^, our work extends these frameworks by incorporating both microstructural and macrostructural measures across 72 anatomically defined pathways and providing a more granular and comprehensive view of WM development, ageing and pathology. Our results show that WM pathways can be reliably characterized across development and ageing using harmonized tractography pipelines. Although diffusion tensor imaging (DTI)-derived microstructure has been well-studied^28,33,35^, tract-level macrostructural features have received limited attention^27^. Here we demonstrate their value and provide intuitive metrics, such as tract volume, average length and surface area, in both unnormalized and brain-size-normalized forms. Together, these benchmarks enable systematic characterization of WM maturation and support broader applications in developmental neuroscience and clinical research.

Second, our normative charts reveal pathway-specific developmental milestones, capturing the diversity in timing and sequence of maturation and decline across WM tracts. These differences reflect both functional specialization and distinct temporal dynamics of WM plasticity, preservation and degeneration^20^. Furthermore, our normative charts agree with foundational^18^ and more recent works of myelin development across the lifespan, not only with the timings of myelination waves, but also with the relative myelination timings of certain WM tracts^25^. The open-access trajectories generated here support testing of neurobiological theories^17,31^ such as the last-in, first-out hypothesis, where later-maturing pathways are more susceptible to early ageing, and the gain-predicts-loss hypothesis, which posits that regions undergoing rapid developmental expansion are prone to steeper age-related decline. The observed dissociation between microstructural and macrostructural inflection points further underscores the complex, feature-dependent nature of WM development, inviting future work into the temporal interplay of structural metrics across the lifespan.

Third, we demonstrate that normative WM brain charts derived from typically developing and ageing individuals with no known neurological or psychiatric conditions provide a sensitive and interpretable framework for detecting individualized anomalies in WM structure. Microstructural and macrostructural aberrations in cerebral WM have been implicated in numerous neurological disorders, as referenced in this work. Although we have been able to measure many of these parameters for over a decade, the lack of a reliable robust reference atlas has limited our ability to leverage these measurements as a tool for diagnosis and prognosis on an individual patient level. If we hope to utilize these measurements in a clinically productive manner, it is imperative to establish a readily available normative atlas of WM structural characteristics. Just as with any blood or body fluid laboratory test, a benchmark of normal values, ideally in a demographically similar population, is critical to interpreting the result in an individual patient. By providing a normative baseline, a single snapshot in time can be used to determine whether an individual exhibits an atypical phenotype relative to their age and sex. Crucially, the GAMLSS framework moves beyond solely a median trajectory and explicitly models the full population distribution (including its location, scale and shape), allowing quantification of inter-individual variability. By quantifying deviations from these distributions using centile scores, we identified distinct patterns of microstructural and macrostructural atypicality in individuals across a variety of diagnostic groups (Fig. 5, Extended Data Figs. 6 and 7 and Supplementary Figs. S3.F1–S3.F5). These results highlight the utility of centile-based assessments for mapping disease-related alterations at the level of specific tracts and features. Although mechanistic inferences are beyond the scope of this study, the sensitivity of this framework to detect subtle and widespread WM abnormalities underscores its potential for clinical applications in both diagnosis and tracking of neurological disorders.

Finally, our framework enables standardized effect size estimation and tract-level quantification of WM deviation in OOS datasets. By aligning new data to normative trajectories using maximum likelihood estimation, we enable centile scoring even in external cohorts collected across disparate sites and protocols. This facilitates cross-cohort comparisons, phenotype-feature associations and broader reproducibility across neuroimaging studies. As an illustrative example with Alzheimer’s disease cohorts, these aligned centile scores provide a robust foundation for exploring the relationship between WM features and clinical or cognitive outcomes. We note that as the brain charts were constructed with typically ageing and developing individuals, aligning OOS datasets requires some typically ageing and developing individuals in the OOS dataset as well (see Supplementary Information, ‘OOS alignment analysis’).

Several limitations warrant consideration. Although the aggregated dataset is among the largest to date, age distribution was uneven, with relative under-representation of infancy and mid-adulthood (Supplementary Fig. S2.F2). To maintain the statistical power of our normative reference model, we did not include phenotypes beyond sex (for example, ethnicity or genotype status) owing to limited data availability and reduced sample size (Supplementary Table S2.T1). Further, normative trajectories were derived from cross-sectional data; future longitudinal validation will be important, as individual trajectories can differ from population-level trends^36^. Our cross-sectional approach was chosen to ensure statistical power and model stability for OOS alignment, as detailed in Supplementary Information, ‘Considerations for cross-sectional versus longitudinal brain charts’. Although there was data representation from several continents, the datasets used to create the brain charts are predominantly from European and North American populations (Supplementary Table S2.T2). This bias is unfortunately common in neuroimaging and genetics, echoing limitations that are found even in foundational clinical references such as the US Centers for Disease Control and Prevention and World Health Organization growth charts^12^. This highlights a critical need for the global scientific community to increase ethnic, socioeconomic and demographic diversity in future MRI research. In our assessments relating developmental and ageing milestones for myelination and other properties, we note there are limitations in defining discrete developmental time points across continuous curves. Tractography-based metrics are inherently sensitive to preprocessing parameters and may be less reliable in certain populations or developmental stages^10,37^. Future work may also include alternative bundle segmentation methods^14^, with varying levels of specificity and sensitivity^38^, alternative definitions and interpretations of pathways, or under-investigated pathways of the brainstem or short association fibres^26^. Although we utilized conventional DTI for microstructural features, alternative multicompartment diffusion models may offer increased biological specificity^39,40^. Finally, we caution that these WM brain charts are not immediately suitable for the quantitative diagnosis of individual patients in a clinical setting. Substantial caveats exist even for the diagnostic interpretation of traditional anthropometric growth charts^41^. Similarly, considerable future research and validation will be necessary to translate these WM charts from a foundational research framework into a clinically validated diagnostic tool.

In conclusion, we have created comprehensive WM brain charts that define normative microstructural and macrostructural properties across the human lifespan, and have demonstrated their utility in identifying developmental milestones, detecting abnormalities in clinical populations and assessing relationships with cognitive and clinical variables. By openly sharing the underlying trajectories, scoring tools and harmonization pipelines, we aim to accelerate research into the structural basis of cognition, disease and ageing. This work lays the foundation for a new class of precision neuroscience tools grounded in normative WM architecture. The WM brain charts and code for aligning OOS datasets have been made available for researchers at https://zenodo.org/records/15367425 (ref. ^42^).

## Methods

### Ethics

The research consists of secondary analyses of de-identified primary datasets. Information regarding informed consent of participants (or guardians) in primary studies can be found in the references in Supplementary Table S2.T2. Secondary analysis of these data was also approved by the Vanderbilt University Medical School Institutional Review Board (IRB no. 210968).

### Software reporting

All analysis visualizations and statistics were generated using Python v3.9.20. Notable Python libraries used are pandas (v2.2.3), numpy (v2.0.2), seaborn (v0.13.2), matplotlib (v3.9.2), rpy2 (v3.5.11) and scipy (v1.13.1). For creating 3D qualitative visualizations of WM tracts, we used the mrview tool inside the MRtrix3 (version 3.0.4). Alternatively, for qualitative region of interest 3D visualizations, the OpenDIVE visualization library (v0.3.0) was used with a different Python (v3.10.18) for library compatibility purposes. Regarding the image preprocessing and processing software, please refer to ‘MRI processing pipeline’.

### Data

We aggregated diffusion-weighted imaging (DWI) and T1-weighted (T1w) data from 50 independent studies spanning 0 to 100 years of age (Extended Data Table 1), encompassing 75,036 DWI scans from cognitively normal and clinical participants. All data were converted from DICOM to NIfTI using dcm2niix and organized in BIDS format in accordance with previous work^44^. Notably, the 50 included datasets were harmonized through standardized preprocessing, postprocessing, and feature-wise statistical harmonization using GAMLSS.

### MRI processing pipeline

DWI data were preprocessed using the PreQual pipeline^45^, which corrects for susceptibility-induced, motion-related and eddy current distortions, and performs slice-wise signal imputation. Specifically, image denoising was performed using the MRtrix3 toolkit^46^ (v3.0.4) implementation of Marchenko–Pastur principal component analysis. Following this, TOPUP from the FSL (v6.0.4) software library^47^ is used for susceptibility-distortion correction. For DWI without reverse phase encoding scans acquired, TOPUP is run using a synthetic b0 image created from a T1w scan from the same imaging session via the Synb0-DISCO^48^ algorithm (v3.1). FSL’s EDDY is then used for motion and eddy current-distortion correction, also performing slice-wise signal imputation with the flag ‘--repol‘. Following preprocessing, DTI models were fit to all volumes with b-values ≤ 1,500 s mm^−2^ (refs. ^40,49^) using dwi2tensor from MRtrix3. DTI-derived scalar maps—including FA, MD, AD and RD—were computed using tensor2metric from MRtrix3^46^.

To enable consistent tract segmentation, all diffusion data were resampled to 1 mm isotropic resolution using MRtrix3^50^. Tractography was performed using TractSeg^51^ (v2.8), which automatically segments 72 anatomically defined cerebral WM pathways (see Extended Data Table 2 for a list of tracts and abbreviations). For each tract, we computed streamline density-weighted averages of DTI metrics (FA, MD, AD and RD) as well as macrostructural features—volume, length and surface area—using the Scilpy toolkit^52^ (v1.5.0) scripts scil_compute_bundle_mean_std.py and scil_evaluate_bundles_individual_measures.py, respectively. Both raw and ratio-normalized macrostructural features are provided, where volume, surface area and average length of tracts are normalized to total brain volume (excluding ventricles), estimated total intracranial volume and cerebral WM volume.

T1w images were included only when acquired in the same session as DWI data. Brain segmentation was performed using the recon-all command from FreeSurfer (v7.2.0)^53^, yielding estimates of cerebral WM volume, brain volume excluding ventricles and total intracranial volume. For participants aged ≤2 years, we employed infant_recon_all from the infant FreeSurfer pipeline^54^ (v0.0) to account for age-specific brain morphology. Cerebral WM masks were defined using MRtrix3^46^ 5TT labels, excluding the cerebellum and brainstem. T1w images and corresponding WM segmentations were rigidly registered to DWI space using FSL’s^47^ epi_reg. Whole-brain WM DTI metrics were then computed by averaging values within the WM mask.

The PreQual preprocessing pipeline can be downloaded from https://zenodo.org/records/14058394. We also make the entire postprocessing pipeline available at https://zenodo.org/records/17144460.

### Data selection

Following the approach of Bethlehem et al.^6^, we restricted our analysis to cross-sectional data. Participant scans were excluded if key demographic information—age, sex or cognitive status—was missing or not reported for that participant. For each dataset, sex was used as reported and encoded as a binary variable, whereas age was a continuous value in years. Regarding infants in the BABIES-ABC, HBCD and dHCP datasets, we used corrected age based on the due date to better account for prematurity and provide a more accurate developmental context.

Quality control (QC) procedures followed our previously established framework for large-scale dMRI analysis^55^. QC metrics were visually reviewed across PreQual preprocessing outputs, FreeSurfer segmentations, and T1w–DWI registration results. Scans were excluded if any preprocessing step failed or was deemed unusable. TractSeg outputs were excluded if more than 12 of the 72 tracts failed to reconstruct. Outlier removal was performed separately for each tract-level feature within each dataset, with observations excluded if exceeding ±4 standard deviations from the mean. We note it is possible for FreeSurfer to have failed with successful tractography, and vice-versa, in which cases we retained only data from the contrast that passed quality assessment. The number of sessions and participants retained after QC is detailed in Extended Data Table 1.

Normative trajectories were modelled using only participants labelled as ‘typically developing’ or ‘control’ within their respective studies to reflect patterns of healthy WM development and ageing. Participants who were initially labelled as typically ageing but later transitioned to a clinical diagnosis were also excluded when fitting normative trajectories. At the tract level, features were retained only if the tract was reconstructed with the full target of 2,000 streamlines—the default setting in TractSeg.

### Normative modelling of features

We utilized GAMLSS^15^, a flexible regression framework endorsed by the World Health Organization for constructing normative growth curves^12,16^. GAMLSS extends generalized linear and additive models by allowing the simultaneous estimation of multiple distribution parameters—not only the mean, but also variance, skewness and kurtosis—through functions that vary with age and other covariates. This flexibility enables precise modelling of lifespan trajectories for WM features.

Formally, GAMLSS defines each parameter of the assumed response distribution through additive predictors:

1 $${g}_{k}({\theta }_{k})={X}_{k}{\beta }_{k}+\mathop{\sum }\limits_{j=1}^{{J}_{k}}{Z}_{{kj}}{\gamma }_{{kj}},$$

where ${g}_{k}(\cdot )$ is the link function for the $k$th parameter, ${X}_{k}$ and ${\beta }_{k}$ are the design matrix and fixed effects. The summation incorporates ${J}_{k}$ smooth functions, where ${Z}_{{kj}}$ are the design matrices for the basis expansions and ${\gamma }_{{kj}}$ are the corresponding smoothing coefficients. This formulation allows flexible, non-linear modelling of the entire distribution as a function of covariates such as age, sex and dataset.

Previous work has shown dMRI-derived features to have skewed distributions. Following Bethlehem et al.^6^, we used the generalized gamma (GG) distribution with fractional polynomial ($\mathrm{fp}$) fitting to estimate lifespan trajectories. The GG distribution offers substantial flexibility, accommodating a broad range of distributional shapes, and is therefore suitable for modelling both microstructural and macrostructural imaging features. The model parameters, location (*μ*), scale (*σ*) and shape (*ν*), were defined as:

2 $${g}_{1}(\mu )={x}_{\mathrm{sex}}{\beta }_{1,\mathrm{sex}}+{{\rm{fp}}}_{1}({x}_{\mathrm{age}})+{\gamma }_{1,D}$$

3 $${g}_{2}(\sigma )={x}_{\mathrm{sex}}{\beta }_{2,\mathrm{sex}}+{{\rm{fp}}}_{2}({x}_{\mathrm{age}})+{\gamma }_{2,D}$$

4 $${g}_{3}(\nu )=\alpha ,$$

where ${g}_{1}(\cdot )$ and ${g}_{2}(\cdot )$ denote log link functions, whereas ${g}_{3}(\cdot )$ is an identity link. Age was modelled as a continuous predictor using fractional polynomial transformations ${\mathrm{fp}}_{k}(\cdot )$, enabling non-linear representation of age-related effects. Sex was included as a binary fixed effect (${x}_{\mathrm{sex}}$) and dataset-specific variability was modelled via random intercepts (${\gamma }_{1,D}$ and ${\gamma }_{2,D}$). The GG distribution was parameterized as described in Rigby et al.^56^ to enable compatibility with GAMLSS framework. Model selection was performed by the Bayesian information criterion^57^ across all combinations of 1–3 fractional polynomial terms for the $\mu$ and $\sigma$ parameters. The shape parameter $\nu$ was treated as a global offset without age- or dataset-specific effects, consistent with Bethlehem et al.^6^.

We modelled normative trajectories for 509 features, including 288 tract-level (72 tracts × 4 features) microstructural measures (mean FA, MD, AD and RD) and 216 macrostructural (72 tracts × 3 features) measures (volume, average length and surface area). An additional five global WM features—mean FA, MD, AD, RD and WM volume—were also modelled. Macrostructural features were further normalized using total intracranial volume, brain volume excluding ventricles, and global WM volume to account for anatomical scaling.

To derive scaling factors for normalization, we assumed a spherical brain geometry and estimated radius and surface area using:

5 $${r}_{\mathrm{Brain}}={\left(\frac{3{V}_{\mathrm{Brain}}}{4{\rm{\pi }}}\right)}^{\frac{1}{3}}$$

and

6 $${\mathrm{SA}}_{\mathrm{Brain}}=4{\rm{\pi }}{r}_{\mathrm{Brain}}$$

respectively, where ${V}_{\mathrm{Brain}}$ denotes the normalizing volume, with separate scaling factors derived for estimated total intracranial volume, brain volume excluding ventricles, and global WM volume. *V*_Brain_, SA_Brain_ and *r*_Brain_ (Equations 5 and 6) were used to normalize tract-level volume, surface area and average length metrics, respectively.

When considering both normalized and unnormalized features, we had 1,157 features in total for which we created lifespan curves: 509 unnormalized (288 microstructure, 216 macrostructure and 5 global) and 648 normalized (216 macrostructure normalized in 3 separate ways) features.

### WM lifespan milestones

For assessing myelination, we studied the median RD trajectories of tract-specific brain charts. Following the work from Yeatman et al.^17^, we performed piecewise segmentations of the trajectories to assess whether the myelination time points align with proposed periods of myelin development and maturation derived in refs. ^18,25,26^. Specifically, we performed a piecewise segmentation of the median trajectories to identify three break points that minimize the sum of squared errors across the resulting linear segments (where the piecewise model was fit between ages 1 and 90 years). The segments correspond to myelination periods of rapid development in infancy, slower myelination in adolescence, the stable plateau of young adulthood, and the decline of ageing. For each segment, we calculated both the average slope and the per cent change per year (Supplementary Table SDT.T5).

### Centile scores across cognitive groups

We used the fitted normative trajectories and dataset-specific random effects to compute individualized centile scores for non-control participants across all tract-level features. In total, 6,777 participants from 16 clinical groups passed QC and were included in the analysis (Extended Data Table 3).

As the analysis was restricted to cross-sectional data, we selected a single scan per participant. For individuals with longitudinal data, we retained the most recent scan corresponding to their most severe clinical diagnosis. For example, participants progressing from cognitively unimpaired to MCI and then to Alzheimer’s disease were classified based on their most recent scan with a clinical Alzheimer’s disease or dementia diagnosis.

For each clinical group, we compared the distribution of centile scores across tract metrics to the 50th percentile expected in the normative population. In addition, we quantified overall deviation from the normative distribution using the nCMD for each participant $j$ (${\mathrm{nCMD}}_{j}$):

7 $${\mathrm{nCMD}}_{j}=\frac{\sqrt{{(x-\mu )}^{T}{S}^{-1}(x-\mu )}}{{N}_{{j}_{\mathrm{tot}}}}$$

where ${N}_{{j}_{\mathrm{tot}}}$ is the number of reconstructed tract features for participant $j$, $x$ is a vector of centile scores (ranging from $0$ to $1$), $\mu =0.5$ denotes the median centile, and ${S}^{-1}$ is the inverse of the covariance matrix for the features.

We computed nCMD separately for tract-level microstructural features, macrostructural features, and their combination. Group-level comparisons were then made by evaluating the median nCMD in each non-control group relative to the distribution observed in the cognitively normal population, where statistical significance was tested using a one-sample Wilcoxon test (two-sided) after Bonferroni correction for multiple comparisons.

### Alignment for OOS datasets

A central utility of normative brain charts is their use as reference models for external, OOS datasets. To enable this, new datasets must be aligned to the fitted trajectories by estimating study-specific offsets for the distributional parameters. Within the GAMLSS framework, each dataset $D$ is modelled with random effect terms ${\gamma }_{1,D}$ and ${\gamma }_{2,D}$, which account for dataset-specific variabilities in the location ($\mu$) and scale ($\sigma$) parameters, respectively. For an unseen dataset $S$, alignment involves estimating these two unknown offsets. We demonstrate this process using the ADNI dataset, where we fit additional models without ADNI for the purpose of demonstrating OOS alignment.

For a given brain chart modelling a tract-level metric, the GAMLSS-based lifespan distribution is defined as:

8 $${g}_{1}(\mu )={\mathrm{fp}}_{1}({x}_{\mathrm{age}})+{x}_{\mathrm{sex}}{\beta }_{1,\mathrm{sex}}+{\gamma }_{1,S}$$

9 $${g}_{2}(\sigma )={\mathrm{fp}}_{2}({x}_{\mathrm{age}})+{x}_{\mathrm{sex}}{\beta }_{2,\mathrm{sex}}+{\gamma }_{2,S}$$

10 $${g}_{3}(\nu )=\alpha ,$$

where ${\gamma }_{1,S}$ and ${\gamma }_{2,S}$ are dataset-specific random effects for the ADNI dataset, and all other model parameters are fixed from the trained GAMLSS model fitted without ADNI data.

As all other effects are known from the reference model, alignment requires estimating only the unknown random effects ${\gamma }_{1,S}$ and ${\gamma }_{2,S}$. We initialized both to zero and restricted the estimation procedure to typically ageing individuals in the ADNI cohort. To minimize diagnostic confounding, we selected only participants with no history or future diagnosis of MCI or Alzheimer’s disease and used their earliest available scan. This cognitively normal subset is denoted ${S}_{\mathrm{CN}}$.

For each cognitively normal participant $j$ in ${S}_{\mathrm{CN}}$, we computed the likelihood of their observed metric ${M}_{j}$ under the GG distribution defined by equations (8)–(10). Each of the resulting probability densities ${P}_{j}={GG}({M}_{j}|\mu ,\sigma ,\nu )$ were used to calculate the overall negative log-likelihood across all participants in ${S}_{\mathrm{CN}}$:

11 $${\rm{Negative}}\,\log \mathrm{-}{\rm{likelihood}}=-\mathop{\sum }\limits_{j=1}^{|{S}_{{\rm{CN}}}|}\log ({P}_{j}),$$

We iteratively re-estimated ${\gamma }_{1,S}$ and ${\gamma }_{2,S}$ by minimizing the negative log-likelihood until convergence. The final estimates, ${\hat{\gamma }}_{1,S}$ and ${\hat{\gamma }}_{2,S}$, were then applied to all participants in the ADNI dataset to compute adjusted centile scores aligned to the normative reference space.

### Reporting summary

Further information on research design is available in the Nature Portfolio Reporting Summary linked to this article.

## Online content

Any methods, additional references, Nature Portfolio reporting summaries, source data, extended data, supplementary information, acknowledgements, peer review information; details of author contributions and competing interests; and statements of data and code availability are available at 10.1038/s41586-026-10454-2.

## Supplementary information

Supplementary Information Supplementary information, including figures, tables and references.

Reporting Summary

Supplementary Acknowledgements Datasets.

Supplementary Table 1 Dataset information summary table for typically developing and ageing participants. Sample sizes of typically ageing and developing participants for each dataset used to construct the brain charts, with the minimum and maximum ages in years for each dataset as well as the number of male and female participants.

Supplementary Table 2 Brain chart participant summary information. Sample sizes of typically ageing and developing participants used to construct the brain charts, with the minimum and maximum ages in years for each chart as well as the number of male and female participants.

Supplementary Table 3 Participant count of each tract and feature for diagnostic groups. Sample sizes used for the statistical tests in Fig. 5, Extended Data Figs. 6 and 7 and Supplementary Information, section C analyses.

Supplementary Table 4 Tract-specific median centiles of brain charts. SDT.T4.1-504 contain the discretized, sex-agnostic brain charts used to derive the myelination plots and assessments in Fig. 4 and Supplementary Fig. S4.F9. Age is expressed in years. Tract measurements are reported in metric-specific units: diffusivities in mm^2^ s^−1^, volume in mm^3^, surface area in mm^2^, average length in mm, and FA is dimensionless (unitless).

Supplementary Table 5 WM lifespan milestones and rates. Developmental and ageing time points and rates used to generate the plots and results in Fig. 4 and Supplementary Fig. S4.F9. T1, T2, and T3 denote the three identified break points separating developmental and ageing phases. ‘Infancy’ refers to the interval from start to T1, ‘Adolescence’ from T1 to T2, ‘Young Adult’ from T2 to T3, and ‘Aging’ from T3 to the end of the age range. Age is expressed in years. Tract measurements are reported in metric-specific units: diffusivities in mm^2^ s^−1^, volume in mm^3^, surface area in mm^2^, average length in mm, and FA is dimensionless (unitless). Rates are expressed in tract-specific units per year.

Supplementary Table 6 Case–control comparison p-values. SDT.T6.1 contains the Bonferroni-adjusted *P* values for the statistical tests (one-sample two-sided Wilcoxon signed-rank test) in Fig. 5a. SDT.T6.2–17 contain the Bonferroni-adjusted *P* values for the statistical tests in Fig. 5b,c, Extended Data Figs. 6 and 7 and Supplementary Fig. S3.F1 analyses. Note that the adjustments were based on a false positive rate of $\alpha =0.05$.

Supplementary Table 7 Case–control comparison *P* values with Benjamini–Hochberg false discovery rate correction. SDT.T7.1–16 contain the Benjamini–Hochberg false discovery rate-adjusted *P* values for the statistical tests (one-sample two-sided Wilcoxon signed-rank test) in Supplementary Figs. S3.F2–S3.F5 analyses. Note that the adjustments were based on a false positive rate of $\alpha =0.05$.

Supplementary Table 8 Case–control comparison distances to 50th centile. SDT.T8.1–16 contain the median distances to the 50th normative centile for each tract and feature across diagnostic groups used in Fig. 5b,c, Extended Data Figs. 6 and 7 and Supplementary Information, section 3, ‘Additional diagnostic group centile deviations’. Negative values indicate the median was higher than the 50th centile, whereas positive values indicate that the median was lower. Values range from 0 to 1, with 0.5 as the 50th centile.

Supplementary Table 9 Centiles trajectories of brain charts. SDT.T9.1–5 contain the brain charts for the global metrics; SDT.T9.6–509 contain the brain charts for the tract-specific metrics (unnormalized); and SDT.T9.510–1157 contain the brain charts for the tract-specific macrostructural metrics (normalized). Included in each table are the male and female centile trajectories for the 2.5th, 25th, 50th, 75th and 97.5th percentiles; the interquartile range normalized by the 50th centile; and the derivatives of the 50th centiles with respect to both age and log(age). Charts are sampled in logarithmically spaced intervals. Age is reported in years, while centile trajectory values are reported in metric-specific units: diffusivities in mm^2^ s^−1^, volume in mm^3^, surface area in mm^2^, average length in mm, and FA is dimensionless (unitless).

Peer Review File

## Figures and Tables

**Extended Data Table 1:**
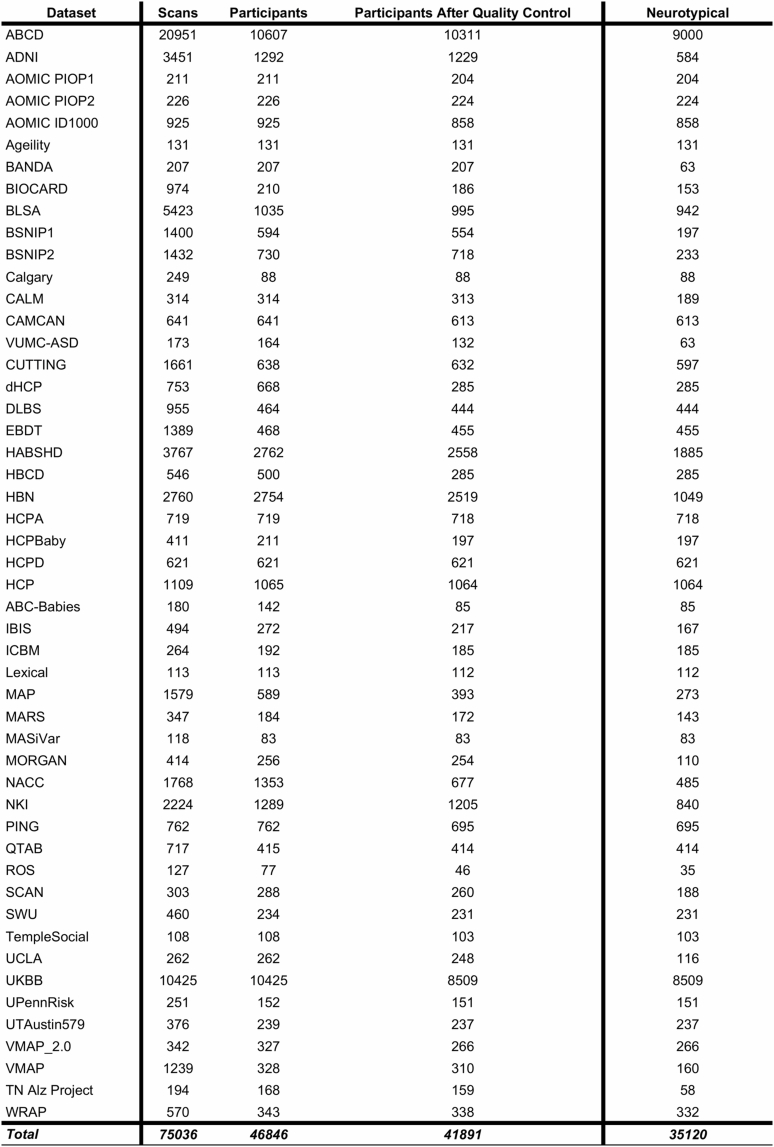
Participants from each dataset used to create the WM brain charts

**Extended Data Table 2:**
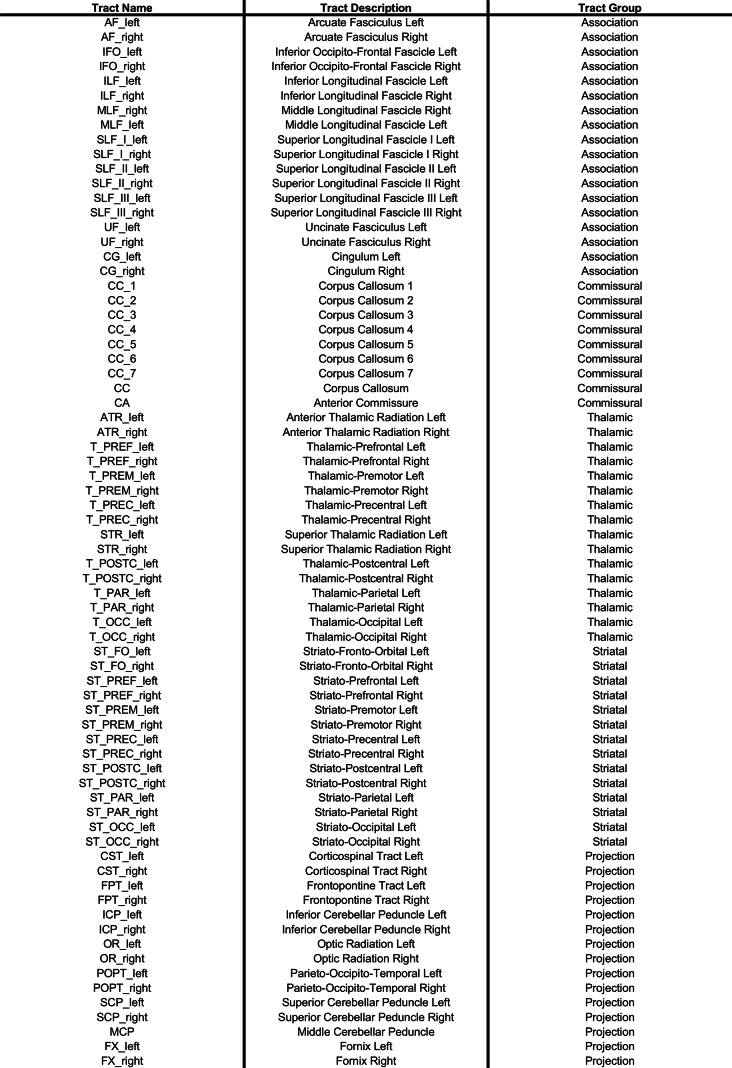
The 72 TractSeg^51^ tract definitions with the corresponding tract groups

Note that for fitting out-of-sample datasets, the values in the “Tract Name” column should be used for the CSV when performing out-of-sample alignment.

**Extended Data Table 3:**
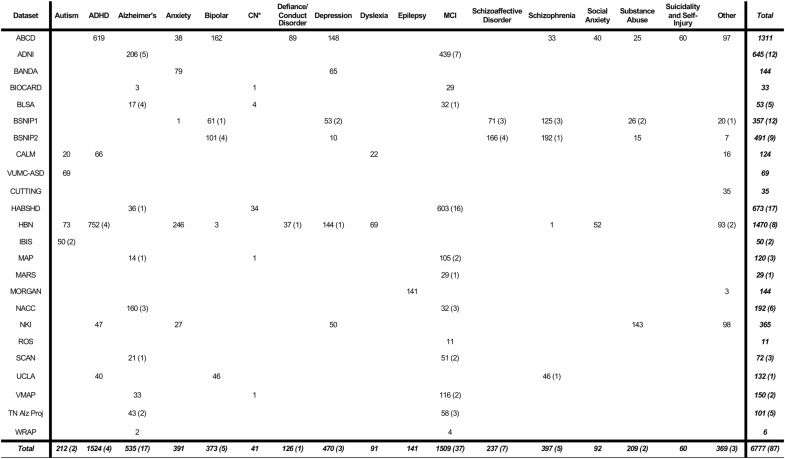
Participants classified as cognitively abnormal for each dataset and diagnosis

Values in parentheses denote the number dropped from the +/−4 standard deviation outlier removal detailed in the Methods. CN* denotes individuals diagnosed as cognitively unimpaired at scan time, but were later diagnosed as having some degree of cognitive impairment (including dementia).

## Data Availability

All derived data and associated demographic information from publicly available datasets, as well as from datasets for which the Data Use Agreement (DUA) permits sharing, have been made accessible at https://zenodo.org/records/18891847 (ref. ^58^). For datasets where the DUA does not permit redistribution of derivative data, these materials have been returned to or shared directly with the respective data custodians. Data from the Alzheimer’s Disease Neuroimaging Initiative (ADNI) are available upon request from https://adni.loni.usc.edu/. Data from the AOMIC-PIOP1 dataset are freely available for download on OpenNeuro: https://openneuro.org/datasets/ds002785/versions/2.0.0. Data from the AOMIC-PIOP2 dataset are freely available for download on OpenNeuro: https://openneuro.org/datasets/ds002790/versions/2.0.0. Data from the AOMIC-ID1000 dataset are freely available for download on OpenNeuro: https://openneuro.org/datasets/ds003097/versions/1.2.1. Data from the Boston Adolescent Neuroimaging of Depression and Anxiety (BANDA) dataset are available upon request from https://www.humanconnectome.org/study/connectomes-related-anxiety-depression. Data from BIOCARD are available upon request after filling out a data use application: https://www.gaaindata.org/partner/BIOCARD. Data from the Baltimore Longitudinal Study of Aging (BLSA) are available upon request from https://www.blsa.nih.gov/. Data from the Calgary Preschool MRI Dataset are freely available for download at: 10.17605/OSF.IO/AXZ5R. Data from the Centre for Attention Learning and Memory (CALM) dataset are available upon request from https://calm.mrc-cbu.cam.ac.uk/researchers/. Data from the Cambridge Center for Ageing Neuroscience (CAMCAN) dataset are available upon request from https://camcan-archive.mrc-cbu.cam.ac.uk/dataaccess/. Data from the Dallas Lifespan Brain Study (DLBS) dataset are freely available for download on OpenNeuro at https://openneuro.org/datasets/ds004856/versions/1.2.0. Data from the Health and Aging Brain Study—Health Disparities (HABS-HD) dataset are available upon request from https://apps.unthsc.edu/itr/reports. Imaging data and basic demographic information for the Healthy Brain Network (HBN) dataset are freely available to download from https://fcon_1000.projects.nitrc.org/indi/cmi_healthy_brain_network/. Phenotypic data are available upon request by filling out a data use agreement (https://fcon_1000.projects.nitrc.org/indi/cmi_healthy_brain_network/Phenotypic.html). Data from the Human Connectome Project—Aging (HCPA) dataset are available upon request from https://www.humanconnectome.org/study/hcp-lifespan-aging. Data from the Lifespan Baby Connectome Project (HCPBaby) dataset are available upon request from https://www.humanconnectome.org/study/lifespan-baby-connectome-project. Data from the Human Connectome Project—Development (HCPD) dataset are available upon request from https://www.humanconnectome.org/study/hcp-lifespan-development. Data from the Human Connectome Project—Young Adult (HCP) dataset are freely available for download from https://www.humanconnectome.org/study/hcp-young-adult. Data from the Infant Brain Imaging Study (IBIS) are available for download from the National Institutes of Mental Health data archive upon request from https://nda.nih.gov/edit_collection.html?id=19. Data from the International Consortium for Brain Mapping (ICBM) dataset are available upon request from www.loni.usc.edu/ICBM. Data from the Longitudinal Brain Correlates of Multisensory Lexical Processing in Children study (shortened to Lexical in this manuscript) are freely available for download on OpenNeuro (https://openneuro.org/datasets/ds001894/versions/1.4.2). Data from the Memory and Aging Project (MAP), Religious Orders Study (ROS) and the Minority Aging Research Study (MARS) datasets are available upon request from https://www.radc.rush.edu/. More information about participant demographics and study information can be found at https://www.rushu.rush.edu/research-rush-university/departmental-research/rush-alzheimers-disease-center/rush-alzheimers-disease-center-research/epidemiologic-research. Data from the Multisite, Multiscanner, and Multisubject Acquisitions for Studying Variability in Diffusion Weighted Magnetic Resonance Imaging (MASiVar) dataset are freely available for download on OpenNeuro at https://openneuro.org/datasets/ds003416/versions/2.0.2. Data from the National Alzheimer’s Coordinating Center (NACC) and the Standardized Centralized Alzheimer’s and Related Dementias Neuroimaging (SCAN) are available upon request from https://naccdata.org/requesting-data/data-request-process. Imaging data and basic demographic information for the Nathan Kline Institute—Rockland Sample (NKI) dataset are freely available to download from https://rocklandsample.org/accessing-the-neuroimaging-data-releases. Phenotypic data are available upon request by filling out a data use agreement (https://rocklandsample.org/phenotypic-data). Data from the Pediatric Imaging, Neurocognition, and Genetics dataset (PING) are available for download from the National Institutes of Mental Health data archive upon request from https://nda.nih.gov/edit_collection.html?id=2607. Data from the Queensland Twin Adolescent Brain (QTAB) dataset are freely available for download on OpenNeuro at https://openneuro.org/datasets/ds004146/versions/1.0.4. Data from the UCLA Consortium for Neuropsychiatric Phenomics LA5c Study (UCLA) dataset are freely available for download on OpenNeuro at https://openneuro.org/datasets/ds000030/versions/1.0.0. Data coming from the Southwestern University (SWU) dataset, part of the Consortium for Reliability and Reproducibility (CoRR), were downloaded via NITRC-IR from the 1000 Functional Connectomes Project. In order to access the CoRR datasets through NITRC, users must be logged into NITRC at the time of download and registered with the 1000 Functional Connectomes Project/INDI website. More information about this subset can be found at https://fcon_1000.projects.nitrc.org/indi/CoRR/html/swu_4.html. Data from the Southwest University (SWU) Longitudinal Imaging Multimodal dataset are freely available for download at https://fcon_1000.projects.nitrc.org/indi/retro/southwestuni_qiu_index.html. Data from the Social Reward and Nonsocial Reward Processing Across the Adult Lifespan: An Interim Multi-echo fMRI and Diffusion Dataset (referred to as TempleSocial in this manuscript) are freely available for download on OpenNeuro at https://openneuro.org/datasets/ds005123/versions/1.1.3. Data from UK Biobank (UKBB) are available upon request from https://www.ukbiobank.ac.uk/. Data from the UPennRisk dataset are freely available for download on OpenNeuro at https://openneuro.org/datasets/ds002843/versions/1.0.1. Data from the dataset ‘A longitudinal neuroimaging dataset on language processing in children ages 5, 7, and 9 years old’ (referred to as UTAustin579 in this manuscript) are freely available for download on OpenNeuro at https://openneuro.org/datasets/ds003604/versions/1.0.7. Data from the Vanderbilt Memory and Aging Project (VMAP_JEFFERSON, VMAP_2.0, TN Aging Project) are available for download upon request from https://vmacdata.org/vmap/data-requests. Data from the Wisconsin Registry for Alzheimer’s Prevention (WRAP) are available upon request from https://wrap.wisc.edu/data-requests-2/. Data from the HEALthy Brain and Child Development (HBCD) Study are available for download upon request from https://hbcdstudy.org/data-sharing/. Data from the Adolescent Brain Cognitive Development (ABCD) Study are available for download upon request from (https://abcdstudy.org/scientists/data-sharing/). Data used in this manuscript from the Ageility Project (Phase 1 only) are available for download from https://www.nitrc.org/projects/age-ility. Data from the Early Brain Development in Twins (EBDT) dataset are available for download from the National Institutes of Mental Health data archive upon request from https://nda.nih.gov/edit_collection.html?id=2384. Data from the Bipolar and Schizophrenia Consortium for Parsing Intermediate Phenotypes dataset (BSNIP1) and its renewal (BSNIP2) are available for download from the National Institutes of Mental Health data archive upon request from https://nda.nih.gov/edit_collection.html?id=2274 and https://nda.nih.gov/edit_collection.html?id=2165. Data from the Developing Human Connectome Project (dHCP) are available for download from the National Institutes of Mental Health data archive upon request from https://nda.nih.gov/edit_collection.html?id=3955. Vanderbilt University data (MORGAN, BABIES-ABC, CUTTING, VUMC-ASD) are subject to third party restrictions. Please contact corresponding authors for data requests.

## Code availability

Code for OOS data alignment, obtaining centile curves, and fitting the GAMLSS models is available in a containerized Docker image that is downloadable from https://zenodo.org/records/15367425 (ref. ^42^). Instructions for running the Docker image can also be found at this URL and in the Supplementary Information. The postprocessing pipeline to obtain the microstructural and macrostructural measurements from dMRI data is also available as a Docker image downloadable from https://hub.docker.com/r/kimm58/wm_lifespan_processing. Instructions for running the Docker image are available at https://zenodo.org/records/17144461 (ref. ^59^).
