# Publisher Correction: White matter micro- and macrostructure brain charts for the human lifespan

**DOI:** 10.1038/s41586-026-10693-3

**Published:** 2026-06-02

**Authors:** Michael E. Kim, Chenyu Gao, Karthik Ramadass, Nancy R. Newlin, Praitayini Kanakaraj, Sam Bogdanov, Gaurav Rudravaram, Derek Archer, Timothy J. Hohman, Angela L. Jefferson, Victoria L. Morgan, Alexandra Roche, Dario J. Englot, Susan M. Resnick, Lori L. Beason-Held, Laurie E. Cutting, Laura A. Barquero, Micah A. D’archangel, Tin Q. Nguyen, Kathryn L. Humphreys, Yanbin Niu, Sophia Vinci-Booher, Carissa J. Cascio, The HABS-HD Study Team, Sid O’Bryant, L. Taylor Davis, Zhiyuan Li, Simon N. Vandekar, Panpan Zhang, John C. Gore, Bennett A. Landman, Kurt G. Schilling

**Affiliations:** 1 Department of Computer Science, Vanderbilt University, Nashville, TN USA; 2 Department of Electrical and Computer Engineering, Vanderbilt University, Nashville, TN USA; 3 Medical Scientist Training Program, Vanderbilt University, Nashville, TN USA; 4 Vanderbilt Memory and Alzheimer’s Center, Vanderbilt University Medical Center, Nashville, TN USA; 5 Vanderbilt Genetics Institute, Vanderbilt University Medical Center, Nashville, TN USA; 6 Vanderbilt Brain Institute, Vanderbilt University, Nashville, TN USA; 7 Department of Medicine, Vanderbilt University Medical Center, Nashville, TN USA; 8 Department of Neurology, Vanderbilt University Medical Center, Nashville, TN USA; 9 Department of Psychology and Human Development, Vanderbilt University, Nashville, TN USA; 10 Department of Psychiatry and Behavioral Sciences, Vanderbilt University Medical Center, Nashville, TN USA; 11 Department of Radiology and Radiological Sciences, Vanderbilt University Medical Center, Nashville, TN USA; 12 Vanderbilt University Institute of Imaging Science, Nashville, TN USA; 13 Department of Biomedical Engineering, Vanderbilt University, Nashville, TN USA; 14 Department of Neurological Surgery, Vanderbilt University Medical Center, Nashville, TN USA; 15 Laboratory of Behavioral Neuroscience, National Institute on Aging, National Institutes of Health, Baltimore, MD USA; 16 Department of Special Education, Peabody College of Education and Human Development, Nashville, TN USA; 17 Life Span Institute and Department of Psychology, University of Kansas, Lawrence, KS USA; 18 Department of Computer Science, Park University, Parkville, MO USA; 19 Department of Biostatistics, Vanderbilt University Medical Center, Nashville, TN USA; 20 Department of Family Medicine, University of North Texas Health Science Center, Fort Worth, TX USA; 21 Laboratory of Neuro Imaging, Department of Neurology, University of California School of Medicine, Los Angeles, CA USA; 22 Department of Neurology, Johns Hopkins University, Baltimore, MD USA

**Keywords:** Brain imaging, Data processing

Correction to: *Nature* 10.1038/s41586-026-10454-2 Published online 13 May 2026

In the version of this article initially published, the label for Fig. 3a read “Tract mean AD” instead of “White matter tracts”. The error has been corrected in the HTML and PDF versions of the article.

