# Supplementary material for: White matter micro- and macrostructure brain charts for the human lifespan
Source: Nature. 2026 May 13;655(8124):979–89. doi: 10.1038/s41586-026-10454-2 (PMC13391363; doi:10.1038/s41586-026-10454-2)
Supplement: Supplementary file 1 — Supplementary information, including figures, tables and references. [file 41586_2026_10454_MOESM1_ESM.pdf]

nature portfolio

https://doi.org/10.1038/s41586-026-10454-2

Supplementary information

White matter micro- and macrostructure brain charts for the human lifespan

In the format provided by the authors and unedited

Supplemental Information

White matter micro and macrostructure brain charts for the human lifespan

Michael E. Kim[a] *, Chenyu Gao[b], Karthik Ramadass[a,b], Nancy R. Newlin[a], Praitayini Kanakaraj[a], Sam Bogdanov[c], Gaurav Rudravaram[b], Derek Archer[d,e,f], Timothy J. Hohman[d,e], Angela L. Jefferson[d,g,h,i,j], Victoria L. Morgan[k,l,m], Alexandra Roche[l], Dario J. Englot[a,b,k,m,n] Susan M. Resnick[o], Lori L. Beason-Held[o], Laurie Cutting[f,p], Laura A. Barquero[p], Micah A. D'archangel[p], Tin Q. Nguyen[f,l,p], Kathryn L. Humphreys[i], Yanbin Niu[i], Sophia Vinci-Booher[i], Carissa J. Cascio[q], The HABS-HD Study Team[A], Alzheimer's Disease Neuroimaging Initiative[B], The BIOCARD Study Team[C], L. Taylor Davis[k], Zhiyuan Li[r], Simon N. Vandekar[s], Panpan Zhang[d,s], John C. Gore[k,l,m], Bennett A. Landman[a,b,f,h,i,j,k,l,m,s] *, Kurt G. Schilling[b,k,l] *

[a] Vanderbilt University, Department of Computer Science, Nashville, TN, USA
[b] Vanderbilt University, Department of Electrical and Computer Engineering, Nashville, TN, USA
[c] Vanderbilt University, Medical Scientist Training Program, Nashville, TN, USA
[d] Vanderbilt University Medical Center, Vanderbilt Memory and Alzheimer's Center, Nashville, TN, USA
[e] Vanderbilt University Medical Center, Vanderbilt Genetics Institute, Nashville, TN, USA
[f] Vanderbilt University, Vanderbilt Brain Institute, Nashville, TN, USA
[g] Vanderbilt University Medical Center, Department of Medicine, Nashville, TN, USA
[h] Vanderbilt University Medical Center, Department of Neurology, Nashville, TN, USA
[i] Vanderbilt University, Department of Psychology and Human Development, Nashville, TN, USA
[j] Vanderbilt University Medical Center, Department of Psychiatry and Behavioral Sciences, Nashville, TN, USA
[k] Vanderbilt University Medical Center, Department of Radiology and Radiological Sciences, Nashville, TN, USA
[l] Vanderbilt University Institute of Imaging Science, Nashville, TN, USA
[m] Vanderbilt University, Department of Biomedical Engineering, Nashville, TN, USA
[n] Vanderbilt University Medical Center, Department of Neurological Surgery, Nashville, TN, USA
[o] Laboratory of Behavioral Neuroscience, National Institute on Aging, National Institutes of Health, Baltimore, MD, USA
[p] Peabody College of Education and Human Development, Department of Special Education, Nashville, Tennessee, USA
[q] University of Kansas, Life Span Institute and Department of Psychology, Lawrence, KS, USA
[r] Park University, Department of Computer Science, Parkville, MO, USA
[s] Vanderbilt University Medical Center, Department of Biostatistics, Nashville, TN, USA

[A] HABS-HD MPIs: A list of authors and their affiliations appears at the end of the paper

[B] Alzheimer's Disease Neuroimaging Initiative: A list of authors and their affiliations can be accessed via a URL in the acknowledgements section

[C] The BIOCARD Study Team: A list of authors and their affiliations accessed via a URL in the acknowledgements section

[*] *Corresponding Author*

Table of Contents

1. *Additional Brain Charts* ____ 3
2. *Data Information* ____ 14
3. *Additional Diagnostic Group Centile Deviations* ____ 17
   i. Centile Score Deviations of Other Diagnostic Groups ____ 17
   ii. Effect Sizes of Centile Score Deviations ____ 22
4. *Model Fitting* ____ 26
   i. Number of fractional polynomial terms for μ and σ ____ 26
   ii. Empirical Model Stability Analysis ____ 27
   iii. Voxel Size Effect on FA ____ 28
   iv. Batch Correction and Site Harmonization ____ 28
   v. Sex-related Effects on White Matter Brain Charts ____ 30
   vi. Maxima and Minima of White Matter Tract Trajectories ____ 32
   vii. Use of Milestones to Examine Development and Aging ____ 33
5. *Comparison to Existing Reference Charts* ____ 34
   i. White Matter Lifespan Modeling in the Literature ____ 34
   ii. Comparison to Bethlehem et al. ____ 35
   iii. Comparison to Zhu et al. ____ 37
6. *Data Quality* ____ 40
7. *Anomaly Detection* ____ 43
8. *Out-of-Sample Alignment* ____ 44
   i. Stability of Out-of-Sample Alignment ____ 44
   ii. Alignment with Other Preprocessing Pipelines ____ 45
   iii. How to Perform Out-of-Sample Data Alignment ____ 46
   iv. How to obtain centile trajectories of features ____ 47
9. *Considerations for Cross-sectional vs. Longitudinal Brain Charts* ____ 48
10. *References* ____ 49

1. Additional Brain Charts

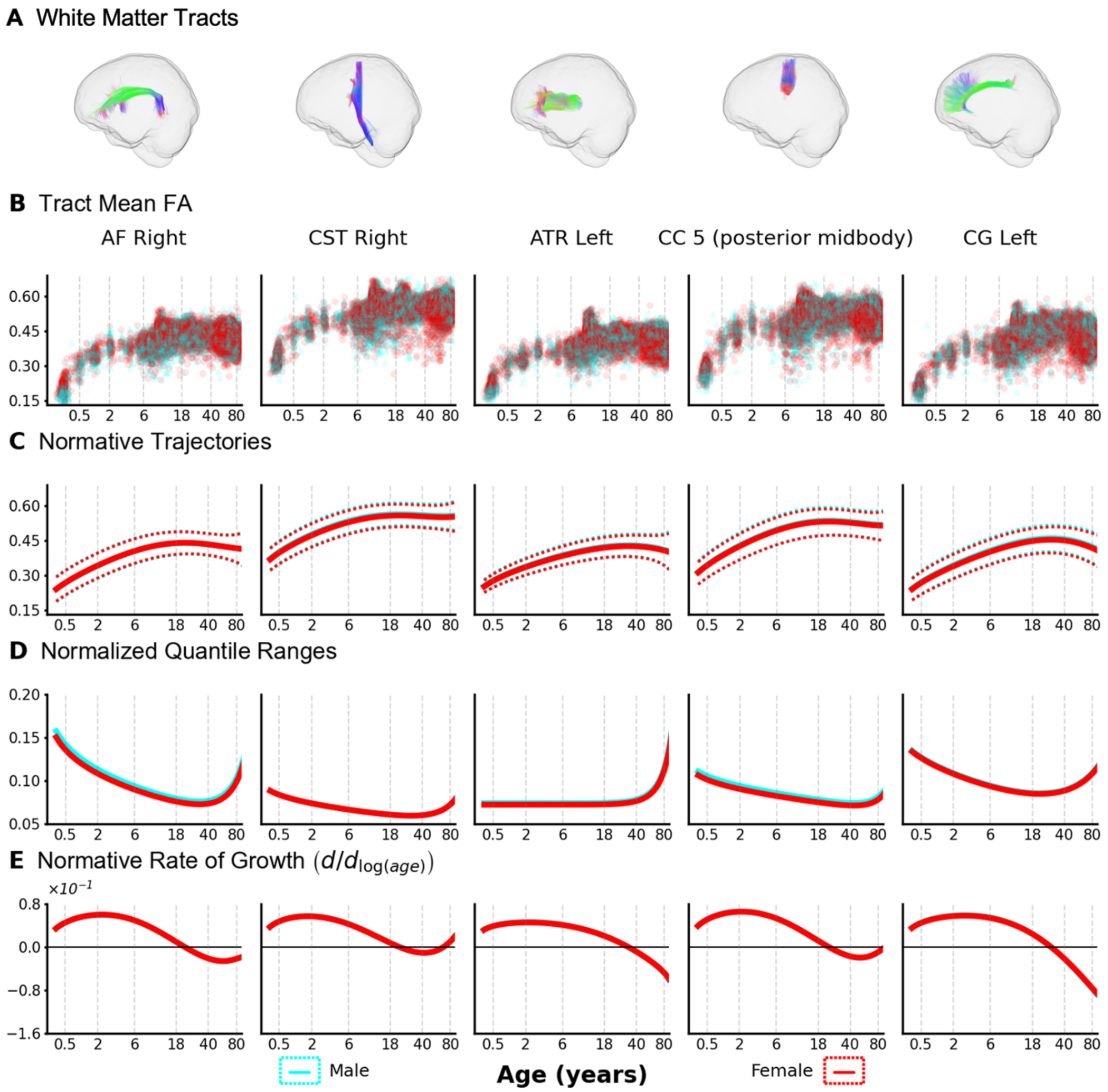

Supplemental Figure S1.F1. Tract-specific Microstructural Brain Charts - Contralateral. Lifespan brain charts for white matter microstructure (FA shown for contralateral tracts of Figure 2) reveal distinct developmental trajectories and variability across different WM pathways. A) Five exemplar tracts are shown (left to right): right arcuate fasciculus (AF Right); right corticospinal tract (CST Right); left anterior thalamic radiation (ATR Left); posterior midbody of the corpus callosum (CC 5 (posterior midbody)); and the left cingulum (CG Left). B) Raw FA data points for these tracts extend across the lifespan. C) Normative GAMLSS trajectories show FA increasing during development, plateauing in adulthood, and declining in later life, where the timing and magnitude vary by tract. Median (solid lines) and 2.5th/97.5th percentiles (dotted lines) are shown. D) Normalized quantile ranges indicate FA variability is generally lowest in middle age and increases later in life, though developmental variability patterns differ between tracts. E) The normative rate of change ($d/d_{\log(age)}$) suggests that FA peaks at different ages and changes at different rates depending on the specific tract. Note: The x-axes (age in years) are log-scaled to emphasize developmental and aging periods.

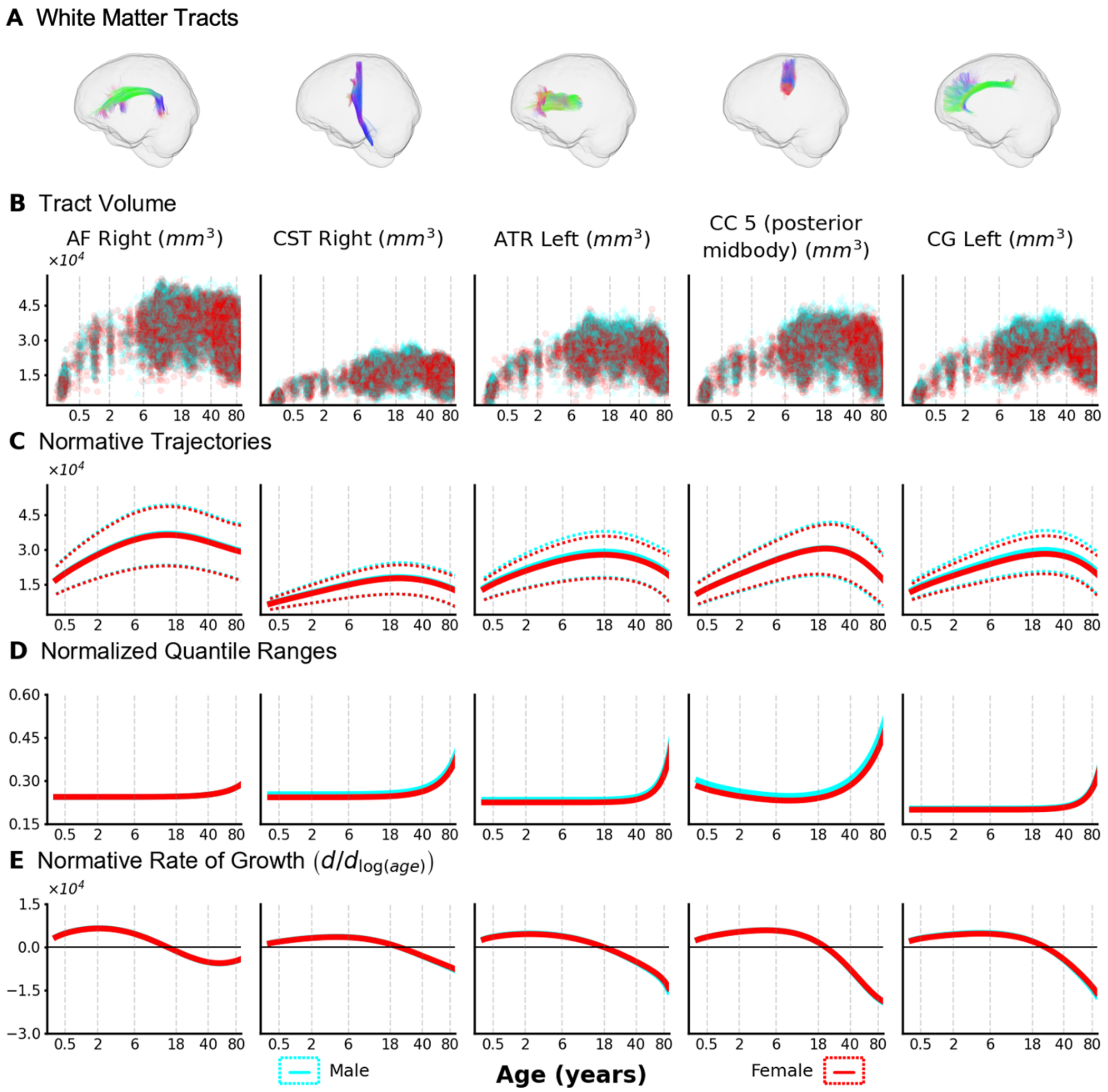

Supplemental Figure S1.F2. Tract-specific Macrostructural Brain Charts - Contralateral. Lifespan brain charts for white matter *macrostructure* (tract volume shown for contralateral tracts of Figure 3) reveal distinct developmental trajectories and variability across different WM pathways. A) Five exemplar tracts are shown (left to right): right arcuate fasciculus (AF Right); right corticospinal tract (CST Right); left anterior thalamic radiation (ATR Left); posterior midbody of the corpus callosum (CC 5 (posterior midbody)); and the left cingulum (CG Left). B) Raw tract volume data points for these tracts span the lifespan, illustrating differences in typical volume ranges between tracts. C) Normative GAMLSS trajectories show tract volume increasing during development, peaking in adolescence or early adulthood, and declining in later life, with tract-specific timing and magnitude. Median (solid lines) and 2.5th/97.5th percentiles (dotted lines) are shown. D) Normalized quantile ranges indicate that volume variability increases later in life and differs between sexes, being generally lowest in younger ages. E) The normative rate of change ($d/d_{\log(age)}$) suggests that tract volume peaks at different ages and the rate of subsequent decline varies depending on the specific tract. Note: The x-axes (age in years) are log-scaled to emphasize developmental and aging periods.

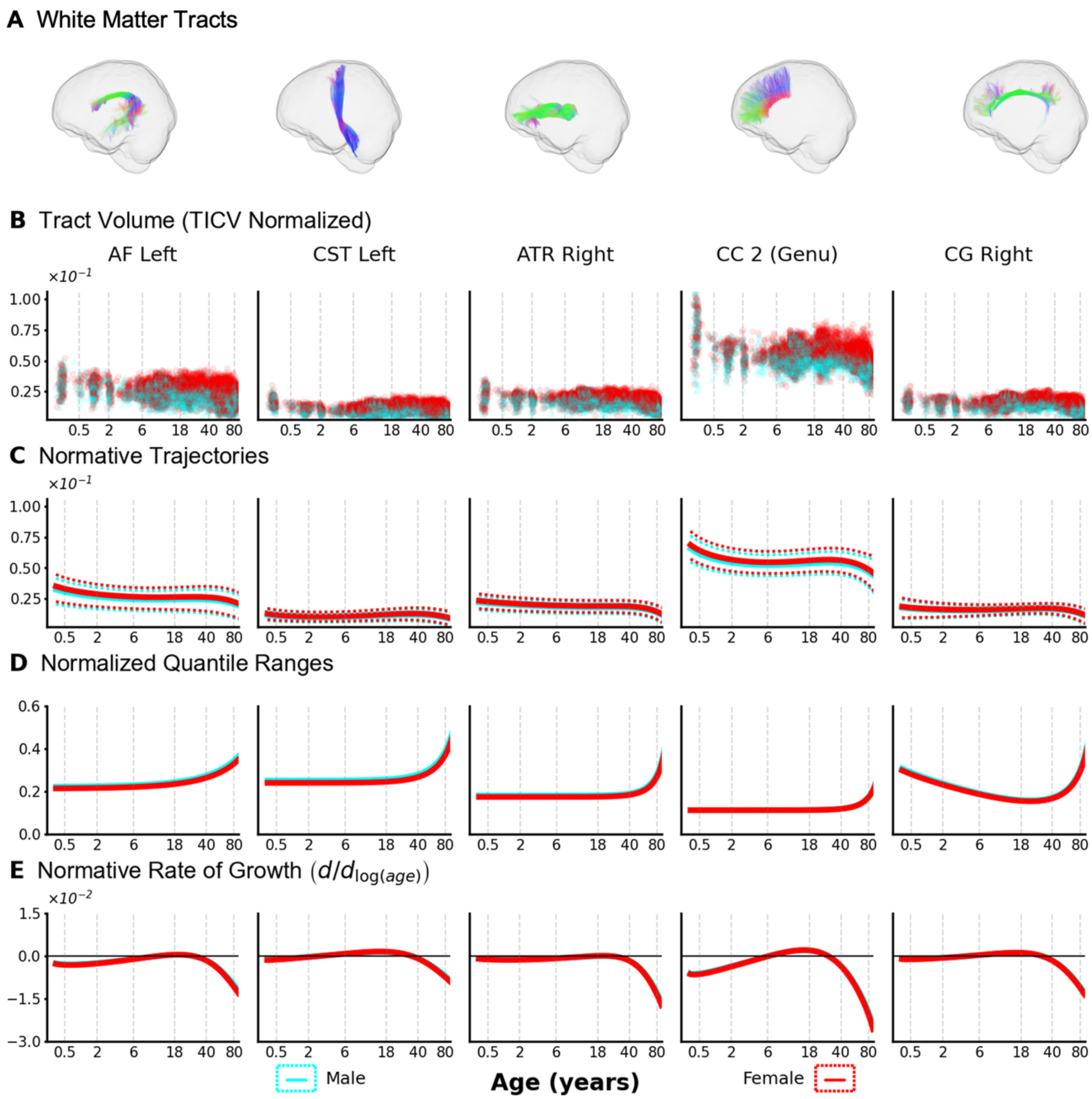

Supplemental Figure S1.F3. Charts for tract volume normalized by TICV. Lifespan trajectories of WM tract volume normalized by estimated total intracranial vault volume show most tracts decrease in relative volume in infancy, stabilize in middle age, and then decrease again in late life. A.) From left to right, left arcuate fasciculus; left corticospinal tract, right anterior thalamic radiation; genu of the corpus callosum; and the right cingulum. B.) Raw data points indicate that TICV-normalized volumes are higher in females than males, with this trend also appearing in C.) the lifespan trajectories for these measures. D.) Additionally, the normalized quantile ranges indicate that variability for TICV-normalized tract volume is lowest at the beginning of the lifespan and monotonically increases throughout life, with a very sharp increase in older age. E.) Like most other features, change is most rapid in infancy and decreases in magnitude in later age ranges.

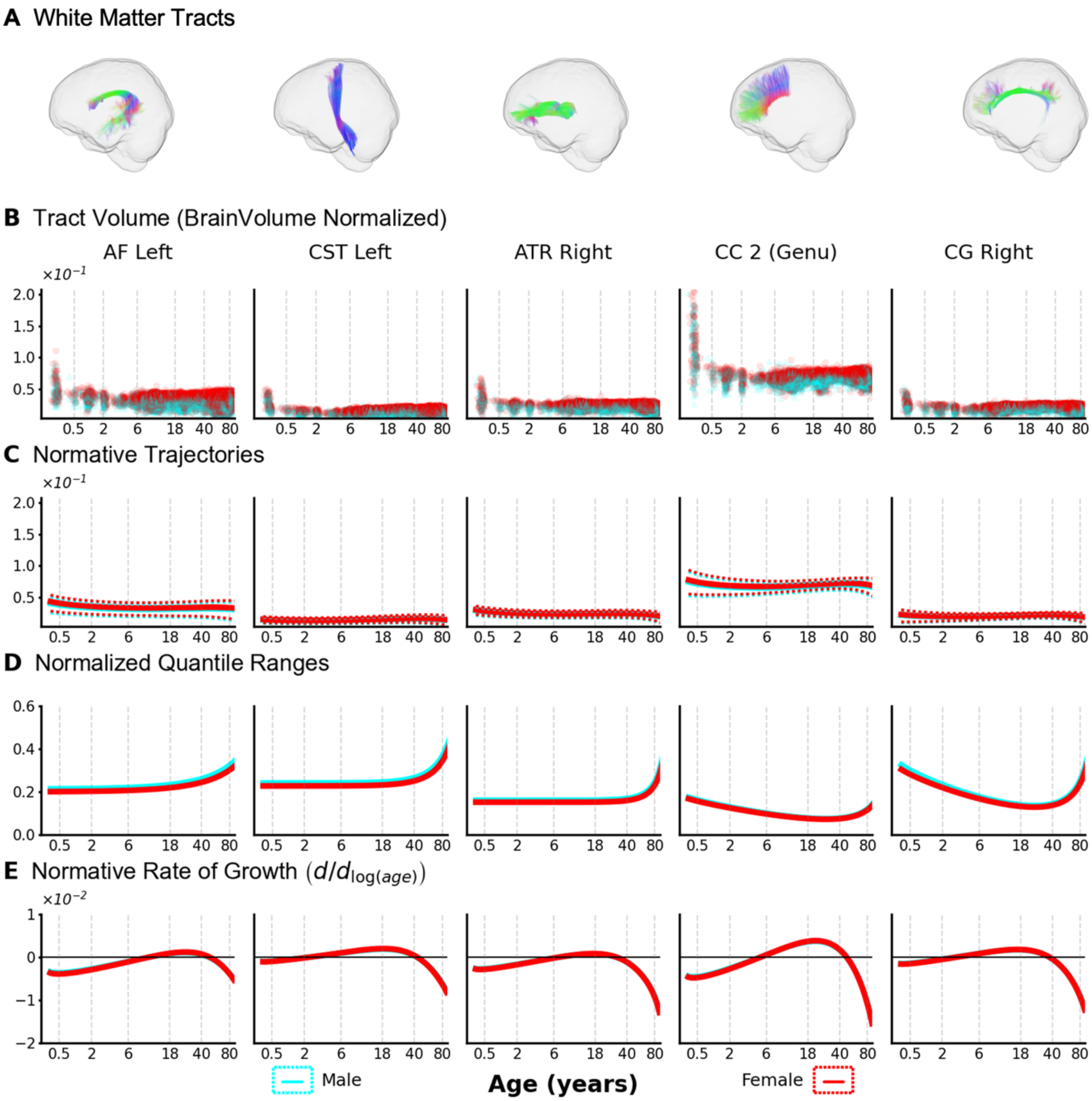

Supplemental Figure S1.F4. Charts for tract volume normalized by brain volume without ventricles. Lifespan trajectories of WM tract volume normalized by brain volume without ventricles show most tracts decrease in relative volume in infancy, stabilize or increase in middle age, and then decrease again in late life. A.) From left to right, left arcuate fasciculus; left corticospinal tract, right anterior thalamic radiation; genu of the corpus callosum; and the right cingulum. B.) Raw data points indicate that tract volumes normalized by brain volume without ventricles are higher in females than males, with this trend also appearing in C.) the lifespan trajectories for these measures. D.) Additionally, the normalized quantile ranges indicate that variability for WM tract volume normalized by brain volume without ventricles is similar compared to the non-normalized counterparts. E.) Like most other features, change is most rapid in infancy and decreases in magnitude in later age ranges.

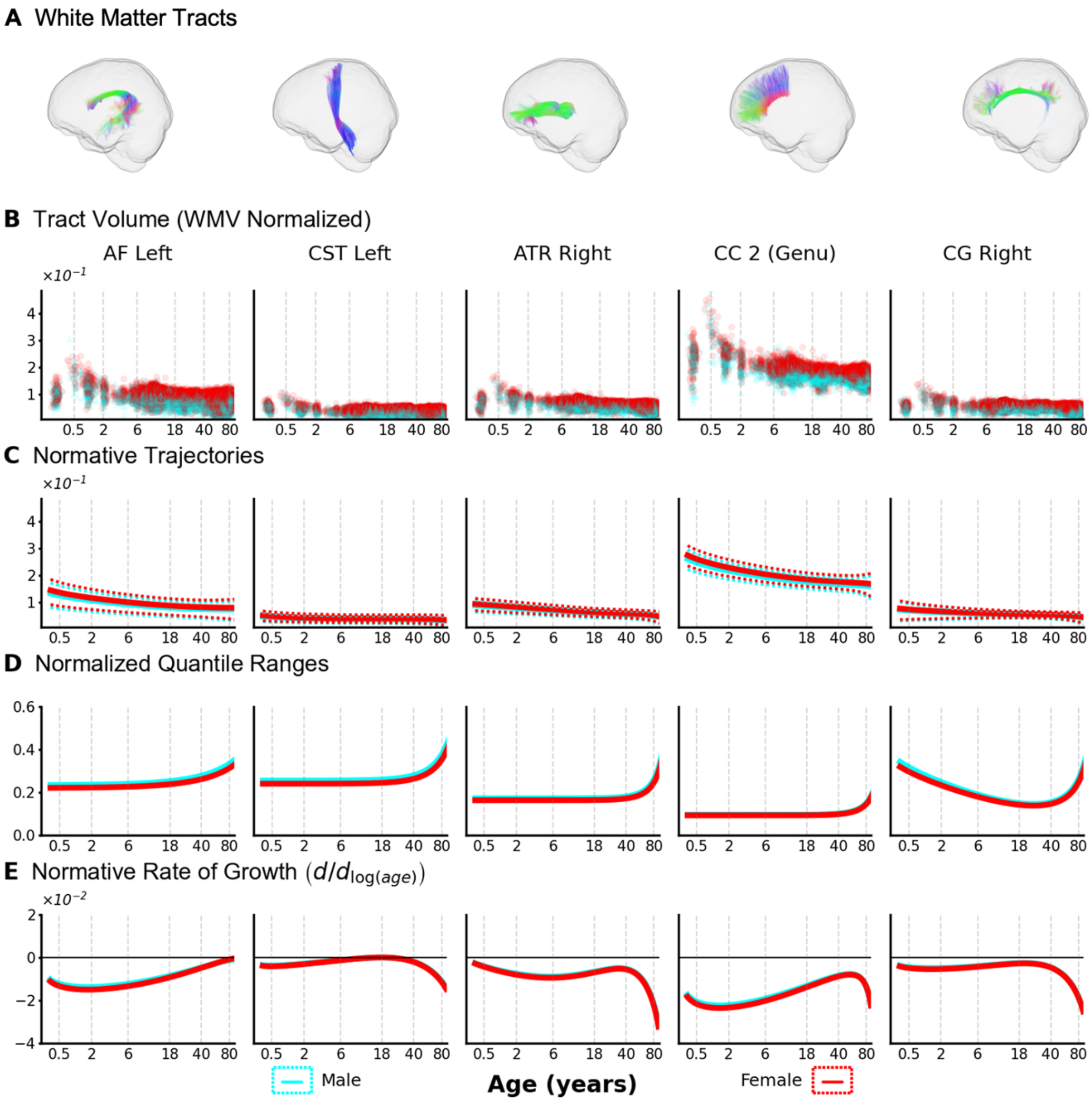

Supplemental Figure S1.F5. Charts for tract volume normalized by cerebral WM volume. Lifespan trajectories of WM tract volume normalized by cerebral WM volume show most tracts decrease in relative volume in infancy and then continue to decrease at lower rates throughout the rest of the lifespan. A.) From left to right, left arcuate fasciculus; left corticospinal tract, right anterior thalamic radiation; genu of the corpus callosum; and the right cingulum. B.) Raw data points indicate that tract volumes normalized by cerebral WM volume are higher in females than males, with this trend also appearing in C.) the lifespan trajectories for these measures. D.) The normalized quantile ranges indicate that variability is higher in males than females throughout the lifespan. Further, variability at the beginning of the lifespan is higher for some tracts when compared to the non-normalized counterparts. E.) Like most other features, change is most rapid in infancy and decreases in magnitude in later age ranges.

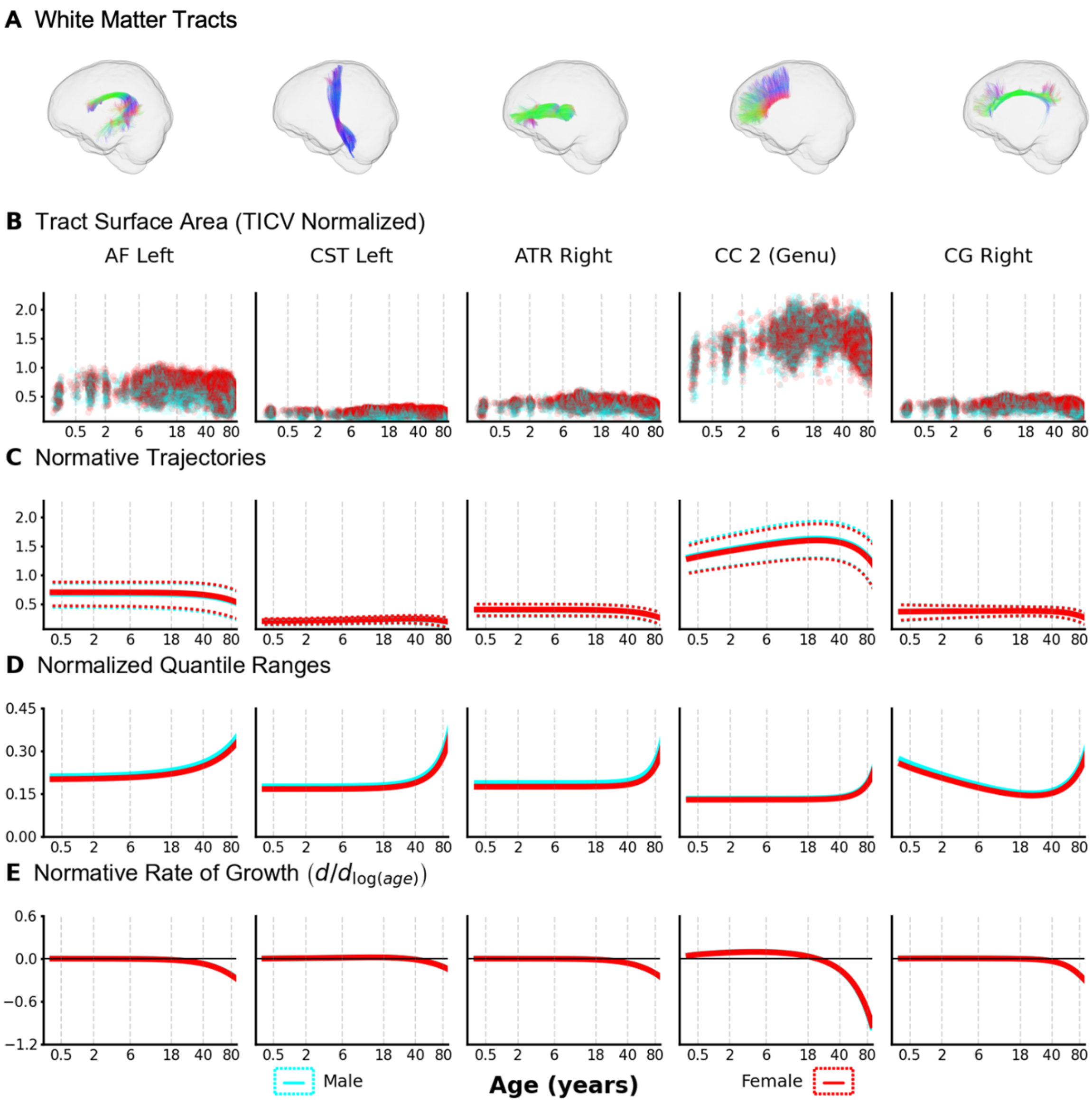

Supplemental Figure S1.F6. Charts for surface area normalized by TICV. Lifespan trajectories of WM surface area normalized by surface area derived from estimated total intracranial vault volume show tracts to have a small to zero increase in the ratio across most of the lifespan, with decreases in older age. A.) From left to right, left arcuate fasciculus; left corticospinal tract, right anterior thalamic radiation; genu of the corpus callosum; and the right cingulum. B.) Raw data points indicate that sex-specific trends in tract surface areas normalized by surface area derived from estimated total intracranial vault volume are tract specific, with some tracts having larger ratios in males and vice versa. This trend also is apparent in C.) the lifespan trajectories for these measures. D.) The normalized quantile ranges indicate that variability is higher in males than females throughout the lifespan. Further, variability is similar for tracts when compared to the non-normalized counterparts, with slight decreases for the normalized trajectories. E.) Unlike most other features, the values do not change across most of the lifespan, however, they do decrease towards the end of the lifespan like most other macrostructural features.

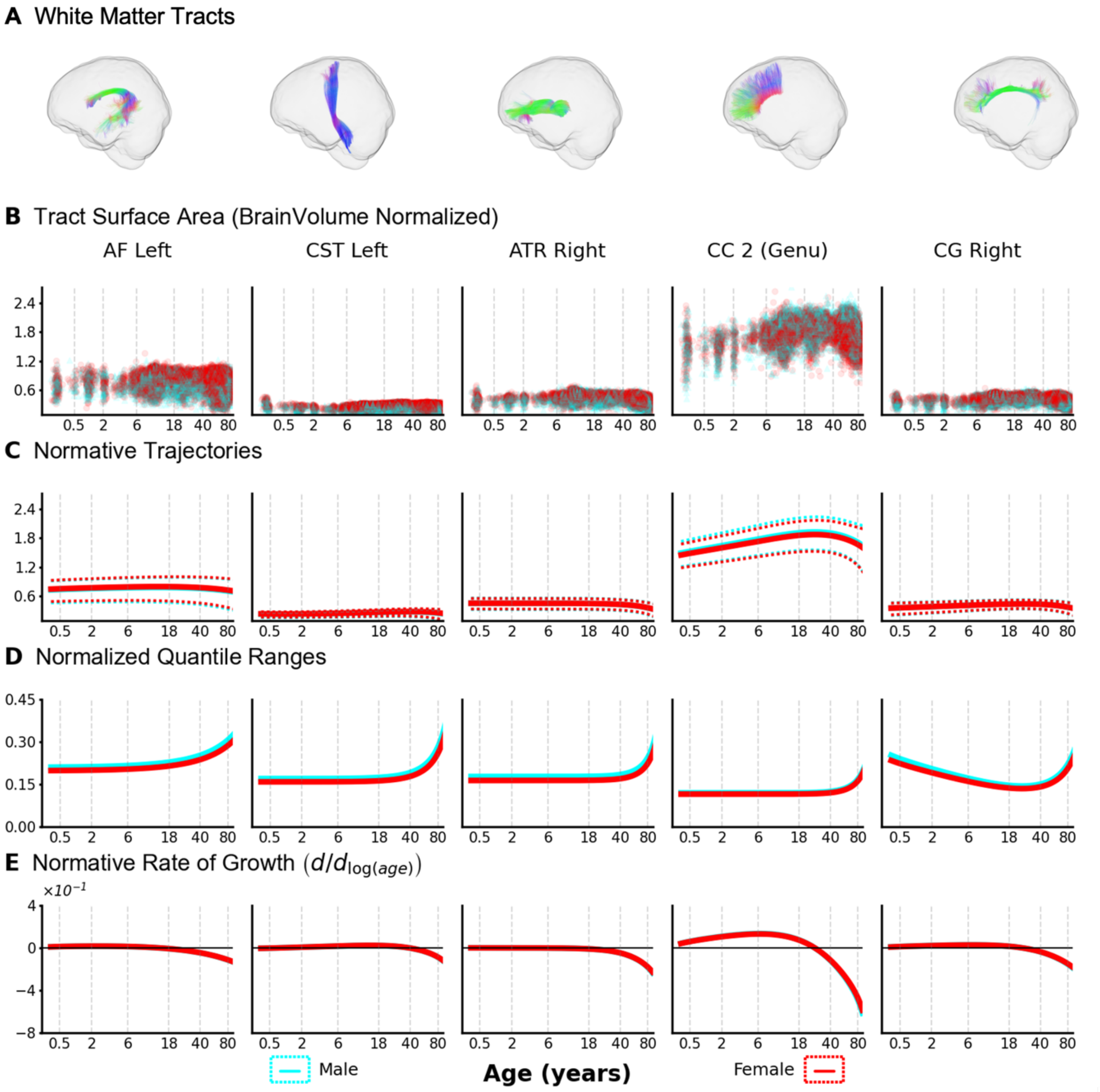

Supplemental Figure S1.F7. Charts for surface area normalized by brain volume without ventricles. Lifespan trajectories of WM surface area normalized by surface area derived from brain volume without ventricles show tracts to have a small to zero increase in the ratio across most of the lifespan, with decreases in older age. A.) From left to right, left arcuate fasciculus; left corticospinal tract, right anterior thalamic radiation; genu of the corpus callosum; and the right cingulum. B.) Raw data points indicate that sex-specific trends in tract surface areas normalized by surface area derived from brain volume without ventricles are tract specific, with some tracts having larger ratios in males and vice versa. This trend also is apparent in C.) the lifespan trajectories for these measures. D.) The normalized quantile ranges indicate that variability is higher in males than females throughout the lifespan. Further, variability is similar for tracts when compared to the non-normalized counterparts, with slight decreases for the normalized trajectories. E.) Unlike most other features, the values do not change across most of the lifespan, however, they do decrease towards the end of the lifespan like most other macrostructural features.

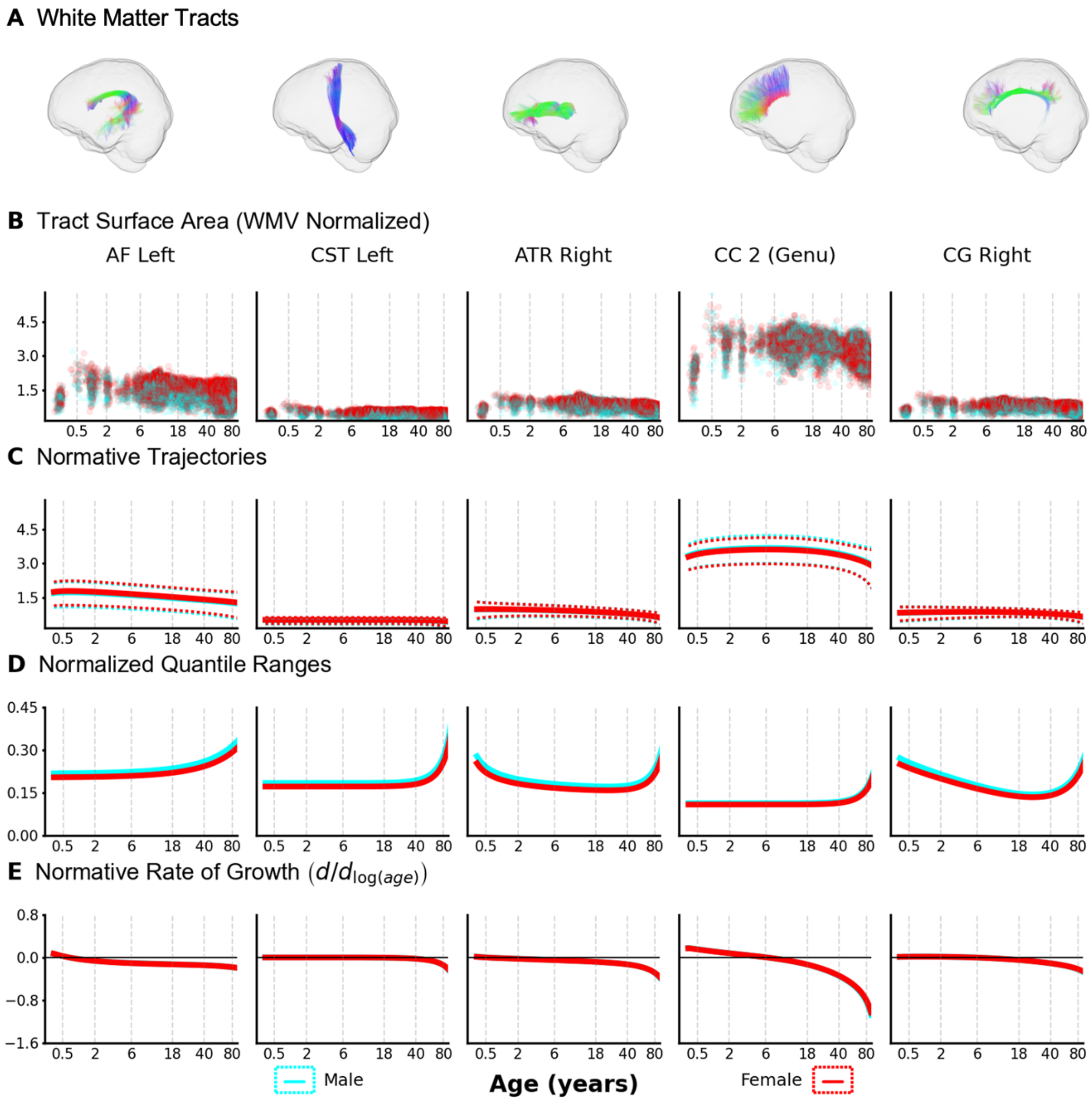

Supplemental Figure S1.F8. Charts for surface area normalized by cerebral WM volume. Lifespan trajectories of WM surface area normalized by surface area derived from cerebral WM volume show tracts to have a small decrease in the ratio across most of the lifespan, with larger decreases in older age. A.) From left to right, left arcuate fasciculus; left corticospinal tract, right anterior thalamic radiation; genu of the corpus callosum; and the right cingulum. B.) Raw data points indicate that sex-specific trends in tract surface areas normalized by surface area derived from cerebral WM volume are tract specific, with some tracts having larger ratios in males and vice versa. This trend also is apparent in C.) the lifespan trajectories for these measures. D.) The normalized quantile ranges indicate that variability is slightly higher in males than females throughout the lifespan. Further, variability is similar for tracts when compared to the non-normalized counterparts. E.) Unlike most other features, the values decrease across the entire lifespan for most tracts.

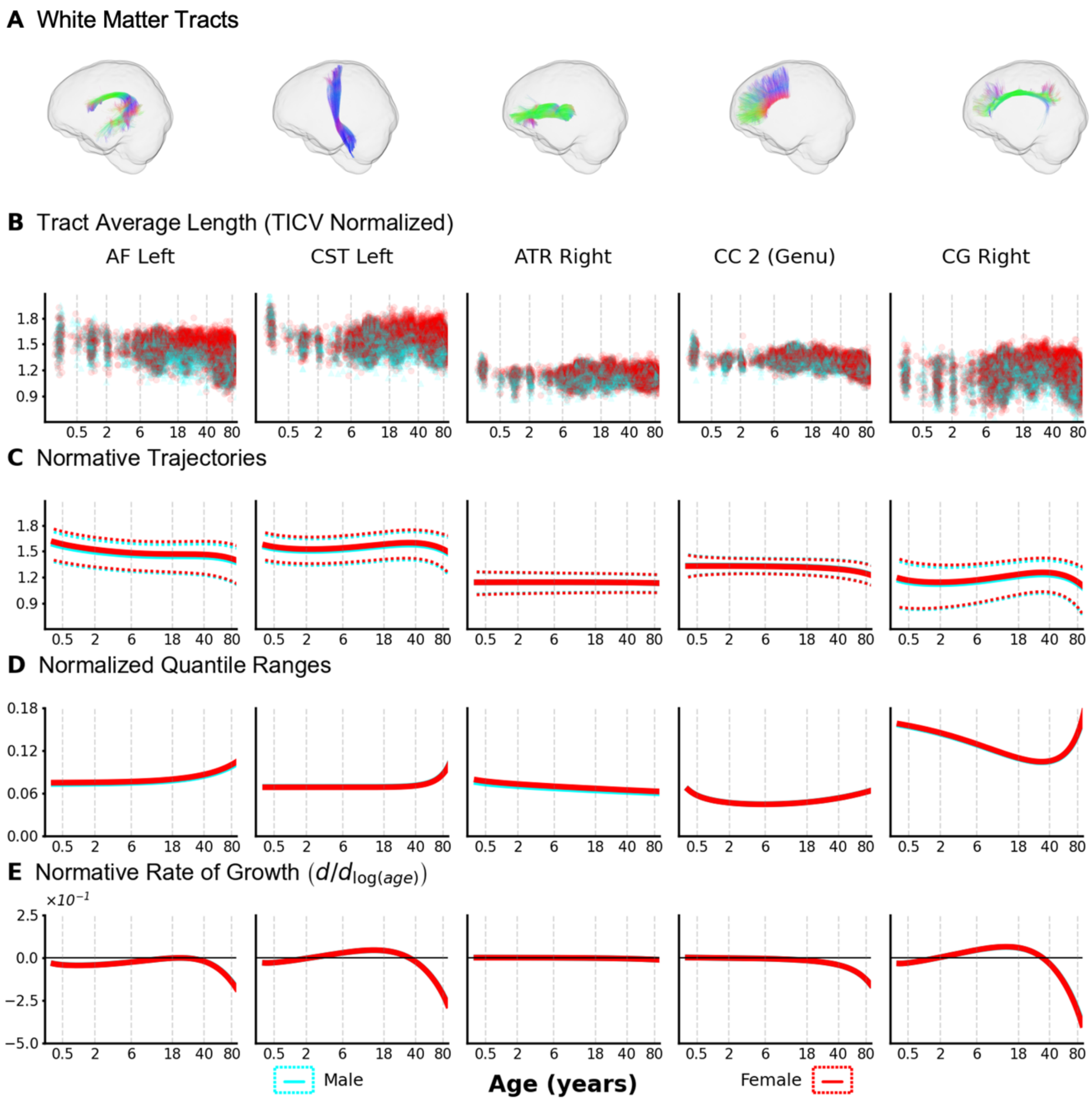

Supplemental Figure S1.F9. Charts for average tract length normalized by TICV. Lifespan trajectories of average tract length normalized by radius derived from estimated total intracranial volume (TICV) show tracts to be highly variable with increasing age. A.) From left to right, left arcuate fasciculus; left corticospinal tract, right anterior thalamic radiation; genu of the corpus callosum; and the right cingulum. B.) Raw data points indicate that tract average lengths normalized by radius derived from estimated total intracranial volume are slightly larger in females than males. This trend also is apparent in C.) the lifespan trajectories for these measures. D.) The normalized quantile ranges indicate that variability is similar between males and females throughout the lifespan. Further, variability is similar for tracts when compared to the non-normalized counterparts. E.) Unlike non-normalized average tract length, the values for TICV radius-normalized average tract length do not have sharp increases at the beginning of the lifespan.

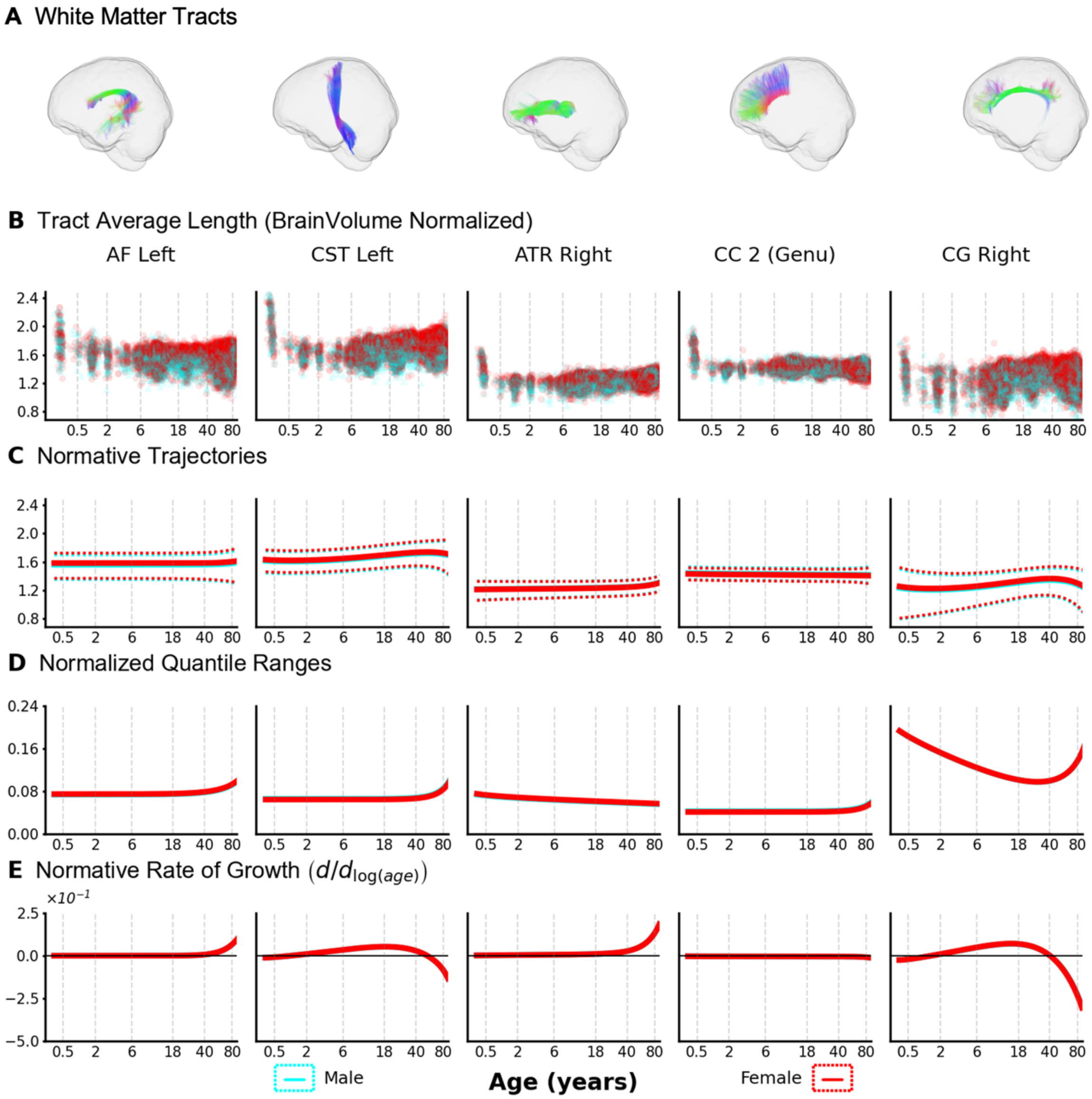

Supplemental Figure S1.F10. Charts for average tract length normalized by brain volume without ventricles. Lifespan trajectories of average tract length normalized by radius derived from brain volume without ventricles show tracts to be highly variable with increasing age. A.) From left to right, left arcuate fasciculus; left corticospinal tract, right anterior thalamic radiation; genu of the corpus callosum; and the right cingulum. B.) Raw data points indicate that tract average lengths normalized by radius derived from brain volume without ventricles are similar for males and females. This trend also is apparent in C.) the lifespan trajectories for these measures. D.) The normalized quantile ranges indicate that variability is similar between males and females throughout the lifespan. Further, variability is similar for tracts when compared to the non-normalized counterparts. E.) Unlike non-normalized average tract length, the values for average tract length normalized by radius from brain volume without ventricles increase at the end of the lifespan for some tracts.

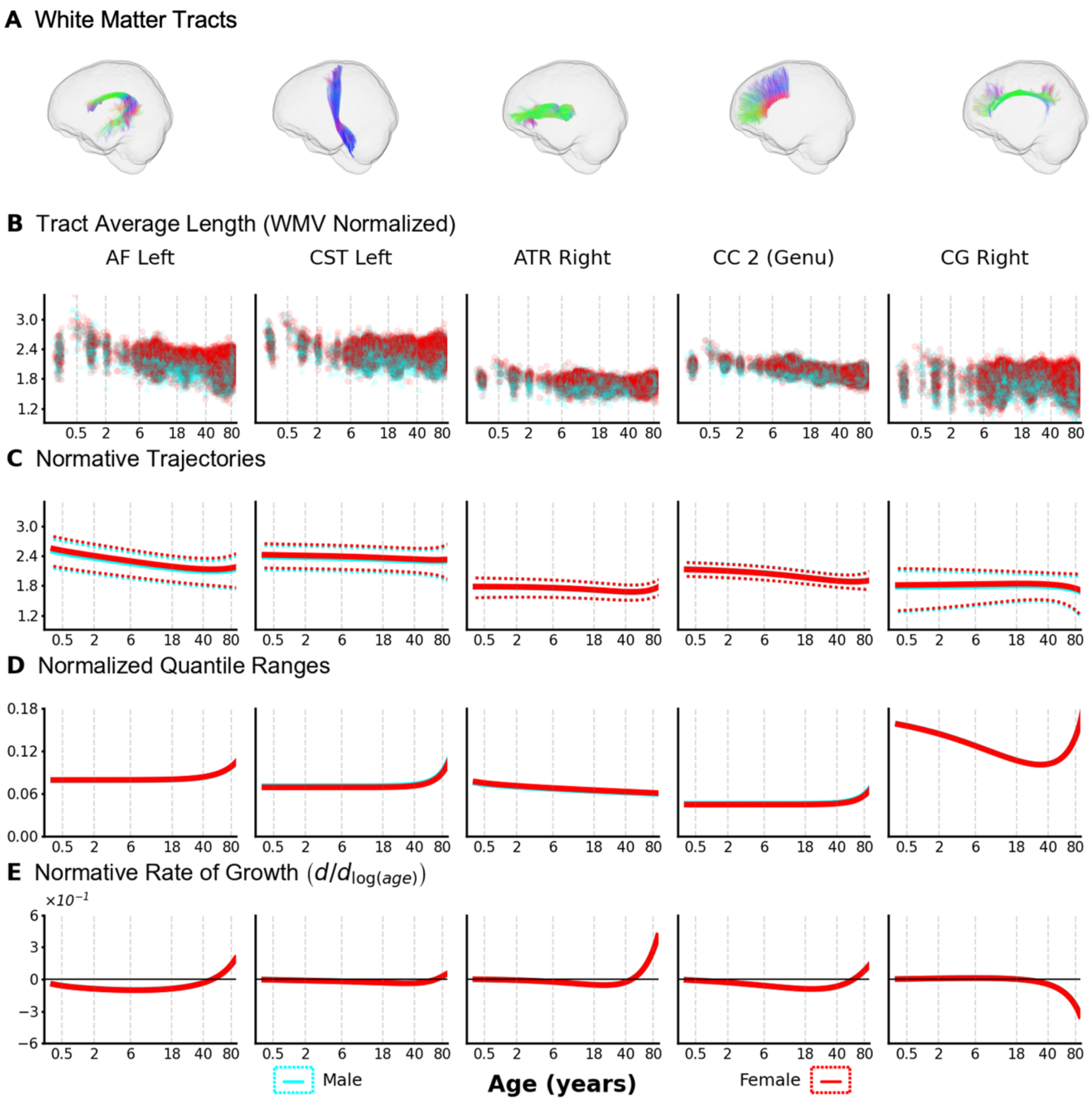

Supplemental Figure S1.F11. Charts for average tract length normalized by cerebral WM volume. Lifespan trajectories of average tract length normalized by radius derived from cerebral WM volume show tracts to mostly decrease with increasing age. A.) From left to right, left arcuate fasciculus; left corticospinal tract, right anterior thalamic radiation; genu of the corpus callosum; and the right cingulum. B.) Raw data points indicate that tract average lengths normalized by radius derived from cerebral WM volume are similar for males and females, with female trajectories sitting slightly higher for some tracts. This trend also is apparent in C.) the lifespan trajectories for these measures. D.) The normalized quantile ranges indicate that variability is similar between males and females throughout the lifespan. Further, variability is similar for tracts when compared to the non-normalized counterparts. E.) Unlike non-normalized average tract length, the values for average tract length normalized by radius from brain volume without ventricles increase at the end of the lifespan for some tracts.

2. Data Information

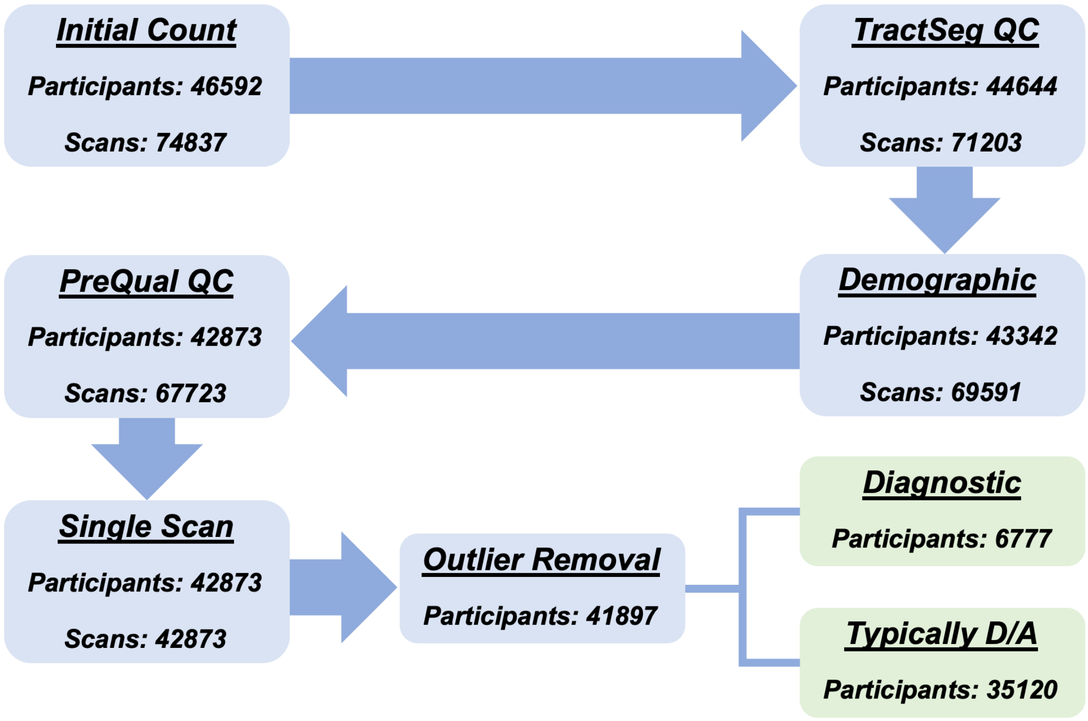

Supplemental Figure S2.F1. Flowchart of Quality Control Sample Sizes. Starting with data from 46,592 participants, the several rounds of quality control procedures resulted in a total of 35,120 typically developing and aging participants that were used to create the WM brain charts and 6,777 participants coming from various diagnostic groups.

Supplemental Table S2.T1. Demographic information for the typically developing and aging participants used to create the WM brain charts.

Race Category	Hispanic/Latino		Non-Hispanic/Latino		Ethnicity Unreported		Total
	Female	*Male*	*Female*	*Male*	*Female*	*Male*	
**American Indian/Alaska**	2	0	7	7	7	4	*27*
**Asian**	1	1	63	55	133	141	*394*
**Black or African American**	2	4	430	205	567	288	*1496*
**White**	53	51	1537	992	6413	4827	*12873*
**More than One**	36	52	44	59	70	52	*313*
**Unknown**	134	186	3667	3977	5774	5278	*19016*
Total	*228*	*294*	*5748*	*5295*	*12965*	*10590*	*35120*

Total Number of Males: *16179* | ***Total Number of Females:*** *18941*

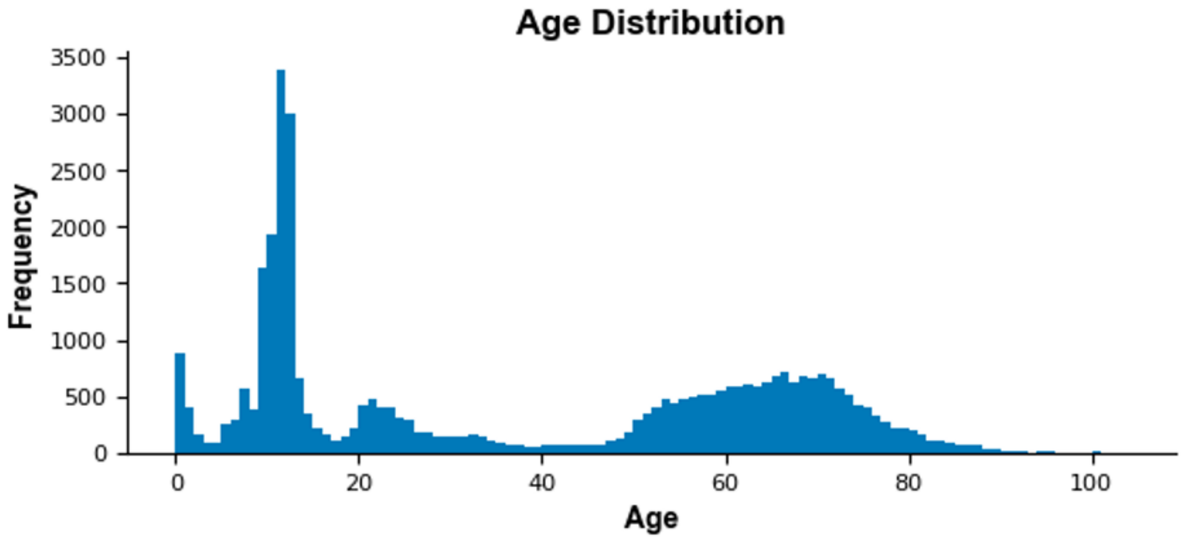

Supplemental Figure S2.F2. Age histogram for participants used in normative modeling. Note that ages are binned in 1-year intervals.

Supplemental Table S2.T2. Acquisition information for dMRI data from each dataset, along with the respective scanning/facility location.

Dataset	Voxel Size	Acquisition Parameters	Facility Location
ABCD[1]	1.7 x 1.7 x 1.7	7 b0; 6 b500; 15 b1000; 15 b2000; 60 b3000	Varied (USA)
ADNI[2]	2.0 x 2.0 x 2.0	(Varied) 7 b0; 48 b1000	Varied (USA)
Ageility[3]	2.0 x 2.0 x 2.0	1 b0; 64 b1000; 64 b3000	Newcastle, Australia
AOMIC ID1000[4]	2.0 x 2.0 x 2.0	1 b0; 32 b1000 [sequence acquired x3]	Amsterdam, Netherlands
AOMIC PIOP1[4]	2.0 x 2.0 x 2.0	1 b0; 32 b1000	Amsterdam, Netherlands
AOMIC PIOP2[4]	2.0 x 2.0 x 2.0	1 b0; 32 b1000	Amsterdam, Netherlands
BANDA[5]	1.5 x 1.5 x 1.5	28 b0; 190 b1500; 190 b3000	Boston, Massachusetts (USA)
BIOCARD[6]	0.828 x 0.828 x 2.2	1 b0; 32 b700	Baltimore, Maryland (USA)
BLSA[7]	0.8125 x 0.8125 x 2.2$	1 b0; 32 b700	Baltimore, Maryland (USA)
BSNIP1[8]	1.72 x 1.72 x 3.0	1 b0; 32 b1000	Varied (USA)
BSNIP2[9]	2.0 x 2.0 x 2.0	7 b0; 64 b900	Varied (USA)
Calgary[10]	0.78 x 0.78 x 2.2	5 b0; 30 b800	Calgary, Canada
CALM[11]	2.0 x 2.0 x 2.0	5 b0; 64 b1000	Cambridge, England
CAMCAN[12]	2.0 x 2.0 x 2.0	3 b0; 30 b1000; 30 b2000	Cambridge, England
VUMC-ASD	2.5 x 2.5 x 2.5	1 b0; 92 b1600<br>3 b0; 6 b500; 32 b1000; 64 b2000	Nashville, Tennessee (USA)
CUTTING	2.5 x 2.5 x 2.5	1 b0; 60 b2000	Nashville, Tennessee (USA)
dHCP[13]	1.5 x 1.5 x 1.5	20 b0; 64 b400; 88 b1000; 128 b2600	United Kingdom
DLBS[14]	1.75 x 1.75 x 3.0	1 b0; 30 b1000	Dallas, Texas (USA)
EBDT[15]	2.0 x 2.0 x 2.0	7 b0; 42 b1000	Chapel Hill, North Carolina (USA)
HABSHD[16]	1.72 x 1.72 x 2.5	4 b0; 64 b1000 [sequence acquired x3]	Fort Worth, Texas (USA)
HBCD[17]	1.7 x 1.7 x 1.7	22 b0; 12 b500; 24 b1000; 36 b2000; 58 b3000	Varied (USA)
HBN[18]	1.8 x 1.8 x 1.8	1 b0; 64 b1000; 64 b2000	Varied (USA)
HCP[19]	1.25 x 1.25 x 1.25	18 b0; 90 b1000; 90 b2000; 90 b3000	St. Louis, Missouri; Minneapolis, Michigan (USA)
HCPA[20]	1.5 x 1.5 x 1.5	28 b0; 186 b1500; 184 b3000	St. Louis, Missouri; Minneapolis, Michigan (USA)
HCPBaby[21]	1.5 x 1.5 x 1.5	7 b0; 18 b500; 24 b1000; 108 b1500; 48 b2000; 68 b2500; 170 b3000	Chapel Hill, North Carolina; Minneapolis, Michigan (USA)
HCPD[22]	1.5 x 1.5 x 1.5	28 b0; 186 b1500; 184 b3000	St. Louis, Missouri; Minneapolis, Michigan (USA)
ABC-Babies	1.78 x 1.78 x 2.2	1 b0; 32 b700; 64 b2000	Nashville, Tennessee (USA)
IBIS[23]	2.0 x 2.0 x 2.0	1 b0; multi-shell (b=100-1000 in steps of 100), 2-3 volumes/shell	Varied (USA)
ICBM[24]	1.25 x 1.25 x 2.5	5 b0; 30 b1000	Los Angeles, California (USA)

Lexical[25]	2.0 x 2.0 x 2.0	1 b0; 64 b1000	Chicago, Illinois (USA)
MASiVar[26]	2.14 x 2.14 x 2.2	34 b0; 86 b1000; 112 b2000	Boston, Massachusetts (USA)
MORGAN	2.5 x 2.5 x 2.5	1 b0; 92 b1600	Nashville, Tennessee (USA)
NACC[27]	2.0 x 2.0 x 2.0	(Varied) 1 b0; 64 b1000	Varied (USA)
NKI[28]	2.0 x 2.0 x 2.0	9 b0; 128 b1500	Rockland County, New York (USA)
PING[29]	2.5 x 2.5 x 2.5	1 b0; 32 b1000	Varied (USA)
QTAB[30]	2.0 x 2.0 x 2.0	3 b0; 5 b1000; 15 b3000	Queensland, Australia
ROS[31]	2.0 x 2.0 x 2.0	6 b0; 40 b1000	Chicago, Illinois (USA)
MAP[32]	2.0 x 2.0 x 2.0	6 b0; 40 b1000	Chicago, Illinois (USA)
MARS[33]	2.0 x 2.0 x 2.0	1 b0; 40 b1000	Chicago, Illinois (USA)
SCAN[34]	2.0 x 2.0 x 2.0	13 b0; 6 b500; 48 b1000; 60 b2000	Varied (USA)
SWU[35]	2.0 x 2.0 x 2.0	3 b0; 30 b1000	Chongqing, China
TempleSocial[36,37]	2.0 x 2.0 x 2.0	3 b0; 6 b300; 21 b1000; 24 b2000; 12 b3200; 19 b3300; 61 b5000	Philadelphia, Pennsylvania (USA)
UCLA_LA5c[38]	1.98 x 1.98 x 2.0	1 b0; 64 b1000	Los Angeles, California (USA)
UKBB[39]	2.02 x 2.02 x 2	8 b0; 50 b1000; 50 b2000	United Kingdom
UPennRisk[40,41]	1.875 x 1.875 x 2.0	1 b0; 30 b1000	Philadelphia, Pennsylvania (USA)
UTAustin579[42]	2.0 x 2.0 x 4.0	6 b0; 64 b800	Austin, Texas (USA)
VMAP_2.0[43]	2.33 x 2.33 x 2.5	1 b0; 38 b1000; 56 b2000	Nashville, Tennessee (USA)
VMAP[43]	2.0 x 2.0 x 2.0	1 b0; 32 b1000	Nashville, Tennessee (USA)
TN Alzheimer's Project	2.33 x 2.33 x 2.5	1 b0; 38 b1000; 56 b2000	Nashville, Tennessee (USA)
WRAP[44]	0.9375 x 0.9375 x 2.5	8 b0; 40 b1300	Madison, Wisconsin ((USA)

$ BLSA was acquired at 2.2 x 2.2 x 2.2 mm^3, but resampled to 0.8125 x 0.8125 x 2.2 mm^3 in k-space

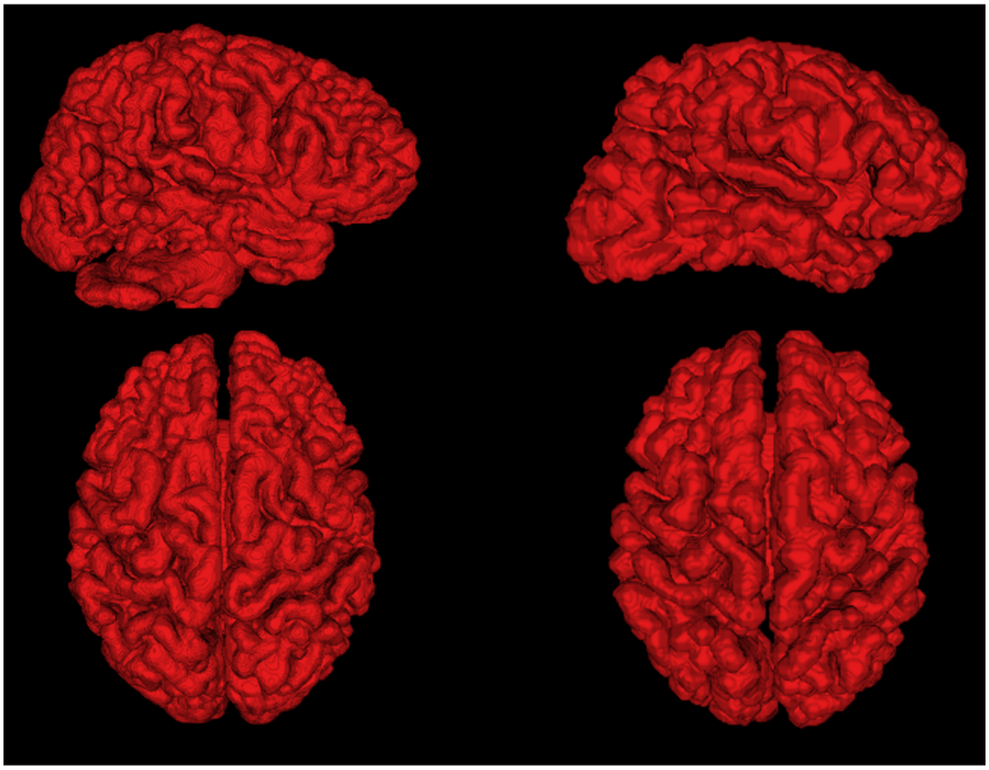

Supplemental Figure S2.F3. Comparison of TractSeg vs FreeSurfer white matter coverage. The overlap in coverage between (left) the aggregate of all TractSeg tracts and (right) the white matter mask from FreeSurfer is substantial. Visualizations of both come from the same scan of a single participant. The most notable difference is that the FreeSurfer mask does not include the cerebellar or brainstem regions.

3. **Additional Diagnostic Group Centile Deviations**

For sample sizes of each diagnostic cohort, as well as the datasets they originate from, please see **Extended Data Table ED.T3**.

i. Centile Score Deviations of Other Diagnostic Groups

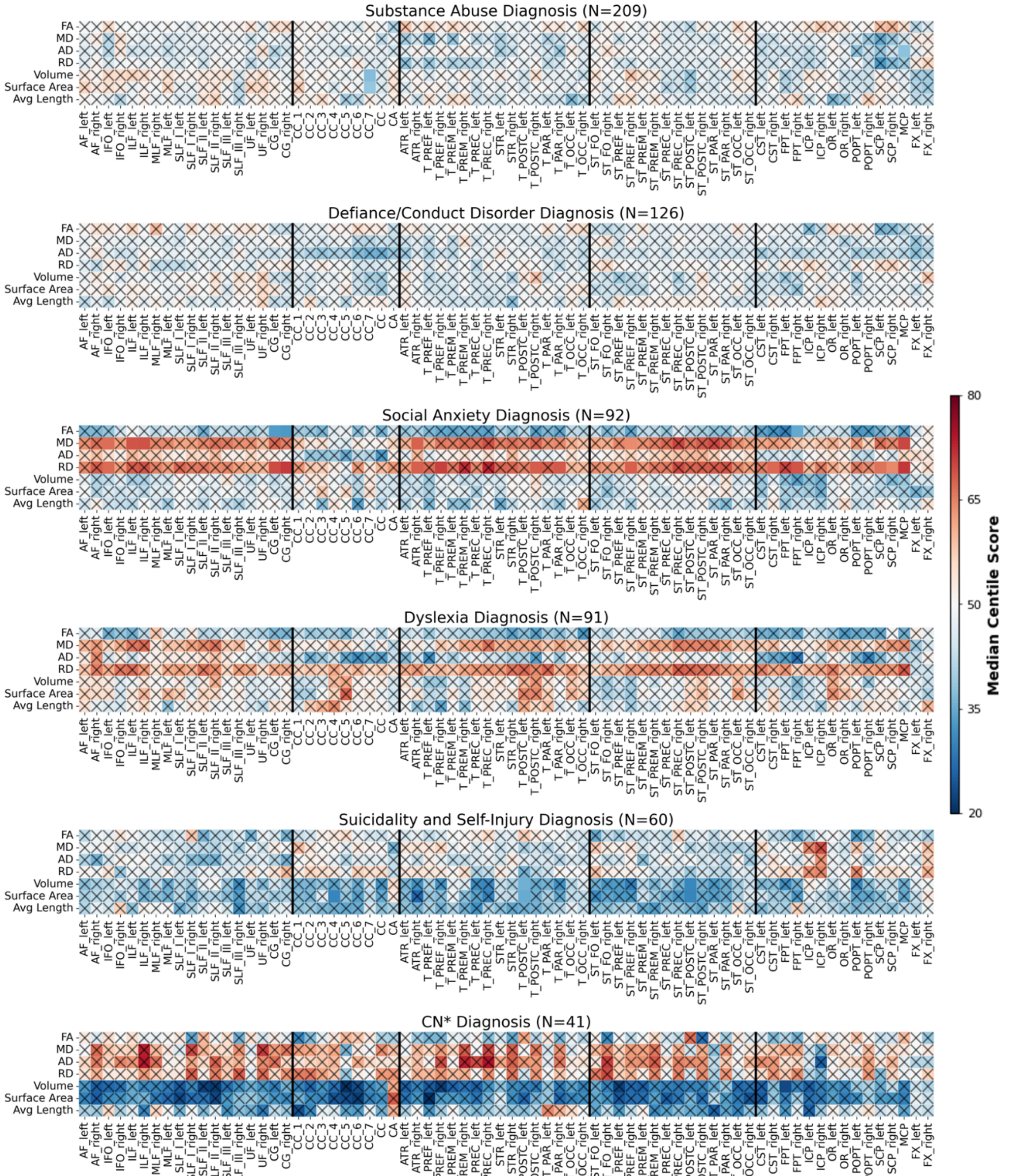

Supplemental Figure S3.F1. Centile score differences across other neurocognitive disorders with few or sparse significant deviations. For many other clinical cohorts (from top to bottom: substance abuse,

defiance/conduct disorders, social anxiety disorder, dyslexia, suicidality and self-injury, and individuals who start as cognitively unimpaired and then transition to cognitive impairment, or CN*), we do not observe significant deviations in centile scores. However, there are still non-significant patterns across tracts, such as reduced tract macrostructure in CN* participants and increased MD/RD in the social anxiety cohort. Gray "X" indicates features where the median centile was not significantly different from the 50th normative centile under a two-sided one-sample Wilcoxon signed-rank test following Bonferroni correction for multiple comparisons (significant at $p < 9.92 * 10^{-5}$).

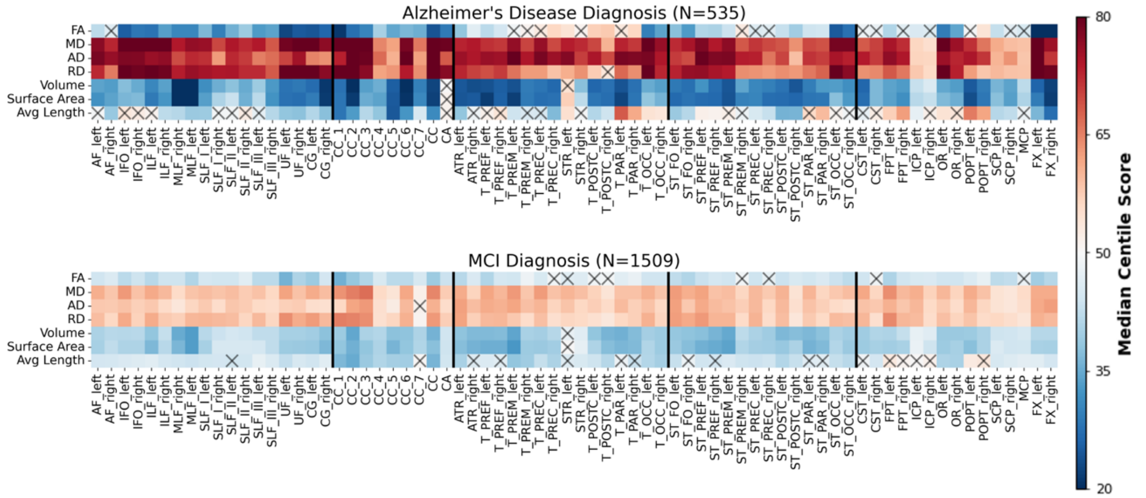

Supplemental Figure S3.F2. Centile score differences across MCI and Alzheimer's Disease – FDR-BH correction. As an alternative analysis to Bonferroni correction, we also employ Benjamini-Hochberg false discovery rate (FDR-BH) correction (significance threshold of $\alpha = 0.05$) when assessing significance of centile score differences across tracts and features for Alzheimer's disease (top) and mild cognitive impairment (MCI) cohorts under a two-sided one-sample Wilcoxon signed-rank test. Regardless of the correction method, we observe widespread deviations across tracts and features. Gray "X" indicates features where the median centile was not significantly different from the 50th normative centile.

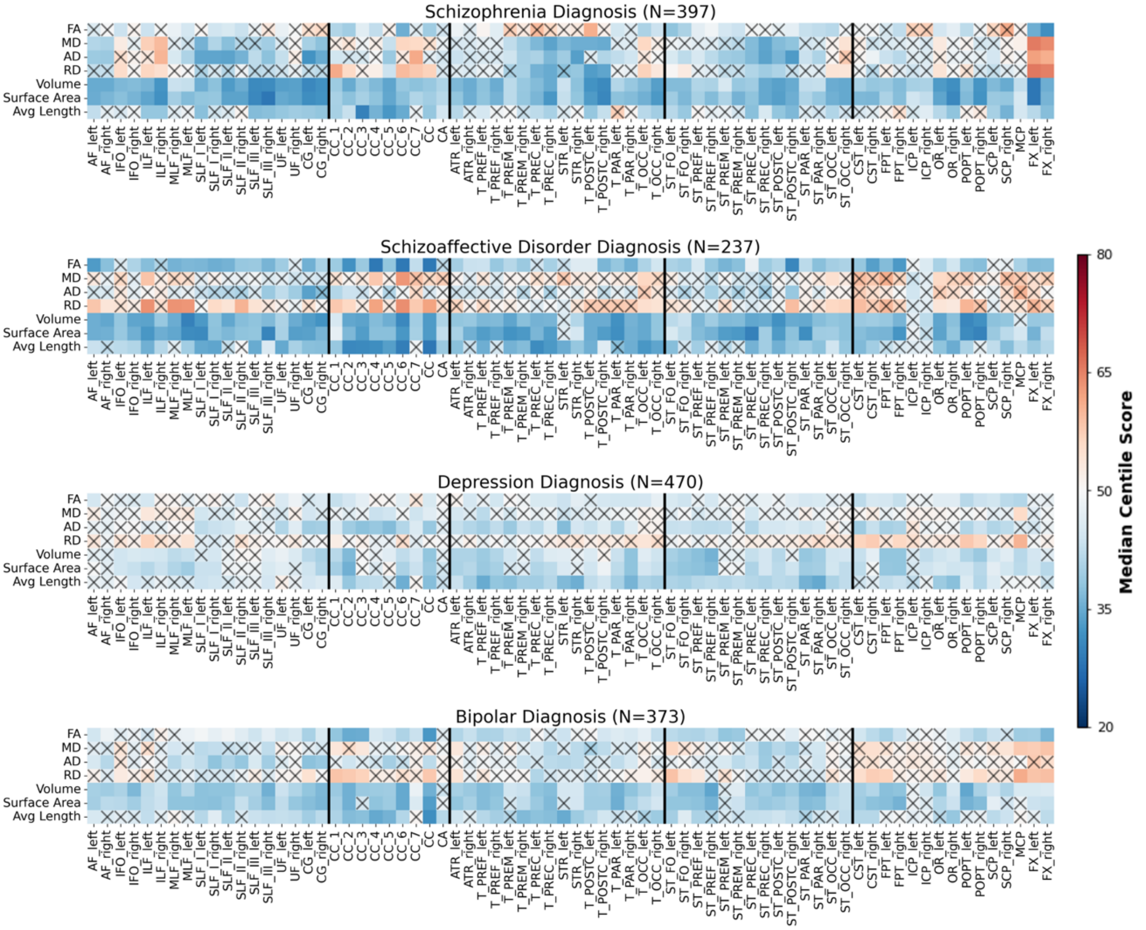

Supplemental Figure S3.F3. Centile score differences across major depressive and psychotic disorders – FDR-BH correction. As an alternative analysis to Bonferroni correction, we also employ Benjamini-Hochberg false discovery rate (FDR-BH) correction (significance threshold of $\alpha = 0.05$) when assessing significance of centile score differences across tracts and features for major psychoses and major depressive disorder (top to bottom: schizophrenia, schizoaffective disorder, major depressive disorder, and bipolar disorder) under a two-sided one-sample Wilcoxon signed-rank test. All cohorts show widespread reductions in tract macrostructure, with localized reductions in tract microstructure. Gray "X" indicates features where the median centile was not significantly different from the 50th normative centile.

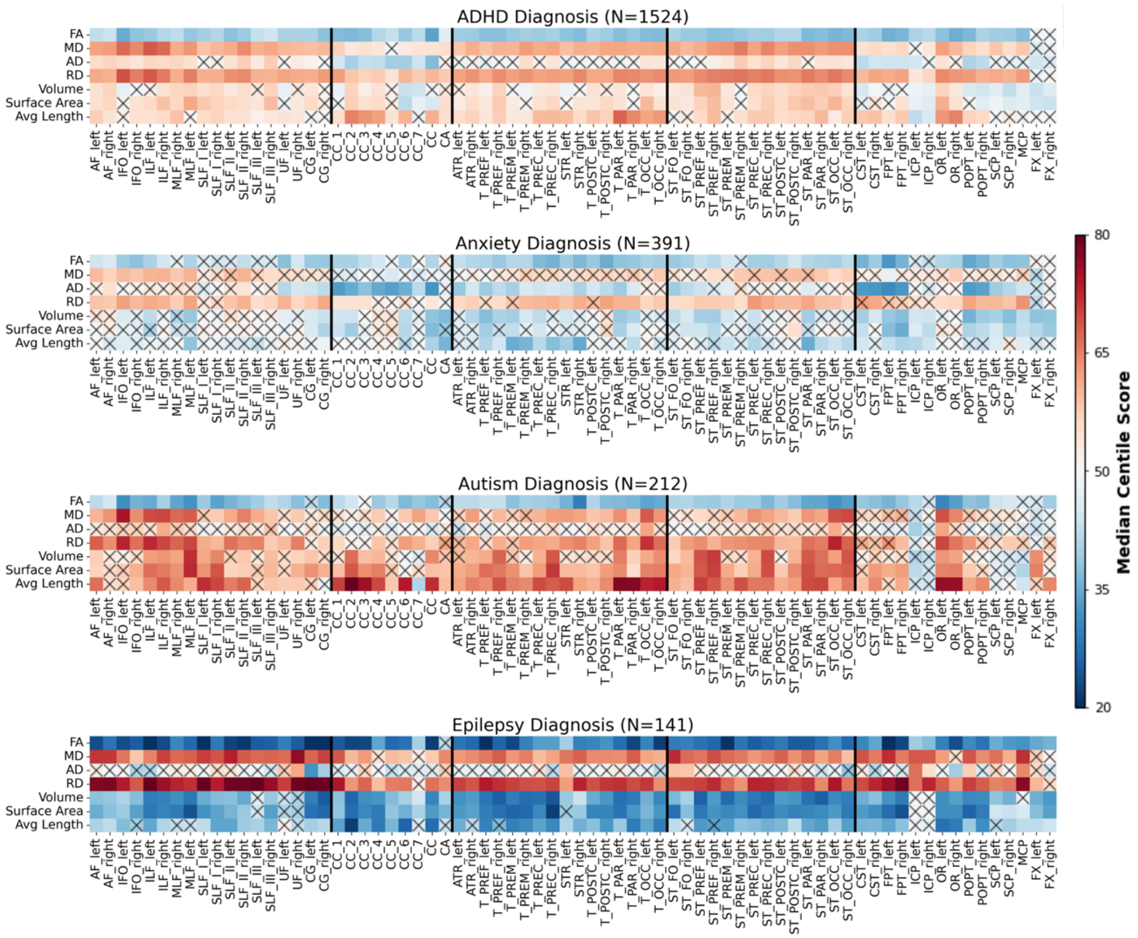

Supplemental Figure S3.F4. Centile score differences across other neurocognitive disorders with significant widespread deviations – FDR-BH correction. As an alternative analysis to Bonferroni correction, we also employ Benjamini-Hochberg false discovery rate (FDR-BH) correction when assessing significance (significance threshold of $\alpha = 0.05$) of centile score differences across tracts and features for many other clinical cohorts (top to bottom: attention-deficit hyperactivity disorder (ADHD), anxiety, autism spectrum disorder, and epilepsy) under a two-sided one-sample Wilcoxon signed-rank test. We observe either localized deviations in tract microstructure and macrostructure or consistent deviations across all tracts within specific macrostructural and microstructural features. Individuals with epilepsy and ADHD showed substantial deviations in FA and RD across the WM, suggesting diffuse microstructural abnormalities, alongside more localized reductions in volume, length, and surface area in select tracts. For individuals diagnosed with autism spectrum disorder, we observe increases in macrostructure across tracts, with a subset of tracts showing significant deviations. Finally, we observe sparsely significant deviations in microstructure and macrostructure for individuals diagnosed with anxiety. Gray "X" indicates features where the median centile was not significantly different from the 50th normative centile.

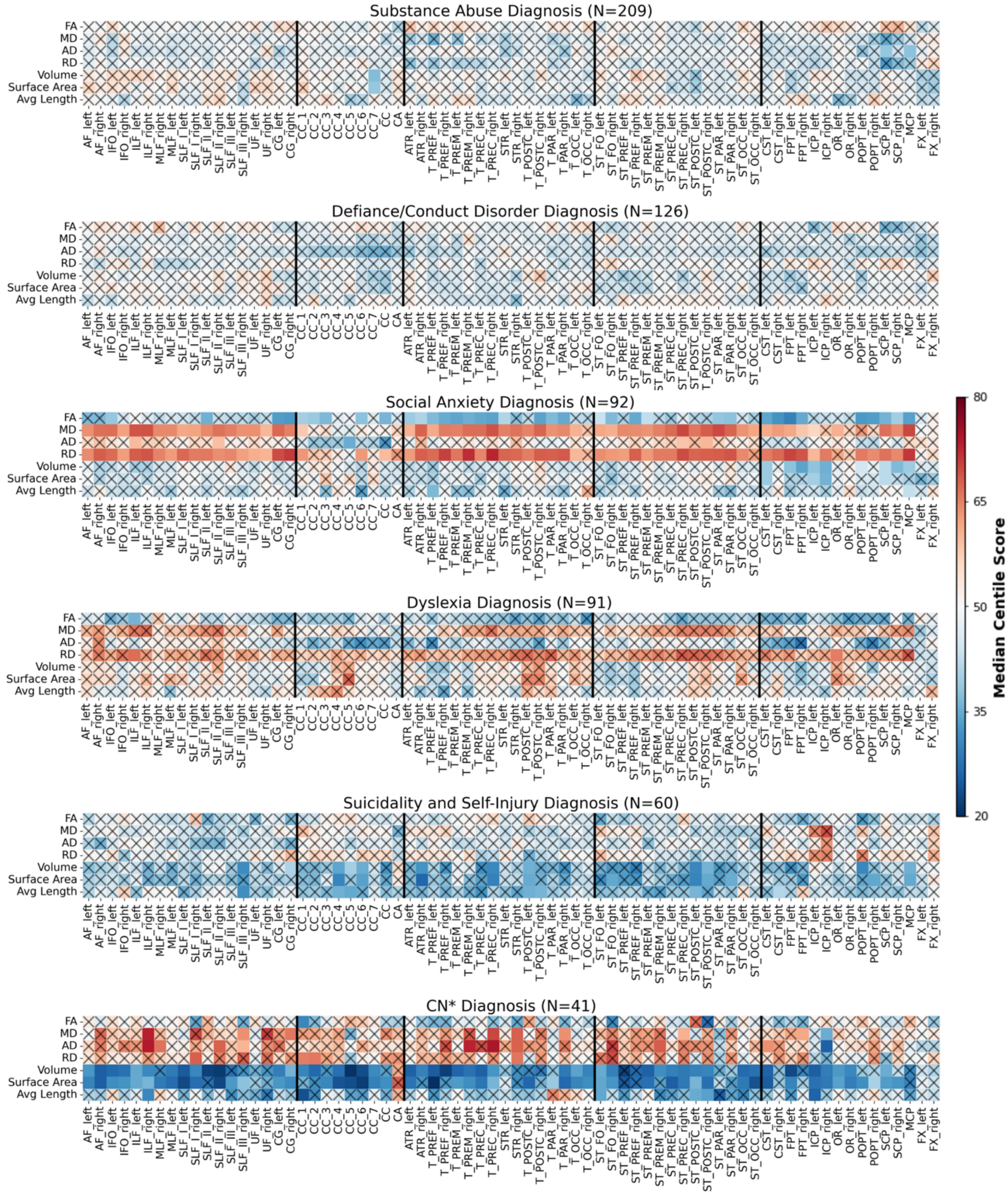

Supplemental Figure S3.F5. Centile score differences across other neurocognitive disorders with significant localized deviations – FDR-BH correction. As an alternative analysis to Bonferroni correction, we also employ Benjamini-Hochberg false discovery rate (FDR-BH) correction when assessing significance (significance threshold of $\alpha = 0.05$) of centile score differences across tracts and features for many other clinical cohorts (from top to bottom: substance abuse, defiance/conduct disorders, social anxiety disorder, dyslexia, suicidality and self-injury, and individuals who start as cognitively unimpaired and then transition to cognitive impairment, or CN*) under a two-sided one-sample Wilcoxon signed-rank test. We observe widespread microstructural deviations (MD, RD) for individuals with social anxiety disorder, and localized deviations in microstructure and macrostructure for CN* individuals. Notably, despite a smaller sample size (N<100), we observe significant local microstructural deviations in the OR_left (RD) and ILF_left (FA, RD) for individuals with dyslexia, which is consistent with prior literature [83–85]. Gray "X" indicates features where the median centile was not significantly different from the 50th normative centile.

ii. Effect Sizes of Centile Score Deviations

For all comparisons of clinical/non-typical cohorts, we use the two-sided one-sample Wilcoxon test, a non-parametric test, to assess significantly different centile distributions. Thus, we calculate effect size as the magnitude of the rank-biserial coefficient r_b:

$$r_b = 1 - \frac{4|W|}{N(N+1)} \quad (1)$$

where W is the two-sided Wilcoxon statistic and N is the sample size. For the cohorts with consistently significant features, we observe consistently larger effect sizes across tracts (**Supplemental Figure S3.F6, S3.F7**). Effect sizes for the other cohorts, which consist of mostly non-significant features, have much smaller and more sporadic effect sizes (**Supplemental Figure S3.F8**). Still, we observe moderate effect sizes in some features for some of these diagnostic groups. We also observe large effect sizes for the nCMD measurements (**Supplemental Table S3.T1**); however, we note that for cohorts with smaller sample sizes, the calculated effect sizes may be over or underinflated.

Supplemental Table S3.T1. Rank-biserial effect sizes for non-typical cohorts of the normalized centile mahalanobis distance (nCMD) metrics.

Cohort	nCMD Micro	nCMD Macro	nCMD Combined
Autism (N=212)	0.436	0.495	0.558
MCI (N=1509)	0.549	0.212	0.485
Alzheimer's Disease (N=535)	0.741	0.432	0.712
ADHD (N=1524)	0.057	0.303	0.162
Schizophrenia (N=397)	0.457	0.040	0.407
Depression (N=470)	0.031	0.059	0.103
Anxiety (N=391)	0.140	0.152	0.052

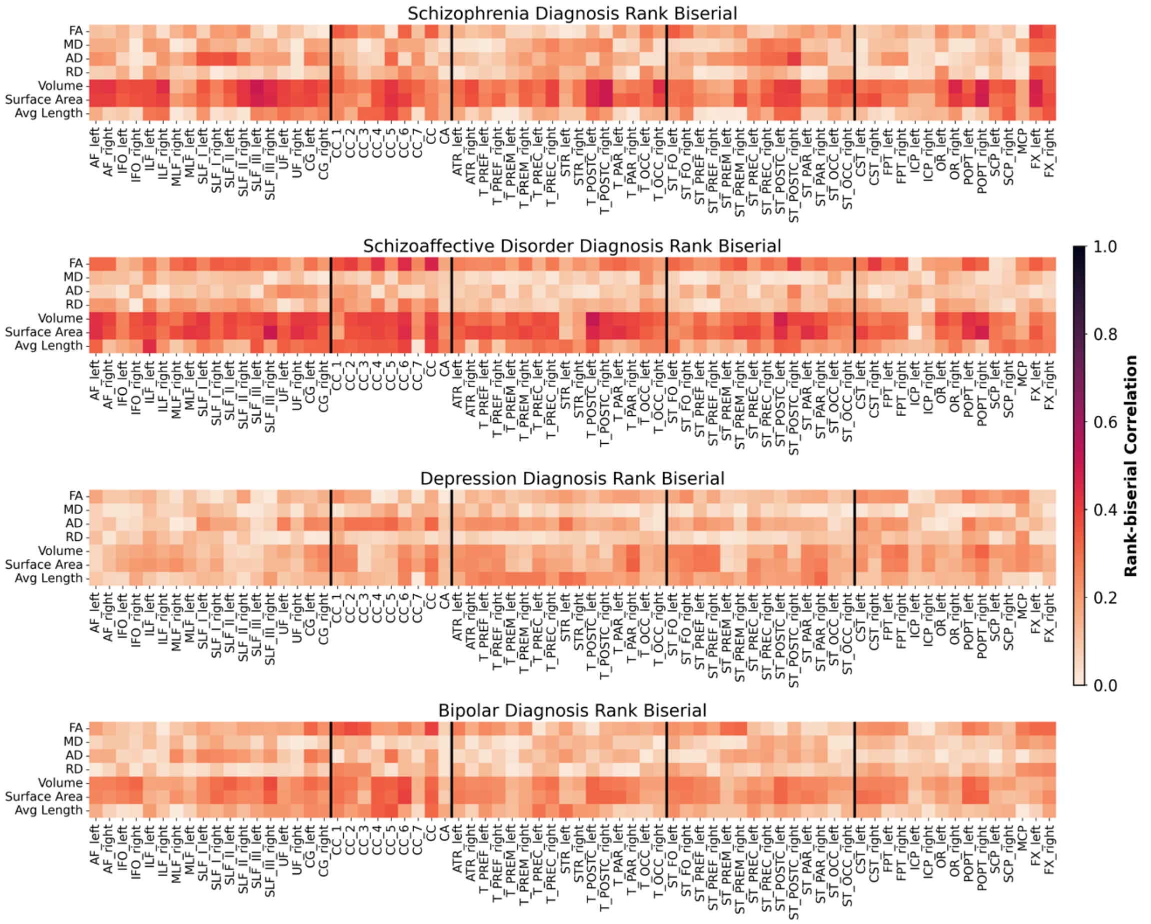

Supplemental Figure S3.F6. Effect size magnitudes of centile distances for major depressive and psychotic diagnostic groups. Rank-biserial correlations (magnitude) of major depressive and psychotic diagnostic group centile score differences from the median centile (from top to bottom, schizophrenia, schizoaffective disorder, depression, bipolar disorder). Larger effect sizes were observed for macrostructural measures when assessing schizophrenia, schizoaffective disorder, and bipolar disorder group deviations.

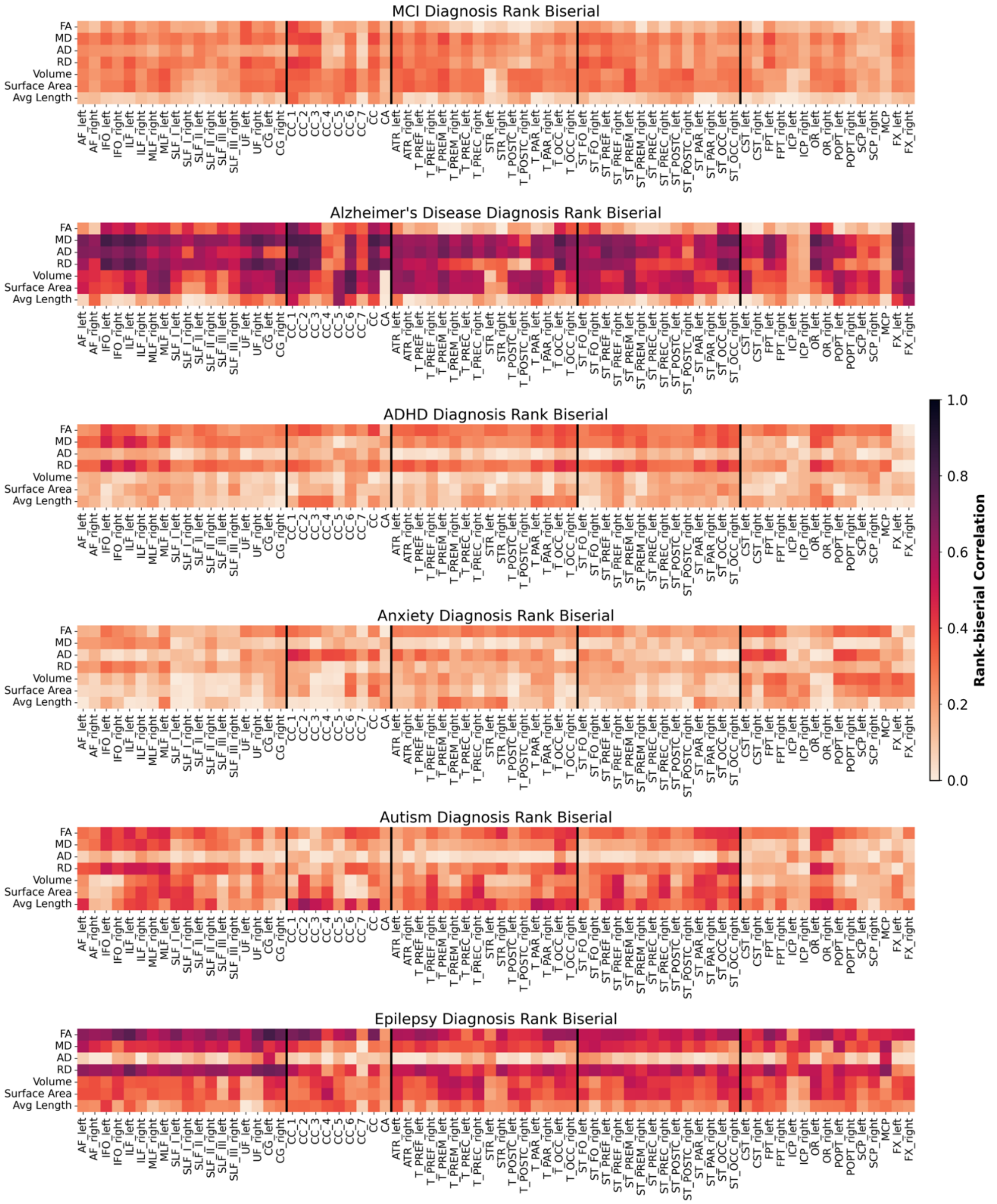

Supplemental Figure S3.F7. Effect size magnitudes of centile distances for other diagnostic groups with significant effects. Rank-biserial correlations (magnitude) of centile score differences from the median centile for other diagnostic groups with significant deviations (from top to bottom, mild cognitive impairment, Alzheimer's disease, ADHD, anxiety, autism spectrum disorder, epilepsy). Large effect sizes were observed across most features for Alzheimer's disease as well as FA, MD, RD for epilepsy. Moderate effect sizes were seen across features for the mild cognitive impairment diagnostic group and for several features of the ADHD, autism, and anxiety diagnostic groups.

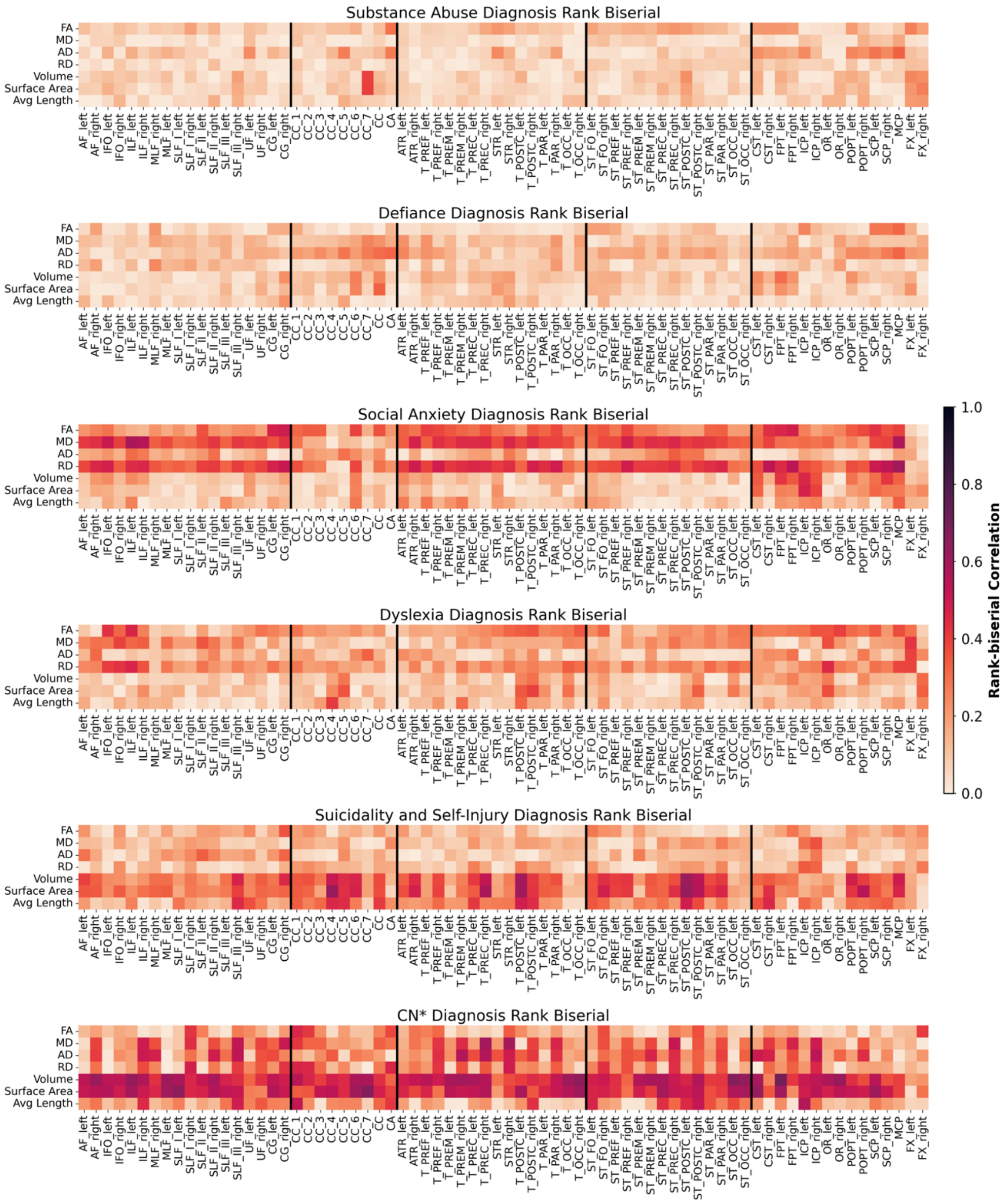

Supplemental Figure S3.F8. Effect size magnitudes of centile distances for other diagnostic groups with non-significant effects. Rank-biserial correlations (magnitude) of centile score differences from the median centile for other diagnostic groups with non-significant deviations (from top to bottom, substance abuse, defiance/conduct disorder, social anxiety, dyslexia, suicidality and self-injury, and individuals who start as cognitively unimpaired and then transition to cognitive impairment, or CN*). Although we did not observe significant centile score deviations for these diagnostic groups, the differences have moderate effect sizes for macrostructural features in the CN* and suicidality and self-injury diagnostic groups, as well as FA, MD, RD for social anxiety.

4. **Model Fitting**

i. Number of fractional polynomial terms for $\boldsymbol{\mu}$ and $\boldsymbol{\sigma}$

Upon normalization, the macrostructural features appear to have a slightly less complex dependency on age, as evidenced by the decrease in number of terms for best fit models of μ (**Supplemental Figures S4.F1, S4.F2**). This trend is more apparent for surface area than it is for volume or average length. We hypothesize this result may be due to a removal of variance introduced by the normalization of global cerebral tissue volume estimates, as people with larger heads will likely have larger WM tract volumes as well. However, this hypothesis was not formally tested in this work. For σ, however, there appears to be no large change in how non-linear the relationship with age is after normalization. Additionally, σ, which is related to the variance or spread of the distribution, appears to have a less complex age-related effect than the location of the distribution, or μ. This can be seen in the majority of best fit models having 2 or 3 fractional polynomial terms for μ, whereas most models have only 1 or 2 terms for σ.

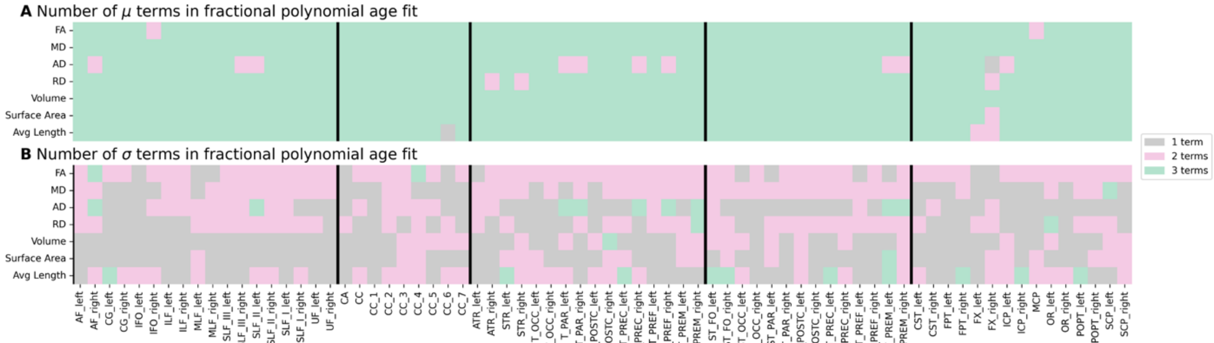

Supplemental Figure S4.F1. Fractional polynomial term numbers. The greater number of fractional polynomial terms for A.) μ, related to the location of the generalized gamma distribution, as opposed to B.) σ, which is related to the scaling of the distribution, suggests that the location of the distribution has a more non-linear relationship with age than the scaling.

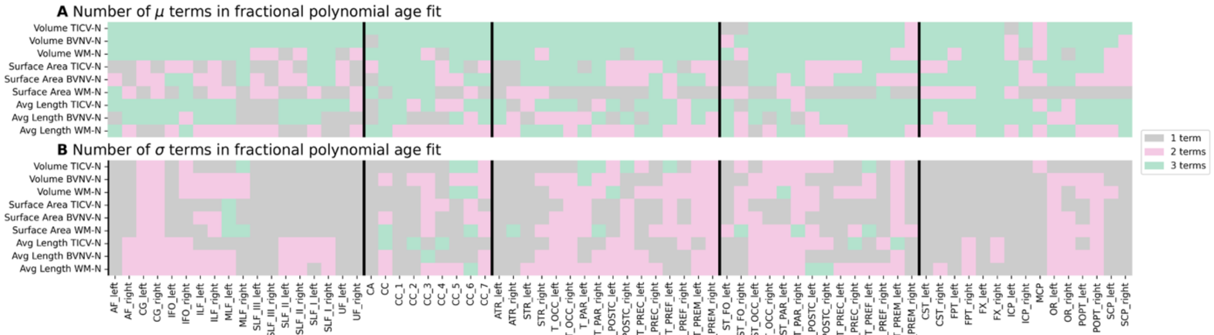

Supplemental Figure S4.F2. Fractional polynomial term numbers – normalized measures. Normalized macrostructural measures appear to have a less non-linear relationship with age than unnormalized measures, demonstrated by the shift from green boxes in **Supplemental Figure S4.F1** (above) to pink and gray boxes for A.) the μ term. However, normalization of macrostructural measures does not appear to change the non-linearity of B.) the σ term when compared to the unnormalized measures.

ii. Empirical Model Stability Analysis

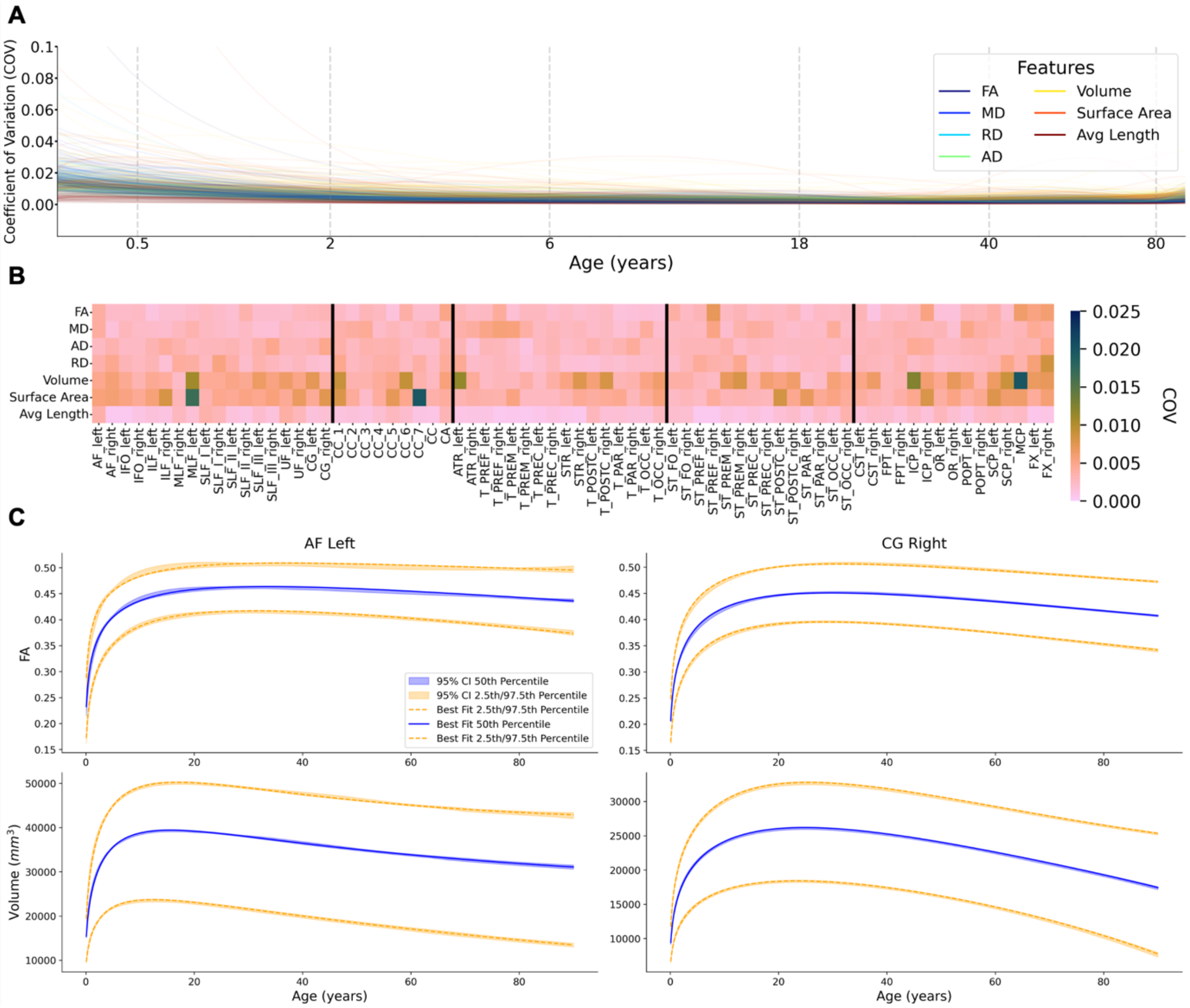

Supplemental Figure S4.F3. Model stability assessment using leave-one-study-out (LOSO) bootstrapping. We observe WM brain chart models to be stable across the entire lifespan. (A) Coefficient of variation (COV) from a LOSO bootstrapping analysis on the WM brain charts suggest a high stability across the majority of the lifespan for all models, with most models having a maximum of 1% COV at a given point in the lifespan. Age axis is log-scaled to highlight the spread of models at the beginning of the lifespan. (B) Averaging COV across all points in the lifespan demonstrate that most models have high stability as seen by low COV, with macrostructural models having a slightly larger COV on average. (C) Example plots of median (blue), and 2.5th and 97.5th (orange) percentiles with 95% confidence intervals derived from the LOSO bootstrapping show high stability across the entire lifespan and percentiles (top row is fractional anisotropy, bottom row is volume; left column is the left arcuate fasciculus (AF left), right column is the right cingulum (CG right)).

iii. Voxel Size Effect on FA

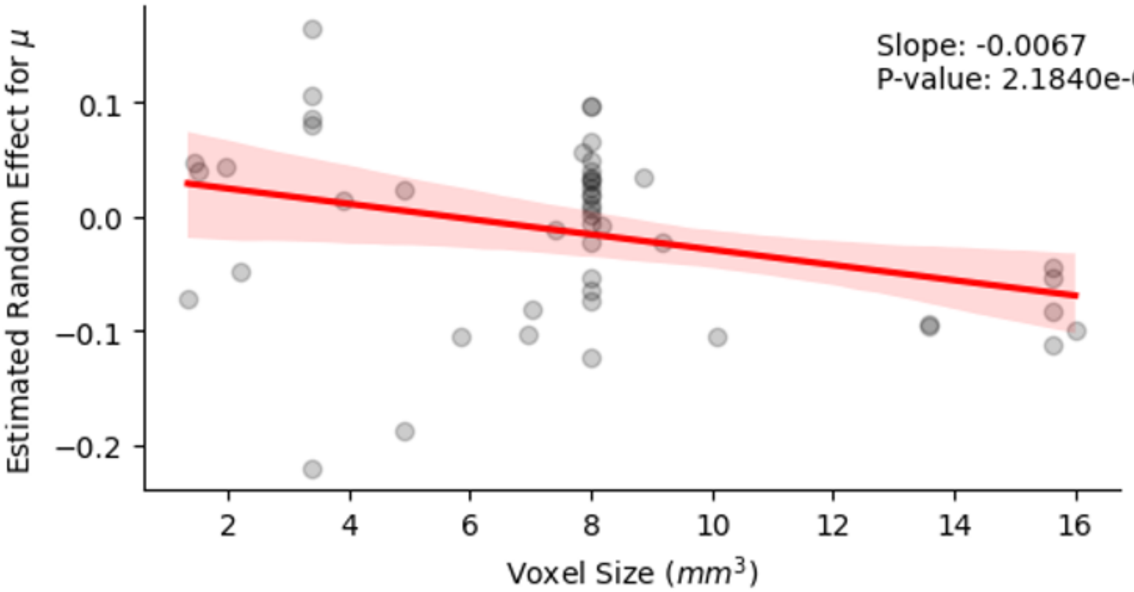

Supplemental Figure S4.F4. Voxel size correlation with dataset-estimated shift. We observe that for the whole-brain white matter FA, voxel size has a significant ($p < 0.05$) negative correlation with the estimated values for the GAMLSS random effect terms for μ. This indicates that the GAMLSS model is appropriately modeling acquisition-related variability across datasets for the brain charts. Note that in the GAMLSS formulation, μ is log-transformed, and thus the random effect terms are multiplicative instead of additive. For example, a random effect value of -0.1 corresponds to a multiplicative shift in the dataset values by $e^{-0.1} = 0.904$.

iv. Batch Correction and Site Harmonization

As described in the Methods section, we use "primary study" or "dataset" as the batch effect term to be estimated in GAMLSS. However, for many studies there are subsets of the data acquired with different acquisition parameters or physical scanners. Thus, we run a sensitivity analysis comparing the estimated centile scores of participants when using "dataset" as a batch variable to centile scores estimated using a combination of physical site, scanner, and acquisition as the batch variable (**Supplemental Figure S4.F5**). Across all tracts, the correlations between "dataset"-batch and "site-scanner-acquisition"-batch centile scores for participants were higher for macrostructural and lower for microstructural features. However, all correlations were greater than 0.88, with the majority being above 0.95. Within the microstructural features, AD centile scores showed the lowest correlation coefficients on average, while RD correlations were higher on average. Further, some tracts appeared to have higher correlation coefficients than others on average, with no distinct trends within tract groups.

While a nested covariance structure for site, scanner, and acquisition might be a more appropriate modeling of the batch effects, GAMLSS does not currently support specification of such a nested covariance structure. Consequently, in the model, any given "batch" for a subset of a dataset is inherently considered to be as different from any other subset as it is from any other

primary dataset. If such a nested covariance structure could be specified, the centile scores might be even more highly correlated between the "dataset"-batch and "site-scanner-acquisition"-batch models. While we have reduced variability introduced in data processing through containerization of all pipelines, these data processing considerations cannot account for variability introduced in any part of the raw data acquisition.

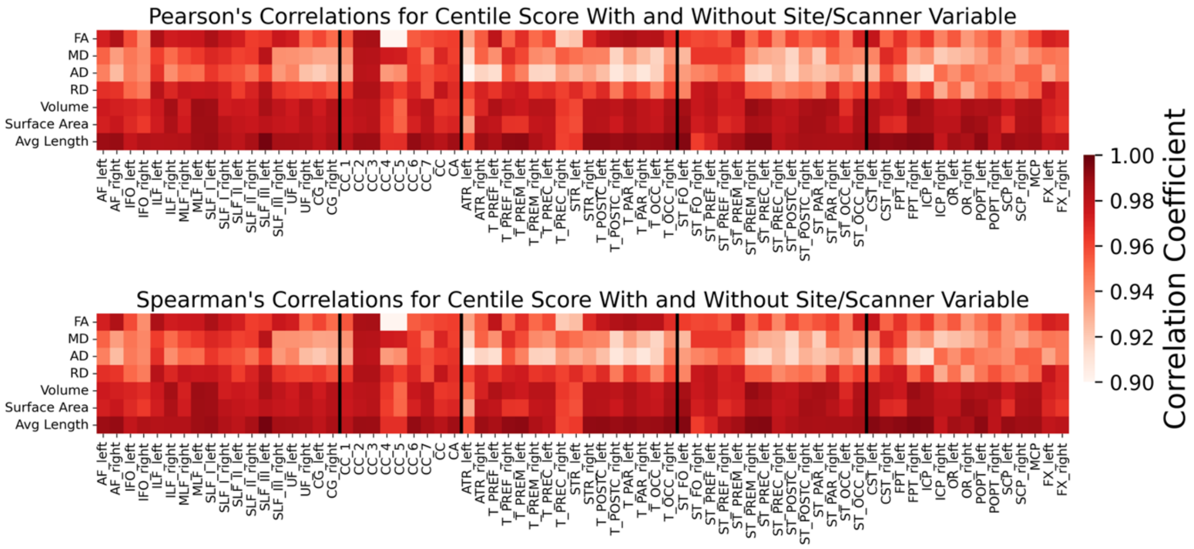

Supplemental Figure S4.F5. Correlation of centile scores for scanner-site-acquisition-batch vs dataset-batch models. When encoding site, dataset, and scanner as the random effect to be estimated in the GAMLSS model fitting, correlations of centile scores are high when compared to the model that uses only dataset as the random effect variable. Both (Top) Pearson's and (Bottom) Spearman's correlation coefficients of centile scores are above 0.88 for all models, with most models above 0.95. Macrostructural model correlations are noticeably higher than those for microstructural feature models. Additionally, AD models appear to have the lowest average correlation out of all feature models.

v. Sex-related Effects on White Matter Brain Charts

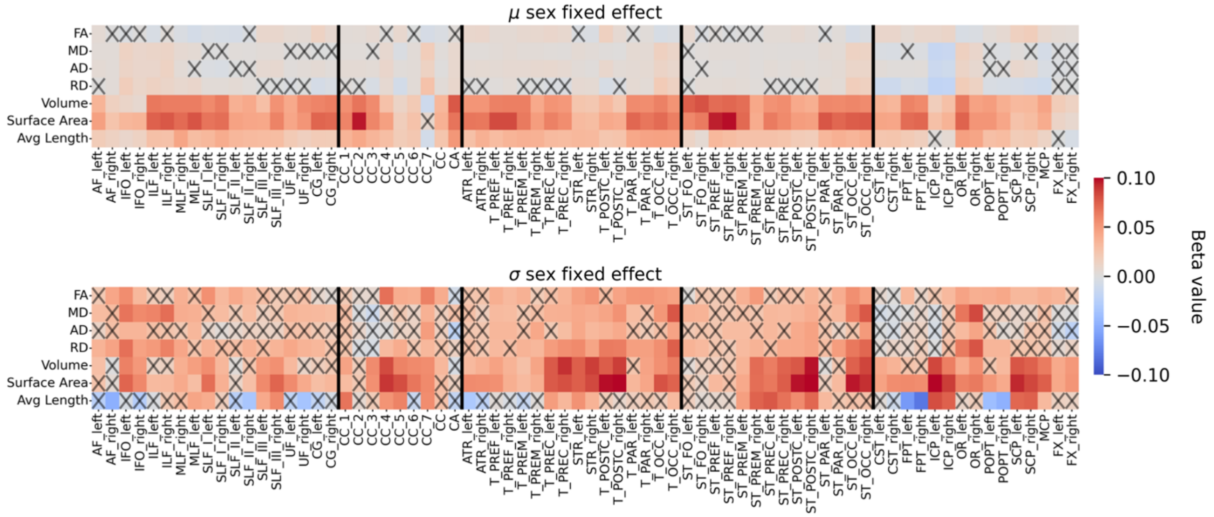

Supplemental Figure S4.F6. Significance of sex fixed effect across models. In the GAMLSS fitting, we find that the fixed effect of sex for μ (top) is significant for most models, whereas the sex fixed effect for σ (bottom) is much less significant (after Bonferroni correction for multiple comparisons, $p < 9.92 * 10^{-5}$). Positive (red) indicates that corresponding trajectory values for males are larger than those of females, whereas blue indicates the opposite. Macrostructural trajectories tend to have much larger values for μ than microstructural trajectories. All trajectories tend to have larger male variability, indicated by the positive (red) values for the σ fixed effect. Note that, due to the GAMLSS model specification, fixed effect values are logarithmically scaled and multiplicative in nature. Gray "X" indicates features where the sex effect is not significant.

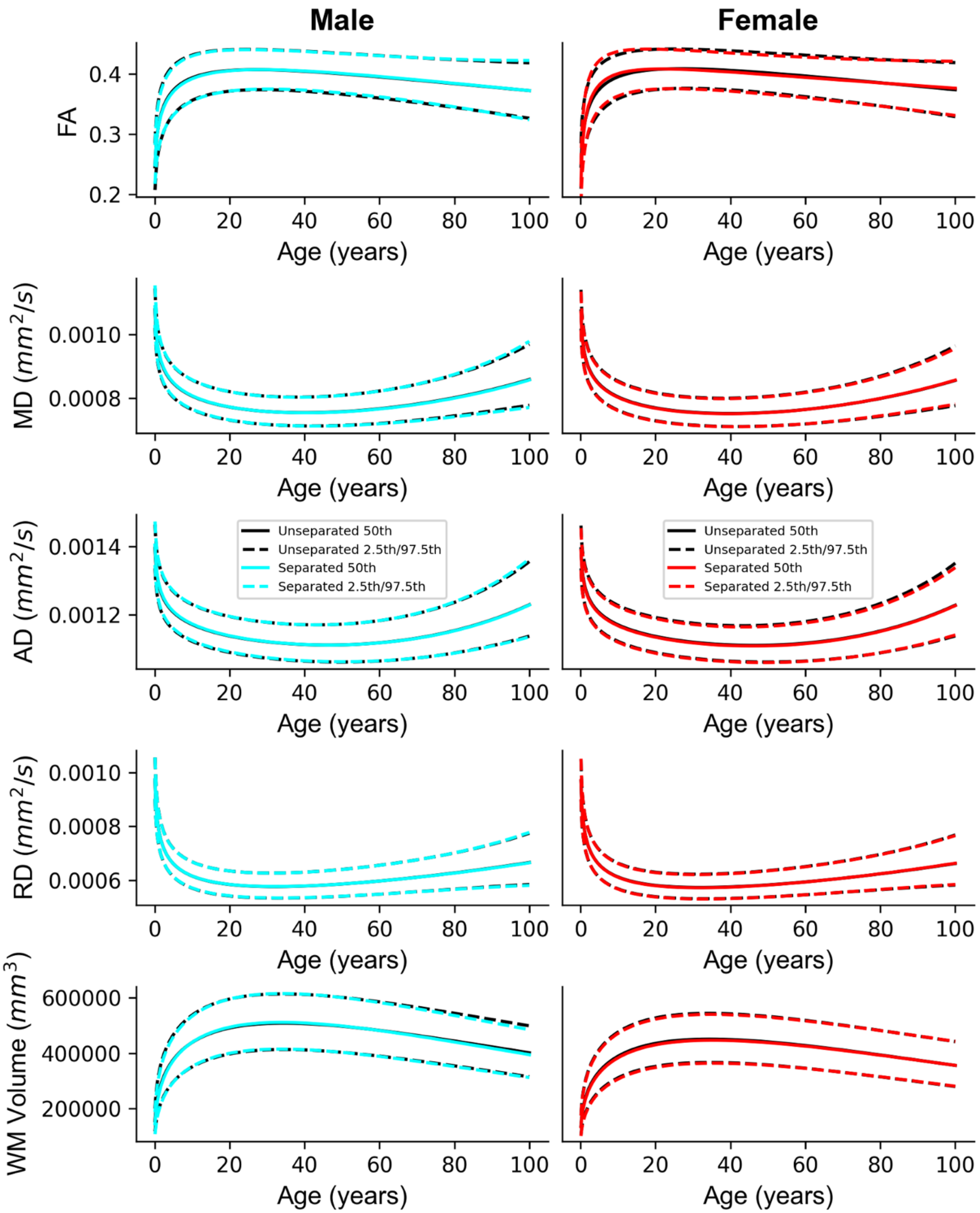

Supplemental Figure S4.F7. Comparison of sex-separated vs unseparated models. Normative models fit with sex as a linear effect (”Unseparated” - black lines) are very similar to models that were fit with male and female populations completely separated (“Separated” – cyan/red lines), indicating that the GAMLSS model fitting for the WM brain charts is appropriately capturing the sex-specific trajectories for microstructure and macrostructure (from top to bottom: global WM features of FA, MD, AD, RD, and volume). Male trajectories are plotted in the left and female trajectories are plotted on the right.

vi. Maxima and Minima of White Matter Tract Trajectories

Aggregated across all 72 tracts (**Supplemental Figure S4.F8**), we observed systematic variation in the timing of developmental milestones. Among microstructural metrics, axial diffusivity (AD) generally peaked later than other features, suggesting prolonged maturation of myelin-sensitive properties. Within several tract classes, we also observed an anterior-to-posterior patterning - where tracts located anteriorly reached peak maturation earlier than more posterior tracts, reflecting coordinated spatial gradients in WM development [46].

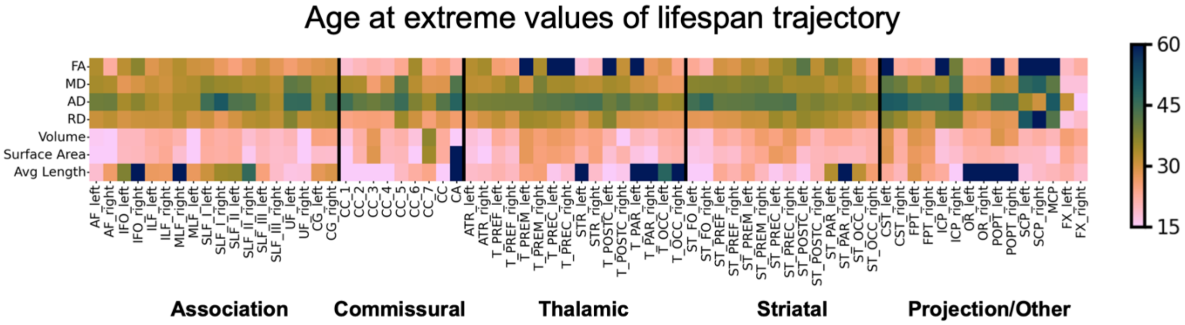

Supplemental Figure S4.F8. Age at extreme values of tract brain charts. A heatmap summarizes the estimated age at which each feature reaches its extreme value (peak or trough) across all 72 measured tracts, organized by tract class. This demonstrates systematic variations in milestone timing depending on the specific feature, pathway, and tract group. Across many tracts, macrostructural features tend to reach their milestones earlier in life compared to microstructural features.

vii. Use of Milestones to Examine Development and Aging

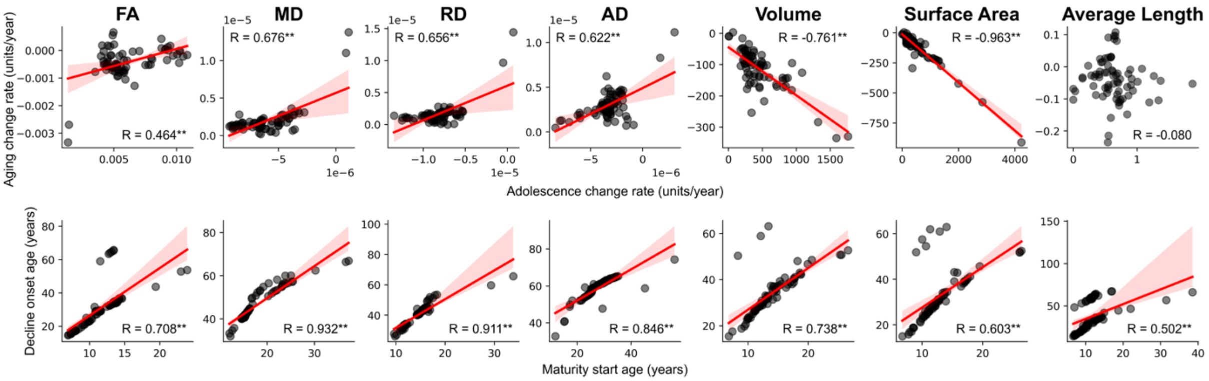

Supplemental Figure S4.F9. White matter charts can be used to test several theories that relate development to aging. These theories include: (1) hypotheses related to the rates of development versus rates of decline (top row), such as the classic gain-predicts-loss theory (i.e., regions which develop most rapidly will decline most rapidly), and the opposing slowest-peak-first-out theory (i.e., regions that develop at the slowest rate are most vulnerable to aging and deteriorate more rapidly); and (2) hypotheses related to developmental timing (bottom row), including the first-in-last-out theory (i.e., brain regions which develop first are the last to degenerate). Microstructural features (FA/MD/AD/RD) show statistically significant relationships between development and aging rates, providing support for the slowest-peak-first-out hypothesis (e.g., pathways with the slowest change in FA during development experienced the fastest decline in aging). Conversely, macrostructural features support the classic gain-predicts-loss hypothesis (e.g., pathways that develop at the fastest rate also decline at the fastest rate). However, our results on developmental timing (bottom row) do not confirm a first-in-last-out theory; rather, they suggest that tracts that reach maturity first are also those that begin to decline first. We note, however, that testing these timing models is complicated by the challenge of defining discrete developmental time points across continuous curves, as well as the ambiguity in the literature regarding whether 'first-in' refers to prenatal emergence versus lifespan maturation. Pearson correlation coefficients are reported for each association, with “**” indicating significance of the association under a least squares linear regression following Bonferroni correction ($p < 0.0035$). For significant associations, regression lines are plotted in red, with 95% confidence intervals shaded pink.

5. Comparison to Existing Reference Charts

The investigation of age-related changes in WM using neuroimaging data has been a well-established area of research for decades. Fewer, more recent works have been published on the use of neuroimaging data to construct growth charts for WM measurements[47,48], but have not been as extensive with regard to the number of features. Specifically, Bethlehem et al. only investigate global WM volume, whereas Zhu et al. investigate only microstructure through FA.

i. White Matter Lifespan Modeling in the Literature

Supplemental Table S5.T1. Past literature studying white matter lifespan trajectories.

Study	Age Range (years)	Participants	Datasets	Microstructure	Macrostructure	White Matter Info
Storsve et al. 2016 [49]	23-87	201 (402 sessions)	1	DTI	N/A	TRACULA (18 tracts)
Giorgio et al. 2010 [50]	23-81	66	1	DTI	N/A	WM Voxels
Beck et al. 2021 [51]	18-94	573 (702 sessions)	2	DTI, NODDI, DKI, RSI, WMTI, SMT mc: 6 models, 20 scalars	N/A	TBSS skeleton
Henriques et al. 2023 [52]	18-88	636	1	DTI, DKI, NODDI	N/A	JHU Atlas (48 ROIs)
Toschi et al., 2020 [53]	13-62	91	1	DTI, CHARMED	N/A	TBSS skeleton + JHU overlap (50 ROIs)
Lebel et al. 2012 [54]	5-83	403	1	DTI	WM Volume	12 pathways (Manual)
Slater et al. 2019 [55]	7-84	801	1	DTI, NODDI, g-ratio	N/A	20 bilateral + callosal tracts (AFQ)
Yeatman et al. 2014 [56]	7-85	102	1	DTI (FA,MD only)	N/A	24 pathways (AFQ)
Groves et al. 2012 [57]	8-85	484	2	DTI (FA,MD,MO)	N/A	TBSS skeleton + ICA
Hoagey et al. 2019 [58]	20-94	186	1	DTI (FA,MD)	N/A	Population-based WM Voxels
Kochunov et al. 2011 [59]	11-90	1031	1	DTI (FA only)	N/A	TBSS skeleton + JHU overlap (11 ROIs)

Bender et al. 2015 [60]	19-78	96 (192 sessions)	1 (2 timepoints)	DTI (FA,AD,RD)	N/A	TBSS skeleton + JHU overlap (13 ROIs)
Sexton et al. 2014 [61]	20-84	203 (406 sessions)	1 (2 timepoints)	DTI	N/A	TBSS skeleton + manual ROIs (4 ROIs)
Sala et al. 2012 [62]	13-70	84	1	DTI	Tract volume	8 ROIs from FA atlas
Westlye et al. 2010 [63]	8-85	430	2	DTI	WM Volume	TBSS skeleton + JHU overlap (16 ROIs)
Billiet et al. 2015 [64]	17-70	59	1	DTI, NODDI, DKI, MET2	N/A	Voxel-wise in WM mask; also 17 JHU ROIs
Molloy et al. 2021 [65]	18-75	79	1	DTI	N/A	TBSS skeleton + JHU overlap (20 ROIs)
Ardekani et al. 2007 [66]	26-69	20	1	DTI	N/A	6 Manually Traced ROIs
Schilling et al., 2023 [67]	0-100	2789	4	DTI,NODDI	Tract volumes, lengths, areas, etc.	63 TractSeg ROIs
Conte et al. 2024 [68]	2wk-100yrs	6186	12	FA,MD	WM Volume; Tract Density	XTRACT (29 tracts)

ii. Comparison to Bethlehem et al.

As our method for assessing global WM macrostructure follows Bethlehem et al.[48] in the use of FreeSurfer and GAMLSS, we can more directly compare our brain charts to theirs (**Supplemental Figure S5.F1**). Notably, our curves follow the same trend of a rapid increase during infancy that plateaus during early adulthood, then finally dropping off during aging. We believe this agreement in capturing the general trend indicates that our brain charts are comparable to other established normative models for neuroimaging.

However, we note that there are some slight differences in the two sets of global WM volume curves. While the age at peak is not very representative, this value has tended to vary in the literature quite a bit as well. Specifically, our observed peak for WM volume is slightly less that Lebel et al. (peak at 37 years) [14], about 15 years later than Courchesne et al. (beginning of the 4th decade) [15] and 5 years later than Bethlehem et al. [13], and 10 years earlier than Giorgio et al.[16]. Furthermore, the Bethlehem et al. curves were fit using pre-natal data and used multiple different methods to obtain WM volume measurements, specifically, different FreeSurfer versions and hand drawn ROIs (**Supplemental Table S5.T1**). In contrast, our curves contained only post-natal data and stayed consistent with our data processing approach. Finally, Bethlehem et al. included multiple scans per participant, which was not explicitly modeled in their

GAMLSS formula, whereas we only include one scan per participant. These factors, in addition to the largely increased sample size used by Bethlehem et al., could be contributing to the slight differences between the curves.

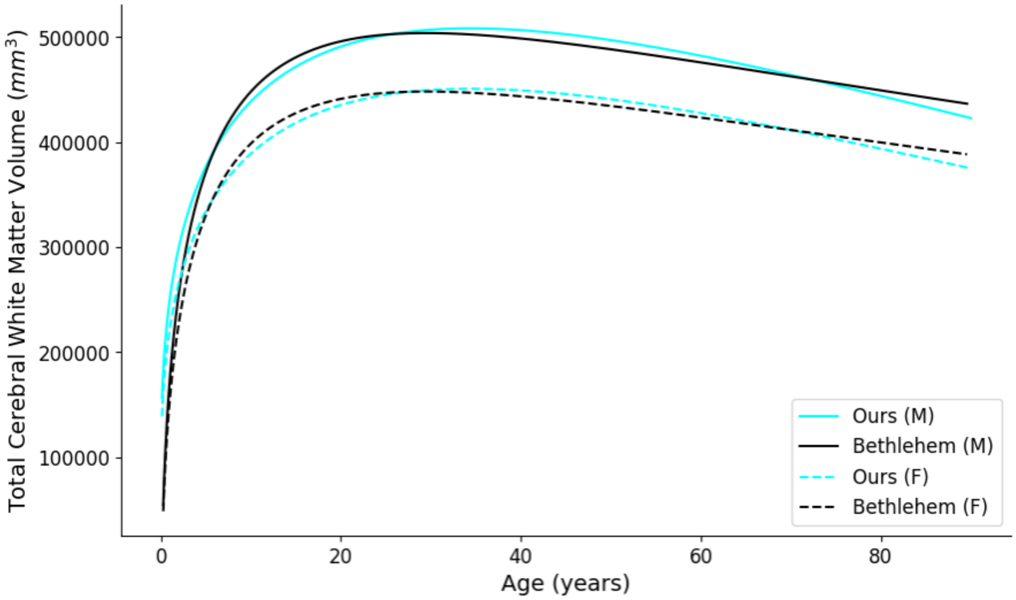

Supplemental Figure S5.F1. Comparison of our global white matter volume curve to Bethlehem et al. Our brain charts follow the same trend as Bethlehem et al., which is a rapid increase during infancy that plateaus during early adulthood and finally drops off during aging. We believe this agreement in capturing the general trend indicates that our brain charts are comparable to other established normative models for neuroimaging.

Supplemental Table S5.T2. Comparison of methods for our work to Bethlehem et al. (2022)

Study	***Participants***	***Repeated Scans***	***Normative Modeling***	***Processing Variability***	***Distribution Family***	***Out-of-sample Centile Scoring***	***Public release***
**Bethlehem**	101,457	Yes	GAMLSS; fractional polynomials for age	Yes; modeled in GAMLSS	Generalized Gamma	Yes, MLE	Website*
**Our Work**	35,120	No	GAMLSS; fractional polynomials for age	No	Generalized Gamma	Yes, MLE	Zodeno&

*https://brainchart.shinyapps.io/brainchart/; &https://zenodo.org/records/15367425

iii. Comparison to Zhu et al.

The FA brain charts created by Zhu et al. use the preprocessing pipeline defined by the ENIGMA consortium[69] to obtain quantitative values of microstructure within regions of interest (ROIs) defined by the JHU DTI atlas (**Supplemental Table S5.T2**). While these atlas-based approaches are commonly used methods for quantifying microstructure within specific brain regions, we note that such practices are not subject specific. Furthermore, these methods do not permit assignment of a single voxel to multiple different tracts, resulting in ROIs that do not completely represent complete white matter pathways (**Supplemental Figure S5.F3**). In our approach, even though TractSeg predefines 72 white matter tracts, the approach is tractography-based, allowing us to study subject-specific white matter pathways derived directly from an individuals' dMRI data. This is an especially important aspect of our methodology, as white matter pathways are known to overlap in space[70,71], and tractography-based methods allow assignment of a single voxel to multiple different tracts. In this regard, we are the first to use WM tracts/pathways for constructing WM brain charts, as we are not using the atlas-based methods used in other research papers.

Supplemental Table S5.T3. Comparison of our WM brain charts to existing microstructural brain charts in the literature.

Study	Age Range	Datasets	Sample Size for Brain Charts	Total Sample Size (including validation)	Preprocessing	Normative modeling	Features {total number}
Zhu et al. 2025 [47]	3-95	10	13,297	40,898	EPI- and eddy-current distortion correction; TBSS with JHU labels[69]	GAM + ComBat variations	FA {25}
Villalón-Reina et al. (preprint) [72]	4-91	19	54,583	54,583	EPI- and eddy-current distortion correction; TBSS with JHU labels[69]	HBR	FA, MD, AD, RD {88}
Our work	0-100	50	35,120	41,897	DWI Preprocessing (PreQual); DTI Fitting (MRtrix); Tractography (TractSeg); Whole-brain White Matter Segmentation (FreeSurfer)	GAMLSS	FA, MD, AD, RD, Volume, Surface Area, Average Length, Normalized Macrostructure {1157}

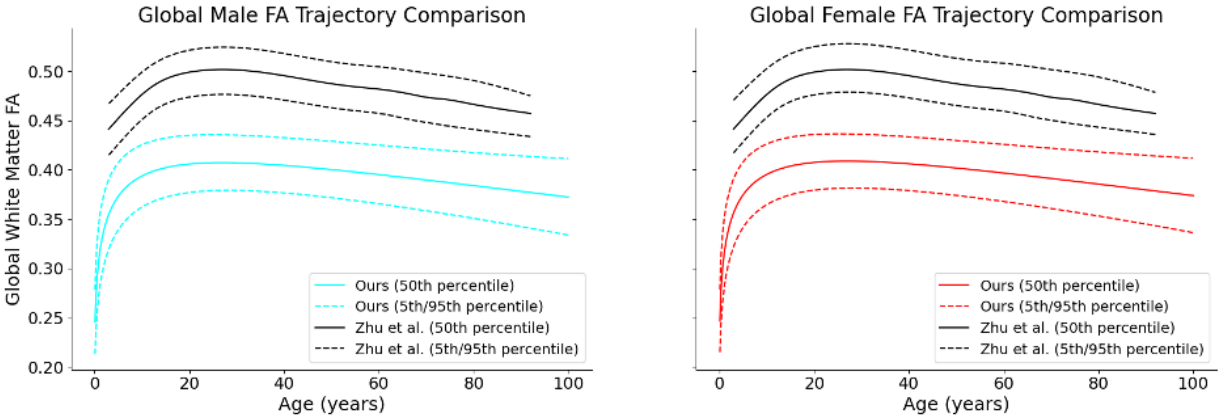

Supplemental Figure S5.F2. Comparison of lifespan global FA trajectory to Zhu et al. Our trajectory follows the same general trend as Zhu et al., with a sharp increase in adolescence, followed by a plateau in early adulthood, and then a decline in aging. Noticeably, the trajectories from Zhu et al. sit at higher FA values, due to the use of phenotype extraction along only the TBSS-defined white matter skeleton: Compared to our method, which contains information from both central and peripheral WM regions, the TBSS method places a larger focus on central WM, and specifically the central white matter skeleton defined by the highest FA within the sheet-like (or cylinder-like) white matter pathways. (**Supplemental Figure S5.F4**).

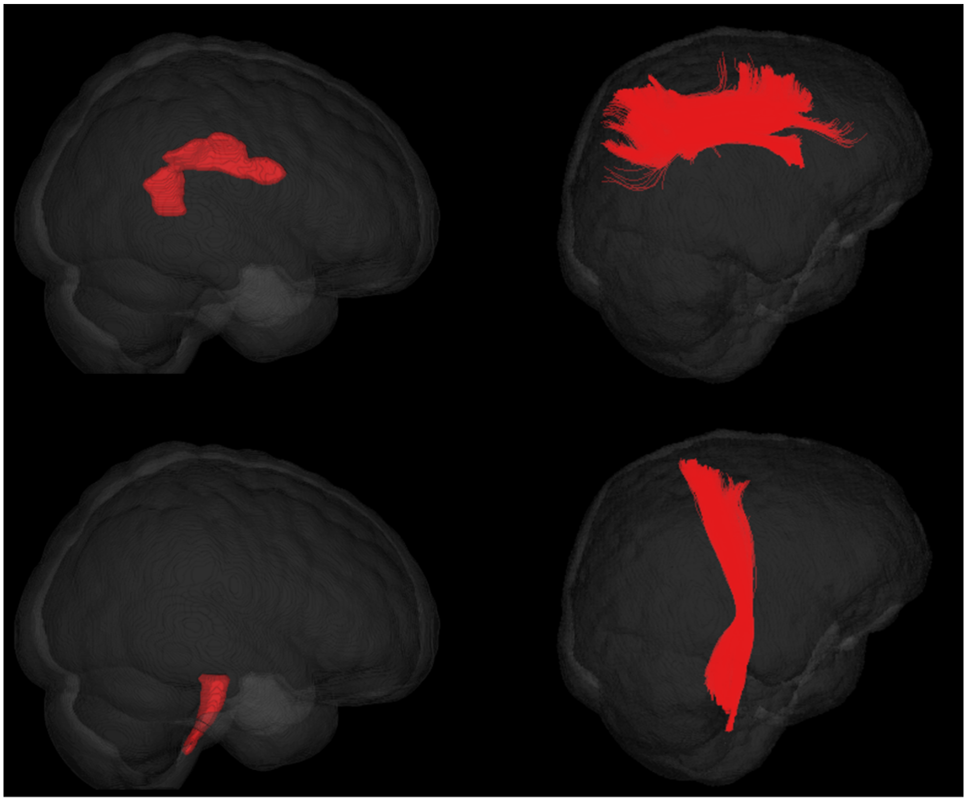

Supplemental Figure S5.F3. Comparison to Zhu et al. for tract specific white matter coverage. Zhu et al.'s method for tract-specific white matter coverage is based on the JHU atlas labels (left), whereas our method is based on subject-specific tractography using TractSeg (right). Notably, atlas-based approaches result in fundamentally spatially separated regions that do not constitute full pathways. Visualized are the left superior longitudinal fascicle (top) and the left corticospinal tract (bottom).

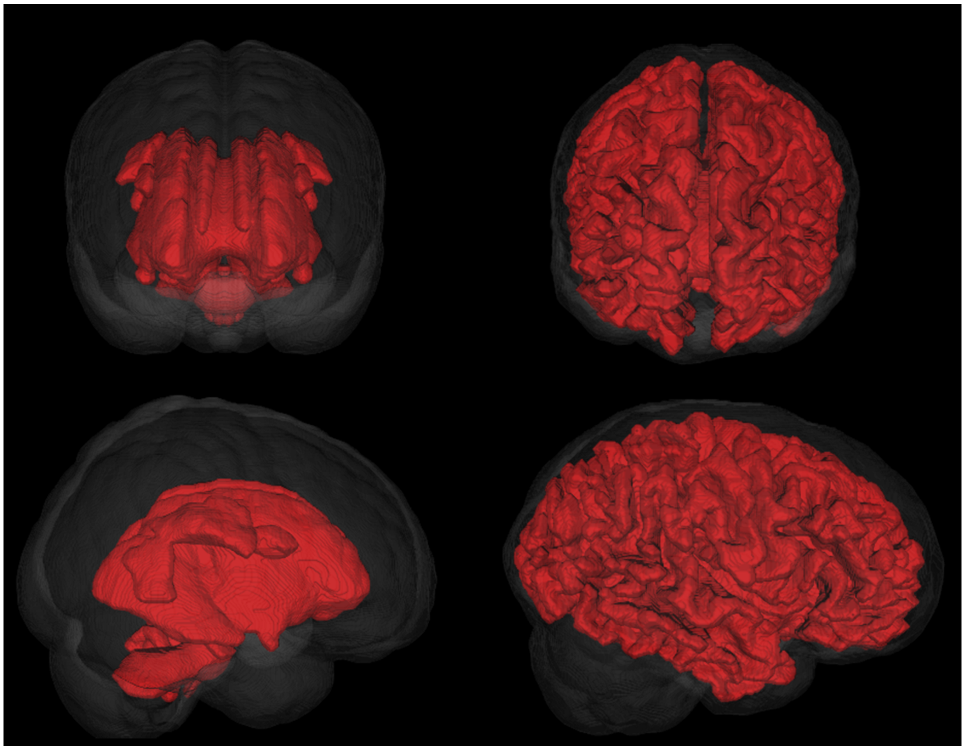

Supplemental Figure S5.F4. Comparison to Zhu et al. white matter coverage for global microstructure. Zhu et al.'s method utilizes the combined JHU atlas labels (left) to define white matter coverage for assessing global microstructure. In contrast, our method uses the general white matter mask derived from FreeSurfer (right). The FreeSurfer mask provides broader overall white matter coverage compared to the JHU atlas, which encompasses only specific white matter tracts. Furthermore, the FreeSurfer-based segmentation captures greater subject-specific anatomical variation by not relying on the boundaries of an atlas generated from a small cohort of young adults. The JHU labels are visualized with respect to the corresponding atlas, whereas the FreeSurfer labels are visualized from a particular individual from our database.

6. **Data Quality**

As mentioned in the main manuscript, we conducted a thorough quality control process that involved visualizing each scan and performing quantitative outlier removal (see Methods: Data Selection). The final results of this process can be found in **Extended Data Table ED.T1**. Notably, infant datasets tend to have many more scans that are not included in the final participant pool for creating the brain charts. We attribute this effect largely to TractSeg, which was trained on an adult population. As infant data are more out-of-distribution compared to other datasets that the TractSeg models were not trained on, many infant scans showed poor tract reconstruction.

Furthermore, we conducted an analysis to assess both image quality across datasets and how image quality affects downstream centile score estimates. Specifically, we visualized distributions for the PreQual image quality metrics (IQMs) of mean relative displacement, the chi-squared error metric in shells used for diffusion tensor fitting, contrast-to-noise ratio (CNR) as calculated by EDDY for the main shell used for tensor fitting for scans included in creating the brain charts (**Supplemental Figure S6.F1**). We do not observe IQMs to be influenced by age at time of scan, as distributions are not consistently higher or lower at any given age range. In other words, IQM distribution appeared to be dataset-dependent rather than age-dependent.

For assessing correlation of estimated centile scores with IQMs, we computed Spearman's correlation coefficients between centile scores and each IQM (**Supplemental Figure S6.F2**). All correlations were either low or non-existent, with nearly all correlations is below 0.15 and none above 0.19. These results suggest that image quality of the dMRI data does not largely affect the estimated centile scores.

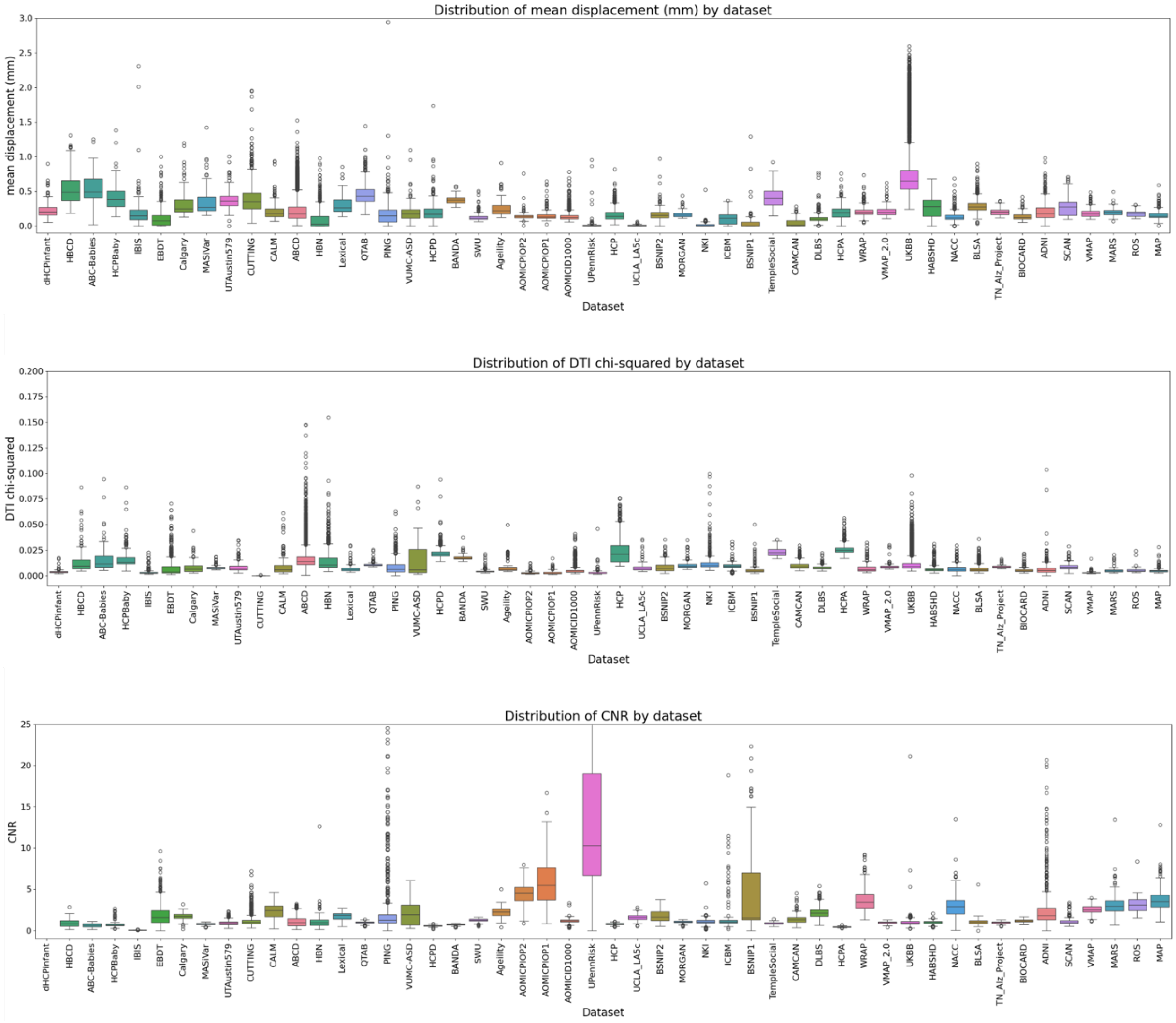

Supplemental Figure S6.F1. Distribution of PreQual image quality metrics by dataset. We do not observe age at time of scans to largely influence image quality metrics (IQMs) for dMRI preprocessing, as IQM distributions are not consistently higher or lower at any age range. Plotted are (top) motion as mean relative displacement, (middle) chi-squared error metric in the shells used for the diffusion tensor imaging fit, and (bottom) contrast to noise ratio (CNR) as calculated by eddy for the main shell used for the tensor fitting. Datasets are ordered by mean age of participants, with the youngest ages on the left and oldest on the right. Distributions are visualized as boxplots, with a solid black line representing the median, box widths representing the interquartile ranges (IQRs), whiskers representing the 1.5*IQR range, and datapoints past whiskers plotted individually.

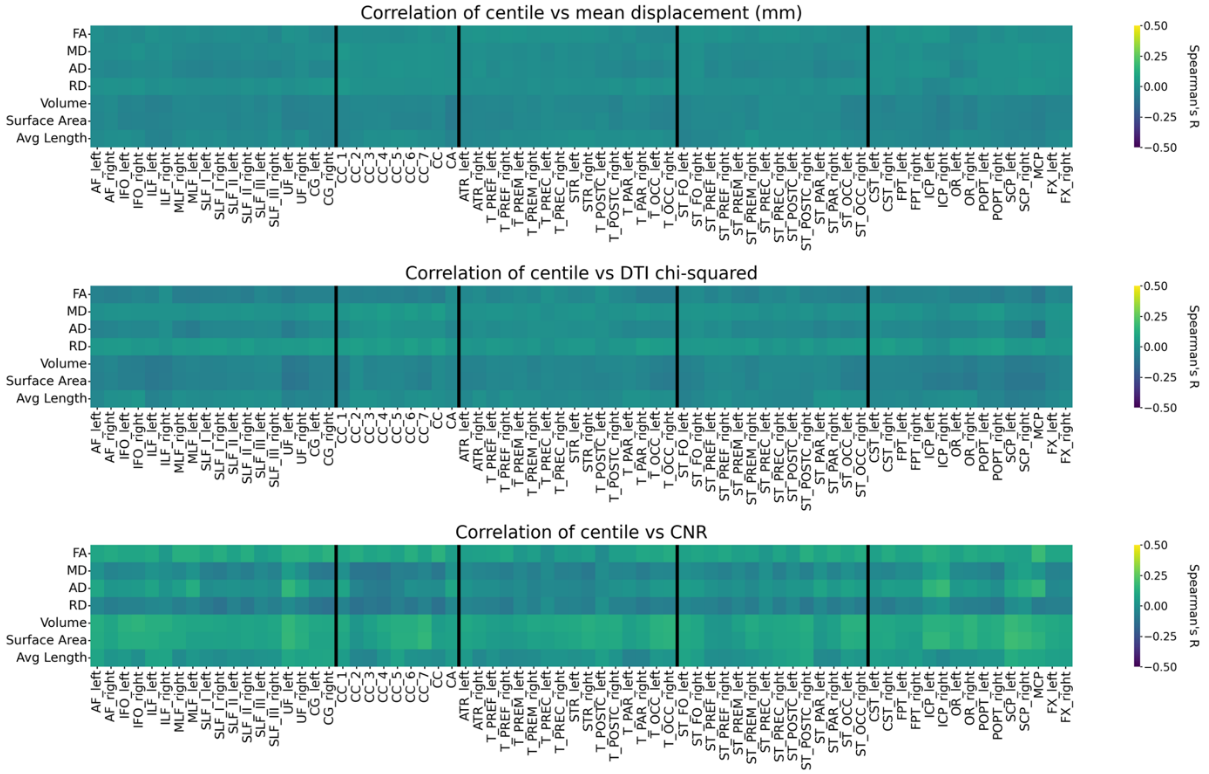

Supplemental Figure S6.F2. Correlation of PreQual image quality metrics with centile scores. We do not observe image quality metrics (IQMs) for dMRI preprocessing to be correlated with observed centile scores, as the absolute value of nearly all correlations is below 0.15, with none above 0.19. Plotted are Spearman's correlation coefficients with (top) motion as mean relative displacement, (middle) chi-squared error metric in the shells used for the diffusion tensor imaging fit, and (bottom) contrast to noise ratio (CNR) as calculated by eddy for the main shell used for the tensor fitting.

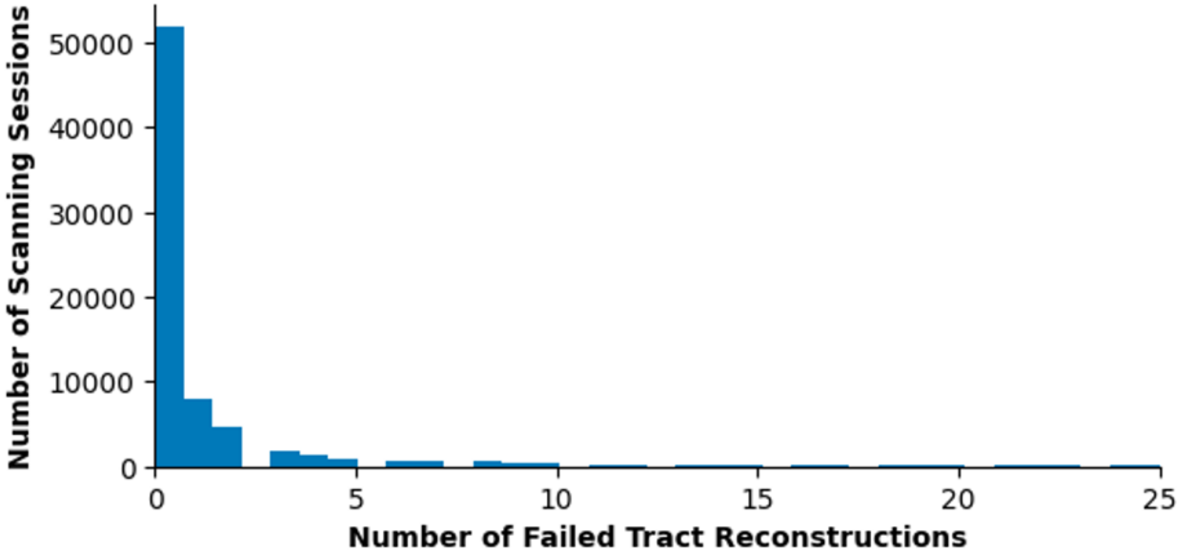

Supplemental Figure S6.F3. Tract reconstruction failures per session. While the majority of scanning sessions reconstructed most of the 72 TractSeg tracts, there were several scanning sessions that failed tract reconstruction for a large portion of tracts.

7. Anomaly Detection

To assess the utility of our brain charts for performing predictive analyses on clinical cohorts, we performed a classification task to distinguish participants with Alzheimer's disease and mild cognitive impairment (MCI) from typically developing and aging individuals. For the classification, we used centile scores for the microstructural features of FA, MD, AD, and RD and for the macrostructural features of volume, surface area, and average length for all 72 tracts (504 total centile score features). A multilayer perceptron (MLP) with two hidden layers was trained on the 504-dimensional input, using ReLU activation between layers, class-weighted Cross Entropy as a loss function, and the Adam optimizer with a learning rate of 0.001. The network was trained with 5-fold cross validation, with classes evenly split across folds. For individuals whose scans did not reconstruct all 72 tracts in the processing pipeline, the *miceforest* algorithm was used for data imputation (https://github.com/AnotherSamWilson/miceforest). We report area under the receiver operating characteristic curve (ROC-AUC) and balanced accuracy in **Supplemental Tables S7.T1 and S7.T2**. The 0.910 +/- 0.910 and 0.829 +/- 0.015 average AUCs across folds indicate promising discriminative performance; however, we still advocate that the utility of these brain charts should be as research tools rather than clinical diagnostic tools.

Supplemental Table S7.T1. Results of Alzheimer's disease classification experiment.

Fold	Balanced Accuracy	ROC-AUC
**Fold 0**	0.810	0.888
**Fold 1**	0.807	0.901
**Fold 2**	0.820	0.907
**Fold 3**	0.866	0.937
**Fold 4**	0.789	0.916
**Average**	**0.819 +/- 0.029**	**0.910 +/- 0.018**

Supplemental Table S7.T2. Results of mild cognitive impairment classification experiment.

Fold	Balanced Accuracy	ROC-AUC
**Fold 0**	0.746	0.851
**Fold 1**	0.721	0.818
**Fold 2**	0.721	0.812
**Fold 3**	0.737	0.833
**Fold 4**	0.712	0.832
**Average**	**0.728 +/- 0.014**	**0.829 +/- 0.015**

8. **Out-of-Sample Alignment**

i. Stability of Out-of-Sample Alignment

To assess the stability of the out-of-sample (OOS) alignment process, we performed OOS alignment for the HCPA dataset at varying sample sizes. At each sample size (from 700 to 50 in increments of 50), we randomly selected participants from the dataset without replacement, with 100 different bootstrapped assessments at each sample size. We did this for both FA and volume of the right cingulum and isthmus of the corpus callosum. Across all features, we observed that the OOS alignment process is more stable when estimating the μ random effect term compared to the σ random effect term (**Supplemental Figure S8.F1**). Although stability of estimates vary across the tracts, features, and parameters, we observe a conservative threshold for stability in OOS alignment at N=100. This threshold is similar to that from Bethlehem et al.[48], suggesting that our method is in agreement with existing normative brain charts.

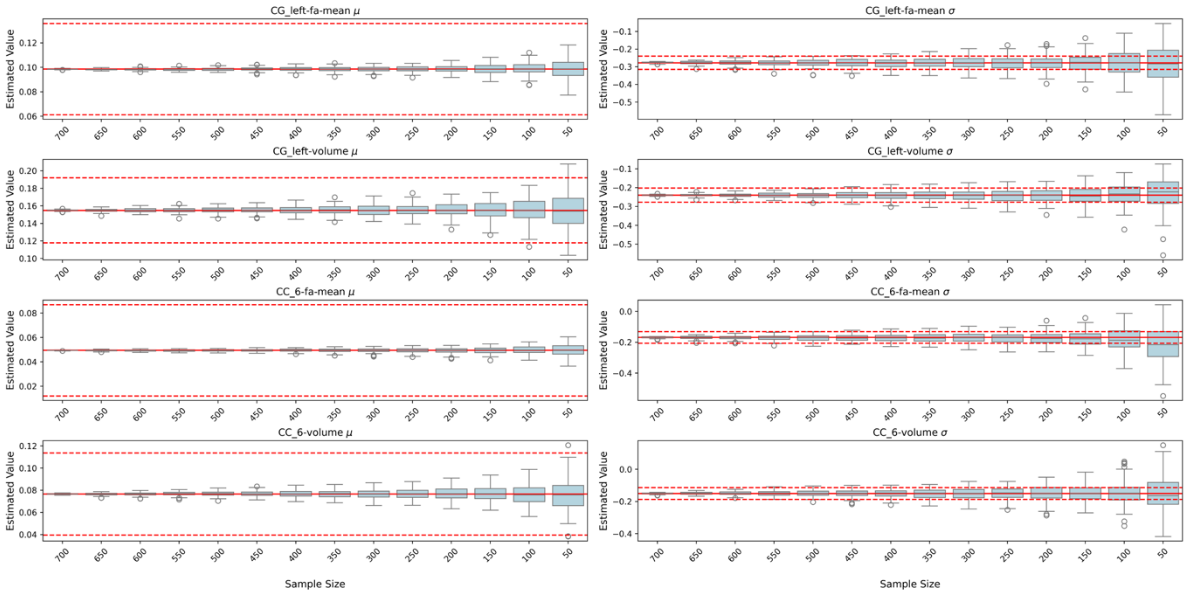

Supplemental Figure S8.F1. Out-of-sample alignment bootstrapping experiment for determining minimum sample size. Out-of-sample (OOS) alignment appears stable and within the GAMLSS-estimated standard error (solid red lines indicate the GAMLSS-estimated value, dashed red lines indicate error bars) for sample sizes greater than or equal to 100. The estimated random effect term for μ (left) appears more stable for OOS alignment than it is for σ (right), as the distributions for bootstrapped random effects fall within the GAMLSS error range at smaller sample sizes for μ. We observe varying levels of stability for different features and tracts (from top to bottom: FA of right cingulum, volume of right cingulum, FA of isthmus of corpus callosum, volume of isthmus of corpus callosum), with N=100 as a conservative threshold. Distributions of bootstrapped estimates are visualized as boxplots, with a solid black line representing the median, box widths representing the interquartile ranges (IQRs), whiskers representing the 1.5*IQR range, and datapoints past whiskers plotted individually.

We note that, as part of our postprocessing pipeline, constrained spherical deconvolution (CSD) is run to extract peak directions for downstream tractography. Although not evaluated in this manuscript, it is generally accepted that CSD requires roughly 30 directions for accurate estimation; however, CSD can also be performed with fewer directions, albeit with less stable estimates.

ii. Alignment with Other Preprocessing Pipelines

The DWI preprocessing pipeline that we use, PreQual, is generically state-of-the-art, as it contains all the fundamental steps that are considered "essential" (corrections for susceptibility-distortions, motion, eddy-current-distortions)[73]. These fundamental steps are present in most dMRI preprocessing workflows. Although we used PreQual to preprocess our DWI data, we acknowledge that alternative tools for DWI preprocessing exist, such as DTIPrep/Dmriprep[74,75] DSI-Studio[76], ExploreDTI[77], and TORTOISE[78]. We also include additional steps that are considered beneficial for data quality (denoising; slice-wise outlier imputation). Thus, researchers can generically use different preprocessing pipelines.

To assess whether or not data corrected with different DWI preprocessing pipelines are align-able to our brain charts, in an extreme case, we performed out-of-sample alignment using data from the AOMICPIOP2 dataset[4] that had not had any DWI preprocessing. We then compared the original centile scores for each participant to those obtained via the raw data alignment. Most features are highly correlated (**Supplemental Figure S8.F2**), suggesting that even in this extreme case, data are able to be aligned to our brain charts without having run data through the PreQual preprocessing pipeline. However, we do note that there are a few features, notably smaller tracts like the fornix (FX) or anterior commissure (CA), that are not as highly correlated. Regarding specific tract groups, we observed that cerebellar tracts (MCP, ICP, SCP) and projection tracts (FPT, CST) also exhibited lower correlations than other tract groups, likely due to their increased sensitivity to distortion artifacts in these brain regions.[79] We did not observe any features to be more or less correlated across tracts when compared to other features.

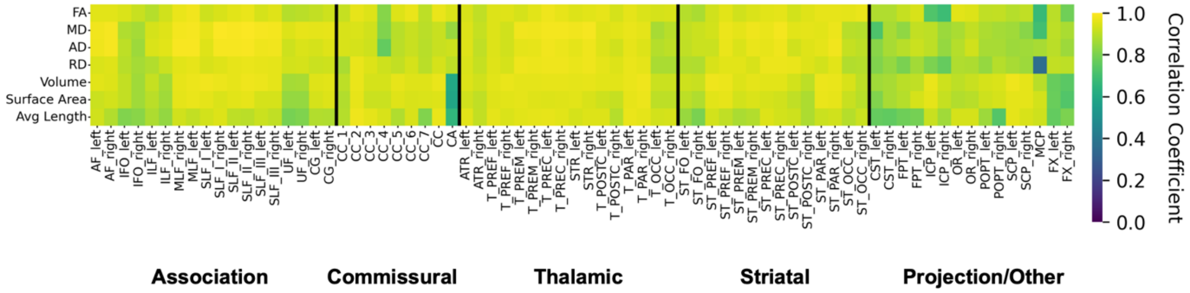

Supplemental Figure S8.F2. Pearson correlation of centile scores for the AOMICPIOP2 dataset with and without dMRI preprocessing. Centile scores from both preprocessed and raw data are highly correlated across most tracts and features; this extreme case indicates that DWI data that have undergone different preprocessing workflows are can be appropriately aligned to our brain charts. Measurements from smaller tracts (FX, CA) and cerebellar/projection tracts are most affected by lack of DWI preprocessing, showing reduced correlations when compared to other tracts.

iii. How to Perform Out-of-Sample Data Alignment

One of the most important aspects of brain charts is the ability to score new data within the normative trajectories to determine how abnormal quantitative brain metrics are. We have demonstrated how we perform alignment of new datasets to the centile curves (see Methods: Maximum Likelihood Estimation for Out-of-Sample Datasets). For any researchers who would like to use these brain charts, we release a Docker container as an easily reproducible method of performing this alignment with new data and provide the following tutorial:

1.) Before doing anything, Docker needs to be installed. Official instructions can be found here: (https://docs.docker.com/get-started/get-docker/). [80] Docker must be running properly before proceeding.

2.) Download the Docker image (as a .tar file) from Zenodo at this link: https://zenodo.org/records/15367425

   Save the .tar file somewhere it can be easily found later, such as a Downloads folder.

3.) Open a terminal (command line tool). Load the Docker image, using the following command-line prompt:

```
docker load -i </path/to/docker/.tar/file>
```

   The Docker image can also be loaded through the Docker Desktop GUI.

4.) Confirm that the Docker image is loaded by running:

```
docker images
```

5.) Before alignment, the data must be properly formatted in a CSV file that can be read by the Docker image. The CSV is required to have columns age, sex, and $diagnosis$, where $age \geq 0$ is a numerical value, sex is a binary variable where "male" is encoded as 1 and "female" is encoded as 0, and $diagnosis$ is a categorical variable. Typically developing/aging (also referred to as "cognitively normal") participants are encoded as "CN" for $diagnosis$, and to perform alignment there must be rows in the CSV file that contain "CN" as the $diagnosis$. For better alignment, ensure as many "CN" participants as possible, and note that having a small number of participants may result in poorly aligned data and thus poorly estimated centile scores. There must also be at least one quantitative variable column in the CSV file, where quantitative variables are named as:

```
<tract>-<measure>
```

   `<tract>` must be one of the TractSeg defined tract names found in **Extended Data Table ED.T2**, whereas `<measure>` must be one of `{fa-mean, md-`

`mean, ad-mean, rd-mean, volume, surface_area, avg_length}.`
Thus, the CSV should follow formatting such as:

age	sex	diagnosis	AF_left-fa-mean	AF_right-md-mean	…
75.1	0	CN	0.453	0.00110	…
45	1	CN	0.562	0.00140	…
62.5	1	AD	0.398	0.00098	…
…	…	…	…	…	…

Note that in cases where rows have empty entries, centile scores will not be calculated for these metrics and as a result will have a missing entry in the respective centile score output. Further, only rows labeled with a "CN" $diagnosis$ and with non-missing centile values will be used for estimating the random effect terms for a particular measure.

6.) Run the Docker using the following command:

```
docker run --rm -v </path/to/OOS.csv>:/INPUTS/input.csv \
-v </path/to/output/directory>:/OUTPUTS \
r_lifespan_env \
python3 /WMLifespan/scripts/perform_OOS_alignment.py \
/INPUTS/input.csv /OUTPUTS/aligned.csv
```

where `aligned.csv` is the destination file you wish to save the aligned centile score values.

As detailed in the Methods section, these normative curves are cross-sectional in nature. Thus, researchers performing out-of-sample alignment should only include cross-sectional data in the CSV file, or one scan per participant. Should researchers wish to evaluate longitudinal data with the cross-sectional models, the flag can be used to also save the estimated random effect terms for the dataset. We also note that this alignment to the normative models assumes that the data in the CSV file come from the same primary dataset. Calculation of centile scores for multiple datasets need to be done in separate Docker commands, each with their own distinct input CSV file.

iv. How to obtain centile trajectories of features

For researchers who wish to examine the normative trajectories of features more closely, we also provide a method for obtaining centiles of trajectories in a CSV format:

1.) To begin, follow steps 1.) through 4.) of the section "How to Perform Out-of-Sample Data Alignment" in order to properly set up the Docker image.

2.) To obtain centile curves for a given tract and metric, run the following command:

```
docker run --rm \
-v </path/to/output/directory>:/OUTPUTS \
r_lifespan_env \
python3 /WMLifespan/scripts/output_centile_curves.py \
<tract> <measure> /OUTPUTS/centiles.csv
```

where centiles.csv is the destination file you wish to save discrete values of the normative trajectory for the given <tract> and <measure>. Note that <tract> must be one of the TractSeg defined tract names found in **Extended Data Table ED.T2**, whereas <measure> must be one of {fa-mean, md-mean, ad-mean, rd-mean, volume, surface_area, avg_length}.

9. Considerations for Cross-sectional vs. Longitudinal Brain Charts

Previous work has shown that cross-sectional normative models can be inaccurate in predicting measured longitudinal trajectories [81], as longitudinal models can better separate true developmental change from generational differences that exist in the data and cross-sectional data may be unrepresentative of individuals. Although many of our datasets contained longitudinal data, there were several challenges we encountered and aspects about the normative models we needed to consider for longitudinal brain charts [82]. First, despite the subset of datasets with participant data at multiple time points, for the majority of participants in the study we only have cross-sectional data available. Incorporating longitudinal data would require estimation of random effects for each participant, introducing significant computational complexity in the model. We found that we did not have enough data points to stably estimate individual-level random effects. Furthermore, having to estimate these individual-level random effects would greatly decrease the stability and increase complexity of the out-of-sample centile scoring process, as alignment with N participants would require estimation of $2N$ terms instead of only 2 terms with the cross-sectional models. These constraints and considerations led us to develop a robust cross-sectional approach that maintains generalizability and computational feasibility. We acknowledge the potential benefits of longitudinal modeling and suggest this as an important avenue for future research.

10. References

[1] Casey BJ, Cannonier T, Conley MI, Cohen AO, Barch DM, Heitzeg MM, et al. The Adolescent Brain Cognitive Development (ABCD) study: Imaging acquisition across 21 sites. Dev Cogn Neurosci 2018;32:43–54. https://doi.org/10.1016/J.DCN.2018.03.001.

[2] Weber CJ, Carrillo MC, Jagust W, Jack CR, Shaw LM, Trojanowski JQ, et al. The Worldwide Alzheimer's Disease Neuroimaging Initiative: ADNI-3 updates and global perspectives. Alzheimer's & Dementia: Translational Research & Clinical Interventions 2021;7:e12226. https://doi.org/10.1002/TRC2.12226.

[3] Karayanidis F, Keuken MC, Wong A, Rennie JL, de Hollander G, Cooper PS, et al. The Age-ility Project (Phase 1): Structural and functional imaging and electrophysiological data repository. Neuroimage 2016;124:1137–42. https://doi.org/10.1016/J.NEUROIMAGE.2015.04.047.

[4] Snoek L, van der Miesen MM, Beemsterboer T, van der Leij A, Eigenhuis A, Steven Scholte H. The Amsterdam Open MRI Collection, a set of multimodal MRI datasets for individual difference analyses. Sci Data 2021;8:85. https://doi.org/10.1038/s41597-021-00870-6.

[5] Siless V, Hubbard NA, Jones R, Wang J, Lo N, Bauer CCC, et al. Image acquisition and quality assurance in the Boston Adolescent Neuroimaging of Depression and Anxiety study. Neuroimage Clin 2020;26:102242. https://doi.org/10.1016/J.NICL.2020.102242.

[6] Miller MI, Younes L, Ratnanather JT, Brown T, Trinh H, Lee DS, et al. Amygdalar atrophy in symptomatic Alzheimer's disease based on diffeomorphometry: the BIOCARD cohort. Neurobiol Aging 2015;36:S3–10. https://doi.org/10.1016/J.NEUROBIOLAGING.2014.06.032.

[7] Ferrucci L. The Baltimore Longitudinal Study of Aging (BLSA): A 50-Year-Long Journey and Plans for the Future. The Journals of Gerontology: Series A 2008;63:1416–9. https://doi.org/10.1093/GERONA/63.12.1416.

[8] Kristian Hill S, Reilly JL, Keefe RSE, Gold JM, Bishop JR, Gershon ES, et al. Neuropsychological impairments in schizophrenia and psychotic bipolar disorder: findings from the Bipolar-Schizophrenia Network on Intermediate Phenotypes (B-SNIP) study. Am J Psychiatry 2013;170:1275–84. https://doi.org/10.1176/APPI.AJP.2013.12101298.

[9] Parker DA, Trotti RL, McDowell JE, Keedy SK, Keshavan MS, Pearlson GD, et al. Differentiating biomarker features and familial characteristics of B-SNIP psychosis Biotypes. Translational Psychiatry 2025 15:1 2025;15:1–10. https://doi.org/10.1038/s41398-025-03501-5.

[10] Reynolds J, Long X, Paniukov D, Bagshawe M, Dewey D, Lebel C. Calgary Preschool MRI Dataset 2019. https://doi.org/10.17605/OSF.IO/AXZ5R.

[11] Holmes J, Bryant A, Gathercole SE. Protocol for a transdiagnostic study of children with problems of attention, learning and memory (CALM). BMC Pediatrics 2019 19:1 2019;19:1–11. https://doi.org/10.1186/S12887-018-1385-3.

[12] Shafto MA, Tyler LK, Dixon M, Taylor JR, Rowe JB, Cusack R, et al. The Cambridge Centre for Ageing and Neuroscience (Cam-CAN) study protocol: a cross-sectional, lifespan, multidisciplinary examination of healthy cognitive ageing. BMC Neurol 2014;14. https://doi.org/10.1186/S12883-014-0204-1.

[13] Edwards AD, Rueckert D, Smith SM, Abo Seada S, Alansary A, Almalbis J, et al. The Developing Human Connectome Project Neonatal Data Release. Front Neurosci 2022;16:886772. https://doi.org/10.3389/FNINS.2022.886772/BIBTEX.

[14] Park DC, Hennessee JP, Smith ET, Chan MY, Chen X, Dakanali M, et al. The Dallas Lifespan Brain Study: A Comprehensive Adult Lifespan Data Set of Brain and Cognitive Aging. Scientific Data 2025 12:1 2025;12:1–14. https://doi.org/10.1038/s41597-025-04847-7.

[15] Chen Y, Zhu H, Shen D, An H, Gilmore J, Lin W. Mapping Growth Patterns and Genetic Influences on Early Brain Development in Twins. Med Image Comput Comput Assist Interv 2009;12:232. https://doi.org/10.1007/978-3-642-04271-3_29.

[16] O'Bryant SE, Johnson LA, Barber RC, Braskie MN, Christian B, Hall JR, et al. The Health & Aging Brain among Latino Elders (HABLE) study methods and participant characteristics. Alzheimer's & Dementia: Diagnosis, Assessment & Disease Monitoring 2021;13. https://doi.org/10.1002/dad2.12202.

[17] Nelson CA, Frankeberger J, Chambers CD. An introduction to the HEALthy Brain and Child Development Study (HBCD) study. Dev Cogn Neurosci 2024;69:101441. https://doi.org/10.1016/J.DCN.2024.101441.

[18] Richie-Halford A, Cieslak M, Ai L, Caffarra S, Covitz S, Franco AR, et al. An analysis-ready and quality controlled resource for pediatric brain white-matter research. Scientific Data 2022 9:1 2022;9:1–27. https://doi.org/10.1038/s41597-022-01695-7.

[19] Van Essen DC, Ugurbil K, Auerbach E, Barch D, Behrens TEJ, Bucholz R, et al. The Human Connectome Project: A data acquisition perspective. Neuroimage 2012;62:2222–31. https://doi.org/10.1016/j.neuroimage.2012.02.018.

[20] Bookheimer SY, Salat DH, Terpstra M, Ances BM, Barch DM, Buckner RL, et al. The Lifespan Human Connectome Project in Aging: An overview. Neuroimage 2019;185:335–48. https://doi.org/10.1016/J.NEUROIMAGE.2018.10.009.

[21] Howell BR, Styner MA, Gao W, Yap PT, Wang L, Baluyot K, et al. The UNC/UMN Baby Connectome Project (BCP): An overview of the study design and protocol development. Neuroimage 2019;185:891–905. https://doi.org/10.1016/j.neuroimage.2018.03.049.

[22] Harms MP, Somerville LH, Ances BM, Andersson J, Barch DM, Bastiani M, et al. Extending the Human Connectome Project across ages: Imaging protocols for the Lifespan Development and Aging projects. Neuroimage 2018;183:972–84. https://doi.org/10.1016/j.neuroimage.2018.09.060.

[23] Wolff JJ, Gu H, Gerig G, Elison JT, Styner M, Gouttard S, et al. Differences in white matter fiber tract development present from 6 to 24 months in infants with autism. Am J Psychiatry 2012;169:589–600. https://doi.org/10.1176/APPI.AJP.2011.11091447.

[24] Mazziotta JC, Woods R, Iacoboni M, Sicotte N, Yaden K, Tran M, et al. The myth of the normal, average human brain—The ICBM experience: (1) Subject screening and eligibility. Neuroimage 2009;44:914–22. https://doi.org/10.1016/j.neuroimage.2008.07.062.

[25] Lytle MN, McNorgan C, Booth JR. A longitudinal neuroimaging dataset on multisensory lexical processing in school-aged children. Scientific Data 2019 6:1 2019;6:1–12. https://doi.org/10.1038/s41597-019-0338-5.

[26] Cai LY, Yang Q, Kanakaraj P, Nath V, Newton AT, Edmonson HA, et al. MASiVar: Multisite, multiscanner, and multisubject acquisitions for studying variability in diffusion weighted MRI. Magn Reson Med 2021;86:3304–20. https://doi.org/10.1002/MRM.28926.
[27] Beekly DL, Ramos EM, Lee WW, Deitrich WD, Jacka ME, Wu J, et al. The National Alzheimer's Coordinating Center (NACC) database: The uniform data set. Alzheimer Dis Assoc Disord 2007;21:249–58. https://doi.org/10.1097/WAD.0B013E318142774E.
[28] Nooner KB, Colcombe SJ, Tobe RH, Mennes M, Benedict MM, Moreno AL, et al. The NKI-Rockland sample: A model for accelerating the pace of discovery science in psychiatry. Front Neurosci 2012;6:32787. https://doi.org/10.3389/FNINS.2012.00152/BIBTEX.
[29] Jernigan TL, Brown TT, Hagler DJ, Akshoomoff N, Bartsch H, Newman E, et al. The Pediatric Imaging, Neurocognition, and Genetics (PING) Data Repository. Neuroimage 2015;124:1149. https://doi.org/10.1016/J.NEUROIMAGE.2015.04.057.
[30] Strike LT, Hansell NK, Chuang KH, Miller JL, de Zubicaray GI, Thompson PM, et al. The Queensland Twin Adolescent Brain Project, a longitudinal study of adolescent brain development. Scientific Data 2023 10:1 2023;10:1–18. https://doi.org/10.1038/s41597-023-02038-w.
[31] Bennett DA, Schneider JA, Arvanitakis Z, Wilson RS. OVERVIEW AND FINDINGS FROM THE RELIGIOUS ORDERS STUDY. Curr Alzheimer Res 2012;9:628. https://doi.org/10.2174/156720512801322573.
[32] Bennett DA, Schneider JA, Buchman AS, De Leon CM, Bienias JL, Wilson RS. The Rush Memory and Aging Project: Study Design and Baseline Characteristics of the Study Cohort. Neuroepidemiology 2005;25:163–75. https://doi.org/10.1159/000087446.
[33] Barnes LL, Shah RC, Aggarwal NT, Bennett DA, Schneider JA. The Minority Aging Research Study: Ongoing Efforts to Obtain Brain Donation in African Americans without Dementia. Curr Alzheimer Res 2012;9:734. https://doi.org/10.2174/156720512801322627.
[34] SCAN n.d. https://scan.naccdata.org/ (accessed November 2, 2025).
[35] SLIM n.d. https://doi.org/10.15387/fcp_indi.retro.slim.
[36] Smith D V., Sharp CJ, Dachs A, Wyngaarden J, Sazhin D, Yang Y, et al. Social reward and nonsocial reward processing across the adult lifespan: An interim multi-echo fMRI and diffusion dataset. Data Brief 2024;56:110810. https://doi.org/10.1016/J.DIB.2024.110810).
[37] Social Reward and Nonsocial Reward Processing Across the Adult Lifespan: An Interim Multi-echo fMRI and Diffusion Dataset - OpenNeuro n.d. https://openneuro.org/datasets/ds005123/versions/1.1.3 (accessed January 13, 2025).
[38] UCLA Consortium for Neuropsychiatric Phenomics LA5c Study - OpenNeuro n.d. https://openneuro.org/datasets/ds000030/versions/1.0.0 (accessed January 13, 2025).
[39] Alfaro-Almagro F, Jenkinson M, Bangerter NK, Andersson JLR, Griffanti L, Douaud G, et al. Image processing and Quality Control for the first 10,000 brain imaging datasets from UK Biobank. Neuroimage 2018;166:400–24. https://doi.org/10.1016/J.NEUROIMAGE.2017.10.034.
[40] Cognitive Training - OpenNeuro n.d. https://openneuro.org/datasets/ds002843/versions/1.0.1 (accessed January 13, 2025).
[41] Kable JW, Caulfield MK, Falcone M, McConnell M, Bernardo L, Parthasarathi T, et al. No effect of commercial cognitive training on brain activity, choice behavior, or cognitive

performance. Journal of Neuroscience 2017;37:7390–402. https://doi.org/10.1523/JNEUROSCI.2832-16.2017.

[42] Wang J, Lytle MN, Weiss Y, Yamasaki BL, Booth JR. A longitudinal neuroimaging dataset on language processing in children ages 5, 7, and 9 years old. Sci Data 2022;9. https://doi.org/10.1038/S41597-021-01106-3.

[43] Jefferson AL, Gifford KA, Acosta LMY, Bell SP, Donahue MJ, Davis LT, et al. The Vanderbilt Memory & Aging Project: Study Design and Baseline Cohort Overview. Journal of Alzheimer's Disease 2016;52:539–59. https://doi.org/10.3233/JAD-150914.

[44] Sager MA, Hermann B, La Rue A. Middle-aged children of persons with Alzheimer's disease: APOE genotypes and cognitive function in the Wisconsin Registry for Alzheimer's Prevention. J Geriatr Psychiatry Neurol 2005;18:245–9. https://doi.org/10.1177/0891988705281882.

[45] Wasserthal J, Neher P, Maier-Hein KH. TractSeg - Fast and accurate white matter tract segmentation. Neuroimage 2018;183:239–53. https://doi.org/10.1016/J.NEUROIMAGE.2018.07.070.

[46] Liu Y, Hsu CCH, Huang CC, Zhang Y, Zhao J, Tsai SJ, et al. Connectivity-Based Topographical Changes of the Corpus Callosum During Aging. Front Aging Neurosci 2021;13:753236. https://doi.org/10.3389/FNAGI.2021.753236/BIBTEX.

[47] Zhu AH, Nir TM, Javid S, Villalón-Reina JE, Rodrigue AL, Strike LT, et al. Lifespan reference curves for harmonizing multi-site regional brain white matter metrics from diffusion MRI. Scientific Data 2025 12:1 2025;12:1–18. https://doi.org/10.1038/s41597-025-05028-2.

[48] Bethlehem RAI, Seidlitz J, White SR, Vogel JW, Anderson KM, Adamson C, et al. Brain charts for the human lifespan. Nature 2022 604:7906 2022;604:525–33. https://doi.org/10.1038/s41586-022-04554-y.

[49] Storsve AB, Fjell AM, Yendiki A, Walhovd KB. Longitudinal Changes in White Matter Tract Integrity across the Adult Lifespan and Its Relation to Cortical Thinning. PLoS One 2016;11:e0156770. https://doi.org/10.1371/journal.pone.0156770.

[50] Giorgio A, Santelli L, Tomassini V, Bosnell R, Smith S, De Stefano N, et al. Age-related changes in grey and white matter structure throughout adulthood. Neuroimage 2010;51:943–51. https://doi.org/10.1016/j.neuroimage.2010.03.004.

[51] Beck D, de Lange A-MG, Maximov II, Richard G, Andreassen OA, Nordvik JE, et al. White matter microstructure across the adult lifespan: A mixed longitudinal and cross-sectional study using advanced diffusion models and brain-age prediction. Neuroimage 2021;224:117441. https://doi.org/10.1016/j.neuroimage.2020.117441.

[52] Henriques RN, Henson R, Correia MM. Unique information from common diffusion MRI models about white-matter differences across the human adult lifespan. Imaging Neuroscience 2023;1:1–25. https://doi.org/10.1162/imag_a_00051.

[53] Toschi N, Gisbert RA, Passamonti L, Canals S, De Santis S. Multishell diffusion imaging reveals sex-specific trajectories of early white matter degeneration in normal aging. Neurobiol Aging 2020;86:191–200. https://doi.org/10.1016/j.neurobiolaging.2019.11.014.

[54] Lebel C, Gee M, Camicioli R, Wieler M, Martin W, Beaulieu C. Diffusion tensor imaging of white matter tract evolution over the lifespan. Neuroimage 2012;60:340–52. https://doi.org/10.1016/j.neuroimage.2011.11.094.

[55] Slater DA, Melie-Garcia L, Preisig M, Kherif F, Lutti A, Draganski B. Evolution of white matter tract microstructure across the life span. Hum Brain Mapp 2019;40:2252–68. https://doi.org/10.1002/hbm.24522.
[56] Yeatman JD, Wandell BA, Mezer AA. Lifespan maturation and degeneration of human brain white matter. Nat Commun 2014;5:4932. https://doi.org/10.1038/ncomms5932.
[57] Groves AR, Smith SM, Fjell AM, Tamnes CK, Walhovd KB, Douaud G, et al. Benefits of multi-modal fusion analysis on a large-scale dataset: Life-span patterns of inter-subject variability in cortical morphometry and white matter microstructure. Neuroimage 2012;63:365–80. https://doi.org/10.1016/j.neuroimage.2012.06.038.
[58] Hoagey DA, Rieck JR, Rodrigue KM, Kennedy KM. Joint contributions of cortical morphometry and white matter microstructure in healthy brain aging: A partial least squares correlation analysis. Hum Brain Mapp 2019;40:5315–29. https://doi.org/10.1002/hbm.24774.
[59] Kochunov P, Glahn DC, Lancaster J, Thompson PM, Kochunov V, Rogers B, et al. Fractional anisotropy of cerebral white matter and thickness of cortical gray matter across the lifespan. Neuroimage 2011;58:41–9. https://doi.org/10.1016/j.neuroimage.2011.05.050.
[60] Bender AR, Raz N. Normal-appearing cerebral white matter in healthy adults: mean change over 2 years and individual differences in change. Neurobiol Aging 2015;36:1834–48. https://doi.org/10.1016/j.neurobiolaging.2015.02.001.
[61] Sexton CE, Walhovd KB, Storsve AB, Tamnes CK, Westlye LT, Johansen-Berg H, et al. Accelerated Changes in White Matter Microstructure during Aging: A Longitudinal Diffusion Tensor Imaging Study. The Journal of Neuroscience 2014;34:15425–36. https://doi.org/10.1523/JNEUROSCI.0203-14.2014.
[62] Sala S, Agosta F, Pagani E, Copetti M, Comi G, Filippi M. Microstructural changes and atrophy in brain white matter tracts with aging. Neurobiol Aging 2012;33:488-498.e2. https://doi.org/10.1016/j.neurobiolaging.2010.04.027.
[63] Westlye LT, Walhovd KB, Dale AM, Bjornerud A, Due-Tonnessen P, Engvig A, et al. Life-Span Changes of the Human Brain White Matter: Diffusion Tensor Imaging (DTI) and Volumetry. Cerebral Cortex 2010;20:2055–68. https://doi.org/10.1093/cercor/bhp280.
[64] Billiet T, Vandenbulcke M, Mädler B, Peeters R, Dhollander T, Zhang H, et al. Age-related microstructural differences quantified using myelin water imaging and advanced diffusion MRI. Neurobiol Aging 2015;36:2107–21. https://doi.org/10.1016/j.neurobiolaging.2015.02.029.
[65] Molloy CJ, Nugent S, Bokde ALW. Alterations in Diffusion Measures of White Matter Integrity Associated with Healthy Aging. The Journals of Gerontology: Series A 2021;76:945–54. https://doi.org/10.1093/gerona/glz289.
[66] Ardekani S, Kumar A, Bartzokis G, Sinha U. Exploratory voxel-based analysis of diffusion indices and hemispheric asymmetry in normal aging. Magn Reson Imaging 2007;25:154–67. https://doi.org/10.1016/j.mri.2006.09.045.
[67] Schilling KG, Chad JA, Chamberland M, Nozais V, Rheault F, Archer D, et al. White matter tract microstructure, macrostructure, and associated cortical gray matter morphology across the lifespan. Imaging Neuroscience 2023;1:1–24. https://doi.org/10.1162/IMAG_A_00050/118326/WHITE-MATTER-TRACT-MICROSTRUCTURE-MACROSTRUCTURE.

[68] Conte S, Zimmerman D, Richards JE. White matter trajectories over the lifespan. PLoS One 2024;19. https://doi.org/10.1371/JOURNAL.PONE.0301520.
[69] Jahanshad N, Kochunov P V., Sprooten E, Mandl RC, Nichols TE, Almasy L, et al. Multi-site genetic analysis of diffusion images and voxelwise heritability analysis: A pilot project of the ENIGMA–DTI working group. Neuroimage 2013;81:455–69. https://doi.org/10.1016/J.NEUROIMAGE.2013.04.061.
[70] Schilling KG, Tax CMW, Rheault F, Landman BA, Anderson AW, Descoteaux M, et al. Prevalence of white matter pathways coming into a single white matter voxel orientation: The bottleneck issue in tractography. Hum Brain Mapp 2022;43:1196–213. https://doi.org/10.1002/HBM.25697;PAGE:STRING:ARTICLE/CHAPTER.
[71] Maier-Hein KH, Neher PF, Houde JC, Côté MA, Garyfallidis E, Zhong J, et al. The challenge of mapping the human connectome based on diffusion tractography. Nature Communications 2017 8:1 2017;8:1–13. https://doi.org/10.1038/s41467-017-01285-x.
[72] Villalón-Reina JE, Zhu AH, Benavidez S, Moreau CA, Feng Y, Chattopadhyay T, et al. Lifespan Normative Modeling of Brain Microstructure. BioRxiv 2024:2024.12.15.628527. https://doi.org/10.1101/2024.12.15.628527.
[73] Veraart J, Christiaens D, Dai E, Edwards LJ, Golkov V, Mohammadi S, et al. A data-driven variability assessment of brain diffusion MRI preprocessing pipelines. Proceedings of the Joint Annual ISMRM-ESMRMB Meeting, 2022.
[74] Dubos J, Park SK, Vlasova R, Prieto JC, Styner M. Dmriprep: open-source diffusion MRI quality control framework with graphical user interface. Https://DoiOrg/101117/122654470 2023;12464:759–63. https://doi.org/10.1117/12.2654470.
[75] Oguz I, Farzinfar M, Matsui J, Budin F, Liu Z, Gerig G, et al. DTIPrep: quality control of diffusion-weighted images. Front Neuroinform 2014;8:4. https://doi.org/10.3389/fninf.2014.00004.
[76] Yeh FC. DSI Studio: an integrated tractography platform and fiber data hub for accelerating brain research. Nature Methods 2025 22:8 2025;22:1617–9. https://doi.org/10.1038/s41592-025-02762-8.
[77] Leemans A, Jeurissen B, Sijbers J, Jones DK. ExploreDTI: A Graphical Toolbox for Processing, Analyzing, and Visualizing Diffusion MR Data. 17th Annual Meeting of The International Society for Magnetic Resonance in Medicine, Honolulu, HI: 2009.
[78] Irfanoglu MO, Nayak A, Jenkins J, Pierpaoli C. TORTOISEv3: Improvements and New Features of the NIH Diffusion MRI Processing Pipeline. 25th Annual Meeting of The International Society for Magnetic Resonance in Medicine, Honolulu: 2017.
[79] Huang S, Zhong L, Shi Y. Automated Mapping of Residual Distortion Severity in Diffusion MRI. Computational Diffusion MRI : MICCAI Workshop 2024;14328:58. https://doi.org/10.1007/978-3-031-47292-3_6.
[80] Get Docker | Docker Docs n.d. https://docs.docker.com/get-started/get-docker/ (accessed April 2, 2025).
[81] Di Biase MA, Tian YE, Bethlehem RAI, Seidlitz J, Alexander-Bloch AF, Thomas Yeo BT, et al. Mapping human brain charts cross-sectionally and longitudinally. Proc Natl Acad Sci U S A 2023;120:e2216798120. https://doi.org/10.1073/PNAS.2216798120/SUPPL_FILE/PNAS.2216798120.SAPP.PDF.
[82] Cole TJ. Sample size and sample composition for constructing growth reference centiles. Stat Methods Med Res 2020;30:488. https://doi.org/10.1177/0962280220958438.

[83] Fan Q, Anderson AW, Davis N, Cutting LE. Structural connectivity patterns associated with the putative visual word form area and childrens reading ability. Brain Res 2014;1586:118–29. https://doi.org/10.1016/j.brainres.2014.08.050.
[84] Wang Y, Mauer M V., Raney T, Peysakhovich B, Becker BLC, Sliva DD, et al. Development of Tract-Specific White Matter Pathways During Early Reading Development in At-Risk Children and Typical Controls. Cereb Cortex 2017;27:2469–85. https://doi.org/10.1093/cercor/bhw095.
[85] Vandermosten M, Boets B, Wouters J, Ghesquière P. A qualitative and quantitative review of diffusion tensor imaging studies in reading and dyslexia. Neurosci Biobehav Rev 2012;36:1532–52. https://doi.org/10.1016/j.neubiorev.2012.04.002.
