# Supplementary material for: White matter micro- and macrostructure brain charts for the human lifespan
Source: Nature. 2026 May 13;655(8124):979–89. doi: 10.1038/s41586-026-10454-2 (PMC13391363; doi:10.1038/s41586-026-10454-2)
Supplement: Supplementary file 2 — Reporting Summary [file 41586_2026_10454_MOESM2_ESM.pdf]

nature portfolio

Corresponding author(s): Michael E. Kim

Last updated by author(s): Mar 9, 2026

Reporting Summary

Nature Portfolio wishes to improve the reproducibility of the work that we publish. This form provides structure for consistency and transparency in reporting. For further information on Nature Portfolio policies, see our Editorial Policies and the Editorial Policy Checklist.

Statistics

For all statistical analyses, confirm that the following items are present in the figure legend, table legend, main text, or Methods section.

n/a	Confirmed	
☐	☒	The exact sample size (*n*) for each experimental group/condition, given as a discrete number and unit of measurement
☐	☒	A statement on whether measurements were taken from distinct samples or whether the same sample was measured repeatedly
☐	☒	The statistical test(s) used AND whether they are one- or two-sided<br>*Only common tests should be described solely by name; describe more complex techniques in the Methods section.*
☐	☒	A description of all covariates tested
☐	☒	A description of any assumptions or corrections, such as tests of normality and adjustment for multiple comparisons
☐	☒	A full description of the statistical parameters including central tendency (e.g. means) or other basic estimates (e.g. regression coefficient) AND variation (e.g. standard deviation) or associated estimates of uncertainty (e.g. confidence intervals)
☐	☒	For null hypothesis testing, the test statistic (e.g. F, t, r) with confidence intervals, effect sizes, degrees of freedom and P value noted<br>*Give P values as exact values whenever suitable.*
☒	☐	For Bayesian analysis, information on the choice of priors and Markov chain Monte Carlo settings
☐	☒	For hierarchical and complex designs, identification of the appropriate level for tests and full reporting of outcomes
☐	☒	Estimates of effect sizes (e.g. Cohen's d, Pearson's r), indicating how they were calculated

Our web collection on statistics for biologists contains articles on many of the points above.

Software and code

Policy information about availability of computer code

Data collection	No custom software was used by the investigators to collect data for this study.
Data analysis	Code to both build and use the white matter brain charts described in this study is available as a Docker image at the following Zenodo repository: https://zenodo.org/records/15367425. We have included documentation, including extensive tutorials and instructions, on how researchers can both use our brain charts and create their own using our code. Furthermore, we have publicly released the post-processing pipeline as a single containerized image with instructions on how to run the code (with the preprocessing container already available) in order to facilitate researchers being able to use our released brain charts- intructions: https://zenodo.org/records/17144460; post-processing container: https://hub.docker.com/r/kimm58/wm_lifespan_processing; pre-processing container: https://doi.org/10.5281/zenodo.14058394.<br><br>All analysis visualizations and statistics were generated using Python version 3.9.20. Notable python libraries used are pandas (v2.2.3), numpy (v2.0.2), seaborn (v0.13.2), matplotlib (v3.9.2), rpy2 (v3.5.11), and scipy (v1.13.1). Heatmaps to visualize large matrices were created using "heatmap" in seaborn. Density plots were created using "violin_plot" in seaborn. Datapoints and trajectories across the lifespan were plotted using "scatterplot" from seaborn and "plot" in matplotlib respectively, with the "age" axis scaled logarithmically. Histograms were created using the "hist" function in matplotlib. Linear regressions with confidence intervals were created using "regplot" in seaborn. Graphical representations of white matter tracts in the brain were created using mrview from MRtrix3. Two-sided, one-sample Wilcoxon tests were run using "scipy.stats.wilcoxon" from scipy. Mann Whitney.16-U tests were run using "scipy.stats.mannwhitneyu". For creating 3D qualitative visualizations of white matter tracts, we use the mrview tool inside the MRtrix3 (version 3.0.4). Alternatively, for qualitative region of interest 3D visualizations, the OpenDIVE visualization library (v0.3.0, https://github.com/MASILab/open_dive) was used, where a different Python version, 3.10.18, was used in these visualization instances for library compatibility purposes. For Figure 6C, the graphical head is a combination of a FreeSurfer (v7.2) cortical segmentation of a brain scan (which was made by the authors) and graphics (gears, head outline) that were hand-sketched using graphic design software (krita v5.2.16). For processing of the MRI data, we use several open source tools and

pipelines, such as FSL (v6.0.4), MRtrix3 (v3.0.4), ANTs (v2.5.0), scilpy (v1.5.0), synbo-DISCO (v3.1), FreeSurfer (v7.2), and TractSeg (v2.8). A more detailed description of the data processing used can be found in the methods section of the manuscript.

For manuscripts utilizing custom algorithms or software that are central to the research but not yet described in published literature, software must be made available to editors and reviewers. We strongly encourage code deposition in a community repository (e.g. GitHub). See the Nature Portfolio guidelines for submitting code & software for further information.

Data

Policy information about availability of data

All manuscripts must include a data availability statement. This statement should provide the following information, where applicable:

- Accession codes, unique identifiers, or web links for publicly available datasets
- A description of any restrictions on data availability
- For clinical datasets or third party data, please ensure that the statement adheres to our policy

All derived data and associated demographic information from publicly available datasets, as well as from datasets for which the Data Use Agreement (DUA) permits sharing, have been made accessible at https://zenodo.org/records/18891847. For datasets where the DUA does not permit redistribution of derivative data, these materials have been returned to or shared directly with the respective data custodians.

Data from the Alzheimer's Disease Neuroimaging Initiative (ADNI) are available upon request from https://adni.loni.usc.edu/. Data from the AOMIC-PIOP1 dataset are freely available for download on OpenNeuro: https://openneuro.org/datasets/ds002785/versions/2.0.0. Data from the AOMIC-PIOP2 dataset are freely available for download on OpenNeuro: https://openneuro.org/datasets/ds002790/versions/2.0.0. Data from the AOMIC-ID1000 dataset are freely available for download on OpenNeuro: https://openneuro.org/datasets/ds003097/versions/1.2.1. Data from the Boston Adolescent Neuroimaging of Depression and Anxiety (BANDA) dataset are available upon request from https://www.humanconnectome.org/study/connectomes-related-anxiety-depression. Data from BIOCARD are available upon request after filling out a data use application: https://www.gaaindata.org/partner/BIOCARD. Data from the Baltimore Longitudinal Study of Aging (BLSA) are available upon request from https://www.blsa.nih.gov/. Data from the Calgary Preschool MRI Dataset are freely available for download at: https://doi.org/10.17605/OSF.IO/AXZ5R. Data from the Centre for Attention Learning and Memory (CALM) dataset are available upon request from https://calm.mrc-cbu.cam.ac.uk/researchers/. Data from the Cambridge Center for Ageing Neuroscience (CAMCAN) dataset are available upon request from https://camcan-archive.mrc-cbu.cam.ac.uk/dataaccess/. Data from the Dallas Lifespan Brain Study (DLBS) dataset are freely available for download on OpenNeuro: https://openneuro.org/datasets/ds004856/versions/1.2.0. Data from the Health & Aging Brain Study - Health Disparities (HABS-HD) dataset are available upon request from https://apps.unthsc.edu/itr/reports. Imaging data and basic demographic information for the Healthy Brain Network (HBN) dataset are freely available to download from https://fcon_1000.projects.nitrc.org/indi/cmi_healthy_brain_network/. Phenotypic data are available upon request by filling out a data use agreement: https://fcon_1000.projects.nitrc.org/indi/cmi_healthy_brain_network/Phenotypic.html. Data from the Human Connectome Project – Aging (HCPA) dataset are available upon request from https://www.humanconnectome.org/study/hcp-lifespan-aging. Data from the Lifespan Baby Connectome Project (HCPBaby) dataset are available upon request https://www.humanconnectome.org/study/lifespan-baby-connectome-project. Data from the Human Connectome Project – Development (HCPD) dataset are available upon request from https://www.humanconnectome.org/study/hcp-lifespan-development. Data from the Human Connectome Project – Young Adult (HCP) dataset are freely available for download from https://www.humanconnectome.org/study/hcp-young-adult. Data from the Infant Brain Imaging Study (IBIS) are available for download from the National Institutes of Mental Health data archive upon request from https://nda.nih.gov/edit_collection.html?id=19. Data from the International Consortium for Brain Mapping (ICBM) dataset are available upon request from www.loni.usc.edu/ICBM. Data from the Longitudinal Brain Correlates of Multisensory Lexical Processing in Children study (shortened to Lexical in this manuscript) are freely available for download on OpenNeuro: https://openneuro.org/datasets/ds001894/versions/1.4.2. Data from the Memory and Aging Project (MAP), Religious Orders Study (ROS), and the Minority Aging Research Study (MARS) datasets are available upon request from https://www.radc.rush.edu/. Data from the Multisite, Multiscanner, and Multisubject Acquisitions for Studying Variability in Diffusion Weighted Magnetic Resonance Imaging (MASiVar) dataset are freely available for download on OpenNeuro: https://openneuro.org/datasets/ds003416/versions/2.0.2. Data from the National Alzheimer's Coordinating Center (NACC) and the Standardized Centralized Alzheimer's & Related Dementias Neuroimaging (SCAN) are available upon request from https://naccdata.org/requesting-data/data-request-process. Imaging data and basic demographic information for the Nathan Kline Institute – Rockland Sample (NKI) dataset are freely available to download from https://fcon_1000.projects.nitrc.org/indi/enhanced/sharing_neuro.html. Phenotypic data are available upon request by filling out a data use agreement: https://fcon_1000.projects.nitrc.org/indi/enhanced/sharing_phenotypic.html. Data from the Pediatric Imaging, Neurocognition, and Genetics dataset (PING) are available for download from the National Institutes of Mental Health data archive upon request from https://nda.nih.gov/edit_collection.html?id=2607. Data from the Queensland Twin Adolescent Brain (QTAB) dataset are freely available for download on OpenNeuro: https://openneuro.org/datasets/ds004146/versions/1.0.4. Data from the UCLA Consortium for Neuropsychiatric Phenomics LA5c Study (UCLA) dataset are freely available for download on OpenNeuro: https://openneuro.org/datasets/ds000030/versions/1.0.0. Data from the Southwest University (SWU) Longitudinal Imaging Multimodal dataset are freely available for download here: https://fcon_1000.projects.nitrc.org/indi/retro/southwestuni_qiu_index.html. Data from the Social Reward and Nonsocial Reward Processing Across the Adult Lifespan: An Interim Multi-echo fMRI and Diffusion Dataset (referred to as TempleSocial in this manuscript) are freely available for download on OpenNeuro: https://openneuro.org/datasets/ds005123/versions/1.1.3. Data from UK Biobank (UKBB) are available upon request from https://www.ukbiobank.ac.uk/. Data from the UPennRisk dataset are freely available for download on OpenNeuro: https://openneuro.org/datasets/ds002843/versions/1.0.1. Data from the dataset titled "A longitudinal neuroimaging dataset on language processing in children ages 5, 7, and 9 years old" (referred to as UTAustin579 in this manuscript) are freely available for download on OpenNeuro: https://openneuro.org/datasets/ds003604/versions/1.0.7. Data from the Vanderbilt Memory and Aging Project (VMAP_JEFFERSON, VMAP_2.0, TN Aging Project) are available for download upon request from https://vmacdata.org/vmap/data-requests. Data from the Wisconsin Registry for Alzheimer's Prevention (WRAP) are available upon request from https://wrap.wisc.edu/data-requests-2/. Data from the HEALthy Brain and Child Development (HBCD) Study are available for download upon request from (https://hbcdstudy.org/data-sharing/). Data from the Adolescent Brain Cognitive Development (ABCD) Study are available for download upon request from (https://abcdstudy.org/scientists/data-sharing/). Data used in this manuscript from the Ageility Project (Phase 1 only) are available for download from (https://www.nitrc.org/projects/age-ility). Data from the Early Brain Development in Twins (EBDT) dataset are available for download from the National Institutes of Mental Health data archive upon request from https://nda.nih.gov/edit_collection.html?id=2384. Data from the Bipolar & Schizophrenia Consortium for Parsing Intermediate Phenotypes dataset (BSNIP1) and its renewal (BSNIP2) are available for download from the National Institutes of Mental Health data archive upon request from https://nda.nih.gov/edit_collection.html?id=2274 and https://nda.nih.gov/edit_collection.html?id=2165. Data from the Developing Human Connectome Project (dHCP) are available for download from the National Institutes of Mental Health data archive upon request from https://nda.nih.gov/edit_collection.html?id=3955. Vanderbilt University data (MORGAN, BABIES-ABC, CUTTING, VUMC-ASD) subject to third party restrictions. Please contact corresponding authors for data requests.

Research involving human participants, their data, or biological material

Policy information about studies with human participants or human data. See also policy information about sex, gender (identity/presentation), and sexual orientation and race, ethnicity and racism.

Reporting on sex and gender	Sex was used as a covariate of interest in the study and reported according to the source dataset-specific policies. For instances when sex information was unavailable from the individual dataset, reported gender was used as a replacement variable.
Reporting on race, ethnicity, or other socially relevant groupings	No information on race/ethnicity was used for analyses in the manuscript. However, we report the aggregate demographic information regarding race/ethnicity in the Supplemental Information (Supplemental Table SB.T1), where such demographic information was reported by the original individual datasets.
Population characteristics	Age was reported in years, whereas for neonatal datasets it was reported as gestational age. Diagnoses were determined according to the source dataset-specific policies; further information about how individuals were diagnosed for each dataset can be found in the dataset specific references in the Supplemental Information (Supplemental Table SB.T2).
Recruitment	Analyses in the manuscript were based on existing datasets; further information about how individuals were recruited for each dataset can be found in the dataset specific references in the Supplemental Information (Supplemental Table SB.T2).
Ethics oversight	The following statement has been added to the Methods section:<br><br>The research consists of secondary analyses of de-identified primary datasets. Information regarding informed consent of participants (or guardians) in primary studies can be found in the references in Supplemental Table SB.T2. Secondary analysis of these data was also approved by the Vanderbilt University Medical School Institutional Review Board (IRB #210968).

Note that full information on the approval of the study protocol must also be provided in the manuscript.

Field-specific reporting

Please select the one below that is the best fit for your research. If you are not sure, read the appropriate sections before making your selection.

☒ Life sciences ☐ Behavioural & social sciences ☐ Ecological, evolutionary & environmental sciences

For a reference copy of the document with all sections, see nature.com/documents/nr-reporting-summary-flat.pdf

Life sciences study design

All studies must disclose on these points even when the disclosure is negative.

Sample size	Sample size was determined based on the number of participant scans for any particular cohort (either typically developing/aging or clinical) that passed the quality control procedures detailed in the methods section. Although no a priori sample size was calculated, we conduct variability assessments of the models (Supplemental Figure SD.F3) in addition to the built-in ML optimization of our models to ensure that data and brain charts are robust.
Data exclusions	As described in the methods, data were excluded from analyses if age, sex, or diagnosis was missing for the participant at the time of scan, or if the data did not pass the quality control procedures.
Replication	All data processing was standardized through the use of version-controlled containerized code for replicability. Furthermore, we have publicly released the post-processing pipeline as a single containerized image with instructions on how to run the code (with the preprocessing container already available) - intructions: https://zenodo.org/records/17144460; post-processing container: https://hub.docker.com/repository/docker/kimm58/wm_lifespan_processing/general; pre-processing container: https://doi.org/10.5281/zenodo.14058394.
Randomization	Allocation was based on the diagnosis specified by each individual dataset. Participants were assigned to the clinical group that was indicated in the source dataset that they originated from. Typically developing/aging participants were those that were specified as either part of a control group or having no diagnosis present.
Blinding	Blinding was not applicable/not possible for a few reasons. First, we used pre-existing MRI datasets from multiple sources and did not acquire MRI data for the purposes of this study. Second, knowledge of diagnostic status was essential to our study design, as we specifically required identification of typically developing/aging individuals to construct normative models. Furthermore, clinical labels were necessary for evaluating deviations from normative trajectories in clinical groups. As such, blinding to group allocation would have been incompatible with the objectives of the study.

Reporting for specific materials, systems and methods

We require information from authors about some types of materials, experimental systems and methods used in many studies. Here, indicate whether each material, system or method listed is relevant to your study. If you are not sure if a list item applies to your research, read the appropriate section before selecting a response.

Materials & experimental systems

n/a	Involved in the study
☒	☐ Antibodies
☒	☐ Eukaryotic cell lines
☒	☐ Palaeontology and archaeology
☒	☐ Animals and other organisms
☒	☐ Clinical data
☒	☐ Dual use research of concern
☒	☐ Plants

Methods

n/a	Involved in the study
☒	☐ ChIP-seq
☒	☐ Flow cytometry
☐	☒ MRI-based neuroimaging

Plants

Seed stocks

Report on the source of all seed stocks or other plant material used. If applicable, state the seed stock centre and catalogue number. If plant specimens were collected from the field, describe the collection location, date and sampling procedures.

Novel plant genotypes

Describe the methods by which all novel plant genotypes were produced. This includes those generated by transgenic approaches, gene editing, chemical/radiation-based mutagenesis and hybridization. For transgenic lines, describe the transformation method, the number of independent lines analyzed and the generation upon which experiments were performed. For gene-edited lines, describe the editor used, the endogenous sequence targeted for editing, the targeting guide RNA sequence (if applicable) and how the editor was applied.

Authentication

Describe any authentication procedures for each seed stock used or novel genotype generated. Describe any experiments used to assess the effect of a mutation and, where applicable, how potential secondary effects (e.g. second site T-DNA insertions, mosiacism, off-target gene editing) were examined.

Magnetic resonance imaging

Experimental design

Design type

Design specifications were dependent on the previously collected datasets used in this study. However, all scanning sessions used in these analyses required a diffusion-weighted MRI scan.

Design specifications

For data collection, design specifications were dependent on the previously collected datasets used in this study. Some datasets were longitudinal in nature or had repeated scans while others were cross-sectional. Regardless of how many data points were collected, we used only a single scan for each participant.

Behavioral performance measures

Any behavioral performance measures collected for the original datasets used in this study were not used in any analyses.

Acquisition

Imaging type(s)

Diffusion and structural

Field strength

Variable: dependent on the dataset and scanner used for each particular source dataset.

Sequence & imaging parameters

Variable: dependent on the dataset and scanner used for each particular source dataset.

Area of acquisition

Whole brain scan

Diffusion MRI ☒ Used ☐ Not used

Parameters

Variable: dependent on the dataset and scanner used for each particular source dataset.

Preprocessing

Preprocessing software

The preprocessing software used was the PreQual software package developed by Cai et al. (2021), which corrects for susceptibility-, eddy current-, and motion-induced artifacts. PreQual also performs denoising of images and performs slice-wise signal imputation for slices with signal dropout. When available, reverse phase encoding scans were used to correct for susceptibility-induced distortions. Otherwise, a T1-weighted image from the same scanning session was used for the correction with Synbo-DisCo developed by Schilling et al. (2019).

Code (as a container) and instructions on running the preprocessing pipeline can be found here: https://doi.org/10.5281/zenodo.14058394

Normalization

For scans where multiple NIFTI files were preprocessed together (e.g. including a reverse phase encoding scan for distortion correction), intensity normalization across NIFTIs was performed in the PreQual package. For creating the normative models,

dataset-specific biases were modeled as covariates to perform harmonization across multiple datasets.

Normalization template

Data were not normalized to a standard space for this study.

Noise and artifact removal

All denoising and image distortion removal was performed on the diffusion-weighted MRI scans using the PreQual preprocessing package. The denoising comes from MRtrix3's dwidenoise (PCA denoising). Susceptibility-, eddy current-, and motion-induced artifacts were corrected using FSL's topup and eddy. Slice-wise signal imputation for slices with signal dropout was also performed by FSL's eddy.

Volume censoring

No volume censoring was used in this study.

Statistical modeling & inference

Model type and settings

Generalized additive models for location, scale, and shape (GAMLSS) were used to create the normative models described in the manuscript, specifying a generalized gamma distribution. Age was modeled non-linearly using fractional polynomials of one to three terms (model selection was done using Bayesian information criterion) whereas sex was modeled linearly. Dataset/batch was modeled using random effect terms for intercept.

Effect(s) tested

Our study was not based on task or stimulus conditions. Instead, we investigated normative variation in white matter for typically developing/aging individuals across the human lifespan using demographic variables of age and sex (with a random intercept for dataset/batch). For clinical groups, we assessed deviations of centile score distributions based on the median distance from the median centile.

Specify type of analysis: ☐ Whole brain ☐ ROI-based ☒ Both

Anatomical location(s)

Anatomical locations where determined by Tractseg, a deep learning algorithm that segments white matter bundles in the brain. Cerebral white matter was identified using a white matter mask derived from the FreeSurfer segmentation tool.

Statistic type for inference
(See Eklund et al. 2016)

We did not include voxel-wise or cluster-wise analyses in this study.

Correction

Bonferroni correction, obtained based on the number of tests evaluated for an analysis.

Models & analysis

n/a	Involved in the study	
☒	☐	Functional and/or effective connectivity
☒	☐	Graph analysis
☐	☒	Multivariate modeling or predictive analysis

Multivariate modeling and predictive analysis

Independent variables were age in years, sex as a binary variable, and dataset/batch as a random effect term. The GAMLSS framework was used to model white matter features (linearly and non-linearly) using these independent variables. Model selection was performed using Bayesian information criterion.
