# Supplementary material for: White matter micro- and macrostructure brain charts for the human lifespan
Source: Nature. 2026 May 13;655(8124):979–89. doi: 10.1038/s41586-026-10454-2 (PMC13391363; doi:10.1038/s41586-026-10454-2)
Supplement: Supplementary file 3 — Datasets. [file 41586_2026_10454_MOESM3_ESM.pdf]

Data used in preparation of this article were obtained from the Alzheimer's Disease Neuroimaging Initiative (ADNI) database (adni.loni.usc.edu). As such, the investigators within the ADNI contributed to the design and implementation of ADNI and/or provided data but did not participate in analysis or writing of this report. A complete listing of ADNI investigators can be found at: http://adni.loni.usc.edu/wp-content/uploads/how_to_apply/ADNI_Acknowledgement_List.pdf.

Data used in preparation of this article were derived from BIOCARD study, supported by grant U19 – AG033655 from the National Institute on Aging. The BIOCARD study team did not participate in the analysis or writing of this report, however, they contributed to the design and implementation of the study. A listing of BIOCARD investigators can be found on the BIOCARD website (on the 'BIOCARD Data Access Procedures' page, 'Acknowledgement Agreement' document.

Data and/or research tools used in the preparation of this manuscript were obtained from the National Institute of Mental Health (NIMH) Data Archive (NDA). NDA is a collaborative informatics system created by the National Institutes of Health to provide a national resource to support and accelerate research in mental health. This manuscript reflects the views of the authors and may not reflect the opinions or views of the NIH or of the Submitters submitting original data to NDA.

The Pediatric Imaging, Neurocognition, and Genetics (PING) dataset was collected and released openly to contribute to the assessment of typical brain development in a pediatric sample (RC2DA029475-01), (https://www.sciencedirect.com/science/article/pii/S1053811915003572).

The data used in this study come from the Human Connectome Project, which aims to map the structural connections and circuits of the brain and their relationships to behavior by acquiring high-quality magnetic resonance images. We used T1-weighted and diffusion MRI data from the Lifespan Baby Connectome Project (HCPBaby), the Human Connectome Project Development (HCPD) study, the Human Connectome Project Young Adult (HCP) study, the developing Human Connectome Project (dHCP), and the Human Connectome Project Aging (HCPA) study.

Data were provided by the Human Connectome Project, WU-Minn Consortium (Principal Investigators: David Van Essen and Kamil Ugurbil; 1U54MH091657) funded by the 16 NIH Institutes and Centers that support the NIH Blueprint for Neuroscience Research; and by the McDonnell Center for Systems Neuroscience at Washington University.

Research data from the Infant Brain Imaging Study (IBIS) dataset come from the IBIS Autism project, a collaborative effort by investigators to conduct a longitudinal MRI/DTI and behavioral study of infants at high risk for autism (R01HD055741-01).

The Enhanced NKI-RS is a large cross-sectional sample of brain development, maturation and aging, that is currently funded by the NIMH (BRAINS R01MH094639-01; PI Milham) and Child Mind Institute (PI Milham) to characterize 1000 community-ascertained participants using state-of-the-art multiplex imaging-based resting state fMRI (R-fMRI) and diffusion tensor imaging (DTI), genetics, and a broad neurobehavioral phenotypic characterization protocol.

Imaging data and basic demographic information for the Nathan Kline Institute – Rockland Sample (NKI) dataset are freely available to download from https://fcon_1000.projects.nitrc.org/indi/enhanced/sharing_neuro.html. Phenotypic data are available upon request by filling out a data use agreement: https://fcon_1000.projects.nitrc.org/indi/enhanced/sharing_phenotypic.html.

The Healthy Brain Network (HBN) is an ongoing initiative focused on building a biobank of data from 10,000 children and adolescents (ages 5-21) in the New York City area (https://www.nature.com/articles/sdata2017181). Imaging data and basic demographic information for the Healthy Brain Network (HBN) dataset are freely available to download from https://fcon_1000.projects.nitrc.org/indi/cmi_healthy_brain_network/. Phenotypic data are available upon request by filling out a data use agreement: https://fcon_1000.projects.nitrc.org/indi/cmi_healthy_brain_network/Phenotypic.html.

Data collection and sharing for this project was provided by the Cambridge Centre for Ageing and Neuroscience (CamCAN). CamCAN funding was provided by the UK Biotechnology and Biological Sciences Research Council (grant number BB/H008217/1), together with support from the UK Medical Research Council and University of Cambridge, UK. Data used in the preparation of this work were obtained from the CamCAN repository (available at http://www.mrc-cbu.cam.ac.uk/datasets/camcan/). The Cambridge Centre for Ageing and Neuroscience (Cam-CAN) is a large-scale collaborative research project at the University of Cambridge.

VMAP data were collected by Vanderbilt Memory and Alzheimer’s Center Investigators at Vanderbilt University Medical Center. VMAP began in 2012 with the goal of investigating vascular health and brain aging.

BLSA is a prospective cohort study with continuous enrollment that began in 1958. Comprehensive data from BLSA are available upon request by a proposal submission through the cohort website (www.blsa.nih.gov). The BLSA is supported by the Intramural Research Program of the National Institute on Aging, NIH.

Data collection and sharing for ADNI were supported by National Institutes of Health Grant U01-AG024904 and Department of Defense (award number W81XWH-12-2-0012). ADNI is also funded by the National Institute on Aging, the National Institute of Biomedical Imaging and Bioengineering, and through generous contributions from the following: AbbVie, Alzheimer’s Association; Alzheimer’s Drug Discovery Foundation; Araclon Biotech; BioClinica, Inc.; Biogen; Bristol-Myers Squibb Company; CereSpir, Inc.; Cogstate; Eisai Inc.; Elan Pharmaceuticals, Inc.; Eli Lilly and Company; EuroImmun; F. Hoffmann-La Roche Ltd and its affiliated company Genentech, Inc.; Fujirebio; GE Healthcare; IXICO Ltd.; Janssen Alzheimer Immunotherapy Research & Development, LLC.; Johnson & Johnson Pharmaceutical Research & Development LLC.; Lumosity; Lundbeck; Merck & Co., Inc.; Meso Scale Diagnostics, LLC.; NeuroRx Research; Neurotrack Technologies; Novartis Pharmaceuticals Corporation; Pfizer Inc.; Piramal Imaging; Servier; Takeda Pharmaceutical Company; and Transition Therapeutics. The Canadian Institutes of Health Research is providing funds to support ADNI clinical sites in Canada. Private sector contributions are facilitated by the Foundation for the National Institutes

of Health (www.fnih.org). The grantee organization is the Northern California Institute for Research and Education, and the study is coordinated by the Alzheimer's Therapeutic Research Institute at the University of Southern California. ADNI data are disseminated by the Laboratory for Neuro Imaging at the University of Southern California. Data used in the preparation of this article were obtained from the Alzheimer's Disease Neuroimaging Initiative (ADNI) database (adni.loni.usc.edu). The ADNI was launched in 2003 as a public private partnership, led by Principal Investigator Michael W. Weiner, MD. The original goal of ADNI was to test whether serial magnetic resonance imaging (MRI), positron emission tomography (PET), other biological markers, and clinical and neuropsychological assessment can be combined to measure the progression of mild cognitive impairment (MCI) and early Alzheimer's disease (AD). The current goals include validating biomarkers for clinical trials, improving the generalizability of ADNI data by increasing diversity in the participant cohort, and to provide data concerning the diagnosis and progression of Alzheimer's disease to the scientific community. For up-to-date information, see adni.loni.usc.edu.

Research reported in this publication as a part of the Health and Aging Brain Study: Health Disparities (HABS-HD) was supported by the National Institute on Aging of the National Institutes of Health under Award Numbers R01AG054073 and R01AG058533, R01AG070862, P41EB015922 and U19AG078109. The content is solely the responsibility of the authors and does not necessarily represent the official views of the National Institutes of Health.

The NACC database is funded by NIA/NIH Grant U24 AG072122. NACC data are contributed by the NIA-funded ADRCs: P30 AG062429 (PI James Brewer, MD, PhD), P30 AG066468 (PI Oscar Lopez, MD), P30 AG062421 (PI Bradley Hyman, MD, PhD), P30 AG066509 (PI Thomas Grabowski, MD), P30 AG066514 (PI Mary Sano, PhD), P30 AG066530 (PI Helena Chui, MD), P30 AG066507 (PI Marilyn Albert, PhD), P30 AG066444 (PI John Morris, MD), P30 AG066518 (PI Jeffrey Kaye, MD), P30 AG066512 (PI Thomas Wisniewski, MD), P30 AG066462 (PI Scott Small, MD), P30 AG072979 (PI David Wolk, MD), P30 AG072972 (PI Charles DeCarli, MD), P30 AG072976 (PI Andrew Saykin, PsyD), P30 AG072975 (PI David Bennett, MD), P30 AG072978 (PI Ann McKee, MD), P30 AG072977 (PI Robert Vassar, PhD), P30 AG066519 (PI Frank LaFerla, PhD), P30 AG062677 (PI Ronald Petersen, MD, PhD), P30 AG079280 (PI Eric Reiman, MD), P30 AG062422 (PI Gil Rabinovici, MD), P30 AG066511 (PI Allan Levey, MD, PhD), P30 AG072946 (PI Linda Van Eldik, PhD), P30 AG062715 (PI Sanjay Asthana, MD, FRCP), P30 AG072973 (PI Russell Swerdlow, MD), P30 AG066506 (PI Todd Golde, MD, PhD), P30 AG066508 (PI Stephen Strittmatter, MD, PhD), P30 AG066515 (PI Victor Henderson, MD, MS), P30 AG072947 (PI Suzanne Craft, PhD), P30 AG072931 (PI Henry Paulson, MD, PhD), P30 AG066546 (PI Sudha Seshadri, MD), P20 AG068024 (PI Erik Roberson, MD, PhD), P20 AG068053 (PI Justin Miller, PhD), P20 AG068077 (PI Gary Rosenberg, MD), P20 AG068082 (PI Angela Jefferson, PhD), P30 AG072958 (PI Heather Whitson, MD), P30 AG072959 (PI James Leverenz, MD).

This research has been conducted using the UK Biobank resource, application 16315.

Data contributed from MAP/ROS/MARS was supported by NIA R01AG017917, P30AG10161, P30AG072975, R01AG022018, R01AG056405, UH2NS100599, UH3NS100599,

R01AG064233, R01AG15819 and R01AG067482, and the Illinois Department of Public Health (Alzheimer's Disease Research Fund). Data can be accessed at www.radc.rush.edu. More information about participant demographics and study information can be found here: https://www.rushu.rush.edu/research-rush-university/departmental-research/rush-alzheimers-disease-center/rush-alzheimers-disease-center-research/epidemiologic-research.

The data contributed from the Wisconsin Registry for Alzheimer's Prevention was supported by NIA AG021155, AG0271761, AG037639, and AG054047.

Data collection and sharing for this project was provided by the Centre for Attention, Learning and Memory (CALM). CALM funding was provided by the UK Medical Research Council and University of Cambridge, UK. Data used in the preparation of this work were obtained from CALM resource – https://calm.mrc-cbu.cam.ac.uk/. The study protocol is reported in Holmes et al. (2019).

Data used in the preparation of this work were obtained from the International Consortium for Brain Mapping (ICBM) database (www.loni.usc.edu/ICBM). The ICBM project (Principal Investigator John Mazziotta, M.D., University of California, Los Angeles) is supported by the National Institute of Biomedical Imaging and BioEngineering. ICBM is the result of efforts of co-investigators from UCLA, Montreal Neurologic Institute, University of Texas at San Antonio, and the Institute of Medicine, Juelich/Heinrich Heine University - Germany. Data collection and sharing for this project was provided by the International Consortium for Brain Mapping (ICBM; Principal Investigator: John Mazziotta, MD, PhD). ICBM funding was provided by the National Institute of Biomedical Imaging and BioEngineering. ICBM data are disseminated by the Laboratory of Neuro Imaging at the University of Southern California.

The dataset that we refer to as UTAustin in this manuscript is a longitudinal neuroimaging dataset on language processing in children ages 5, 7, and 9 years old collected at The University of Texas at Austin. The dataset is openly available from Openneuro here: https://openneuro.org/datasets/ds003604/versions/1.0.7. We use version 1.0.7.

The Queensland Twin Adolescent Brain (QTAB) Project was established with the purpose of promoting the conduct of health-related research in adolescence. The QTAB dataset comprises multimodal neuroimaging, as well as cognitive and mental health data collected in adolescent twins over two sessions. The MRI protocol consisted of T1-weighted (MP2RAGE), T2-weighted, FLAIR, high-resolution TSE, SWI, resting-state fMRI, DWI, and ASL scans. The QTAB project resource was produced as a result of i) the goodwill and contribution of 422 twin/triplet participants and their parents, ii) funding from the National Health and Medical Research Council, Australia (APP1078756) and the Queensland Brain Institute, University of Queensland, iii) access to several key resources, including the Centre for Advanced Imaging, the Human Studies Unit, Institute of Molecular Bioscience, and the Queensland Cyber Infrastructure Foundation, at the University of Queensland, local and national twin registries at the QIMR Berghofer Medical Research Institute and Twin Research Australia, as well as the many assessments made available by researchers worldwide, and iv) was established with the purpose of promoting the conduct of health-related research in adolescence. The imaging data and basic

demographics are openly accessibly on Openneuro here: https://openneuro.org/datasets/ds004146/versions/1.0.4. We use version 1.0.4.

The Social Reward and Nonsocial Reward Processing Across the Adult Lifespan: An Interim Multi-echo fMRI and Diffusion Dataset (referred to as TempleSocial in this manuscript) comes from a study that aims to investigate whether older adults have a blunted response to some features of social reward. The dataset is openly accessibly on Openneuro here: https://openneuro.org/datasets/ds005123/versions/1.1.3. In particular, we use version 1.1.3.

The Longitudinal Brain Correlates of Multisensory Lexical Processing in Children study (shortened to Lexical in this manuscript) aims to explore developmentally dependent changes in lexical processing for adolescents. The dataset is openly accessibly on Openneuro here: https://openneuro.org/datasets/ds001894/versions/1.4.2. We use version 1.4.2.

The UCLA Consortium for Neuropsychiatric Phenomics LA5c Study (UCLA) is focused on understanding the dimensional structure of memory and cognitive control (response inhibition) functions in both healthy individuals and individuals with neuropsychiatric disorders including schizophrenia, bipolar disorder, and attention deficit/hyperactivity disorder. Neuroimaging data were downloaded from Openneuro here: https://openneuro.org/datasets/ds000030/versions/1.0.0. We use version 1.0.0.

The dataset we refer to as UPennRisk comes from a study at University of Pennsylvania that investigated whether training executive cognitive function could influence choice behavior and brain responses. Neuroimaging data were downloaded from Openneuro here: https://openneuro.org/datasets/ds002843/versions/1.0.1. We use version 1.0.1.

The Dallas Lifespan Brain Study (DLBS) is a longitudinal multi-modal neuroimaging study of the aging mind, which was initiated in 2008 (referred to as Wave 1). Participants returned for two additional waves of data collection with an approximate interval of 4-5 years between waves. The DLBS protocol encompasses various imaging modalities, including structural MRI, diffusion MRI, and functional MRI, as well as comprehensive cognitive and psychosocial assessments. DLBS data can be downloaded from Openneuro here: https://openneuro.org/datasets/ds004856. Specifically, we use version 1.2.0.

The Multisite, Multiscanner, and Multisubject Acquisitions for Studying Variability in Diffusion Weighted Magnetic Resonance Imaging (MASiVar) dataset consists of 319 diffusion scans acquired at 3T from b = 1000 to 3000 s/mm2 across 14 healthy adults, 83 healthy children (5 to 8 years), three sites, and four scanners curated to promote investigation of diffusion MRI variability. In particular, we used only the data coming from healthy children (Cohort IV) for version 2.0.2 of the dataset. Data are available to download from Openneuro here: https://openneuro.org/datasets/ds003416/versions/2.0.2.

Data coming from the Southwestern University (SWU) dataset, part of the Consortium for Reliability and Reproducibility (CoRR), were downloaded via NITRC-IR from the 1000 Functional Connectomes Project. Specifically, the data come from the Emotion and Creativity One Year Retest Dataset subset, comprised of 235 subjects, all of whom were college students.

Each subject underwent two sessions of anatomical, resting state fMRI, and DTI scans, spaced one year apart. In order to access the CoRR datasets through NITRC, users must be logged into NITRC at the time of download and registered with the 1000 Functional Connectomes Project / INDI website. More information about this subset can be found here: https://fcon_1000.projects.nitrc.org/indi/CoRR/html/swu_4.html.

The Preschool MRI study in The Developmental Neuroimaging Lab at the University of Calgary (https://www.developmentalneuroimaginglab.ca) uses different magnetic resonance imaging (MRI) techniques to study brain structure and function in early childhood. The study aims to characterize brain development in early childhood, and to offer baseline data that can be used to understand cognitive and behavioural development, as well as to identify deviations from normal development in children with various diseases, disorders, or brain injuries. The MRI techniques used include diffusion tensor imaging (DTI), anatomical imaging, arterial spin labeling (ASL), and resting state functional MRI (rsfMRI). Data can be downloaded from here: https://osf.io/axz5r/.

The Amsterdam Open MRI Collection (AOMIC) is a collection of three datasets with multimodal (3T) MRI data including structural (T1-weighted), diffusion-weighted, and (resting-state and task-based) functional BOLD MRI data, as well as detailed demographics and psychometric variables from a large set of healthy participants. All raw data is publicly available from the Openneuro data sharing platform: ID1000: https://openneuro.org/datasets/ds003097, PIOP1: https://openneuro.org/datasets/ds002785, PIOP2: https://openneuro.org/datasets/ds002790. We use version 1.2.1 for ID1000 and 2.0.0 for PIOP1 and PIOP2.

The Boston Adolescent Neuroimaging of Depression and Anxiety (BANDA) is a study of 215 adolescents ages 14-17, 152 of whom had a current diagnosis of a DSM-5 (APA, 2013) anxious and/or depressive disorder. The BANDA study collected a rich dataset of brain, clinical, and cognitive/neuropsychological measures from these adolescent subjects. The dataset is available to download upon request on the NDA.

The NACC database is funded by NIA/NIH Grant U24 AG072122. SCAN is a multi-institutional project that was funded as a U24 grant (AG067418) by the National Institute on Aging in May 2020. Data collected by SCAN and shared by NACC are contributed by the NIA-funded ADRCs as follows:

Arizona Alzheimer’s Center - P30 AG072980 (PI: Eric Reiman, MD); R01 AG069453 (PI: Eric Reiman (contact), MD); P30 AG019610 (PI: Eric Reiman, MD); and the State of Arizona which provided additional funding supporting our center; Boston University - P30 AG013846 (PI Neil Kowall MD); Cleveland ADRC - P30 AG062428 (James Leverenz, MD); Cleveland Clinic, Las Vegas – P20AG068053; Columbia - P50 AG008702 (PI Scott Small MD); Duke/UNC ADRC – P30 AG072958; Emory University - P30AG066511 (PI Levey Allan, MD, PhD); Indiana University - R01 AG19771 (PI Andrew Saykin, PsyD); P30 AG10133 (PI Andrew Saykin, PsyD); P30 AG072976 (PI Andrew Saykin, PsyD); R01 AG061788 (PI Shannon Risacher, PhD); R01 AG053993 (PI Yu-Chien Wu, MD, PhD); U01 AG057195 (PI Liana Apostolova, MD); U19 AG063911 (PI Bradley Boeve, MD); and the Indiana University Department of Radiology and

Imaging Sciences; Johns Hopkins - P30 AG066507 (PI Marilyn Albert, Phd.); Mayo Clinic - P50 AG016574 (PI Ronald Petersen MD PhD); Mount Sinai - P30 AG066514 (PI Mary Sano, PhD); R01 AG054110 (PI Trey Hedden, PhD); R01 AG053509 (PI Trey Hedden, PhD); New York University - P30AG066512-01S2 (PI Thomas Wisniewski, MD); R01AG056031 (PI Ricardo Osorio, MD); R01AG056531 (PIs Ricardo Osorio, MD; Girardin Jean-Louis, PhD); Northwestern University - P30 AG013854 (PI Robert Vassar PhD); R01 AG045571 (PI Emily Rogalski, PhD); R56 AG045571, (PI Emily Rogalski, PhD); R01 AG067781, (PI Emily Rogalski, PhD); U19 AG073153, (PI Emily Rogalski, PhD); R01 DC008552, (M.-Marsel Mesulam, MD); R01 AG077444, (PIs M.-Marsel Mesulam, MD, Emily Rogalski, PhD); R01 NS075075 (PI Emily Rogalski, PhD); R01 AG056258 (PI Emily Rogalski, PhD); Oregon Health and Science University - P30 AG008017 (PI Jeffrey Kaye MD); R56 AG074321 (PI Jeffrey Kaye, MD); Rush University - P30 AG010161 (PI David Bennett MD); Stanford – P30AG066515; P50 AG047366 (PI Victor Henderson MD MS); University of Alabama, Birmingham – P20; University of California, Davis - P30 AG10129 (PI Charles DeCarli, MD); P30 AG072972 (PI Charles DeCarli, MD); University of California, Irvine - P50 AG016573 (PI Frank LaFerla PhD); University of California, San Diego - P30AG062429 (PI James Brewer, MD, PhD); University of California, San Francisco - P30 AG062422 (Rabinovici, Gil D., MD); University of Kansas - P30 AG035982 (Russell Swerdlow, MD); University of Kentucky - P30 AG028283-15S1 (PIs Linda Van Eldik, PhD and Brian Gold, PhD); University of Michigan ADRC - P30AG053760 (PI Henry Paulson, MD, PhD) P30AG072931 (PI Henry Paulson, MD, PhD) Cure Alzheimer's Fund 200775 - (PI Henry Paulson, MD, PhD) U19 NS120384 (PI Charles DeCarli, MD, University of Michigan Site PI Henry Paulson, MD, PhD) R01 AG068338 (MPI Bruno Giordani, PhD, Carol Persad, PhD, Yi Murphey, PhD) S10OD026738-01 (PI Douglas Noll, PhD) R01 AG058724 (PI Benjamin Hampstead, PhD) R35 AG072262 (PI Benjamin Hampstead, PhD) W81XWH2110743 (PI Benjamin Hampstead, PhD) R01 AG073235 (PI Nancy Chiaravalloti, University of Michigan Site PI Benjamin Hampstead, PhD) 1I01RX001534 (PI Benjamin Hampstead, PhD) IRX001381 (PI Benjamin Hampstead, PhD); University of New Mexico - P20 AG068077 (Gary Rosenberg, MD); University of Pennsylvania - State of PA project 2019NF4100087335 (PI David Wolk, MD); Rooney Family Research Fund (PI David Wolk, MD); R01 AG055005 (PI David Wolk, MD); University of Pittsburgh - P50 AG005133 (PI Oscar Lopez MD); University of Southern California - P50 AG005142 (PI Helena Chui MD); University of Washington - P50 AG005136 (PI Thomas Grabowski MD); University of Wisconsin - P50 AG033514 (PI Sanjay Asthana MD FRCP); Vanderbilt University – P20 AG068082; Wake Forest - P30AG072947 (PI Suzanne Craft, PhD); Washington University, St. Louis - P01 AG03991 (PI John Morris MD); P01 AG026276 (PI John Morris MD); P20 MH071616 (PI Dan Marcus); P30 AG066444 (PI John Morris MD); P30 NS098577 (PI Dan Marcus); R01 AG021910 (PI Randy Buckner); R01 AG043434 (PI Catherine Roe); R01 EB009352 (PI Dan Marcus); UL1 TR000448 (PI Brad Evanoff); U24 RR021382 (PI Bruce Rosen); Avid Radiopharmaceuticals / Eli Lilly; Yale - P50 AG047270 (PI Stephen Strittmatter MD PhD); R01AG052560 (MPI: Christopher van Dyck, MD; Richard Carson, PhD); R01AG062276 (PI: Christopher van Dyck, MD); 1Florida - P30AG066506-03 (PI Glenn Smith, PhD); P50 AG047266 (PI Todd Golde MD PhD)

Data used in the preparation of this article were obtained from the HEALthy Brain and Child Development (HBCD) Study (https://hbcdstudy.org/), held in the NIH Brain Development

Cohorts Data Sharing Platform. This is a multisite, longitudinal study designed to recruit approximately 7,000 families and follow them from pregnancy to early childhood.

The HBCD Study is supported by the NIH and additional federal partners under award numbers U01DA055352, U01DA055353, U01DA055366, U01DA055365, U01DA055362, U01DA055342, U01DA055360, U01DA055350, U01DA055338, U01DA055355, U01DA055363, U01DA055349, U01DA055361, U01DA055316, U01DA055344, U01DA055322, U01DA055369, U01DA055358, U01DA055371, U01DA055359, U01DA055354, U01DA055370, U01DA055347, U01DA055357, U01DA055367, U24DA055325, and U24DA055330. A full list of supporters is available at https://hbcdstudy.org/federal-partners/.

A full list of participating sites is available at: https://hbcdstudy.org/recruitment-sites/. HBCD Study Consortium investigators designed and implemented the study and/or provided data but did not necessarily participate in the analysis or writing of this report. This manuscript reflects the views of the authors and may not reflect the opinions or views of the NIH or the HBCD Study Consortium investigators.

Data used in the preparation of this article were obtained from the Adolescent Brain Cognitive DevelopmentSM (ABCD) Study (https://abcdstudy.org), held in the NIMH Data Archive (NDA). This is a multisite, longitudinal study designed to recruit more than 10,000 children age 9-10 and follow them over 10 years into early adulthood. The ABCD Study® is supported by the National Institutes of Health and additional federal partners under award numbers U01DA041048, U01DA050989, U01DA051016, U01DA041022, U01DA051018, U01DA051037, U01DA050987, U01DA041174, U01DA041106, U01DA041117, U01DA041028, U01DA041134, U01DA050988, U01DA051039, U01DA041156, U01DA041025, U01DA041120, U01DA051038, U01DA041148, U01DA041093, U01DA041089, U24DA041123, U24DA041147. A full list of supporters is available at https://abcdstudy.org/federal-partners.html. A listing of participating sites and a complete listing of the study investigators can be found at https://abcdstudy.org/consortium_members/. ABCD consortium investigators designed and implemented the study and/or provided data but did not necessarily participate in the analysis or writing of this report. This manuscript reflects the views of the authors and may not reflect the opinions or views of the NIH or ABCD consortium investigators.

The Bipolar & Schizophrenia Consortium for Parsing Intermediate Phenotypes (BSNIP1; 10.15154/tnzs-a323) dataset (R01MH078113-01, R01MH077852-01, R01MH077851-01, R01MH077945-01, R01MH077862-01) and its renewal (BSNIP2; 10.15154/8v3w-et72) (R01MH103368-01, R01MH103366-01) were collected with the aim to improve diagnosis and clinical management of psychosis by defining biologically based biotypes that better predict symptoms, course, and treatment response across major psychoses.

The Early Brain Development in Twins (EBDT) dataset was collected to understand the role of genetic and environmental contributions to brain structure and function in the crucial period of development that is early childhood (U01MH070890-11). (10.15154/1822-fs72)

The Age-ility Project (Phase 1) dataset was collected with the aim of developing novel analysis approaches that integrate information across multiple brain imaging modalities (e.g., dMRI, fMRI, electrophysiology) to build a more complete picture of how brain networks are structurally and functionally organized to support cognition.

The Developing Human Connectome Project (dHCP) is an open science study, funded by the European Research Council to obtain and disseminate Magnetic Resonance Imaging (MRI) data which map the brain's structural and functional development across the period from 20 weeks gestational age to full term. By coupling advances in imaging with bespoke solutions developed for the fetal and neonatal population, principally but not exclusively solving the problems of subject motion, the dHCP captures the development of brain anatomy and connectivity at a systems level (10.15154/92vw-g837).

Data from the VUMC-ASD dataset are collected seeking to elucidate the neural mechanisms underlying atypical sensory processing in autism spectrum disorders (ASD), focusing on tactile and interoceptive sensitivity and their relationship to social and behavioral symptoms. By integrating behavioral, neurophysiological, and neuroimaging approaches, the studies aim to identify how alterations in thalamocortical and salience networks contribute to sensory hypo- and hyper-responsiveness in ASD, ultimately informing early identification and novel intervention strategies. Collection of these data were supported by K01MH090232, R21MH101321, and R01MH102272.

HABS-HD comes from UNT Health Fort Worth, with the following primary investigators:
Sid E O'Bryant (University of North Texas Health Science Center, Fort Worth, TX, USA); Kristine Yaffe (University of California San Francisco, San Francisco, CA, USA); Arthur Toga (University of Southern California, Los Angeles, CA, USA); Robert Rissman (University of Southern California, Los Angeles, CA, USA); and Leigh Johnson (University of North Texas Health Science Center, Fort Worth, TX, USA);

and the HABS-HD Investigators:

University of North Texas Health Science Center, Fort Worth, TX, USA: James R Hall, Melissa Petersen, Robert Barber, Fan Zhang, Rajesh Nandy, David Mason, Stephanie Large, Raul Vintimilla, Zhengyang Zhou, Rocky Vig, Nicole Phillips; University of Southern California, Los Angeles, CA, USA: Meredith Braskie, Kevin King, Michael Donohue, Rema Raman, Matthew Borzage, Yonggang Shi; Washington University in St. Louis, St. Louis, MO, USA: Beau Ances, Ganesh Babulal, Jorge Llibre-Guerra; University of Wisconsin–Madison, Madison, WI, USA: Bradley Christian, Amy Kind, Ozioma Okonkwo; Columbia University, New York, NY, USA: Badri Vardarajan, Joe Lee; Fordham University, New York, NY, USA: Monica Rivera Mindt; Georgetown University Medical Center, Washington, DC, USA: Amrita Cheema; The University of Texas Health Science Center, San Antonio, TX, USA: Raymond Palmer; University of Texas Southwestern Medical Center, Dallas, TX, USA: Roderick McColl; Rush University Medical Center, Chicago, IL, USA: Lisa Barnes; University of California Irvine School of Medicine, Irvine, CA, USA: Mark Mapstone; University of Pittsburgh School of Medicine, Pittsburgh, PA, USA: Annie Cohen; Wake Forest University School of Medicine,

Winston-Salem, NC, USA: Michelle Mielke; Alzheimer's Association, Chicago, IL, USA: Carl Hill

A complete listing of ADNI investigators can be found at: http://adni.loni.usc.edu/wp-content/uploads/how_to_apply/ADNI_Acknowledgement_List.pdf.

A complete list of BIOCARD investigators can be found here: https://biocard.pathology.jhu.edu/our-team/.
