# Supplementary material for: White matter micro- and macrostructure brain charts for the human lifespan
Source: Nature. 2026 May 13;655(8124):979–89. doi: 10.1038/s41586-026-10454-2 (PMC13391363; doi:10.1038/s41586-026-10454-2)
Supplement: Supplementary file 13 — Peer Review File [file 41586_2026_10454_MOESM13_ESM.pdf]

nature portfolio

Peer Review File

White matter micro and macrostructure brain charts for the human lifespan

Corresponding Author: Mr Michael Kim

Any redactions in this file are there to maintain patient confidentiality, the confidentiality of unpublished data, or to remove third-party material.

This file contains all reviewer reports in order by version, followed by all author rebuttals in order by version.

Attachments originally included by the reviewers as part of their assessment can be found at the end of this file.

Version 0:

Reviewer comments:

Referee #1

(Remarks to the Author)
The concept of normative brain charts (alike pediatric growth charts) has recently been extended to brain imaging, offering a valuable framework for quantifying brain structure variability across age and health. The present study provides such reference charts for white matter (WM) based on an impressive cross-sectional dataset (26,199 diffusion MRI scans!). While the present study is a tour de force, my enthusiasms has been tempered by some missing conceptual advances and explorations.

1) The study successfully maps age-related changes in both microstructural and macrostructural white matter properties. These trajectories vary considerably across tracts and features, capturing the heterogeneity of white matter development and degeneration. However, a key conceptual limitation is the lack of integration with long-standing histological data, such as Flechsig's maps of myelin development. Although tract-specific developmental curves are provided, the absence of a unified neuroanatomical framework limits the interpretability of the results. One would expect some theoretical synthesis with classical or contemporary models of myelination and maturation. This paper offers descriptive but not conceptual novelty in this regard.

2) The identification of tract-specific inflexion points and non-linear dynamics is definitely a forte of this work. Yet again, the lack of conceptual linkage to known patterns of myelin maturation reduces the depth of interpretation. Incorporating insights from Flechsig, Yakovlev, and Lecours, or even contemporary transcriptomic developmental maps, would have enriched the biological interpretation.

3) The facilitating of standardised scoring of new datasets is perhaps the most directly useful aspect of the paper. The availability of a harmonised reference enables future studies to score individuals or populations. This contribution is methodological and practical, and it lays the groundwork for future clinical tools. However, the authors propose the clinical relevance of these charts by projecting patient groups (e.g., with MS, schizophrenia) onto the normative curves and identifying deviations. While group-level deviations are statistically significant, no specificity/sensitivity analyses or ROC curves are provided to evaluate the clinical utility of this approach for diagnosis or prognosis. A formal assessment of accuracy (e.g., AUC, predictive validity) is essential to support claims of clinical translatability.

4) The authors state that “white matter is drastically more heritable than grey matter traits,” referencing large-scale genetic studies. However, the cited paper (likely Smith et al., 2021, Nat Neurosci) does not support this as a categorical or generalised statement. While some WM traits (e.g., FA in certain tracts) show higher heritability than some GM traits, the variation is considerable, and no single modality dominates across the board. Additionally, these findings were not tract-specific and may be confounded by cortical folding variability and registration noise. The authors should temper this claim accordingly.

6) The data is cross-sectional, not longitudinal, which is important as cross-sectional trends can mask within-subject

variability or cohort effects. This limitation should be acknowledged more explicitly.

7) The charts assume a normative “median” trajectory, but brain organisation varies across individuals. Studies such as Catani et al. (2007) have shown structural connectivity variants in the general population (e.g., absence of arcuate fasciculus in some individuals). Relying solely on median-based norms could misclassify healthy phenotypic variants as outliers. The lack of phenotypic subtyping (beyond sex) is a limitation that should be addressed in future work.

Minor Comments
There are typos in Reference 3 that should be corrected.

This study presents he first large-scale WM reference charts, which could become a valuable resource for the neuroimaging community. However, the paper could benefit from a stronger neuroanatomical foundation and clearer clinical validation metrics. Additionally, the assumption of a unitary "norm" may obscure biologically valid inter-individual diversity and personalised medicine, which are becoming a pressing clinical matter.

(Remarks on code availability)

Referee #2

(Remarks to the Author)
Michael E. Kim and colleagues present an important update to available "brain growth charts." They have used more than 26k MRI scans from 42 studies to yield derived brain measures of gross white matter anatomy in terms of tract morphometry (macrostructure) and across commonly used diffusion tensors (microstructure). Overall, this work is ambitious and represents a tangible advancement in describing the dynamic changes of brain structure across the human lifespan. This allows defining normative trajectories, identifying milestones in the trajectories, discovering pathology-related changes, and creatingtools to create centile scores for external samples. The work is likely to be highly interesting to a wide audience, including neuroscientists and other related disciplines. The choice of brain measures is the key strength of the manuscript, and it has the potential to bridge the volumetric and diffusivity brain centiles. I think this manuscript is original and sufficiently distinct from prior work. My conclusion is that with (admittedly substantial) additional work, this manuscript can be a highly relevant contribution by 1) serving as a benchmark work for the creation of national brain charts that are 'validated' in international samples, 2) providing a bridging effort between existing volumetric brain charts and DTI brain charts, and 3) creating standard protocols for tractography-based region of interest delineation to serve applied basic and clinical studies. My concerns about the current version of the manuscript are outlined below.

Representativeness of the neuroimaging data sets:

1. Given that the datasets of the current study are almost exclusively from the United States, I would suggest a rephrasing of the manuscript to brain charts of the North American population that could be 'tested/validated' by centile scoring of data sets from other countries (UK, Netherlands, etc.). This would not undermine the value of the work, in my opinion. Looking forward, it is highly likely that national brain growth charts are used jointly with international charts for assessing each population, similar to the growth charts of body growth charts that are in routine clinical use.

reference: Cole TJ. The development of growth references and growth charts. Ann Hum Biol. 2012 Sep;39(5):382-94. doi: 10.3109/03014460.2012.694475. Epub 2012 Jul 11. PMID: 22780429; PMCID: PMC3920659.

- Could the authors claim national representativeness of the US population? Would it be possible to increase the available data by increasing the coverage across different US states?

On a related note, the study is also missing a few notable data sets. Why is the ABCD study missing from the used data sets? It would be a key to increasing the national representativeness. Why do the authors only include ca. 10,000 UK Biobank participants when the available high-quality DTI data includes in excess of 57k scans, and depending on criteria, the amount of 'healthy' participants is ca. 30k scans? Why is the developing human connectome (dHCP) project data missing from the used data sets?

Comparisons to closely related work:

2. The brain charts for the chosen brain measures are original and increase the impact of the work. The authors used the largest amount of individual studies to build their source data but have a lower sample size than in prior work reporting the creation of DTI brain charts.

- Zhu et al. 2025: N = 40,898 - 10 studies
- Villalón-Reina et al. (preprint): N = 54,583 - 19 studies
- Kim et al. (manuscript): N=26,199 - 42 studies.

I would ask the authors to elaborate on how they justify that their sample is sufficient in terms of sample size and sampling strategy based on prior work (running appropriate simulations would be ideal).

reference: Bozek J, Griffanti L, Lau S, Jenkinson M. Normative models for neuroimaging markers: Impact of model selection, sample size, and evaluation criteria. Neuroimage. 2023 Mar;268:119864. doi: 10.1016/j.neuroimage.2023.119864. Epub 2023 Jan 5. PMID: 36621581; PMCID: PMC11636675.

3. There are important similarities and differences in DTI brain chart reports that would be important to summarize. I would ask the authors to provide a table in the supplementary materials that compares their work to other closely related work in terms of covered age range, sample characteristics, preprocessing, derived brain metrics, and tools to build the reference charts, etc., in comparison to references Zhu et al. 2025 & Villalón-Reina et al. (preprint).

4. Brain atlases vary from study to study, and some atlases have variable labels for identical 'white matter tracts.' I would ask the authors to provide an atlas comparison to Zhu et al. 2025, which used the ENIGMA consortium procedures and JHU white matter atlas to define the ROIs. This could be achieved, for instance, through reporting overlaps for the regions of interest in MNI space (TractSeg atlas vs. JHU atlas labels).

5. The authors state that they were successful in "identifying previously uncharacterized developmental milestones and spatial gradients of tract maturation." However, the developmental milestones are hard to verify from the current version of the manuscript due to differential plotting of age in comparison to prior work (which can be easily fixed by adjusting the plots). This would be especially relevant for plots reported in Zhu et al. 2025 & Villalón-Reina et al. (preprint), and the authors could focus on the ROIs that have overlaps with JHU atlas labels.

6. The gradients of tract maturation are a highly relevant and potentially novel aspect of the work, and my impression is that this should be explored in more detail. The authors should map out and visualize known spatial gradients, for instance, the posterior-to-anterior brain developmental pattern. This would also be informative for testing the “last-in, first-out” / “gain-predicts-loss” hypotheses (as part of this work, not in the future as currently stated in the discussion). Authors might be able to make an even more solid case for mapping which of the tensor measures 'matures' and 'declines' in line with the hypotheses. This could be achieved by plotting the areas with one axis first-to-last peak and the other one first-to-last decline.

Comparisons to volumetric brain charts created with GAMLSS

7. The authors use GAMLSS to model the "normative trajectories" of white matter changes across the lifespan, which also enables tools to perform similar "centile scoring" for new studies, which is much in line with volumetric brain charts (Bethlehem et al. 2022). Indeed, many elements in the study are similar to Bethlehem et al. 2022 article, but at times it is unclear how the procedures were different. I would ask the authors to provide a supplementary table outlining similarities and differences to the 2022 brain charts article.

I would encourage a replication approach for the GAMLSS models and their validation and a clearly indicated and justified deviation from Bethlehelm et al. 2022. I would encourage the authors to include a leave-one-study-out (LOSO) validation of the growth centiles and benchmarking the similarity of the harmonization results of the out-of-sample centile scoring with existing methods (that in this case could be eHarmonize from Zhu et al. 2025).

- The authors have chosen the Generalized Gamma distribution. This might not be optimal for FA values that range from 0 to 1.

8. White matter volume charts seem to be in line with prior reports in Figure 1. What is the level of agreement of the total WM volume charts (from FreeSurfer) to those reported by the Bethlehem et al. article? Showing strong agreement would strengthen the study by showing a potential comparability to established gross anatomical WM charts.

9. It is important to be open about the parts of the brain that are not covered in the regions of interest. Do the total WM vs. sum of TractSeg volumes match so that the entire white matter is covered? If not, which parts of the brain are not included in the tract-specific analyses?

10. Strikingly, males and females seem to have almost identical DTI tensor brain charts in most presented centile curves. This is somewhat surprising and also differs from prior work but could well be a true finding. This would be important to emphasize in the main manuscript text and to address in additional analyses. Clearly, there are some differences in TBSS-based and tractography-based data that would require detailed comparisons. Would the sex differences be apparent in smaller, eroded tracts? Another aspect that might be worth including in the comparative analyses is related to volumes that are readily available for the authors. For a given DTI scan, participants that have larger brain volumes have more voxels within the DTI image volume. They would thus have higher proportional spatial resolution (similar to smaller voxel size), which could be detected as higher FA values. This type of negative association between FA and voxel volume is well depicted in Zhu et al. 2025 Figure 3.

Clinical phenotypes

11. The authors use the centile scores to compare patient vs. control groups across "neurological conditions" that span mild cognitive impairment (MCI), Alzheimer’s disease (AD), and epilepsy. The choice of these conditions is not clear to the reader, as none of them are white matter specific in pathophysiology and are not representative of neurological diseases in

general. In the supplements, there are also other case vs. control comparisons for "other clinical cohort groups": ADHD, autism, anxiety, depression, the individuals that convert from no cognitive impairments to either MCI or AD, and substance abuse, which is not clearly justified or referenced in the main manuscript. It could be a viable approach to replicate most of the abnormal phenotypes that were used in Bethlehem et al. 2022 article and ideally extend this to a chosen few additional 'clinical' phenotypes.

- There could also be added value in studying associations between case-control comparisons. Some ideas would be birth weight vs. global measures and language skill scores vs. 'language tracts.' This is relevant also since most of the data is from English-speaking countries, making language skill assessments potentially relevant.

- Authors should reconsider wording that is used in this part of the manuscript ("neurological conditions" / "other clinical cohorts" / "other cognitive groups"), i.e., think of more descriptive names that are used consistently.

Requirements for new centile scoring and future use of the resources

12. There are scant details about DTI preprocessing (citation to prior work is not sufficient). I would ask the authors to provide the key details of the procedures they used by listing the main steps and including a complete description in the supplementary materials.

On a related note, the authors are not testing how image quality measures might influence the DTI tensors and/or the centile scores. PreQual output includes multiple image quality measures, and the authors should both report these parameters in detail for each of the data sets and test if the centile estimation is robust to variability in image quality measures.

13. I was left wondering what the requirements are for new data sets to be suitable for centile scoring. The authors are encouraged to provide as much detail and some analyses using differential preprocessing for out-of-centile scoring. Would investigators need to use PreQual or a similar preprocessing workflow? Are there any requirements for image quality, such as a minimum number of diffusion-encoding directions?

This is highly relevant, as for many existing data sets it is important to maintain consistency by using the same data set in most studies. The key question is, could we use already preprocessed data as an input even if another software package/pipeline has been used for preprocessing? What is the minimum sample size that would be sufficient for centile creation?

14. This work is impressive in that it creates centiles for + 1000 brain measures. It is understandable that all of this information cannot be reported in the manuscript. I found the choice of regions of interest and left/right side that were chosen for main article figures arbitrary and, to a degree, non-informative. Could the main display items focus on a set of white matter tracts or bilateral examples from chosen 'tract groups'?

It would also strengthen the report if the authors created means for checking the ROI-wise centiles for all available measures. This might be too much for an article supplement, but maybe an online repository to share the ROI-wise figures or even a separate tool/container that can create the plots?

Minor comments

15. Minor comments

- The lack of population representativeness in most neuroimaging studies should be clearly stated as a limitation of this study as one of the " ... gaps (that) will require continued efforts to increase global diversity and dataset representativeness."

- Supplementary information could have a table of contents for easier navigation, and all elements in the supplementaries should be referenced in the main manuscript. The authors could consider splitting the supplementary materials to supplementary methods and supplementary results

- The available data is summarized nicely in Supplementary Table S1, but it would help the readers to have a PRISMA-style flow chart of the sample building steps, including the total amount of subjects that were excluded at each step for image quality concerns, due to failed tract reconstruction, or demographics, etc.

- Supplementary Table S1 could be elaborated by including the DTI sequence parameters, preprocessing (distortion correction), and site location (country, city, state). This could also be done in a separate table(s).

- Please review the supplements and online materials for clarity and minor typos.

Code and online resources

16. The authors provide the tools they used to build the brain charts openly for the scientific community through a containerized (Docker pipeline), available at https://zenodo.org/records/15538472. There are also some instructions for replication and model data sets that users can use to try the tools out.

- The authors may have made a typo: r_lifepsan_env.tar >>> r_lifespan_env.tar. They might also think about a more descriptive name that contains other keywords in the container name, such as "dti," "tract," "centile," etc.

- It would be important to provide a set of commands to run the recommended preprocessing (PreQual) and Docker containers wrapping the recommended post-processing (Mrtrix, TractSeg, scilpy). The prospective users could then follow the instructions and trust that their processing is using a fixed set of versions of the tools and is thus in line with recommendations.

- I would suggest that the authors make all the containerized tools available through Docker Hub. The authors should also ensure that Apptainer works for the tools and provide instructions for setting it up.

- The instructions can generally be followed with ease, but it might be beneficial to restructure the sections, e.g., a) installation and trying the installation with the use of model data, b) processing your own data (with example code to run an open access data set from unprocessed data to processed form), c) preparation of the demographics (more examples here for building the tables would not hurt), d) running the models for out-of-sample scoring, and e) creating normative models based on new data sets (with clear guidance on recommended sample size and other demographics).

- The online materials might benefit from more examples. For instance, changing the metric (other examples could include looping through the structures):

```
docker run --rm -v $PWD/testout:/OUTPUTS r_lifespan_env python3 /WMLifespan/scripts/output_centile_curves.py AF_left volume /OUTPUTS/centiles_volume_AF_left.csv

docker run --rm -v $PWD/testout:/OUTPUTS r_lifespan_env python3 /WMLifespan/scripts/output_centile_curves.py AF_left fa-mean /OUTPUTS/centiles_FA_AF_left.csv
```

Of note, these two commands run from any location that has the output directory, i.e., the provided model data input file MS_dataset.csv is not necessary. Making a version with commands where the 'model data' for creating the centiles is given as an input and not used from the Docker container itself would be useful. Having clear pointers in providing input and output is very important.

- Finally, code that was used for this work should be made openly available (as much as possible).

(Remarks on code availability)
The authors provide the tools they used to build the brain charts openly for the scientific community through a containerized (Docker pipeline), available at https://zenodo.org/records/15538472. There are also some instructions for replication and model data sets that users can use to try the tools out.

- The authors may have made a typo: r_lifepsan_env.tar >>> r_lifespan_env.tar. They might also think about a more descriptive name that contains other keywords in the container name, such as "dti," "tract," "centile," etc.

- It would be important to provide a set of commands to run the recommended preprocessing (PreQual) and Docker containers wrapping the recommended post-processing (Mrtrix, TractSeg, scilpy). The prospective users could then follow the instructions and trust that their processing is using a fixed set of versions of the tools and is thus in line with recommendations.

- I would suggest that the authors make all the containerized tools available through Docker Hub. The authors should also ensure that Apptainer works for the tools and provide instructions for setting it up.

- The instructions can generally be followed with ease, but it might be beneficial to restructure the sections, e.g., a) installation and trying the installation with the use of model data, b) processing your own data (with example code to run an open access data set from unprocessed data to processed form), c) preparation of the demographics (more examples here for building the tables would not hurt), d) running the models for out-of-sample scoring, and e) creating normative models based on new data sets (with clear guidance on recommended sample size and other demographics).

- The online materials might benefit from more examples. For instance, changing the metric (other examples could include looping through the structures):

```
docker run --rm -v $PWD/testout:/OUTPUTS r_lifespan_env python3 /WMLifespan/scripts/output_centile_curves.py AF_left volume /OUTPUTS/centiles_volume_AF_left.csv

docker run --rm -v $PWD/testout:/OUTPUTS r_lifespan_env python3 /WMLifespan/scripts/output_centile_curves.py AF_left fa-mean /OUTPUTS/centiles_FA_AF_left.csv
```

Of note, these two commands run from any location that has the output directory, i.e., the provided model data input file

MS_dataset.csv is not necessary. Making a version with commands where the 'model data' for creating the centiles is given as an input and not used from the Docker container itself would be useful. Having clear pointers in providing input and output is very important.

- Finally, code that was used for this work should be made openly available (as much as possible).

Referee #3

(Remarks to the Author)
Review: White matter microstructure and macrostructure brain charts across the human lifespan, by Kim et al.

This manuscript presents a very comprehensive analysis of diffusion MRI data across numerous datasets spanning birth to 100 years. They use this data to provide normative curves of microstructural (FA, MD, AD, RD) and macrostructural (tract volume, surface area, length) white matter development across 72 different brain white matter tracts. They further provide profiles of the timing of maturation across metrics and tracts. Then, they demonstrate the ability of these curves to help identify deviations of brain structure in a variety of example disorders (MCI, Alzheimer's, epilepsy). Finally, they provide an example using out-of-sample data by aligning it to the calculated curves and demonstrating how this can be used for external data.

This is a monumental effort, combining huge amounts of data from around the world into one sample and providing extensive information on normative white matter changes across the entire lifespan. It provides the most comprehensive analysis of white matter changes to date, I imagine will be a valuable resource for many. The supplied code, particularly that enabling out-of-sample datasets to be incorporated, is hugely useful.

The manuscript is well-written and the figures are excellent. Information is clearly conveyed, and the writing follows a very logical progression. Many important points are addressed well, and analyses clearly described.

I have a few questions/comments for improvement:

1. The authors say that they provide “sex-stratified trajectories”. However, in looking at their equation (methods, equations 2 & 3) and results, it appears that only a main effect of sex was included in the models, and trajectories were not allowed to vary between males and females, e.g., “sex was included as a binary fixed effect”. Is that the case? If so, they are missing huge amounts of information that may be gleaned from modeling male and female trajectories separately, as there are most certainly time periods where the shape of trajectories would differ. I would suggest running fully separate models for males and females to more accurately capture development and aging.
2. Variability is estimated using quantile ranges and said to increase in adulthood. I wonder if this is related, at least somewhat, to data quality in the older samples, rather than true neurobiological variability. This would be worthwhile commenting on, and testing if possible.
3. The examples given for atypical brains are primarily diseases of aging (MCI, Alzheimers), while epilepsy may span a wider age range. Useful other examples are provided in Figure 5D, but I would appreciate some idea of the age range of participants in these samples. Does the centile scoring work across the age range? Does it perform equally well in young vs old participants? Males vs females? It would be really useful to understand how consistent/fair/useful these predictions are across populations.
4. Why was longitudinal data excluded? Many of these datasets contain longitudinal data, and it seems that it would greatly enhance the predictive power of these curves, not only by increasing the total number of datapoints, but also by increasing the power to model individual trajectories. Further, cross-sectional models can underestimate true change: https://www.pnas.org/doi/10.1073/pnas.2216798120
5. The pipelines used for processing here are designed for adults (except infant freesurfer), and can have known issues in infants, young children, or elderly participants. Can the authors please comment on data quality across the lifespan in this dataset?

Minor:
1. Bethlehem et al.‘s brain charts include white matter volume, so statements like “previous efforts did not extend to the study of WM” should be changed. Note that this doesn't negate the importance of this work – I just suggest rewording these statements for accuracy.
2. Introduction, 2nd paragraph, “WM specific traits may be even more critical for clinical studies” – in what way? I'm not sure what this sentence means.
3. Introduction, 3rd paragraph: “…largely due to methodological challenges of dMRI”. I think this does not really follow from the first part of the sentence. There are many ways to quantify WM without dMRI. I would revise this.
4. p. 9, “AD generally peaked later than other features, suggesting prolonged maturation of myelin-sensitive properties” AD is generally associated with axonal change, rather than myelin. Can the authors clarify what they mean here?
5. “scans were excluded if key demographic information – … cognitive status – was missing.” What does this mean and how was it assessed?
6. I noted in the online docker code that out-of-sample data requires at least some typical controls to be used. This is understandable, but would be useful to mention in the paper, since I know there are many researchers out there with clinical datasets looking for readily available control comparisons.

(Remarks on code availability)

I have reviewed the code and the online documentation. I did not install and try running the code due to limitations of the computer I'm currently using. The documentation online is excellent, and provides clear instructions for installing and running the code to complete analysis. It also provides a tutorial dataset, which appears to be a useful resource for those learning how to use this code.

Version 1:

Reviewer comments:

Referee #1

(Remarks to the Author)
Thank you for revising your manuscript thoughtfully. I enjoyed reading it again and have one final comment:

In Figure 6 and its accompanying margin text, the authors state that centile scores are used to predict variability in "physiological, behavioral, or functional measurements". However, the only external validation presented in Figure 6C relates to cognitive performance. Cognition represents a specific subclass of behavioural/functional outcomes and is not equivalent to physiological or functional measures more broadly.

This creates a mismatch between the stated scope and the demonstrated example. The authors are encouraged to clarify the terminology used in the margin text and figure captions, explicitly specify the cognitive measure employed (test name or composite), and temper the generality of the claim unless additional non-cognitive physiological or functional variables are shown. As currently presented, Figure 6C should be framed as an illustrative cognition-specific example rather than evidence for broad physiological or functional prediction.

(Remarks on code availability)

Referee #2

(Remarks to the Author)
The authors have addressed my previous comments thoroughly and effectively. I have a few remaining suggestions that I hope will help further refine the manuscript and are necessary to address.

1. Please ensure that all statistical tests performed are clearly stated, effect sizes are reported, and symbols used in the text and figures are explained. For group comparisons, specify the sample composition and sample sizes for each group (e.g., disease vs. controls). This applies to both the main manuscript and the supplementary materials.

2. The main manuscript should note that sex differences in microstructure are generally less pronounced than in macrostructure, though they do exist. Consider referencing the excellent supplementary analyses that provide detailed insights into this point.

3. Figure 4 and related results: What is the primary conclusion regarding these alternative hypotheses? If both patterns occur frequently, consider reporting this across all measures and brain regions. Additionally, revise the discussion section to replace the phrase, testing the hypotheses, with language that reflects the main conclusions drawn from your analyses.

4. Figure 6: Currently, Figure 6 reads more as an overview of potential applications rather than presenting results. It could serve both purposes. Please revise to present the data in a style consistent with other figures, including details on sample characteristics, statistical tests, and effect sizes. This figure and corresponding text could also highlight that N = 100 is sufficient to estimate centiles for new datasets.

5. The rationale for tract selection is well justified in the response letter. Consider including this logic in the manuscript or figure legends, noting that the displayed tracts are those most frequently used in ROI-based studies. Figures could also mention the classical directional coloring convention (e.g., blue for inferior-to-superior).

6. The improvements to shared code and pipelines are excellent. For completeness, please also share the GAMLSS model code. This could for instance be a script for running GAMLSS on one brain area and several metrics of interest and visualizing the results. Please ensure all links are active. I was unable to access the chart plots across all brain areas.

Minor comments

1. Abstract Wording: The phrase "...35,120 diffusion MRI scans from 50 harmonized studies" may be misleading. Consider rephrasing to "50 studies processed using harmonized methods" or similar.

2. Supplemental Figure SE.F2, consider elaborating on expected differences relative to Zhu et al. The authors define DTI tensor values from central and peripheral regions, which explains the lower FA values, which would be good mention.

3. Supplemental Figure SB.F3: Please clarify whether the FreeSurfer mask is shown as an underlay or overlay (the larger image should be the underlay). Indicate whether the visualization represents a single participant or a summary across

participants.

4. Supplemental Figure SF.F2: Adjust color bars to better reflect differences (e.g., range from -0.5 to 0.5 or lower bounds). Current settings may obscure relevant variance across brain regions and metrics.

5. One key advantage of brain charts is their utility in interpreting associations in applied studies. For example, developmental neuroscience often considers stress acceleration/deceleration hypotheses, while adult studies interpret whether “more of metric X” reflects a healthier phenotype. A brief mention of this benefit might be a nice addition for the manuscript.

(Remarks on code availability)

Referee #3

(Remarks to the Author)
This is a resubmission of a manuscript I previously reviewed, developing and showcasing brain growth charts for white matter diffusion metrics across the lifespan. The work was impressive at first submission, and now the authors have done a remarkable job addressing the reviewers’ comments and concerns, including my own. I believe this paper is very strong.

One additional question I have concerns Figure 4D – where the authors test the ‘last in-first out’ hypothesis. The plot for RD shows that early maturity age (e.g., 10-12y) is associated with early decline ages (e.g., 25-35y). This seems to correspond to a ‘first in-first out’ pattern, rather than a ‘last in-first out’ pattern. In the ‘last in-first out’ pattern, I would expect the latest maturing tracts (e.g., 18y, the 'last') to be the first to decline, providing a relationship that is the inverse of that shown here. This graph and the corresponding results should be better explained if they do indeed support a ‘last in-first out’ hypothesis.

Finally, although I appreciate the linkages with myelin work, and the effort the authors have taken to not equate RD with myelin, I think it would be good to be even more explicit that RD may reflect other changes, e.g., in lines 259-270.

(Remarks on code availability)

Open Access This Peer Review File is licensed under a Creative Commons Attribution 4.0 International License, which permits use, sharing, adaptation, distribution and reproduction in any medium or format, as long as you give appropriate credit to the original author(s) and the source, provide a link to the Creative Commons license, and indicate if changes were made.
In cases where reviewers are anonymous, credit should be given to 'Anonymous Referee' and the source.
The images or other third party material in this Peer Review File are included in the article’s Creative Commons license, unless indicated otherwise in a credit line to the material. If material is not included in the article’s Creative Commons license and your intended use is not permitted by statutory regulation or exceeds the permitted use, you will need to obtain

permission directly from the copyright holder.
To view a copy of this license, visit https://creativecommons.org/licenses/by/4.0/

Reviewer 1

The concept of normative brain charts (alike pediatric growth charts) has recently been extended to brain imaging, offering a valuable framework for quantifying brain structure variability across age and health. The present study provides such reference charts for white matter (WM) based on an impressive cross-sectional dataset (26,199 diffusion MRI scans!). While the present study is a tour de force, my enthusiasms has been tempered by some missing conceptual advances and explorations.

Thank you for the overall optimistic evaluation of this work. We have integrated our findings with classical histological literature and frameworks of both development and degeneration, which we believe has significantly improved the framing of our brain charts as they related to white matter microstructure and integrity. We have additionally performed supplementary sensitivity/specificity analysis and clarified confusions on population median versus population distributions.

R1.C1: The study successfully maps age-related changes in both microstructural and macrostructural white matter properties. These trajectories vary considerably across tracts and features, capturing the heterogeneity of white matter development and degeneration. However, a key conceptual limitation is the lack of integration with long-standing histological data, such as Flechsig's maps of myelin development. Although tract-specific developmental curves are provided, the absence of a unified neuroanatomical framework limits the interpretability of the results. One would expect some theoretical synthesis with classical or contemporary models of myelination and maturation. This paper offers descriptive but not conceptual novelty in this regard.

We thank the reviewer for raising this important point. We agree that integrating our findings with a strong neuroanatomical framework significantly enhances the conceptual novelty. To address this (and R1.C2, related to incorporating biological insight), we have substantially revised the "White Matter Developmental Milestones and Patterns of Myelination" section, with a focus on Figure 4:

- Integration with classical histology: By interrogating one of our measures of microstructure (radial diffusivity, as an in vivo proxy for myelination), we link our non-linear, tract-specific trajectories to the foundational models of myelin development from Flechsig (as quantified/summarized in a recent Nature Neuroscience manuscript from deFaria et al., 2021). This analysis, visualized in Figure 4B and 4C, demonstrates that our in vivo charts recapitulate these classical, histologically-defined patterns, but more importantly provide detailed non-invasive measures across dozens of white matter pathways, summarized over 35,000+ individuals, and detailing waves of myelination across the entire lifespan.

- Testing neurobiological hypotheses: We leverage the tract-specific inflexion points and non-linear dynamics (noted as a forte in R1.C2) to show use of the charts to test neurobiological principles of development and aging, including the retrogenesis principle of brain development/aging by testing the "last-in-first-out" and "gain-predicts-loss" hypotheses (Figure 4D).

- Biological interpretation: We have enriched the biological interpretation by situating our developmental milestones alongside diagnostic age ranges, contemporary transcriptomic developmental maps, and visualizing the spatial gradients of maturation (infancy) and decline (aging) across the brain (Figure 4A).

We believe these major revisions provide the integration and linkage to the unified neuroanatomical framework and demonstrate that our brain charts are not just descriptive but serve as a tool for interrogating the fundamental principles of (normal and abnormal) white matter maturation and aging.

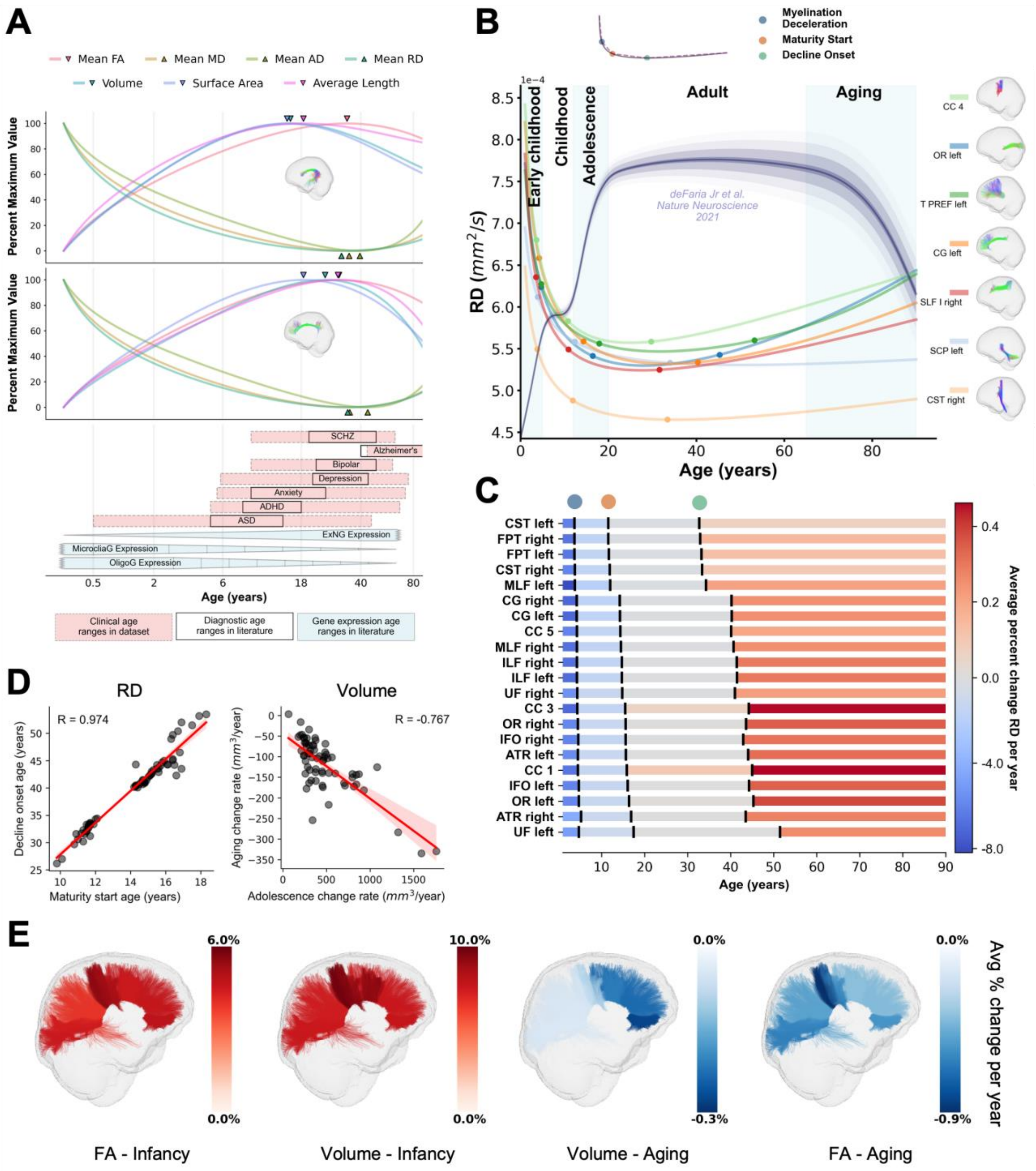
A
Mean FA
Mean MD
Mean AD
Mean RD
Volume
Surface Area
Average Length
Percent Maximum Value
SCHZ
Alzheimer's
Bipolar
Depression
Anxiety
ADHD
ASD
ExNG Expression
MicrogliaG Expression
OligoG Expression
Age (years)
Clinical age ranges in dataset
Diagnostic age ranges in literature
Gene expression age ranges in literature
B
Myelination Deceleration
Maturity Start
Decline Onset
Early childhood
Childhood
Adolescence
Adult
Aging
deFaria Jr et al. Nature Neuroscience 2021
RD (mm^2/s)
Age (years)
CC 4
OR left
T PREF left
CG left
SLF I right
SCP left
CST right
C
CST left
FPT right
FPT left
CST right
MLF left
CG right
CG left
CC 5
MLF right
ILF right
ILF left
UF right
CC 3
OR right
IFO right
ATR left
CC 1
IFO left
OR left
ATR right
UF left
Age (years)
Average percent change RD per year
D
RD
R = 0.974
Decline onset age (years)
Maturity start age (years)
Volume
R = -0.767
Aging change rate (mm^3/year)
Adolescence change rate (mm^3/year)
E
6.0%
0.0%
10.0%
0.0%
0.0%
-0.3%
0.0%
-0.9%
Avg % change per year
FA - Infancy
Volume - Infancy
Volume - Aging
FA - Aging

Figure 4. Tract-specific Developmental Milestones, Neurobiological Patterns, and Spatial Gradients. Lifespan trajectories reveal tract-specific developmental milestones and allow for investigation into neurobiological hypotheses and spatio-temporal dynamics of brain microstructure. A.) Median centile trajectories for all features of the left arcuate fasciculus (top) and the right cingulum (middle) show pathway-specific developmental maturation as peaks (downward triangles) or troughs (upward triangles). Below, a graphical summary situates these trajectories alongside non-MRI milestones. Pink shaded boxes denote the age range of clinical disorders [4] represented in the dataset; black boxes denote diagnostic age ranges from literature; blue shaded boxes indicate transcriptomic development windows [5]. B.) Radial diffusivity (RD) median trajectories for exemplar tracts illustrate waves of development and late-life change. These in vivo patterns are consistent with proposed myelin development and aging patterns [3] based on foundational histological work from Yakovlev and Lecours [1] (myelin changes qualitatively visualized as blue curve reproduced from deFaria et al. [3]) C.) Relative timing and magnitude of change for RD trajectories across selected tracts. Vertical black lines represent the identified myelination timepoints (e.g., rapid early myelin increase, maturity start, decline onset). The color of the bars represents the average percent change per year during different life epochs (darker blue indicates a faster decrease; darker red indicates a faster increase), revealing tract-specific variability in rates of change. D.) Neurobiological hypothesis tests derived from tract milestones and rates. Left: last-in-first-out - tracts that mature later tend to show earlier onset of decline (age at maturity vs. age at decline, RD). Right: gain-predicts-loss - greater adolescent growth in volume predicts steeper decline with aging (slope–slope relation). E.) Spatial organization: anterior-posterior gradients emerge in rates of change across tracts (both microstructure and macrostructure), indicating regionally patterned maturation and decline.

R1.C2: The identification of tract-specific inflexion points and non-linear dynamics is definitely a forte of this work. Yet again, the lack of conceptual linkage to known patterns of myelin maturation reduces the depth of interpretation. Incorporating insights from Flechsig, Yakovlev, and Lecours, or even contemporary transcriptomic developmental maps, would have enriched the biological interpretation.

This, again, is an excellent point. See R1.C1 for detailed revisions. Briefly, we now integrate with both temporal (Figure 4B, D) and spatial patterns (Figure 4E) of myelination (in addition to clinical diagnostic ages, and gene expression ages – Figure 4A).

R1.C3: The facilitating of standardised scoring of new datasets is perhaps the most directly useful aspect of the paper. The availability of a harmonised reference enables future studies to score individuals or populations. This contribution is methodological and practical, and it lays the groundwork for future clinical tools. However, the authors propose the clinical relevance of these charts by projecting patient groups (e.g., with MS, schizophrenia) onto the normative curves and identifying deviations. While group-level deviations are statistically significant, no specificity/sensitivity analyses or ROC curves are provided to evaluate the clinical utility of this approach for diagnosis or prognosis. A formal assessment of accuracy (e.g., AUC, predictive validity) is essential to support claims of clinical translatability.

We thank the reviewer for this important comment and agree with their assessment. Our primary contribution is indeed methodological: to provide a foundational, harmonized reference that lays the groundwork for future clinical tools.

Our goal with the case-control analysis (Figure 5) was to demonstrate the utility and broad applicability of the centile-scoring framework for quantifying atypicality across diverse diagnostic groups. This approach is consistent with prior foundational brain chart papers, such as Bethlehem et al. (Nature, 2022), which also used case-control cohorts to demonstrate the charts' utility. As this is a resource-development paper, a deep investigation into the specific pathophysiology or diagnostic utility for any single disorder is beyond our intended scope.

However, to directly address the reviewer's point on providing a more formal assessment of clinical potential, we have added a new predictive analysis to the supplementary information. In this classification experiment, the centile scores achieved high accuracy (for example, AD classification AUC = 0.910+/- 0.018). This new analysis provides quantitative evidence supporting the potential for these charts to be translated into clinical tools.

Finally, we have added a specific caveat to our Discussion, inspired by similar statements in Bethlehem et al., to clarify that these charts are a foundational research tool and are not yet validated for individual patient diagnosis in a clinical setting.

Finally, we caution that the analogy to pediatric growth charts does not imply that these WM brain charts are immediately suitable for the quantitative diagnosis of individual patients in a clinical setting. Significant caveats exist even for the diagnostic interpretation of traditional anthropometric growth charts.[6] Similarly, considerable future research and validation will be necessary to translate these white matter charts from a foundational research framework into a clinically validated diagnostic tool.

The supplementary predictive analysis:

Anomaly Detection

To assess the utility of our brain charts for performing predictive analyses on clinical cohorts, we performed a classification task to distinguish participants with Alzheimer's disease and mild cognitive impairment (MCI) from typically developing and aging individuals. For the classification, we used centile scores for the microstructural features of FA, MD, AD, and RD and for the macrostructural features of volume, surface area, and average length for all 72 tracts (504 total centile score features). A multilayer perceptron (MLP) with two hidden layers was trained on the 504-dimensional input, using ReLU activation between layers, class-weighted Cross Entropy as a loss function, and the Adam optimizer with a learning rate of 0.001. The network was trained with 5-fold cross validation, with classes evenly split across folds. For individuals whose scans did not reconstruct all 72 tracts in the processing pipeline, the miceforest algorithm was used for data imputation (https://github.com/AnotherSamWilson/miceforest). We report area under the receiver operating characteristic curve (ROC-AUC) and balanced accuracy in Suppplemental Tables SG.T1 and SG.T2. The 0.910 +/- 0.910 and 0.829 +/- 0.015 average AUCs across folds indicate promising discriminative performance; however, we still advocate that the utility of these brain charts should be as research tools rather than clinical diagnostic tools.

Supplemental Table SG.T1. Results of Alzheimer's disease classification experiment.

Fold	Balanced Accuracy	ROC-AUC
**Fold 0**	0.810	0.888

**Fold 1**	0.807	0.901
**Fold 2**	0.820	0.907
**Fold 3**	0.866	0.937
**Fold 4**	0.789	0.916
**Average**	**0.819 +/- 0.029**	**0.910 +/- 0.018**

Supplemental Table SG.T2. Results of mild cognitive impairment classification experiment.

Fold	Balanced Accuracy	ROC-AUC
**Fold 0**	0.746	0.851
**Fold 1**	0.721	0.818
**Fold 2**	0.721	0.812
**Fold 3**	0.737	0.833
**Fold 4**	0.712	0.832
**Average**	**0.728 +/- 0.014**	**0.829 +/- 0.015**

R1.C4: The authors state that "white matter is drastically more heritable than grey matter traits," referencing large-scale genetic studies. However, the cited paper (likely Smith et al., 2021, Nat Neurosci) does not support this as a categorical or generalised statement. While some WM traits (e.g., FA in certain tracts) show higher heritability than some GM traits, the variation is considerable, and no single modality dominates across the board. Additionally, these findings were not tract-specific and may be confounded by cortical folding variability and registration noise. The authors should temper this claim accordingly.

We thank the reviewer for this clarification. We agree that the original statement was an over-generalization of complex heritability findings. Since a detailed genetic analysis is not the primary focus of our manuscript, which is centered on the development and application of normative models, we have simply removed this sentence to avoid misinterpretation and maintain focus on our central findings.

R1.C5: The data is cross-sectional, not longitudinal, which is important as cross-sectional trends can mask within-subject variability or cohort effects. This limitation should be acknowledged more explicitly.

We agree with the reviewer that this is an important limitation. We have now more explicitly acknowledged in the Discussion that our normative trajectories are derived from cross-sectional data. As recent work (Di Biase et al., 2023) has shown, individual longitudinal trajectories can differ from population-level cross-sectional trends.

We adopted the cross-sectional approach for several reasons, including maximizing statistical power, ensuring model stability, and maintaining consistency with the foundational Bethlehem et al. framework, which facilitates out-of-sample alignment. We have added a detailed justification for this methodological choice in the supplementary material (see "Considerations for Cross-sectional vs Longitudinal Brain Charts") and highlight longitudinal validation as a critical avenue for future work.

Text added to limitations includes:

> Further, normative trajectories were derived from cross-sectional data; future longitudinal validation will be important, as individual trajectories can differ from population-level trends.[7] Our cross-sectional approach was chosen to ensure statistical power and model stability for out-of-sample alignment, as detailed in the Supplementary Information ("Considerations for Cross-sectional vs Longitudinal Brain Charts")

And we add the following section to the supplementary material:

> Considerations for Cross-sectional vs Longitudinal Brain Charts
>
> Previous work has shown that cross-sectional normative models can be inaccurate in predicting measured longitudinal trajectories[7], as longitudinal models can better separate true developmental change from generational differences that exist in the data and cross-sectional data may be unrepresentative of individuals. Although many of our datasets contained longitudinal data, there were several challenges we encountered and aspects about the normative models we needed to consider for longitudinal brain charts. First, despite the subset of datasets with participant data at multiple time points, for the majority of participants in the study we only have cross-sectional data available. Incorporating longitudinal data would require estimation of random effects for each participant, introducing significant computational complexity in the model. We found that we did not have enough data points to stably estimate individual-level random effects. Furthermore, having to estimate these individual-level random effects would greatly decrease the stability and increase complexity of the out-of-sample centile scoring process, as alignment with N participants would require estimation of $2N$ terms instead of only 2 terms with the cross-sectional models. These constraints and considerations led us to develop a robust cross-sectional approach that maintains generalizability and computational feasibility. We acknowledge the potential benefits of longitudinal modeling and suggest this as an important avenue for future research.

R1.C6: 7) The charts assume a normative "median" trajectory, but brain organisation varies across individuals. Studies such as Catani et al. (2007) have shown structural connectivity variants in the general population (e.g., absence of arcuate fasciculus in some individuals). Relying solely on median-based norms could misclassify healthy phenotypic variants as outliers. The lack of phenotypic subtyping (beyond sex) is a limitation that should be addressed in future work.

We thank the reviewer for this thoughtful point, as it allows us to clarify a key methodological strength of our study. The reviewer is correct that relying solely on a median trajectory would be a significant limitation and would risk misclassifying healthy variants.

However, our charts are not median-based norms. We specifically chose Generalized Additive Models for Location, Scale, and Shape (GAMLSS) to avoid this very problem. This framework explicitly models the entire population distribution - not just its location (median/mean), but also its scale (variance) and shape (skew). Therefore, our model is designed to capture this exact inter-individual variability, allowing each individual to be benchmarked with a centile score relative to this full distribution. A healthy but rare variant would simply receive an extreme centile (e.g., 2nd or 98th), which correctly quantifies its statistical atypicality without making a clinical "pathological" judgment. As we now clarify in the Discussion, these charts are a foundational tool and not intended for immediate, standalone clinical diagnosis.

We have taken this opportunity to highlight the advantage of capturing inter-individual variability with small additions throughout the introduction, results, and discussion.

Regarding the second point, we agree that the lack of phenotypic subtyping (beyond sex) is a limitation. Incorporating variables such as APOE status or race/ethnicity is a critical goal for future work. We did not include them in the current reference model because this information is not available or consistently reported across all 50 cohorts. We now state this explicitly as a limitation:

> To maintain the statistical power of our normative reference model we did not include phenotypes beyond sex (race/ethnicity, genotype status, etc.) due to limited data availability and reduced sample size (see Supplemental Table SB.T2)

R1.C7: Minor Comments: There are typos in Reference 3 that should be corrected.

We have thoroughly reviewed the references to correct typos and ensure consistency.

R1.C8: This study presents he first large-scale WM reference charts, which could become a valuable resource for the neuroimaging community. However, the paper could benefit from a stronger neuroanatomical foundation and clearer clinical validation metrics. Additionally, the assumption of a unitary "norm" may obscure biologically valid inter-individual diversity and personalised medicine, which are becoming a pressing clinical matter.

We thank the reviewer for this concise summary of the key points. We have performed major revisions to address all three concerns:

1. Stronger Neuroanatomical Foundation: As detailed in R1.C1 & R1.C2, we have substantially revised Figure 4 and the corresponding results section to integrate our charts with classical histological frameworks (Flechsig, Yakovlev, Lecours) and to test major neurobiological hypotheses (LIFO, gain-predicts-loss).

2. Clearer Clinical Validation: As in R1.C3, we have refocused our case-control analysis on 7 major diagnostic groups (per Bethlehem et al.) and provided a new supplementary classification analysis (AUC=0.910 for AD) as a formal assessment of the charts' potential clinical utility. We aim to show broad applicability of our charts, and additionally acknowledge that future research and validation will be necessary to translate these white matter charts from a foundational research framework into a clinically validated diagnostic tool

3. Unitary Norm vs. Inter-individual Diversity: We strongly agree that assuming a unitary 'norm' (e.g., a mean/median) would obscure biologically valid diversity. This is precisely the limitation of simpler models that our study was designed to overcome. Our approach models the full population distribution. We have clarified this in two key places in the manuscript:

In the Discussion, we now explicitly state:

> Crucially, our GAMLSS framework moves beyond a single median trajectory and explicitly models the full population distribution (including its location, scale, and shape), allowing inter-individual variability itself to be quantified.

In the Introduction, inspired by the reviewer's comment and the logic of established WHO/CDC growth charts, we now add the justification:

> Analogous to the WHO and CDC pediatric growth charts, defining the standards of healthy maturation is the necessary prerequisite for identifying and quantifying atypical development and decline.

We believe these additions, combined with our other revisions, fully satisfy the points raised.

Reviewer 2

Michael E. Kim and colleagues present an important update to available "brain growth charts." They have used more than 26k MRI scans from 42 studies to yield derived brain measures of gross white matter anatomy in terms of tract morphometry (macrostructure) and across commonly used diffusion tensors (microstructure). Overall, this work is ambitious and represents a tangible advancement in describing the dynamic changes of brain structure across the human lifespan. This allows defining normative trajectories, identifying milestones in the trajectories, discovering pathology-related changes, and creatingtools to create centile scores for external samples. The work is likely to be highly interesting to a wide audience, including neuroscientists and other related disciplines. The choice of brain measures is the key strength of the manuscript, and it has the potential to bridge the volumetric and diffusivity brain centiles. I think this manuscript is original and sufficiently distinct from prior work. My conclusion is that with (admittedly substantial) additional work, this manuscript can be a highly relevant contribution by 1) serving as a benchmark work for the creation of national brain charts that are 'validated' in international samples, 2) providing a bridging effort between existing volumetric brain charts and DTI brain charts, and 3) creating standard protocols for tractography-based region of interest delineation to serve applied basic and clinical studies. My concerns about the current version of the manuscript are outlined below.

We thank the reviewer for their positive response and thoughtful feedback. The points raised below are excellent considerations, and gave us the opportunity to increase data size, but also integrate considerations on sample populations as they relate to our own work, and to the more common CDC and WHO pediatric charts. We have additionally included several new experiments/analysis that highlight influence of datasets, model variability, image preprocessing considerations, and replication of prior works.

R2.C1: Given that the datasets of the current study are almost exclusively from the United States, I would suggest a rephrasing of the manuscript to brain charts of the North American population that could be 'tested/validated' by centile scoring of data sets from other countries (UK, Netherlands, etc.). This would not undermine the value of the work, in my opinion. Looking forward, it is highly likely that national brain growth charts are used jointly with international charts for assessing each population, similar to the growth charts of body growth charts that are in routine clinical use.
Reference: Cole TJ. The development of growth references and growth charts. Ann Hum Biol. 2012 Sep;39(5):382-94. doi: 10.3109/03014460.2012.694475. Epub 2012 Jul 11. PMID: 22780429; PMCID: PMC3920659.

Could the authors claim national representativeness of the US population? Would it be possible to increase the available data by increasing the coverage across different US states?

On a related note, the study is also missing a few notable data sets. Why is the ABCD study missing from the used data sets? It would be a key to increasing the national representativeness. Why do the authors only include ca. 10,000 UK Biobank participants when the available high-quality DTI data includes in excess of 57k scans, and depending on criteria, the amount of 'healthy' participants is ca. 30k scans? Why is the developing human connectome (dHCP) project data missing from the used data sets?

We thank the reviewer for these comments regarding our dataset and sample populations, which we have addressed with a major revision of our cohort and additional considerations and limitations.

First, we agree that geographic composition matters. Our revised reference cohort remains predominantly North American, but now includes multiple non-US datasets spanning infancy through adulthood (e.g., dHCP in the UK, AOMIC in the Netherlands, QTAB/Age-ility in Australia, and SWU in China). We therefore do not rebrand as “North American charts,” but we now state this imbalance explicitly as a limitation and provide a table listing dataset locations to increase transparency (Supplemental Table SB.T3). We also emphasize that our framework supports out-of-sample (OOS) centile scoring with local control calibration, which is the practical path to validation in additional countries.

In our main text, we now state:

> Although there was data representation from several continents, the datasets used to create the brain charts are predominantly from European and North American populations (see Supplemental Table SB.T3). This bias is unfortunately common in neuroimaging and genetics, echoing limitations in even foundational clinical references like the CDC and WHO growth charts.[8] This highlights a critical need for the global scientific community to increase ethnic, socioeconomic, and demographic diversity in future MRI research.

We similarly advocate “it is highly likely that national brain growth charts are used jointly with international charts for assessing each population, similar to the growth charts of body growth charts that are in routine clinical use.” and briefly note similarities to the WHO and CDC charts which are based on only 6 countries (WHO), or remarkably, only 2 states (Wisconsin and Missouri) for the CDC length charts.

Second, we agree with the reviewer that having such a level of identifiability would increase the usefulness of our brain charts. However, due to participant privacy concerns, many datasets included do not release such information with their data. Additionally, for datasets where this information is obtainable, releasing such information would increase identifiability of participants, which would violate the terms of data use agreements we have in place. However, we have added Supplemental Table SB.T3, which contains the source location of each dataset used in this manuscript and increases transparency about the populations used in creating these reference curves. Further, large research MRI datasets are typically acquired retrospectively, limiting our ability to obtain participants from select regions. Although we cannot guarantee an increase in representational coverage, we include the additional datasets of ABCD, HBCD, dHCP, EBDT, BSNIP1, BSNIP2, VUMC-ASD, and Ageility to increase the total number of typically developing and aging participants used in this study to 35,120.

Regarding datasets: We have now incorporated ABCD and dHCP in the revision, and added HBCD, EBDT, BSNIP1/2, VUMC-ASD, and Age-ility, increasing the reference population to 35,120 participants across 50 studies.

Our UK Biobank contribution is 10,000 participants due to current data-use permissions. While we plan to include more in future iterations, we also note that limiting the UKB contribution prevents a single dataset from heavily biasing the reference population. For context, our inclusion of 10,000 UKB participants (out of 35,120 total) is comparable to Zhu et al., who included 5,000 UKB participants in their reference cohort of 13,297.

R2.C2: The brain charts for the chosen brain measures are original and increase the impact of the work. The authors used the largest amount of individual studies to build their source data but have a lower sample size than in prior work reporting the creation of DTI brain charts.
- Zhu et al. 2025: N = 40,898 - 10 studies
- Villalón-Reina et al. (preprint): N = 54,583 - 19 studies
- Kim et al. (manuscript): N=26,199 - 42 studies.

I would ask the authors to elaborate on how they justify that their sample is sufficient in terms of sample size and sampling strategy based on prior work (running appropriate simulations would be ideal).
Reference: Bozek J, Griffanti L, Lau S, Jenkinson M. Normative models for neuroimaging markers: Impact of model selection, sample size, and evaluation criteria. Neuroimage. 2023 Mar;268:119864. doi: 10.1016/j.neuroimage.2023.119864. Epub 2023 Jan 5. PMID: 36621581; PMCID: PMC11636675.

We thank the reviewer for placing our work within the context of other recent studies and for raising this important point about sample size. We have addressed this in three ways: first, is increased sample size. As mentioned in our response to R2.C1/C3, we have substantially expanded our dataset. We incorporated 8 additional cohorts, including the major ABCD, HBCD, and dHCP studies. This increases our reference population by nearly 50% to 35,120 participants from 50 studies, making it the largest and most diverse tractography-based lifespan cohort to date.

Second is sample composition. We agree that a simple N-count can be misleading. As the reviewer notes, our study includes a far more diverse collection of cohorts (50) than the other papers. The Zhu et al. abstract mentions ~40k participants, but as their paper states, only 13,297 were used to build the actual reference model. The Villalón-Reina et al. preprint, while having a large N, is heavily biased toward a single dataset, with over 37,000 of its 54,583 participants (67%) coming from the UK Biobank. Our study's strength lies in its dataset diversity, which provides a more robust and generalizable reference by sampling across a wider range of scanners and populations. We have added Supplemental Table SE.T3 (below) to highlight these methodological differences.

Third, theoretical Justification and Model Stability. Our sample size of N=35,120 is strongly supported by the established theoretical framework for constructing growth reference centiles. A reference provided by the reviewer, Cole (2021), analyzes the sample size and composition required for stable centile models. While optimal conditions might vary by phenotype and population, Cole suggests these modeling strategies require 7,000–25,000 subjects per sex. Our N of 35,120 (roughly 17.5k per sex) falls squarely within this theoretically-justified optimal range (which should give a roughly equivalent contrast-per-age-range as the more focused development charts).

As confirmatory evidence of our model's stability and the sufficiency of our sample (as recommended by Cole), we performed a leave-one-study-out (LOSO) bootstrapping analysis (see Supplemental Figure SD.F3). The results show a coefficient of variation (COV) on average below 0.5% across the vast majority of the lifespan (for all features/pathways; with few exceptions, particularly at extreme ages), demonstrating that our models are highly stable and not unduly influenced by any single cohort. This robust stability is in line with the comprehensive validation approach of Bethlehem et al., which we detail further in our response to R2.C7.

Supplemental Table SE.T3. Comparison of our WM brain charts to existing microstructural brain charts in the literature.

Study	Age Range	Datasets	Sample Size for Brain Charts	Total Sample Size (including validation)	Preprocessing	Normative modeling	Features {total number}
Zhu et al. 2025 [9]	3-95	10	13,297	40,898	EPI- and eddy-current distortion correction; TBSS with JHU labels[10]	GAM + ComBat variations	FA {25}
Villalón-Reina et al. (preprint) [11]	4-91	19	54,583	54,583	EPI- and eddy-current distortion correction; TBSS with JHU labels[10]	HBR	FA, MD, AD, RD {88}
Our work	0-100	50	35,120	41,897	DWI Preprocessing (PreQual); DTI Fitting (MRtrix); Tractography (TractSeg); Whole-brain White Matter Segmentation (FreeSurfer)	GAMLSS	FA, MD, AD, RD, Volume, Surface Area, Average Length, Normalized Macrostructure {1157}

* For Villalón-Reina et al. and Zhu et al., we note that preprocessing was inconsistent (ENIGMA has different tensor fitting algorithms)

R2.C3: There are important similarities and differences in DTI brain chart reports that would be important to summarize. I would ask the authors to provide a table in the supplementary materials that compares their work to other closely related work in terms of covered age range, sample characteristics, preprocessing, derived brain metrics, and tools to build the reference charts, etc., in comparison to references Zhu et al. 2025 & Villalón-Reina et al. (preprint).

We certainly agree with the reviewer that such a comparison would be important. To that end, we include the following tables in the supplementary material that summarize previous white matter lifespan studies, making the distinction between the literature studying white matter across the lifespan (typically limited to population-mean values or linear associations) and those that develop charting/references (Zhu et al. and Villalón-Reina et al.):

Supplemental Table SE.T1. Other research modeling white matter across the lifespan.

Study	Age Range (years)	Participants	Datasets	Microstructure	Macrostructure	White Matter Info
Storsve et al. 2016 [12]	23-87	201 (402 sessions)	1	DTI	N/A	TRACULA (18 tracts)
Giorgio et al. 2010 [13]	23-81	66	1	DTI	N/A	WM Voxels
Beck et al. 2021 [14]	18-94	573 (702 sessions)	2	DTI, NODDI, DKI, RSI, WMTI, SMT mc: 6 models, 20 scalars	N/A	TBSS skeleton
Henriques et al. 2023 [15]	18-88	636	1	DTI, DKI, NODDI	N/A	JHU Atlas (48 ROIs)
Toschi et al., 2020 [16]	13-62	91	1	DTI, CHARMED	N/A	TBSS skeleton + JHU overlap (50 ROIs)
Lebel et al. 2012 [17]	5-83	403	1	DTI	WM Volume	12 pathways (Manual)
Slater et al. 2019 [18]	7-84	801	1	DTI, NODDI, g-ratio	N/A	20 bilateral + callosal tracts (AFQ)
Yeatman et al. 2014 [19]	7-85	102	1	DTI (FA,MD only)	N/A	24 pathways (AFQ)
Groves et al. 2012 [20]	8-85	484	2	DTI (FA,MD,MO)	N/A	TBSS skeleton + ICA
Hoagey et al. 2019 [21]	20-94	186	1	DTI (FA,MD)	N/A	Population-based WM Voxels
Kochunov et al. 2011 [22]	11-90	1031	1	DTI (FA only)	N/A	TBSS skeleton + JHU overlap (11 ROIs)
Bender et al. 2015 [23]	19-78	96 (192 sessions)	1 (2 timepoints)	DTI (FA,AD,RD)	N/A	TBSS skeleton + JHU overlap (13 ROIs)

Sexton et al. 2014 [24]	20-84	203 (406 sessions)	1 (2 timepoints)	DTI	N/A	TBSS skeleton + manual ROIs (4 ROIs)
Sala et al. 2012 [25]	13-70	84	1	DTI	Tract volume	8 ROIs from FA atlas
Westlye et al. 2010 [26]	8-85	430	2	DTI	WM Volume	TBSS skeleton + JHU overlap (16 ROIs)
Billiet et al. 2015 [27]	17-70	59	1	DTI, NODDI, DKI, MET2	N/A	Voxel-wise in WM mask; also 17 JHU ROIs
Molloy et al. 2021 [28]	18-75	79	1	DTI	N/A	TBSS skeleton + JHU overlap (20 ROIs)
Ardekani et al. 2007 [29]	26-69	20	1	DTI	N/A	6 Manually Traced ROIs
Schilling et al., 2023 [30]	0-100	2789	4	DTI,NODDI	Tract volumes, lengths, areas, etc.	63 TractSeg ROIs
Conte et al. 2024 [31]	2wk-100yrs	6186	12	FA,MD	WM Volume; Tract Density	XTRACT (29 tracts)

Supplemental Table SE.T3. Comparison of methods to other microstructure normative modeling.

Study	Age Range	Datasets	Sample Size for Brain Charts	Total Sample Size (including validation)	Preprocessing	Normative modeling	Features {total number}
Zhu et al. 2025 [9]	3-95	10	13,297	40,898	EPI- and eddy-current distortion correction; TBSS with JHU labels[10]	GAM + ComBat variations	FA {25}
Villalón-Reina et al. (preprint) [11]	4-91	19	54,583	54,583	EPI- and eddy-current distortion correction; TBSS with JHU labels[10]	HBR	FA, MD, AD, RD {88}
Our work	0-100	50	35,120	41,897	DWI Preprocessing (PreQual); DTI Fitting (MRtrix); Tractography (TractSeg); Whole-brain White Matter Segmentation (FreeSurfer)	GAMLSS	FA, MD, AD, RD, Volume, Surface Area, Average Length, Normalized Macrostructure {1157}

* For Villalón-Reina et al. and Zhu et al., we note that preprocessing was inconsistent (ENIGMA has different tensor fitting algorithms)

R2.C4: Brain atlases vary from study to study, and some atlases have variable labels for identical 'white matter tracts.' I would ask the authors to provide an atlas comparison to Zhu et al. 2025, which used the ENIGMA consortium procedures and JHU white matter atlas to define the ROIs. This could be achieved, for instance, through reporting overlaps for the regions of interest in MNI space (TractSeg atlas vs. JHU atlas labels).

We agree that cross-framework interpretability is important. However, a direct overlap analysis (e.g., in MNI space) would be misleading because the two methods are quantifying fundamentally different things – which we believe highlight two strengths of our study: (1) studying white matter "pathways" instead of simple "regions", and (2) subject specific analysis (i.e. tractography) rather than population-atlas analysis:

The JHU/ENIGMA approach, used by Zhu et al., is a regional atlas-based method. It uses a predefined atlas, often combined with a white matter skeleton (TBSS), to extract metrics from static, non-overlapping regions in a common space. While these regions are named after tracts (e.g., "Superior Longitudinal Fasciculus"), they do not represent the full, continuous pathway but rather a limited portion of it.

Our approach uses TractSeg as a subject-specific tractography tool. This reconstructs 72 complete white matter pathways based on the diffusion signal in each individual brain. This method is (1) subject-specific and (2) pathway-based, not regional. A key advantage is that it correctly models anatomical reality, such as the spatial overlap of different tracts [32,33], which atlas-based regional methods do not permit.

Given this "pathway vs. region" distinction, a direct overlap comparison may be misleading. Regardless, we have added a new section to the supplementary material ("Comparison to Existing Reference Charts"). This section includes a detailed explanation and a new figure (Supplemental Figure SE.F3) that visually contrasts the limited regional coverage of the JHU atlas labels against the complete pathway reconstructions used in our study

Finally, [31,32]we do acknowledge that tractography is dependent on definitions and protocols used to derive pathways[34]; however, this issue is not unique to our process and is a fundamental limitation of all neuroimaging in the literature. [35]

To address these points, we have added the section "Comparison to Existing Reference Charts to the supplementary materials, which explains these differences and provides example visualizations that highlight differences.

Comparison to Existing Reference Charts

…

The FA brain charts created by Zhu et al. use the preprocessing pipeline defined by the ENIGMA consortium[10] to obtain quantitative values of microstructure within regions of interest (ROIs) defined by the JHU DTI. While these atlas-based approaches are commonly used methods for quantifying microstructure within specific brain regions, we note that such practices are not subject specific. Furthermore, these methods do not permit assignment of a single voxel to multiple different tracts, resulting in ROIs that do not completely represent complete white matter pathways (Supplemental Figure SE.F3). In our approach, even though TractSeg predefines 72 white matter tracts, the approach is tractography-based, allowing us to study subject-specific white matter pathways derived directly from an individuals' dMRI data. This is an especially important aspect of our methodology, as white matter pathways are known to overlap in space[32,33], and tractography-based methods allow assignment of a single voxel to multiple different tracts. In this regard, we are the first to use WM tracts/pathways for constructing WM brain charts, as we are not using the atlas-based methods used in other research papers.

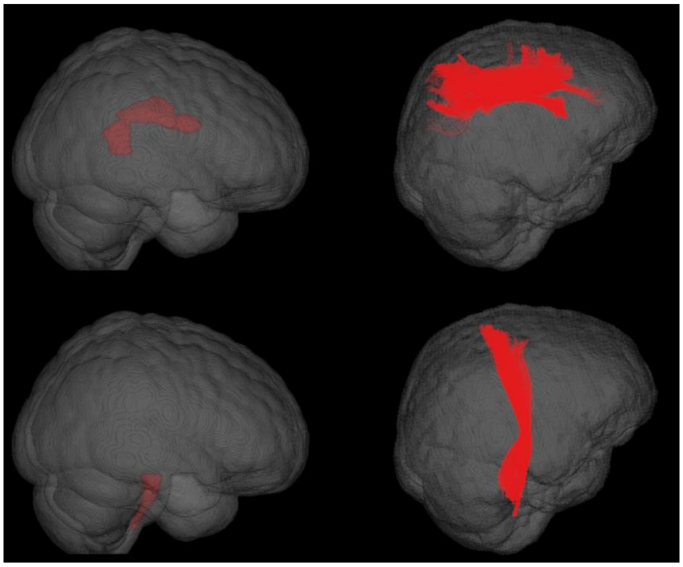

Supplemental Figure SE.F3. Comparison to Zhu et al. for tract specific white matter coverage. Zhu et al.'s method for tract-specific white matter coverage is based on the JHU atlas labels (left), whereas our method is based on subject-specific tractography using TractSeg (right). Notably, atlas-based approaches result in fundamentally spatially separated regions that do not constitute full pathways. Visualized are the left superior longitudinal fascicle (top) and the left corticospinal tract (bottom).

R2.C5: The authors state that they were successful in "identifying previously uncharacterized developmental milestones and spatial gradients of tract maturation." However, the developmental milestones are hard to verify from the current version of the manuscript due to differential plotting of age in comparison to prior work (which can be easily fixed by adjusting the plots). This would be especially relevant for plots reported in Zhu et al. 2025 & Villalón-Reina et al. (preprint), and the authors could focus on the ROIs that have overlaps with JHU atlas labels.

As in R2.C4, we note there are fundamental differences in what is being quantified. We focus on pathway-specific analysis, whereas Zhu et al. use ROIs. Additionally, they only capture features (FA, for example) along the white matter skeleton, which by definition are the highest FA values within the white matter (i.e. their "global white matter" is only a small fraction of the highest FA voxels). While there are advantages and disadvantages to this, we prefer the specificity offered by characterizing the entire trajectory of functionally relevant white matter pathways, tracts, or bundles.

As the models from Villalón-Reina et al. are not released, we can only directly compare trajectories from the Zhu et al. paper (Supplemental Figure SE.F2), and visualized differences between tracts and brain regions (Supplemental Figures SE.F3, SE.F4).

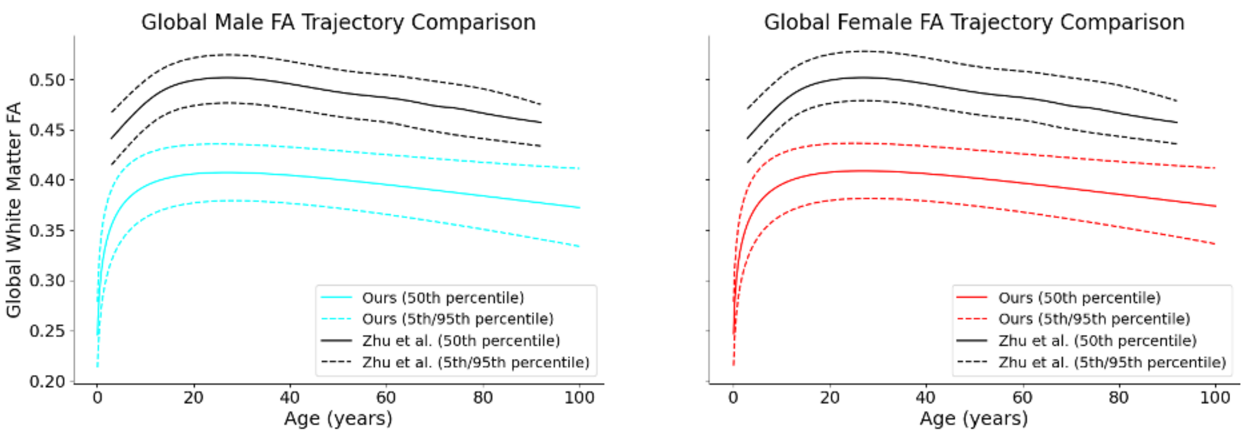

Supplemental Figure SE.F2. Comparison of lifespan trajectory to Zhu et al. Our trajectory follows the same general trend as Zhu et al., with a sharp increase in adolescence, followed by a plateau in early adulthood, and then a decline in aging. Noticeably, the trajectories from Zhu et al. sit at higher FA values, due to the use of phenotype extraction along only the TBSS-defined white matter skeleton (Supplemental Figure SE.F4).

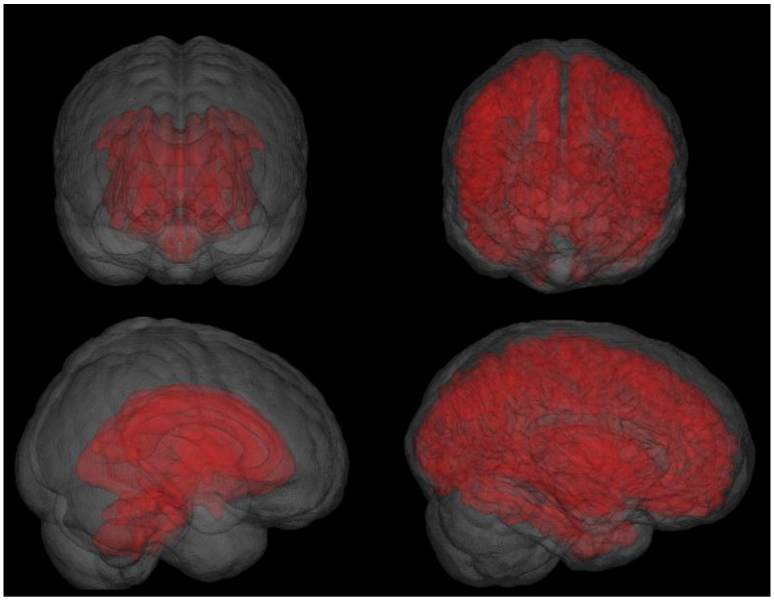

Supplemental Figure SE.F4. Comparison to Zhu et al. white matter coverage for global microstructure. Zhu et al.'s method utilizes the combined JHU atlas labels (left) to define white matter coverage for assessing global microstructure. In contrast, our method uses the general white matter mask derived from FreeSurfer (right). The FreeSurfer mask provides broader overall white matter coverage compared to the JHU atlas, which encompasses only specific white matter tracts. Furthermore, the FreeSurfer-based segmentation captures greater subject-specific anatomical variation by not relying on the boundaries of an atlas generated from a small cohort of young adults. The JHU labels are visualized with respect to the corresponding atlas, whereas the FreeSurfer labels are visualized from a particular individual from our database.

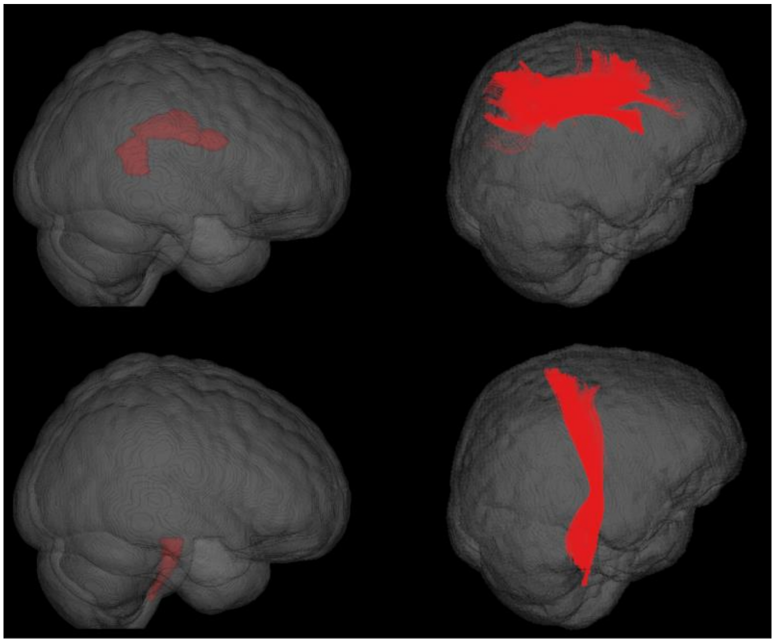

Supplemental Figure SE.F3. Comparison to Zhu et al. for tract specific white matter coverage. Zhu et al.'s method for tract-specific white matter coverage is based on the JHU atlas labels (left), whereas our method is based on subject-specific tractography using TractSeg (right). Notably, atlas-based approaches result in fundamentally spatially separated regions that do not constitute full pathways. Visualized are the left superior longitudinal fascicle (top) and the left corticospinal tract (bottom).

We have placed these comparisons in the newly added section that was mentioned above, titled "Comparison to Existing Reference Charts".

R2.C6: The gradients of tract maturation are a highly relevant and potentially novel aspect of the work, and my impression is that this should be explored in more detail. The authors should map out and visualize known spatial gradients, for instance, the posterior-to-anterior brain developmental pattern. This would also be informative for testing the “last-in, first-out” / “gain-predicts-loss” hypotheses (as part of this work, not in the future as currently stated in the discussion). Authors might be able to make an even more solid case for mapping which of the tensor measures 'matures' and 'declines' in line with the hypotheses. This could be achieved by plotting the areas with one axis first-to-last peak and the other one first-to-last decline.

We strongly agree that this is a highly relevant and potentially novel aspect of our work. This point dovetails with the major revisions we made in response to Reviewer 1, where we moved this from a future direction to a central result of the manuscript. We have substantially revised Figure 4 (and the section on Milestones) to provide the detailed exploration you suggested. We now demonstrate that our charts provide a robust framework for investigating these complex spatio-temporal dynamics. First, we do direct tests of these example hypotheses, operationally defining these hypotheses using rates of change or relative timings of developmental milestones to highlight (using just two examples) of how these can test the last-in-first out or gain-predicts-loss (Figure 4D) hypotheses. Second, we now explicitly map and visualize coordinated spatial gradients of change for both microstructure (FA) and macrostructure (volume) during infancy (maturation) and aging (decline). Showing this for all features, and all sets of pathways is beyond on the scope of the current work, but now enabled at large scale with our charts. Third, we have now integrated our in vivo trajectories with classical histological myelination patterns of Flechsig, Yakovlev, and Lecours.

We believe these major revisions substantially strengthen the manuscript by demonstrating the charts' power to investigate fundamental neurobiological questions.

R2.C7: The authors use GAMLSS to model the "normative trajectories" of white matter changes across the lifespan, which also enables tools to perform similar "centile scoring" for new studies, which is much in line with volumetric brain charts (Bethlehem et al. 2022). Indeed, many elements in the study are similar to Bethlehem et al. 2022 article, but at times it is unclear how the procedures were different. I would ask the authors to provide a supplementary table outlining similarities and differences to the 2022 brain charts article.

I would encourage a replication approach for the GAMLSS models and their validation and a clearly indicated and justified deviation from Bethlehelm et al. 2022. I would encourage the authors to include a leave-one-study-out (LOSO) validation of the growth centiles and benchmarking the similarity of the harmonization results of the out-of-sample centile scoring with existing methods (that in this case could be eHarmonize from Zhu et al. 2025).

The authors have chosen the Generalized Gamma distribution. This might not be optimal for FA values that range from 0 to 1.

This is an excellent suggestion, which prompted significant additional experiments to compare/contrast with existing charts and validate stability and out-of-sample alignment.

The work of Bethlehem et al. clearly motivated our own work; we purposely matched their methodology with three exceptions: 1.) Instead of releasing our brain charts on a privately hosted website, we release them on Zenodo, an archival platform built for retaining research data and results in an immutable way. 2.) Bethlehem et al. used multiple scans per participant but did not include appropriate statistical terms to model subject level effects (although did include supplementary longitudinal experiments). Thus, we include a maximum of one scan per participant in our brain charts. 3.) Multiple different versions of software (i.e. FreeSurfer) were used to extract features for the Bethlehem et al. brain charts. Although they include random effect terms in their model to account for this processing variability, we instead choose to keep all preprocessing and feature extraction pipelines consistent across datasets.

To provide more clarity, we have added Supplemental Table SE.T2 to the supplemental material (section "Comparison to Existing Reference Charts") as requested by the reviewer:

Supplemental Table SE.T2. Comparison of methods for our work to Bethlehem et al. (2022)

Study	*Participants*	*Repeated Scans*	*Normative Modeling*	*Processing Variability*	*Distribution Family*	*Out-of-sample Centile Scoring*	*Public release*
**Bethlehem**	101,457	Yes	GAMLSS; fractional polynomials for age	Yes; modeled in GAMLSS	Generalized Gamma	Yes, MLE	Website*
**Our Work**	35,120	No	GAMLSS; fractional polynomials for age	No	Generalized Gamma	Yes, MLE	Zenodo&

*https://brainchart.shinyapps.io/brainchart/; &https://zenodo.org/records/17561821

We now provide extensive experiments in parallel with Bethlehem et al., which test stability of our models and robustness to dataset effects (using LOSO validation), and to test out-of-sample alignment.

Our LOSO validation shows that our models, on average, experience <<0.5% coefficient of variation across the lifespan across all features and pathways. Although we find greater variation at the extremes (as expected), very few feature/pathway combinations ever show 5% coefficient of variation at any point throughout the lifespan. We show this as plots of LOSO coefficient of variation (COV) (of features/pathways), hot/cold maps of the average COV across the lifespan, and visually showing the bootstrapped centiles for example pathways.

Our out-of-sample alignment experiments are given in supplementary material section H. Although stability of estimates vary across the tracts, features, and parameters, we observe a conservative threshold for stability in OOS alignment at N=100. This threshold is similar to that from Bethlehem et al.[36], suggesting that our method is in agreement with existing normative brain charts. We further show that our OOS alignment is robust to processing pipeline and give detailed step-by-step directions on how to align new data (i.e. how to use) our charts.

Regarding the reviewer's comment about a comparison between existing methods, we regrettably cannot directly align our measurements to the existing eHarmonize curves from Zhu et al. because their ROI-based method is substantially different from our tractography-based method: they represent different sets of WM pathways/regions, and different distributions of derived phenotypes (i.e. they use the TBSS skeleton which explicitly extracts highest FA values). As in R2.C5, we do provide comparative plots for global white matter volume to those from Bethlehem et al. (Supplemental Figure SE.F1) and global white matter FA to those from Zhu et al (Supplemental Figure SE.F2).

We appreciate the reviewer's insight regarding the choice of the Generalized Gamma Distribution (GG), given that Fractional Anisotropy (FA) values are theoretically bounded between 0 and 1. We note that the GG is a highly flexible, three-parameter distribution that is appropriate for modeling any positive-valued continuous random variable. Furthermore, while FA is technically bounded from 0 to 1, the vast majority of our observed data for each tract is well below this theoretical maximum. Specifically, across all tracts, the maximum FA values in highly coherent pathways are rarely ~0.8 (averaged across the pathway). This means that the upper bound is not a major constraint on a potential heavy tail of the distribution.

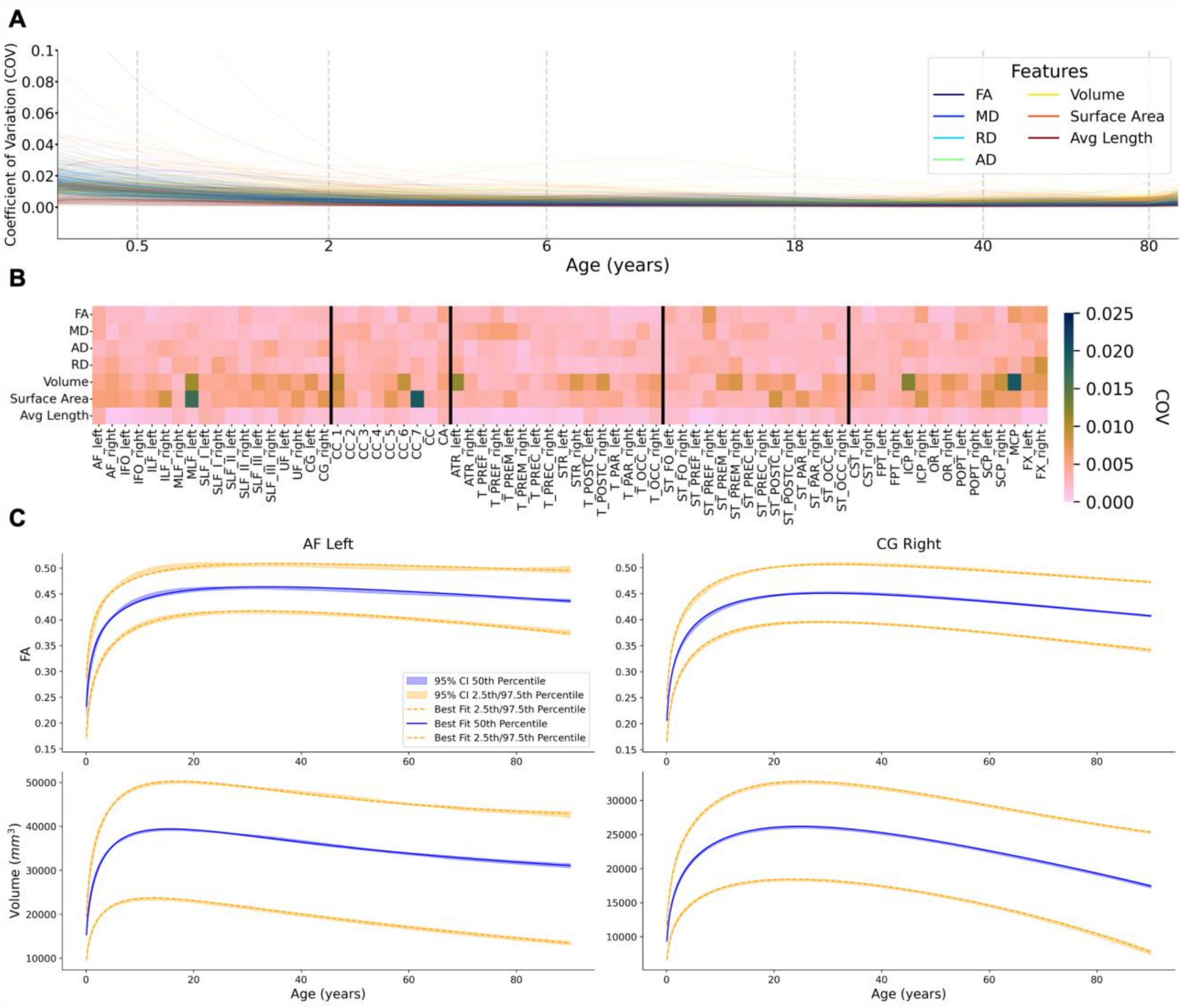

Supplementary Figure SD.F3. Model stability assessment using leave-one-study-out (LOSO) bootstrapping. We observe WM brain chart models to be stable across the entire lifespan. (A) Coefficient of variation (COV) from a LOSO bootstrapping analysis on the WM brain charts suggest a high stability across the majority of the lifespan for all models, with most models having a maximum of 1% COV at a given point in the lifespan. Age axis is log-scaled to highlight the spread of models at the beginning of the lifespan. (B) Averaging COV across all points in the lifespan demonstrate that most models have high stability as seen by low COV, with macrostructural models having a slightly larger COV on average. (C) Example plots of median (blue), and 2.5th and 97.5th (orange) percentiles with 95% confidence intervals derived from the LOSO bootstrapping show high stability across the entire lifespan and percentiles (top row is fractional anisotropy, bottom row is volume; left column is the left arcuate fasciculus (AF left), right column is the right cingulate gyrus (CG right)).

R2.C8: White matter volume charts seem to be in line with prior reports in Figure 1. What is the level of agreement of the total WM volume charts (from FreeSurfer) to those reported by the Bethlehem et al. article? Showing strong agreement would strengthen the study by showing a potential comparability to established gross anatomical WM charts.

We agree that a direct comparison would show comparability and strengthen relevance. Thus, we compare our brain charts for WM volume to those from Bethlehem et al. in an added section to the Supplementary Material:

Comparison to Existing Growth Charts

Other works have been published on the use of neuroimaging data to construct growth charts for WM measurements[9,36], but have not been as extensive with regard to the number of features. Specifically, Bethlehem et al. only investigate global WM volume, whereas Zhu et al. investigate only microstructure through FA.

As our method for assessing global WM macrostructure follows Bethlehem et al. in the use of FreeSurfer and GAMLSS, we can more directly compare our brain charts to theirs (**Supplemental Figure SE.F1**). Notably, our curves follow the same trend of a rapid increase during infancy that plateaus during early adulthood, then finally dropping off during aging. We believe this agreement in capturing the general trend indicates that our brain charts are comparable to other established normative models for neuroimaging.

However, we note that there are some slight differences in the two sets of global WM volume curves. While the age at peak is not very representative, this value has tended to vary in the literature quite a bit as well. Specifically, our observed peak for WM volume is slightly less that Lebel et al. (peak at 37 years) [17], almost 10 years later than Courchesne et al. (beginning of the 4th decade) [37] and 5 years later than Bethlehem et al. [36], and 10 years earlier than Giorgio et al.[13]. Furthermore, the Bethlehem curves were fit using pre-natal data and used multiple different methods to obtain WM volume measurements, specifically, different FreeSurfer versions and hand drawn ROIs, whereas our curves contained only post-natal data and stayed consistent with our data processing. Finally, Bethlehem et al. included multiple scans per participant, which was not explicitly modeled in their GAMLSS formula, whereas we only include one scan per participant. These factors, in addition to the largely increased sample size used by Bethlehem et al., could be contributing to the slight differences between the curves.

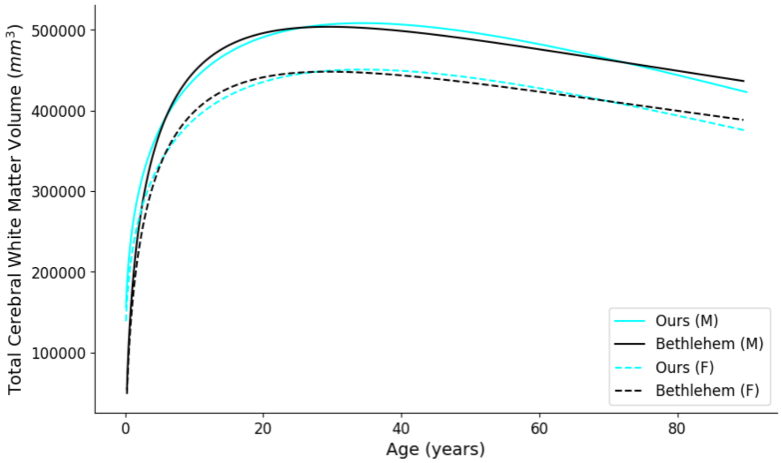

Supplemental Figure SE.F1. Comparison of our global white matter volume curve to Bethlehem et al. Our brain charts (median trajectories plotted) follow the same trend as Bethlehem et al., which is a rapid increase during infancy that plateaus during early adulthood and finally drops off during aging. We believe this agreement in capturing the general trend indicates that our brain charts are comparable to other established normative models for neuroimaging.

R2.C9: It is important to be open about the parts of the brain that are not covered in the regions of interest. Do the total WM vs. sum of TractSeg volumes match so that the entire white matter is covered? If not, which parts of the brain are not included in the tract-specific analyses?

This is an interesting point of discussion. First, we want to emphasize that our "total WM volume" is derived from traditional WM segmentation approaches (FreeSurfer), which differs from pathway-specific volumes for tract-level analysis, but is consistent with classical ways of extracting total tissue volume.

We explicitly do not want to sum the total volume of all pathways, as there is considerable overlap in these pathways, an effect referred to as the "bottleneck challenge" in tractography communities.[33] We point the reviewer to our own work[32] which investigates areas (voxels) of the brain that contain a single well-reconstructed pathway, versus those that contain multiple (or even 8+ pathways). We note that a large majority of the white matter is occupied by the large association, projection, and commissural pathways – but we still do not want to confuse phenotypes of global WM versus phenotypes of tract-specific WM.

Still, we provide a visualization of both the whole brain WM from FreeSurfer and all reconstructed TractSeg tracts for comparison as Supplemental Figure SB.F3.

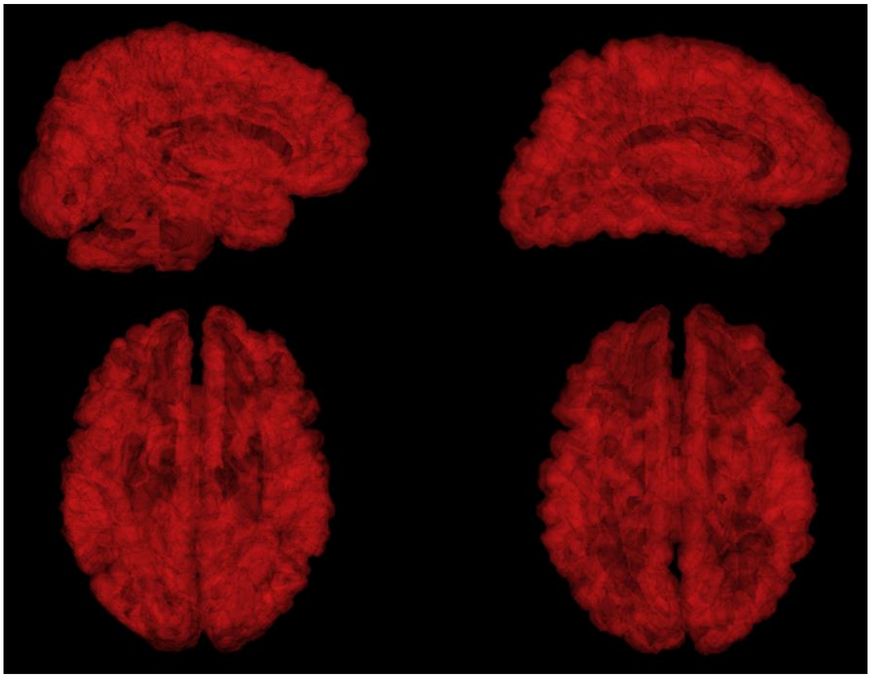

Supplemental Figure SB.F3. Comparison of TractSeg vs FreeSurfer white matter coverage. The overlap in coverage between (left) the aggregate of all TractSeg tracts and (right) the white matter mask from FreeSurfer is substantial. The most notable difference is that the FreeSurfer mask does not include the cerebellar or brainstem regions.

R2.C10: 10. Strikingly, males and females seem to have almost identical DTI tensor brain charts in most presented centile curves. This is somewhat surprising and also differs from prior work but could well be a true finding. This would be important to emphasize in the main manuscript text and to address in additional analyses. Clearly, there are some differences in TBSS-based and tractography-based data that would require detailed comparisons. Would the sex differences be apparent in smaller, eroded tracts?

Another aspect that might be worth including in the comparative analyses is related to volumes that are readily available for the authors. For a given DTI scan, participants that have larger brain volumes have more voxels within the DTI image volume. They would thus have higher proportional spatial resolution (similar to smaller voxel size), which could be detected as higher FA values. This type of negative association between FA and voxel volume is well depicted in Zhu et al. 2025 Figure 3.

We apologize for the lack of clarity regarding this point and would like to emphasize that we do find significant differences between male and female trajectories. We note that reducing the entire population down to a single statistical test of means/medians is losing a tremendous amount of information on the population distribution, so we do not want to include theses statistical tests within the primary manuscript. In line with literature, we find increased FA and diffusivities in males, although results are heterogenous and depending on pathway. We find consistently greater macrostructural features in males than females.

To improve clarity regarding this point, we have included Supplemental Figure SD.F6:

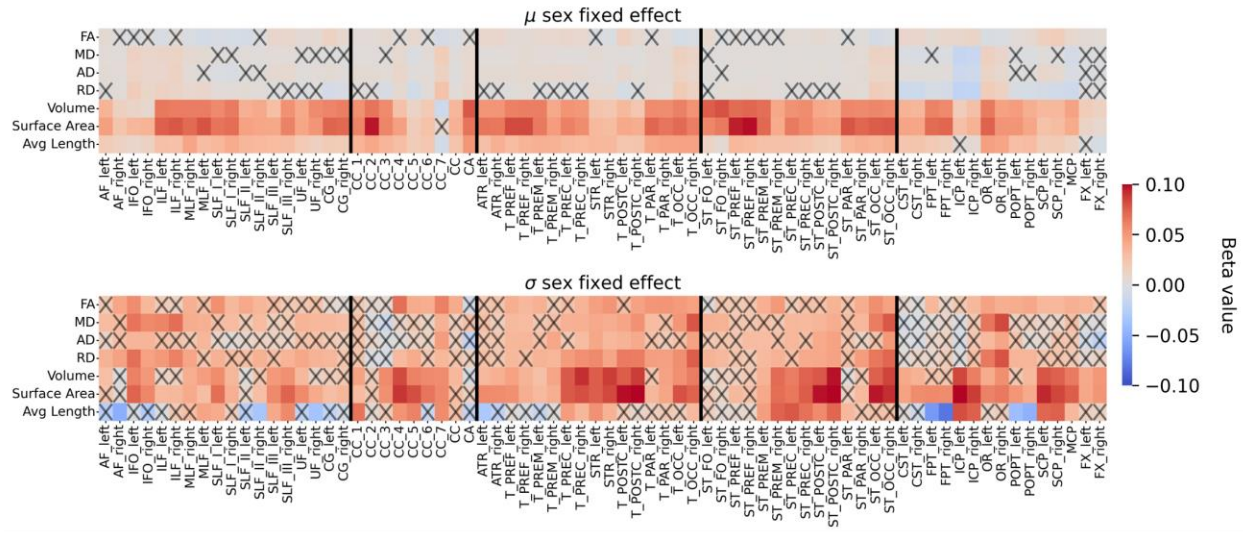

Supplemental Figure SD.F6. Significance of sex fixed effect across models. In the GAMLSS fitting, we find that the fixed effect of sex for μ (top) is significant for most models, whereas the sex fixed effect for σ (bottom) is much less significant (after Bonferroni correction for multiple comparisons). Positive (red) indicates that corresponding trajectory values for males are larger than those of females, whereas blue indicates the opposite. Macrostructural trajectories tend to have much larger values for μ than microstructural trajectories. All trajectories tend to have larger male variability, indicated by the positive (red) values for the σ fixed effect. Note that, due to the GAMLSS model specification, fixed effect values are logarithmically scaled and multiplicative in nature.

Related to voxel size and brain volume: just as depicted in Zhu et al., resolution of the imaging data will affect the FA values, which is a known effect for DTI-based analyses. We have also found this negative association to be the case with our brain charts, where voxel size is negatively correlated with the value of the random effect term for mu in the GAMLSS equations. We have added this analysis to the supplement as Supplemental Figure SD.F4.

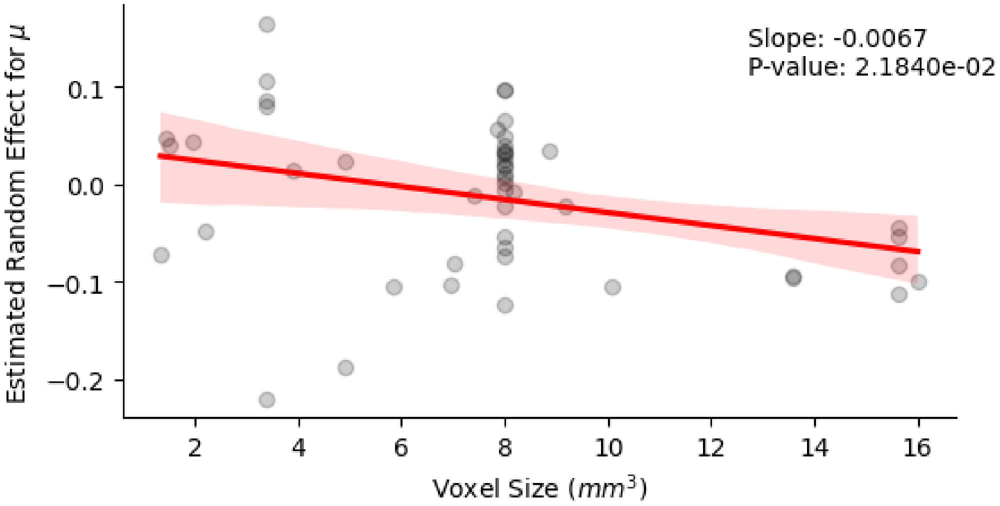

Supplemental Figure SD.F4. Voxel size correlation with dataset-estimated shift. We observe that for the whole-brain white matter FA, voxel size has a significant negative correlation with the estimated values for the GAMLSS random effect terms for μ. This indicates that the GAMLSS model is appropriately modeling acquisition-related variability across datasets for the brain charts. Note that in the GAMLSS formulation, μ is log-transformed, and thus the random effect terms are multiplicative instead of additive. For example, a random effect value of -0.1 corresponds to a multiplicative shift in the dataset values by $e^{-0.1} = 0.904$.

R2.C11: The authors use the centile scores to compare patient vs. control groups across "neurological conditions" that span mild cognitive impairment (MCI), Alzheimer's disease (AD), and epilepsy. The choice of these conditions is not clear to the reader, as none of them are white matter specific in pathophysiology and are not representative of neurological diseases in general. In the supplements, there are also other case vs. control comparisons for "other clinical cohort groups": ADHD, autism, anxiety, depression, the individuals that convert from no cognitive impairments to either MCI or AD, and substance abuse, which is not clearly justified or referenced in the main manuscript. It could be a viable approach to replicate most of the abnormal phenotypes that were used in Bethlehem et al. 2022 article and ideally extend this to a chosen few additional 'clinical' phenotypes.

There could also be added value in studying associations between case-control comparisons. Some ideas would be birth weight vs. global measures and language skill scores vs. 'language tracts.' This is relevant also since most of the data is from English-speaking countries, making language skill assessments potentially relevant.

Authors should reconsider wording that is used in this part of the manuscript ("neurological conditions" / "other clinical cohorts" / "other cognitive groups"), i.e., think of more descriptive names that are used consistently.

We appreciate the suggestion from the reviewer to stick more closely to the clinical cohorts used in Bethlehem et al. To that end, we have altered Figure 5 to focus on the cohorts highlighted in Bethlehem et al. – Depression (MDD), Anxiety (ANX), Attention-Deficit Hyperactivity Disorder (ADHD), Autism Spectrum Disorder (ASD), Schizophrenia (SCZ), Mild Cognitive Impairment (MCI), and Alzheimer's Disease (AD). Additionally, we note that while Bethlehem et. al focused on these clinical cohorts in the main text, they also provided additional case-control comparisons in the Supplementary Information. Thus, we do the same in Supplemental Figures SC.F1-SC.F3.

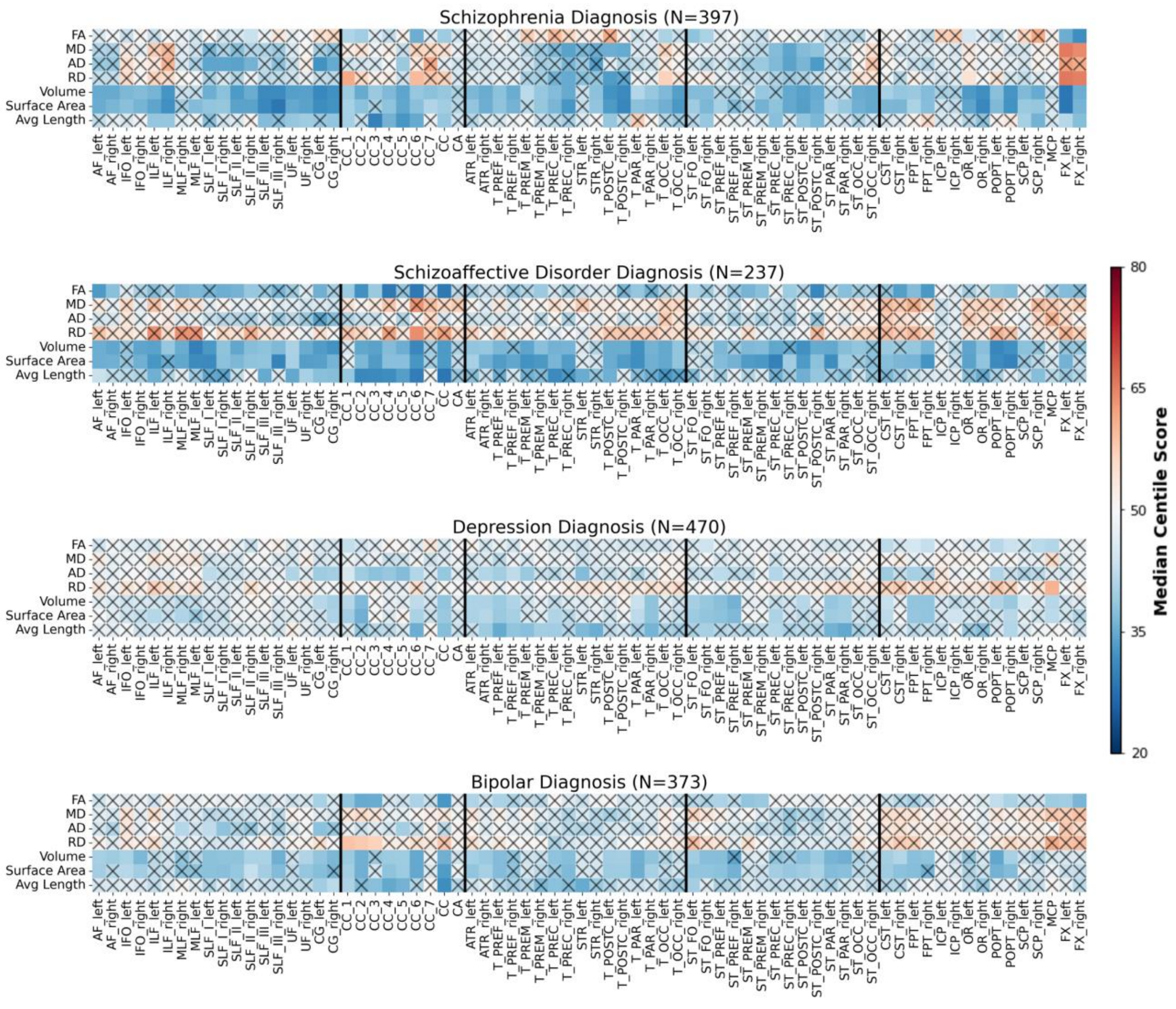

Supplemental Figure SC.F1. Centile score differences across major depressive and psychotic disorders. All major psychoses and major depressive disorder (top to bottom: schizophrenia, schizoaffective disorder, major depressive disorder, and bipolar disorder) showed localized reductions in tract macrostructure and microstructure under the one sample Wilcoxon after Bonferroni correction for multiple comparisons.

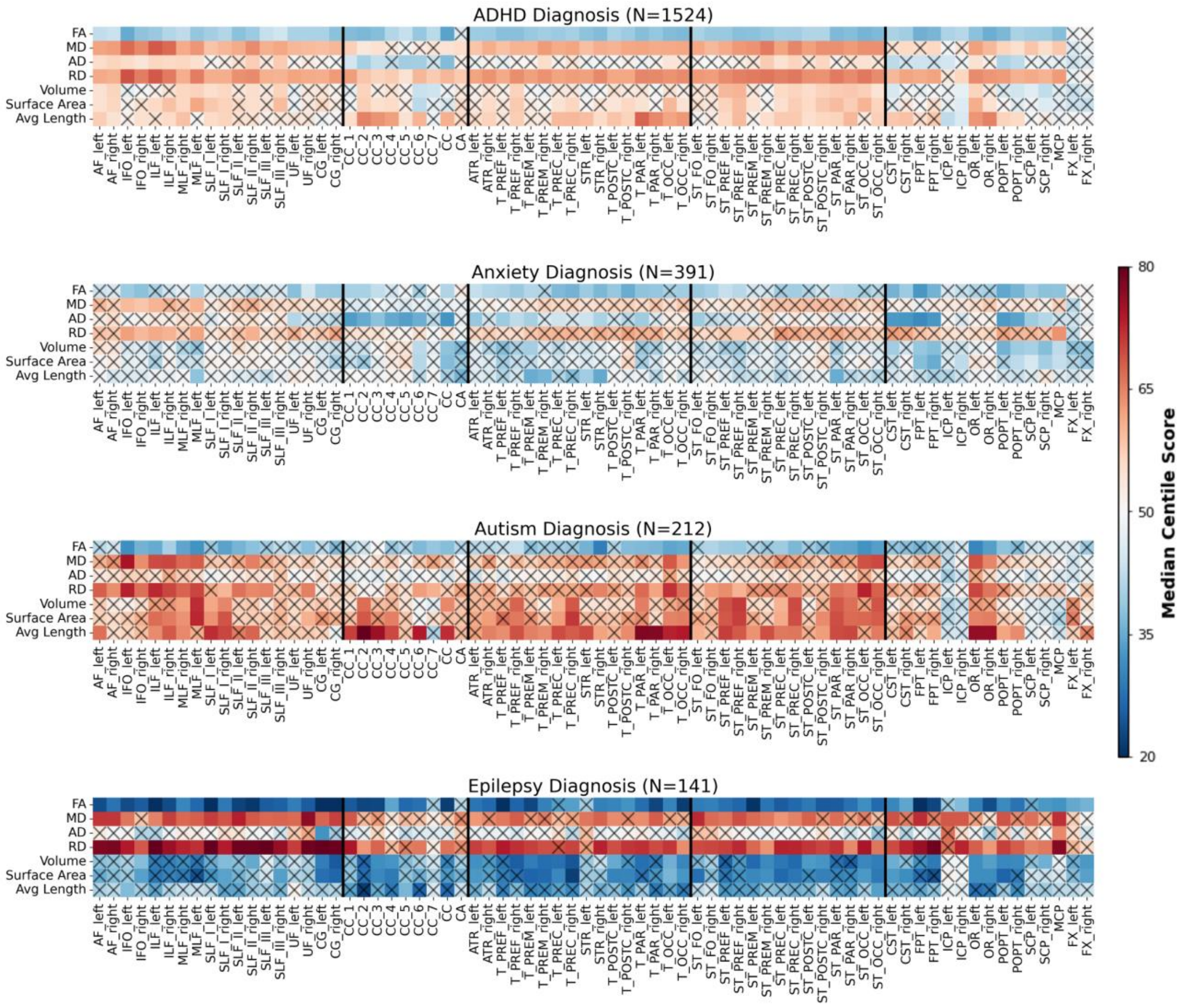

Supplemental Figure SC.F2. Centile score differences across other neurocognitive disorders with significant deviations. For many other clinical cohorts (top to bottom: attention-deficit hyperactivity disorder (ADHD), anxiety, autism spectrum disorder, and epilepsy), we observe either localized deviations in tract microstructure and macrostructure or consistent deviations across all tracts within specific macrostructural and microstructural features. Individuals with epilepsy and ADHD showed substantial deviations in FA and RD across the WM, suggesting diffuse microstructural abnormalities, alongside more localized reductions in volume, length, and surface area in select tracts. For individuals diagnosed with autism spectrum disorder, we observe increases in macrostructure across tracts, with a subset of tracts showing significant deviations. Finally, we observe sparsely significant deviations in microstructure and macrostructure for individuals diagnosed with anxiety.

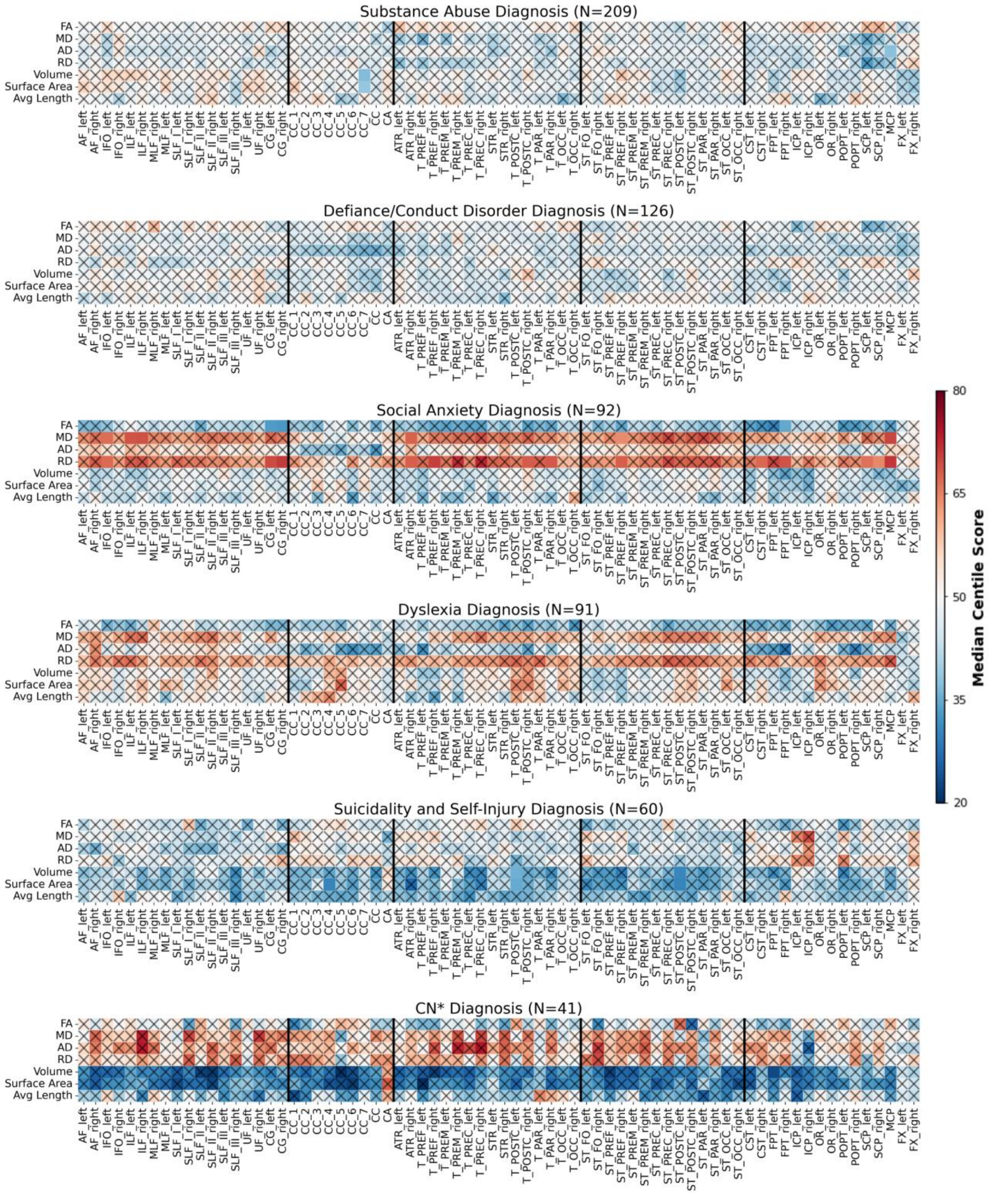

Supplemental Figure SC.F3. Centile score differences across other neurocognitive disorders without significant deviations. For many other clinical cohorts (from top to bottom: substance abuse, defiance/conduct disorders, social anxiety disorder, dyslexia, suicidality and self-injury, and individuals who start as cognitively unimpaired and then transition to cognitive impairment, or CN*), we do not observe significant deviations in centile scores. However, there are still non-significant patterns across tracts, such as reduced tract macrostructure in CN* participants and increased MD/RD in the social anxiety cohort.

Regarding associations with behavior or physiological measures: we fully agree that this type of analysis would be highly relevant for studying brain development and language, particularly given the large portion of data from English-speaking cohorts, making language skill assessments a particularly interesting avenue. However, a comprehensive study of these specific associations is beyond the scope of the current work. Our primary goal is to provide reference charts and a robust resource for the greater neuroimaging field. We have released our brain charts in such a way that, allows other researchers the ability to study any function and relate abnormalities to behavioral, physiological, lifestyle, or genetic factors.

To demonstrate the potential for this type of analysis, we included an illustrative example in Figure 6c, showing how our charts can be used to compare centile scores against another variable of interest (cognitive scores). We have clarified the text in the Results section to explicitly state that this figure serves as an example of how the reference charts can be used to relate abnormality indices to a broad range of external data, such as those suggested by the reviewer:

> …Second, the centile framework enabled associations between WM structure and cognitive performance to be examined, identifying pathway-specific features most strongly linked to clinical variability (**Figure 6c**). In this way, the reference charts can be used to relate abnormality indices to a broad range of external clinical data.

Furthermore, we must note that birth weight and language skill scores are not consistently available across all cohorts in the dataset, which would limit the power of such an analysis in our current publication. Our current focus is on providing a tool that enables this type of research on a massive scale for any researcher with access to relevant external data.

Consistency of nomenclature: we apologize for the lack of consistency regarding the clinical populations not used to fit the normative models. We now refer to these "other clinical cohorts" more broadly as "diagnostic groups" throughout the manuscript.

R2.C12: There are scant details about DTI preprocessing (citation to prior work is not sufficient). I would ask the authors to provide the key details of the procedures they used by listing the main steps and including a complete description in the supplementary materials.

On a related note, the authors are not testing how image quality measures might influence the DTI tensors and/or the centile scores. PreQual output includes multiple image quality measures, and the authors should both report these parameters in detail for each of the data sets and test if the centile estimation is robust to variability in image quality measures.

We apologize for the lack of clarity on our end. For more transparency, we have altered the methods section to list every software, version, of the software used in our pipeline:

Diffusion-weighted imaging (DWI) data were preprocessed using the PreQual pipeline[38], which corrects for susceptibility-induced, motion-related, and eddy current distortions, and performs slice-wise signal imputation. Specifically, image denoising was performed using the MRtrix3[39] (version 3.0.4) implementation of Marchenko-Pastur principal component analysis. Following this, TOPUP from the FSL (version 6.0.4) software library[40] is used for susceptibility-distortion correction. For DWI without reverse phase encoding scans acquired, TOPUP is run using a synthetic b0 image created from a T1-weighted scan from the same imaging session via the Synb0-DISCO[41] algorithm (version 3.1). FSL's EDDY is then used for motion and eddy-current-distortion correction, also performing slice-wise signal imputation with the flag `*--repol*`. [45][48]Following preprocessing, diffusion tensor imaging (DTI) models were fit to all volumes with b-values ≤ 1500 s/mm² [42,43] using *dwi2tensor* from MRtrix3. DTI-derived scalar maps - including fractional anisotropy (FA), mean diffusivity (MD), axial diffusivity (AD), and radial diffusivity (RD) - were computed using *tensor2metric* from MRtrix3 [39].

To enable consistent tract segmentation, all diffusion data were resampled to 1 mm isotropic resolution using MRtrix3 [44]. Tractography was performed using TractSeg [45] (version 2.8), which automatically segments 72 anatomically defined cerebral white matter pathways. For each tract, we computed streamline density-weighted averages of DTI metrics (FA, MD, AD, RD) as well as macrostructural features - volume, length, and surface area - using the Scilpy toolkit (version 1.5.0) (https://github.com/scilus/scilpy.git) scripts *scil_compute_bundle_mean_std.py* and *scil_evaluate_bundles_individual_measures.py* respectively. Both raw and ratio-normalized macrostructural features are provided, where volume, surface area, and average length of tracts are normalized to total brain volume (excluding ventricles), estimated total intracranial volume, and cerebral white matter volume.

T1-weighted images were included only when acquired in the same session as DWI data. Brain segmentation was performed using the *recon-all* command from FreeSurfer (v7.2.0) [46], yielding estimates of cerebral white matter volume, brain volume excluding ventricles, and total intracranial volume. For participants aged ≤2 years, we employed *infant_recon_all* from the infant FreeSurfer pipeline [47] (v0.0) to account for age-specific brain morphology. Cerebral WM masks were defined using MRtrix3 [39] 5TT labels, excluding the cerebellum and brainstem. T1w images and corresponding WM segmentations were rigidly registered to DWI space using FSL's [40] *epi_reg*. Whole-brain WM DTI metrics were then computed by averaging values within the WM mask.

Additionally, we have included a container for all postprocessing as a Docker image that can be downloaded from https://hub.docker.com/r/kimm58/wm_lifespan_processing with instructions for running the container available at https://zenodo.org/records/17144461. This Zenodo page includes detailed instructions on how to run the container as well as a thorough description of all the postprocessing steps in the Appendix.

We agree that reporting the IQMs for PreQual across datasets would increase transparency regarding dataset quality. Thus, we include Supplemental Figure SF.F1, visualizing the metrics of mean Chi-squared, CNR of the main shell used in tensor fitting, and mean relative displacement (motion) across datasets. Further, we include Supplemental Figure SF.F2, which highlights that centile estimation is uncorrelated with any of these IQMs.

Data Quality

As mentioned in the main manuscript, we conducted a thorough quality control process that involved visualizing each scan and performing quantitative outlier removal (see Methods: Data Selection). The final results of this process can be found in Supplemental Table SB.T1. Notably, infant datasets tend to have many more scans that are not included in the final participant pool for creating the brain charts. We attribute this effect largely to TractSeg, which was trained on an adult population. As infant data are more out-of-distribution compared to other datasets that the TractSeg models were not trained on, many infant scans showed poor tract reconstruction.

Furthermore, we conducted an analysis to assess both image quality across datasets and how image quality affects downstream centile score estimates. Specifically, we visualized distributions for the PreQual image quality metrics (IQMs) of mean relative displacement, the chi-squared error metric in shells used for diffusion tensor fitting, contrast-to-noise ratio (CNR) as calculated by EDDY for the main shell used for tensor fitting for scans included in creating the brain charts (Supplemental Figure SF.F1). We do not observe IQMs to be influenced by age at time of scan, as distributions are not consistently higher or lower at any given age range. In other words, IQM distribution appeared to be dataset-dependent rather than age-dependent.

For assessing correlation of estimated centile scores with IQMs, we computed Spearman's correlation coefficients between centile scores and each IQM (Supplemental Figure SF.F2). All correlations were either low or non-existent, with nearly all correlations is below 0.15 and none above 0.19. These results suggest that image quality of the dMRI data does not largely affect the estimated centile scores.

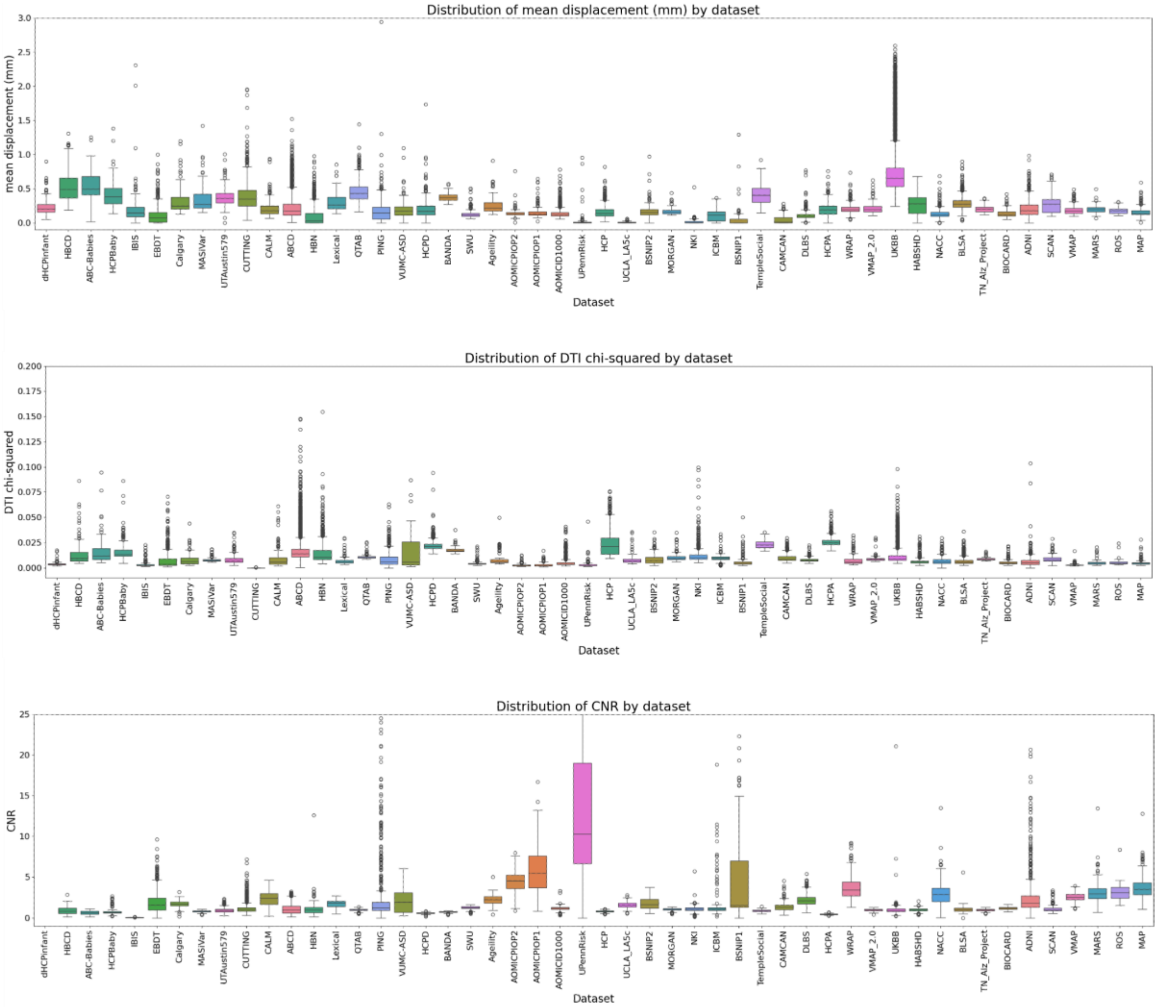

Supplemental Figure SF.F1. Distribution of PreQual image quality metrics by dataset. We do not observe age at time of scans to largely influence image quality metrics (IQMs) for dMRI preprocessing, as IQM distributions are not consistently higher or lower at any age range. Plotted are (top) motion as mean relative displacement, (middle) chi-squared error metric in the shells used for the diffusion tensor imaging fit, and (bottom) contrast to noise ratio (CNR) as calculated by eddy for the main shell used for the tensor fitting. Datasets are ordered by mean age of participants, with the youngest ages on the left and oldest on the right.

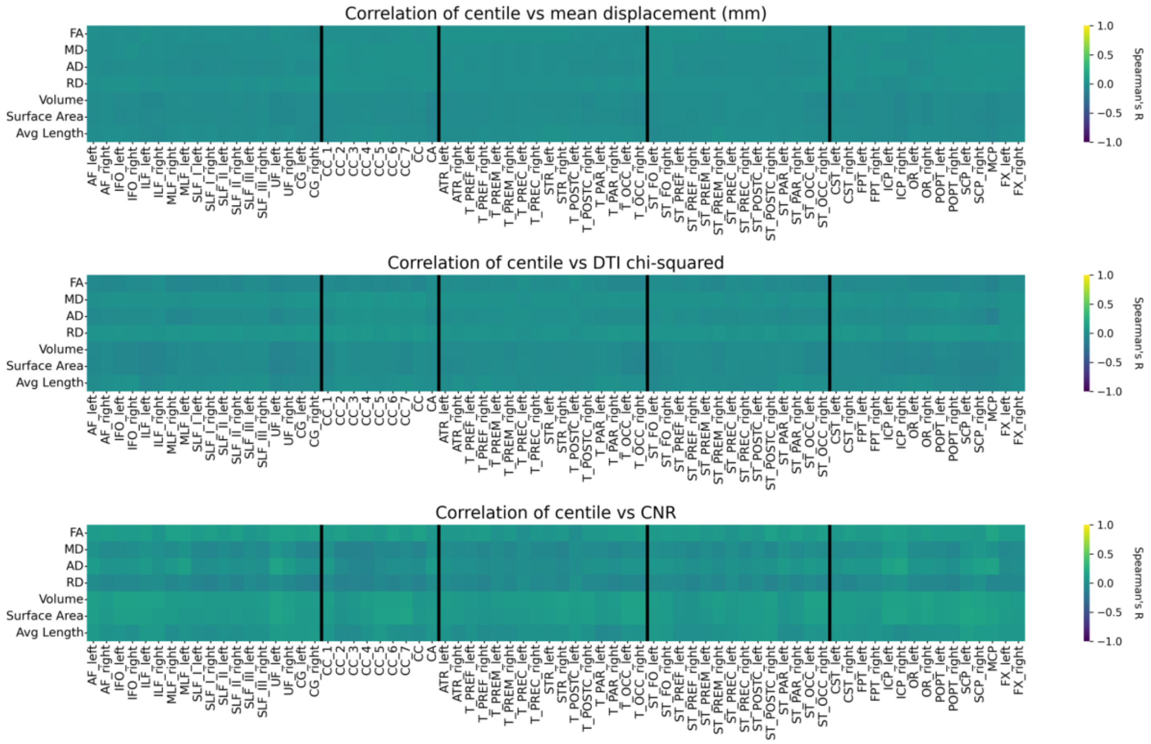

Supplemental Figure SF.F2. Correlation of PreQual image quality metrics with centile scores. We do not observe image quality metrics (IQMs) for dMRI preprocessing to be correlated with observed centile scores, as the absolute value of nearly all correlations is below 0.15, with none above 0.19. Plotted are Spearman's correlation coefficients with (top) motion as mean relative displacement, (middle) chi-squared error metric in the shells used for the diffusion tensor imaging fit, and (bottom) contrast to noise ratio (CNR) as calculated by eddy for the main shell used for the tensor fitting.

R2.C13: I was left wondering what the requirements are for new data sets to be suitable for centile scoring. The authors are encouraged to provide as much detail and some analyses using differential preprocessing for out-of-centile scoring. Would investigators need to use PreQual or a similar preprocessing workflow? Are there any requirements for image quality, such as a minimum number of diffusion-encoding directions?

This is highly relevant, as for many existing data sets it is important to maintain consistency by using the same data set in most studies. The key questions are: could we use already preprocessed data as an input even if another software package/pipeline has been used for preprocessing? What is the minimum sample size that would be sufficient for centile creation?

These are important question raised by the reviewer, with implications possibly extending potential use of our brain charts to institutions that choose different preprocessing pipelines. The preprocessing pipeline that we use, PreQual, is generically state of the art for dMRI preprocessing, as it contains all the fundamental steps that must be done (corrections for susceptibility-distortions, motion, eddy-current-distortions). These fundamental steps are present in most dMRI preprocessing workflows[48–52] and implemented using the most common algorithms/ softwares (FSL, MRtrix3). Additional steps that are considered beneficial for data quality (denoising; slice-wise outlier imputation) are also included in the preprocessing pipeline. Thus, researchers can generically use different preprocessing pipelines. Regardless, we demonstrate that even non-preprocessed data can be moved to our charts as an extreme case in the supplemental material as Supplemental Figure SH.F2:

Out-of-Sample Alignment With Different dMRI Preprocessing

The DWI preprocessing pipeline that we use, PreQual, is generically state-of-the-art, as it contains all the fundamental steps that are considered "essential" (corrections for susceptibility-distortions, motion, eddy-current-distortions)[48]. These fundamental steps are present in most dMRI preprocessing workflows. Although we used PreQual to preprocess our DWI data, we acknowledge that alternative tools for DWI preprocessing exist, such as DTIPrep/Dmriprep[49,53] DSI-Studio[51], ExploreDTI[52], and TORTOISE[50]. We also include additional steps that are considered beneficial for data quality (denoising; slice-wise outlier imputation). Thus, researchers can generically use different preprocessing pipelines.

To assess whether or not data corrected with different DWI preprocessing pipelines are align-able to our brain charts, in an extreme case, we performed out-of-sample alignment using data from the AOMICPIOP2 dataset[54] that had not had any DWI preprocessing. We then compared the original centile scores for each participant to those obtained via the raw data alignment. Most features are highly correlated (Supplemental Figure SH.F2), suggesting that even in this extreme case, data are able to be aligned to our brain charts without having run data through the PreQual preprocessing pipeline. However, we do note that there are a few features, notably smaller tracts like the fornix (FX) or anterior commissure (CA), that are not as highly correlated. Regarding specific tract groups, we observed that cerebellar tracts (MCP, ICP, SCP) and projection tracts (FPT, CST) also exhibited lower correlations than other tract groups, likely due to their increased sensitivity to distortion artifacts in these brain regions.[55] We did not observe any features to be more or less correlated across tracts when compared to other features.

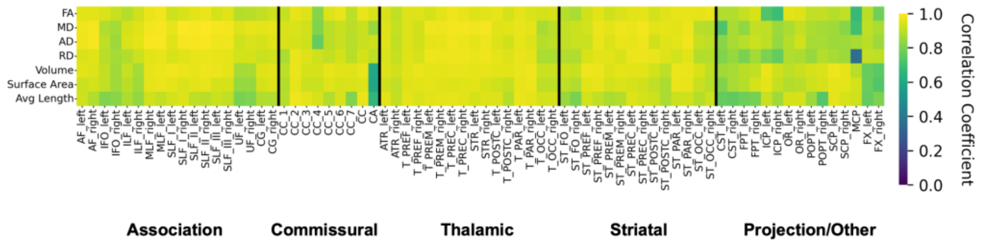

Supplemental Figure SH.F2. Pearson correlation of centile scores for the AOMICPIOP2 dataset with and without dMRI preprocessing. Centile scores from both preprocessed and raw data are highly correlated across most tracts and features; this extreme case indicates that DWI data that have undergone different preprocessing workflows are can be appropriately aligned to our brain charts. Measurements from smaller tracts (FX, CA) and cerebellar/projection tracts are most affected by lack of DWI preprocessing, showing reduced correlations when compared to other tracts.

With regard to image quality requirements, the generic TractSeg pipeline uses constrained spherical deconvolution (CSD) (either single shell or multi-shell) to extract principal directions of diffusion. It is generally accepted that this process requires roughly 30 directions for accurate estimation; however, CSD can also be performed with fewer directions, with well reconstructed pathways using TractSeg techniques. We do not assess the limits of CSD in this manuscript, as we feel it is out of scope for this work. Nonetheless, we include the following line in the Supplementary Information for the newly added section "Out-of-Sample Alignment With Different dMRI Preprocessing":

> We note that, as part of our postprocessing pipeline, constrained spherical deconvolution (CSD) is run to extract peak directions for downstream tractography. Although not evaluated in this manuscript, it is generally accepted that CSD requires roughly 30 directions for accurate estimation; however, CSD can also be performed with fewer directions, albeit with less stable estimates.

For the minimum sample size requirements, we have added an additional analysis for determining a threshold (see also R2.C7). Notably, our threshold is similar to the one that Bethlehem et al. found for OOS alignment:

<u>Out-of-sample Alignment Analysis</u>

To assess the stability of the out-of-sample (OOS) alignment process, we performed OOS alignment for the HCPA dataset at varying sample sizes. At each sample size (from 700 to 50 in increments of 50) ,we randomly selected participants from the dataset without replacement, with 100 different bootstrapped assessments at each sample size. We did this for both FA and volume of the right cingulum and isthmus of the corpus callosum. Across all features, we observed that the OOS alignment process is more stable when estimating the μ random effect term compared to the σ random effect term (Supplemental Figure SH.F1). Although stability of estimates vary across the tracts, features, and parameters, we observe a conservative threshold for stability in OOS alignment at N=100. This threshold is similar to that from Bethlehem et al., suggesting that our method is in agreement with existing normative brain charts.

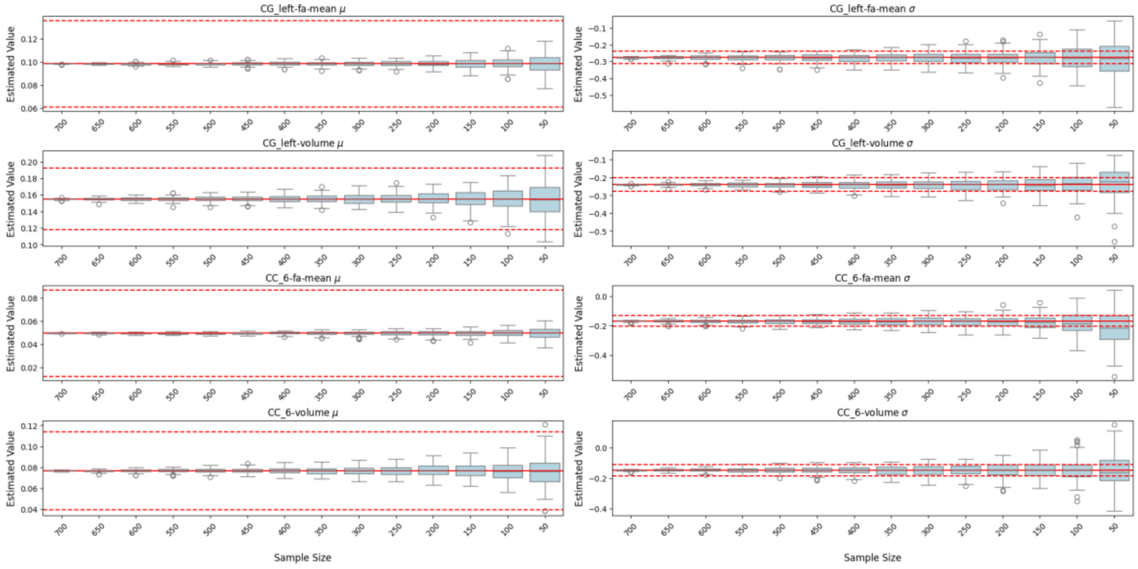

Supplemental Figure SH.F1. Out-of-sample alignment bootstrapping experiment for determining minimum sample size. Out-of-sample (OOS) alignment appears stable and within the GAMLSS-estimated standard error (solid red lines indicate the GAMLSS-estimated value, dashed red lines indicate error bars) for sample sizes greater than or equal to 100. The estimated random effect term for μ (left) appears more stable for OOS alignment than it is for σ (right), as the distributions for bootstrapped random effects fall within the GAMLSS error range at smaller sample sizes for μ. We observe varying levels of stability for different features and tracts (from top to bottom: FA of right cingulum, volume of right cingulum, FA of isthmus of corpus callosum, volume of isthmus of corpus callosum), with N=100 as a conservative threshold.

R2.C14: This work is impressive in that it creates centiles for + 1000 brain measures. It is understandable that all of this information cannot be reported in the manuscript. I found the choice of regions of interest and left/right side that were chosen for main article figures arbitrary and, to a degree, non-informative. Could the main display items focus on a set of white matter tracts or bilateral examples from chosen 'tract groups'?

It would also strengthen the report if the authors created means for checking the ROI-wise centiles for all available measures. This might be too much for an article supplement, but maybe an online repository to share the ROI-wise figures or even a separate tool/container that can create the plots?

The lack of population representativeness in most neuroimaging studies should be clearly stated as a limitation of this study as one of the " ... gaps (that) will require continued efforts to increase global diversity and dataset representativeness."

We selected tracts for display in the main text based on the prevalence of study in the literature[56], with the aim that the manuscript reflects relevance to the broader scientific community. Notably, the corticospinal tract (CST), cingulum (CG), arcuate fasciculus (AF), and corpus callosum (CC) have historically been the most studied tracts in both research and clinical domains. We included the anterior thalamic radiation (ATR) as well in order to provide representation of other tract groups. We have attempted to consistently use this set of representative tracts throughout the manuscript as best as possible (Figure 2, 3, 4a, Supplemental Figures SA.F3-AS.F16). As we create brain charts for each separate tract in TractSeg, we do not combine bilateral examples (many tracts have a left and right) or merge separated parts of a larger tract (such as the corpus callosum).

Since we are not studying laterality or asymmetry in this manuscript, we did not focus on the differences between the right and left tracts. However, we provide the contralateral side of tracts (and a more posterior portion of the corpus callosum) for each of the main figures in the supplementary materials as Supplemental Figures SA.F1 and SA.F2. Studies of laterality are an area of future interest.

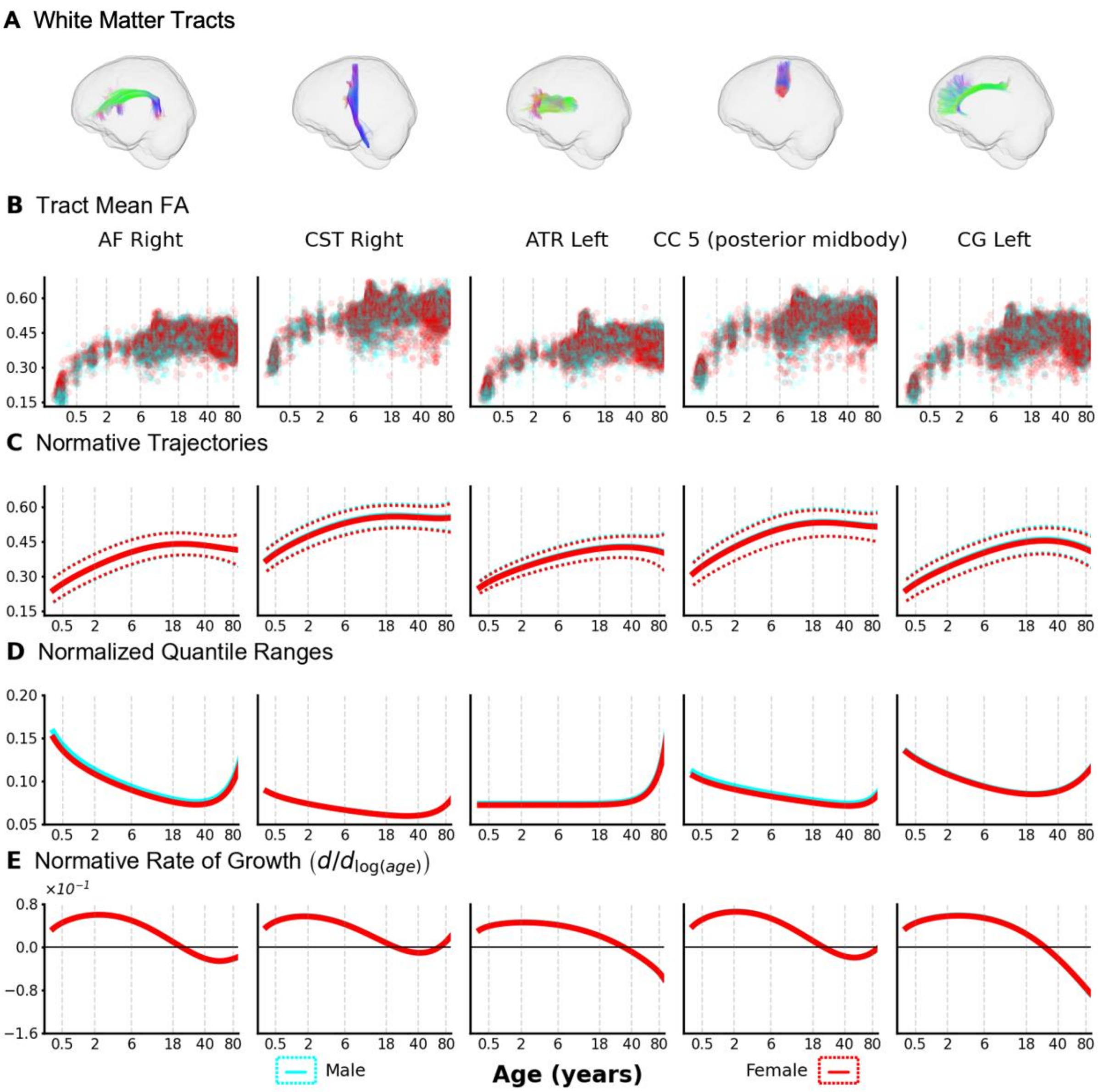

Supplemental Figure SA.F1. Tract-specific Microstructural Brain Charts - Contralateral. Lifespan brain charts for white matter *microstructure* (FA shown for contralateral tracts of **Figure 2**) reveal distinct developmental trajectories and variability across different WM pathways. A) Five exemplar tracts are shown (left to right): right arcuate fasciculus (AF Right); right corticospinal tract (CST Right); left anterior thalamic radiation (ATR Left); posterior midbody of the corpus callosum (CC 5 (posterior midbody)); and the left cingulate gyrus (CG Left). B) Raw FA data points for these tracts extend across the lifespan. C) Normative GAMLSS trajectories show FA increasing during development, plateauing in adulthood, and declining in later life, where the timing and magnitude vary by tract. Median (solid lines) and 2.5th/97.5th percentiles (dotted lines) are shown. D) Normalized quantile ranges indicate FA variability is generally lowest in middle age and increases later in life, though developmental variability patterns differ between tracts. E) The normative rate of change (first derivative) suggests that FA peaks at different ages and changes at different rates depending on the specific tract. Note: The x-axes (age in years) are log-scaled to emphasize developmental and aging periods.

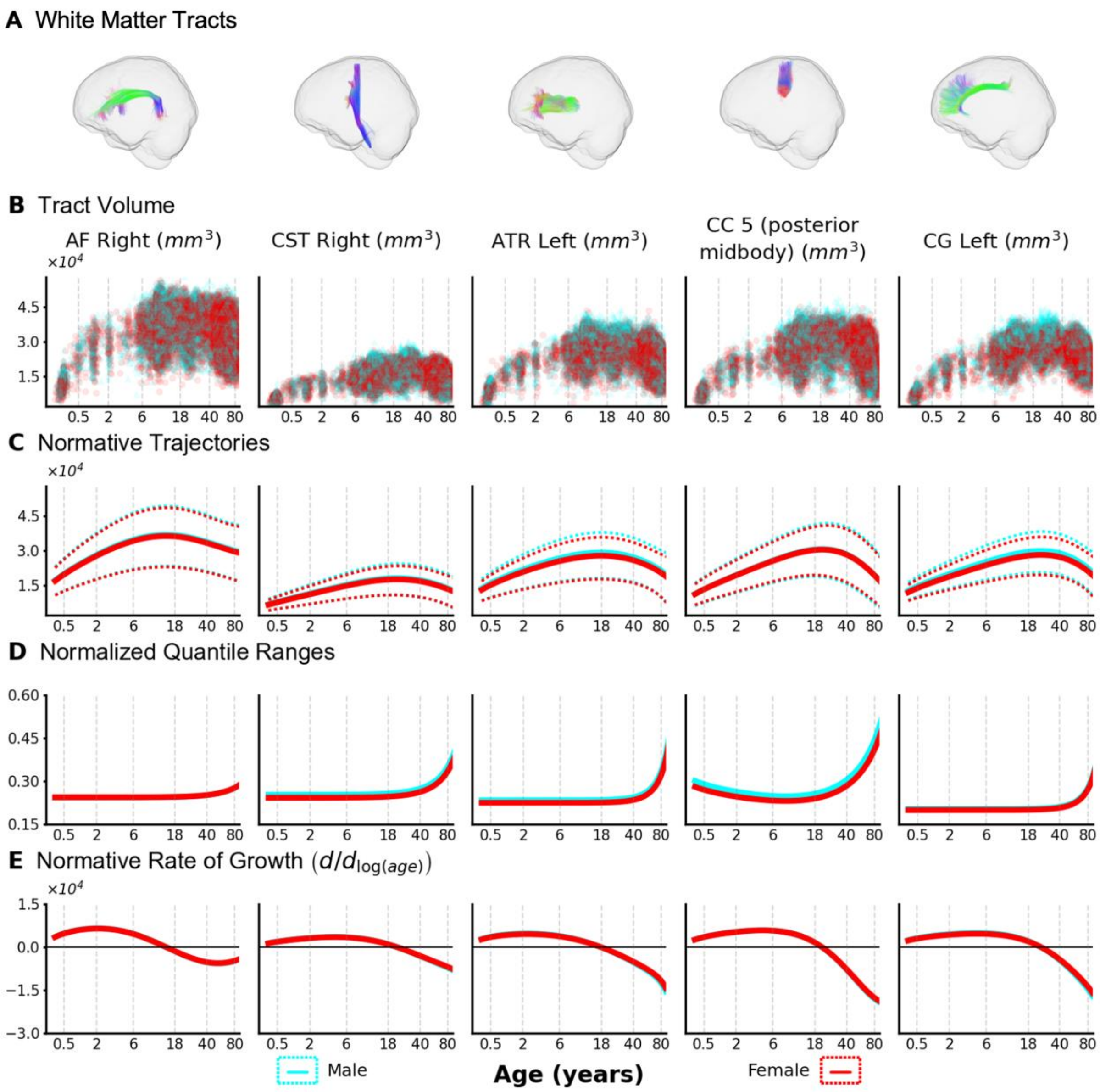

Supplemental Figure SA.F2. Tract-specific Macrostructural Brain Charts - Contralateral. Lifespan brain charts for white matter *macrostructure* (tract volume shown for contralateral tracts of **Figure 3**) reveal distinct developmental trajectories and variability across different WM pathways. A) Five exemplar tracts are shown (left to right): right arcuate fasciculus (AF Right); right corticospinal tract (CST Right); left anterior thalamic radiation (ATR Left); posterior midbody of the corpus callosum (CC 5 (posterior midbody)); and the left cingulate gyrus (CG Left). B) Raw tract volume data points for these tracts span the lifespan, illustrating differences in typical volume ranges between tracts. C) Normative GAMLSS trajectories show tract volume increasing during development, peaking in adolescence or early adulthood, and declining in later life, with tract-specific timing and magnitude. Median (solid lines) and 2.5th/97.5th percentiles (dotted lines) are shown. D) Normalized quantile ranges indicate that volume variability increases later in life and differs between sexes, being generally lowest in younger ages. E) The normative rate of change (first derivative) suggests that tract volume peaks at different ages and the rate of subsequent decline varies depending on the specific tract. Note: The x-axes (age in years) are log-scaled to emphasize developmental and aging periods.

Regarding checking/visualizing ROI-wise centiles: we have written instructions on how to get any desired centile for any pathway and any feature on Zenodo – making these available as readable matrices in the portable and interoperable csv format. However, we agree with the reviewer that sharing the figures of all centiles would be helpful. Thus, we have also released PNGs of every brain chart for all features and all pathways on the Zenodo page at https://zenodo.org/records/17561821.

Regarding population representativeness, please see R2.C1, where we have now explicitly stated this as a limitation.

R2.C15: Supplementary information could have a table of contents for easier navigation, and all elements in the supplementaries should be referenced in the main manuscript. The authors could consider splitting the supplementary materials to supplementary methods and supplementary results.

The available data is summarized nicely in Supplementary Table S1, but it would help the readers to have a PRISMA-style flow chart of the sample building steps, including the total amount of subjects that were excluded at each step for image quality concerns, due to failed tract reconstruction, or demographics, etc.

Supplementary Table S1 could be elaborated by including the DTI sequence parameters, preprocessing (distortion correction), and site location (country, city, state). This could also be done in a separate table(s).

Please review the supplements and online materials for clarity and minor typos.

We have restructured the document to be better organized and included a TOC. Additionally, we have added a flowchart as Supplemental Figure SB.F1.

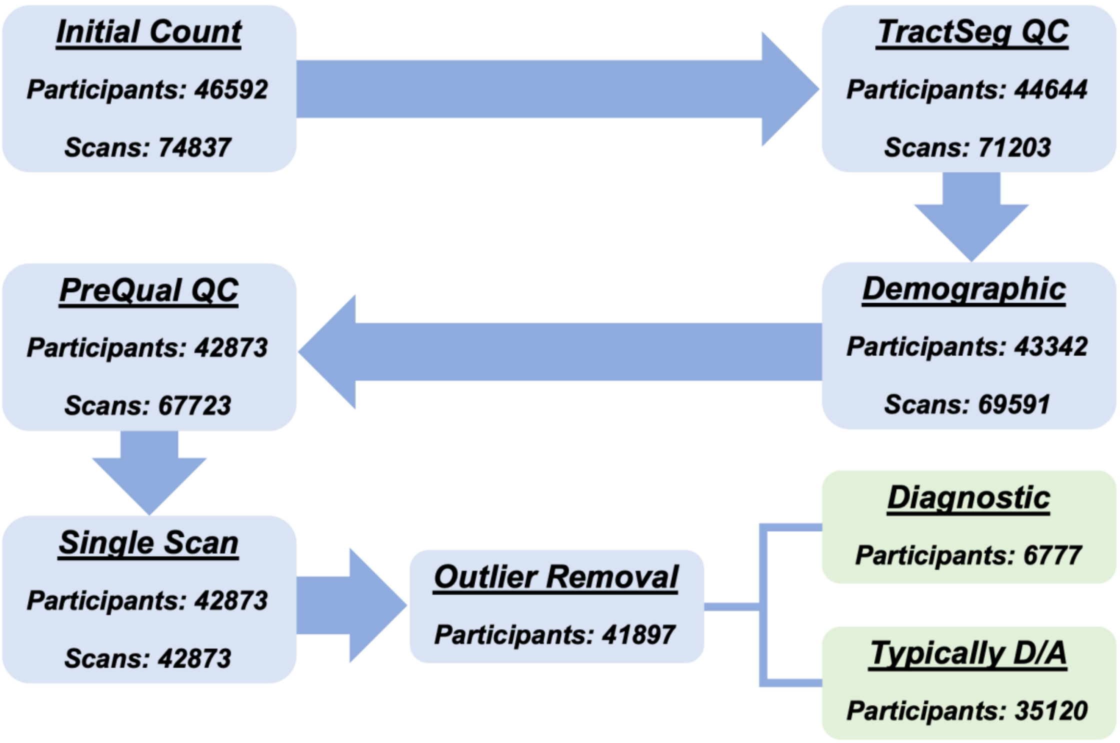

Supplemental Figure SB.F1. Flowchart of participant and scan removal at each stage of quality assurance.

We have additionally included Supplemental Table SB.T3 to the Supplementary Information that summarizes voxel size, acquisition parameters, and facility location:

Dataset	Voxel Size	Acquisition Parameters	Facility Location
ABCD[57]	1.7 x 1.7 x 1.7	7 b0; 6 b500; 15 b1000; 15 b2000; 60 b3000	Varied (USA)
ADNI[58]	2.0 x 2.0 x 2.0	(Varied) 7 b0; 48 b1000	Varied (USA)
Ageility[59]	2.0 x 2.0 x 2.0	1 b0; 64 b1000; 64 b3000	Newcastle, Australia
AOMIC ID1000[54]	2.0 x 2.0 x 2.0	1 b0; 32 b1000 [sequence acquired x3]	Amsterdam, Netherlands
AOMIC PIOP1[54]	2.0 x 2.0 x 2.0	1 b0; 32 b1000	Amsterdam, Netherlands
AOMIC PIOP2[54]	2.0 x 2.0 x 2.0	1 b0; 32 b1000	Amsterdam, Netherlands
BANDA[60]	1.5 x 1.5 x 1.5	28 b0; 190 b1500; 190 b3000	Boston, Massachusetts (USA)
BIOCARD[61]	0.828 x 0.828 x 2.2	1 b0; 32 b700	Baltimore, Maryland (USA)
BLSA[62]	0.8125 x 0.8125 x 2.2$^{\$}$	1 b0; 32 b700	Baltimore, Maryland (USA)
BSNIP1[63]	1.72 x 1.72 x 3.0	1 b0; 32 b1000	Varied (USA)
BSNIP2[64]	2.0 x 2.0 x 2.0	7 b0; 64 b900	Varied (USA)
Calgary[65]	0.78 x 0.78 x 2.2	5 b0; 30 b800	Calgary, Canada
CALM[66]	2.0 x 2.0 x 2.0	5 b0; 64 b1000	Cambridge, England
CAMCAN[67]	2.0 x 2.0 x 2.0	3 b0; 30 b1000; 30 b2000	Cambridge, England
VUMC-ASD	2.5 x 2.5 x 2.5	1 b0; 92 b1600<br>3 b0; 6 b500; 32 b1000; 64 b2000	Nashville, Tennessee (USA)
CUTTING	2.5 x 2.5 x 2.5	1 b0; 60 b2000	Nashville, Tennessee (USA)

dHCP[68]	1.5 x 1.5 x 1.5	20 b0; 64 b400; 88 b1000; 128 b2600	United Kingdom
DLBS[69]	1.75 x 1.75 x 3.0	1 b0; 30 b1000	Dallas, Texas (USA)
EBDT[70]	2.0 x 2.0 x 2.0	7 b0; 42 b1000	Chapel Hill, North Carolina (USA)
HABSHD[71]	1.72 x 1.72 x 2.5	4 b0; 64 b1000 [sequence acquired x3]	Fort Worth, Texas (USA)
HBCD[72]	1.7 x 1.7 x 1.7	22 b0; 12 b500; 24 b1000; 36 b2000; 58 b3000	Varied (USA)
HBN[73]	1.8 x 1.8 x1.8	1 b0; 64 b1000; 64 b2000	Varied (USA)
HCP[74]	1.25 x 1.25 x 1.25	18 b0; 90 b1000; 90 b2000; 90 b3000	St. Louis, Missouri; Minneapolis, Michigan (USA)
HCPA[75]	1.5 x 1.5 x 1.5	28 b0; 186 b1500; 184 b3000	St. Louis, Missouri; Minneapolis, Michigan (USA)
HCPBaby[76]	1.5 x 1.5 x 1.5	7 b0; 18 b500; 24 b1000; 108 b1500; 48 b2000; 68 b2500; 170 b3000	Chapel Hill, North Carolina; Minneapolis, Michigan (USA)
HCPD[77]	1.5 x 1.5 x 1.5	28 b0; 186 b1500; 184 b3000	St. Louis, Missouri; Minneapolis, Michigan (USA)
ABC-Babies	1.78 x 1.78 x2.2	1 b0; 32 b700; 64 b2000	Nashville, Tennessee (USA)
IBIS[78]	2.0 x 2.0 x 2.0	1 b0; multi-shell (b=100-1000 in steps of 100), 2-3 volumes/shell	Varied (USA)
ICBM[79]	1.25 x 1.25 x 2.5	5 b0; 30 b1000	Los Angeles, California (USA)
Lexical[80]	2.0 x 2.0 x 2.0	1 b0; 64 b1000	Chicago, Illinois (USA)
MASiVar[81]	2.14 x 2.14 x 2.2	34 b0; 86 b1000; 112 b2000	Boston, Massachusetts (USA)
MORGAN	2.5 x 2.5 x 2.5	1 b0; 92 b1600	Nashville, Tennessee (USA)
NACC[82]	2.0 x 2.0 x 2.0	(Varied) 1 b0; 64 b1000	Varied (USA)
NKI[83]	2.0 x 2.0 x 2.0	9 b0; 128 b1500	Rockland County, New York (USA)
PING[84]	2.5 x 2.5 x 2.5	1 b0; 32 b1000	Varied (USA)
QTAB[85]	2.0 x 2.0 x 2.0	3 b0; 5 b1000; 15 b3000	Queensland, Australia
ROS[86]	2.0 x 2.0 x 2.0	6 b0; 40 b1000	Chicago, Illinois (USA)
MAP[87]	2.0 x 2.0 x 2.0	6 b0; 40 b1000	Chicago, Illinois (USA)
MARS[88]	2.0 x 2.0 x 2.0	1 b0; 40 b1000	Chicago, Illinois (USA)
SCAN[89]	2.0 x 2.0 x 2.0	13 b0; 6 b500; 48 b1000; 60 b2000	Varied (USA)
SWU[90]	2.0 x 2.0 x 2.0	3 b0; 30 b1000	Chongqing, China
TempleSocial[91,92]	2.0 x 2.0 x 2.0	3 b0; 6 b300; 21 b1000; 24 b2000; 12 b3200; 19 b3300; 61 b5000	Philadelphia, Pennsylvania (USA)
UCLA_LA5c[93]	1.98 x 1.98 x2.0	1 b0; 64 b1000	Los Angeles, California (USA)
UKBB[94]	2.02 x 2.02 x 2	8 b0; 50 b1000; 50 b2000	United Kingdom
UPennRisk[95,96]	1.875 x 1.875 x 2.0	1 b0; 30 b1000	Philadelphia, Pennsylvania (USA)
UTAustin579[97]	2.0 x 2.0 x 4.0	6 b0; 64 b800	Austin, Texas (USA)
VMAP_2.0[98]	2.33 x 2.33 x 2.5	1 b0; 38 b1000; 56 b2000	Nashville, Tennessee (USA)
VMAP[98]	2.0 x 2.0 x 2.0	1 b0; 32 b1000	Nashville, Tennessee (USA)
TN Alzheimer's Project	2.33 x 2.33 x 2.5	1 b0; 38 b1000; 56 b2000	Nashville, Tennessee (USA)
WRAP[99]	0.9375 x 0.9375 x 2.5	8 b0; 40 b1300	Madison, Wisconsin ((USA)

$ BLSA was acquired at 2.2 x 2.2 x 2.2 mm^3, but resampled to 0.8125 x 0.8125 x 2.2 mm^3 in k-space

Lastly, we believe we have fixed typos in the online materials.

R2.C16: 16. The authors provide the tools they used to build the brain charts openly for the scientific community through a containerized (Docker pipeline), available at https://zenodo.org/records/15538472. There are also some instructions for replication and model data sets that users can use to try the tools out.

The authors may have made a typo: r_lifepsan_env.tar >>> r_lifespan_env.tar. They might also think about a more descriptive name that contains other keywords in the container name, such as "dti," "tract," "centile," etc.

It would be important to provide a set of commands to run the recommended preprocessing (PreQual) and Docker containers wrapping the recommended post-processing (Mrtrix, TractSeg, scilpy). The prospective users could then follow the instructions and trust that their processing is using a fixed set of versions of the tools and is thus in line with recommendations.

I would suggest that the authors make all the containerized tools available through Docker Hub. The authors should also ensure that Apptainer works for the tools and provide instructions for setting it up.

The instructions can generally be followed with ease, but it might be beneficial to restructure the sections, e.g., a) installation and trying the installation with the use of model data, b) processing your own data (with example code to run an open access data set from unprocessed data to processed form), c) preparation of the demographics (more examples here for building the tables would not hurt), d) running the models for out-of-sample scoring, and e) creating normative models based on new data sets (with clear guidance on recommended sample size and other demographics).

The online materials might benefit from more examples. For instance, changing the metric (other examples could include looping through the structures):

```
docker run --rm -v $PWD/testout:/OUTPUTS r_lifespan_env python3
/WMLifespan/scripts/output_centile_curves.py AF_left volume
/OUTPUTS/centiles_volume_AF_left.csv

docker run --rm -v $PWD/testout:/OUTPUTS r_lifespan_env python3
/WMLifespan/scripts/output_centile_curves.py AF_left fa-mean
/OUTPUTS/centiles_FA_AF_left.csv
```

Of note, these two commands run from any location that has the output directory, i.e., the provided model data input file MS_dataset.csv is not necessary. Making a version with commands where the 'model data' for creating the centiles is given as an input and not used from the Docker container itself would be useful. Having clear pointers in providing input and output is very important.

Finally, code that was used for this work should be made openly available (as much as possible).

We thank the reviewer for their thorough review of the code, and suggestions for improvements and dissemination. We have addressed each point individually.

1. We agree that renaming the file for the container would be more descriptive. Thus, we have changed this in the Zenodo release to https://zenodo.org/records/17561821.

2. Preprocessing: we released a single Docker image containing all the postprocessing pipelines on Dockerhub at https://hub.docker.com/r/kimm58/wm_lifespan_processing with instructions to install and use the container on a Zenodo page at https://zenodo.org/records/17144461. We do note that we have not included the PreQual preprocessing pipeline in this container because different researchers may prefer a specific dMRI preprocessing procedure or data may already come to researchers preprocessed. However, PreQual is available as a separate container at https://zenodo.org/records/14593034, which contains instructions for the commands to use in order to run the container.

3. Availability of containerized tools. We have released the post-processing pipeline as a Docker image at https://hub.docker.com/r/kimm58/wm_lifespan_processing with instructions to install and use the container on a Zenodo page at https://zenodo.org/records/17144461. The Zenodo page also contains instructions on how to convert the Docker image into an Apptainer/Singularity for usage on high-performace computing clusters, with additional instructions on how to run the Apptainer/Singularity version.

4. Restructuring instruction: We have restructured the sections so that 1.) first is the tutorial section of how to load and run the Docker container with the sample dataset, 2.) next is how to run the container to obtain centile curves, 3.) followed by how to align out of sample datasets (including the data processing and preparation of demographics), and then 4.) finally how to create normative models based on new data sets (located in the Appendix).

We note that all code has been containerized and thus does not require any software installation beyond Docker/Apptainer/Singularity: the container with the brain charts are available at https://zenodo.org/records/17561821, the PreQual container is available at https://zenodo.org/records/14593034, and the postprocessing docker image is available at https://hub.docker.com/r/kimm58/wm_lifespan_processing (instructions for use are available at https://zenodo.org/records/17144461). For each of these three containers, we provide example commands and open access data for running the containers, including the processing and out-of-sample centile scoring. With regard to building the demographics tables, we have altered the instructions on the Zenodo page to increase clarity. Finally, we have provided instructions in the Appendix of the white matter brain charts Zenodo page of how researchers can construct their own normative models using our code, which we also provide in the container.

5. Zenodo instructions: We apologize for the confusion in the Zenodo instructions. The MS_dataset.csv file is indeed not used for obtaining centile curves in our provided tutorial. We have altered the wording on the Zenodo page to make this more clear:

> The `example_centiles.csv` file is the example output file for the centile extraction, while the `OOS_aligned.csv` and `MS_dataset.csv` files are the example output and input files respectively for the out-of-sample alignment.

Furthermore, as the reviewer suggested, we have added instructions for looping through all the structures and obtaining centile curves for all features:

> To obtain the centiles for all tracts and features, loop through all the tractseg tracts and features (using the provided file, `tractseg_tracts.txt`):

```
metrics=("fa-mean" "md-mean" "ad-mean" "rd-mean" "volume" "surface_area"
"avg_length")
cat tract_list.txt | while IFS= read -r tract; do
for metric in "${metrics[@]}"; do
docker run --rm \
-v /home/user/testout:/OUTPUTS \
r_lifespan_env \
python3 /WMLifespan/scripts/output_centile_curves.py \
AF_left volume /OUTPUTS/${tract}_${metric}_centiles.csv
done; done
```

6. Code availability. We have released the dMRI preprocessing Docker (https://zenodo.org/records/14593034), postprocessing Docker (https://hub.docker.com/r/kimm58/wm_lifespan_processing; https://zenodo.org/records/17144461), and Docker as the brain charts along with the OOS alignment process (https://zenodo.org/records/17561821).

Reviewer 3

This manuscript presents a very comprehensive analysis of diffusion MRI data across numerous datasets spanning birth to 100 years. They use this data to provide normative curves of microstructural (FA, MD, AD, RD) and macrostructural (tract volume, surface area, length) white matter development across 72 different brain white matter tracts. They further provide profiles of the timing of maturation across metrics and tracts. Then, they demonstrate the ability of these curves to help identify deviations of brain structure in a variety of example disorders (MCI, Alzheimer's, epilepsy). Finally, they provide an example using out-of-sample data by aligning it to the calculated curves and demonstrating how this can be used for external data.

This is a monumental effort, combining huge amounts of data from around the world into one sample and providing extensive information on normative white matter changes across the entire lifespan. It provides the most comprehensive analysis of white matter changes to date, I imagine will be a valuable resource for many. The supplied code, particularly that enabling out-of-sample datasets to be incorporated, is hugely useful.

The manuscript is well-written and the figures are excellent. Information is clearly conveyed, and the writing follows a very logical progression. Many important points are addressed well, and analyses clearly described.

We are grateful for this positive and thorough appraisal of our work. We appreciated highlighting it as a “monumental effort” and “valuable resource”. We have addressed all comments below, which we believe have strengthened the manuscript, providing analysis and clarifications on sex-stratified trajectories, sources of variability, exclusion of longitudinal data, and data quality across the lifespan.

R3.C1: I have a few questions/comments for improvement: 1. The authors say that they provide “sex-stratified trajectories”. However, in looking at their equation (methods, equations 2 & 3) and results, it appears that only a main effect of sex was included in the models, and trajectories were not allowed to vary between males and females, e.g., “sex was included as a binary fixed effect”. Is that the case? If so, they are missing huge amounts of information that may be gleaned from modeling male and female trajectories separately, as there are most certainly time periods where the shape of trajectories would differ. I would suggest running fully separate models for males and females to more accurately capture development and aging.

We agree with the reviewer that a completely separate modeling of male/female trajectories can in principle capture sex-specific shape differences. For this reference release, we follow prior normative charting practice by including sex as a fixed effect on both location (μ) and dispersion (σ) in GAMLSS, yielding sex-specific centiles while preserving pooled sample size for stable fits and simple out-of-sample (OOS) alignment[36,100,101]. To address the reviewer’s concern, we compared the pooled model (with sex in μ, σ) to fully sex-separated models. For both global and tract-specific metrics, the resulting trajectories are highly similar across most of the lifespan (below, and in supplementary material).

Given (i) the empirical similarity, (ii) edge-age stability and coverage, and (iii) the added complexity sex-specific models would introduce for OOS centile scoring, we retain the pooled specification for the main charts.

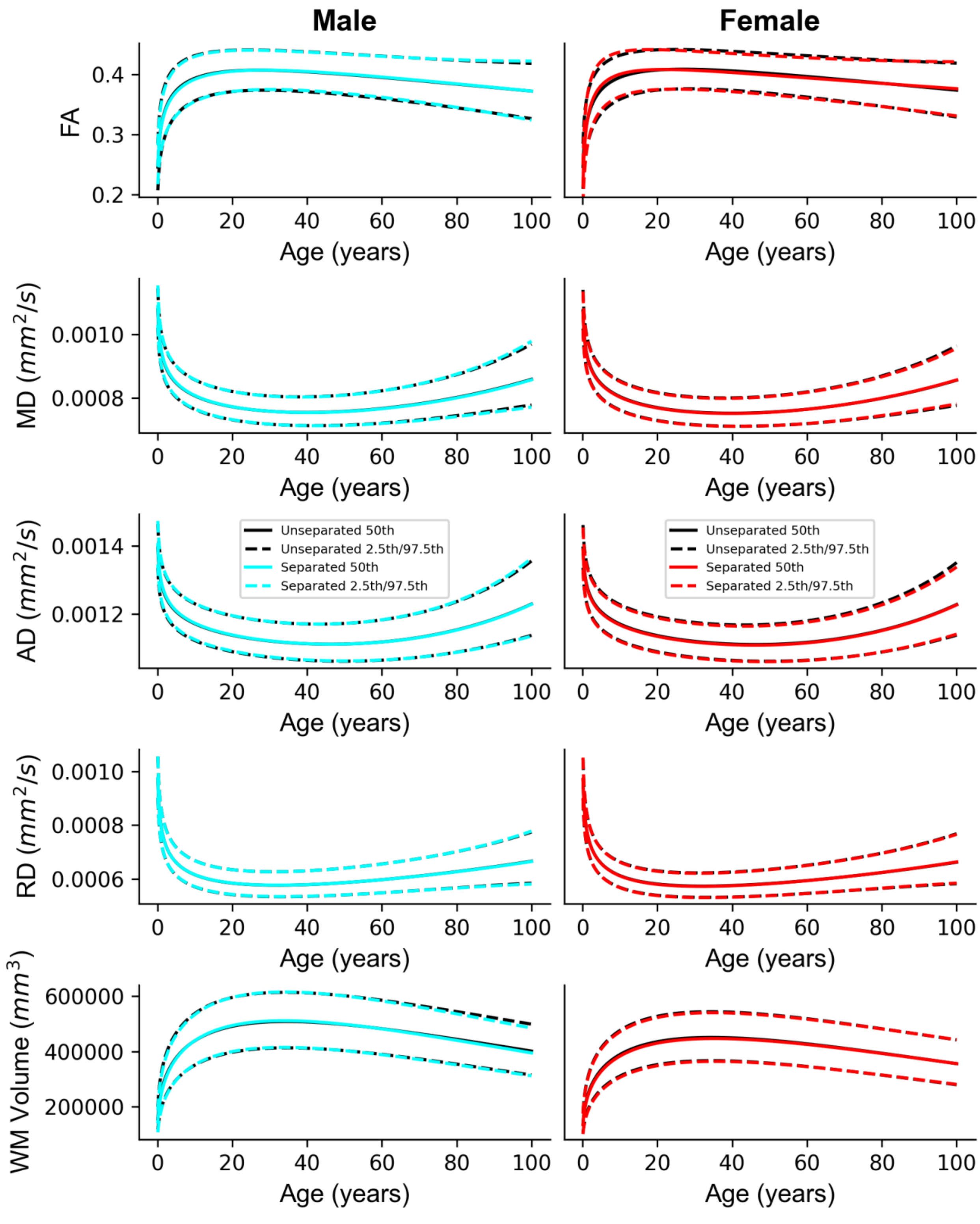

Male
Female
FA
0.2
0.3
0.4
MD (mm^2/s)
0.0008
0.0010
AD (mm^2/s)
0.0012
0.0014
RD (mm^2/s)
0.0006
0.0008
0.0010
WM Volume (mm^3)
200000
400000
600000
0
20
40
60
80
100
Age (years)
Unseparated 50th
Unseparated 2.5th/97.5th
Separated 50th
Separated 2.5th/97.5th

Supplemental Figure SD.F7. Comparison of sex-separated vs unseparated models. Normative models fit with sex as a linear effect (”Unseparated” - black lines) are very similar to models that were fit with male and female populations completely separated (“Separated” – cyan/red lines), indicating that the GAMLSS model fitting for the WM brain charts is appropriately capturing the sex-specific trajectories for microstructure and macrostructure (from top to bottom: global WM features of FA, MD, AD, RD, and volume). Male trajectories are plotted in the left and female trajectories are plotted on the right.

R3.C2: Variability is estimated using quantile ranges and said to increase in adulthood. I wonder if this is related, at least somewhat, to data quality in the older samples, rather than true neurobiological variability. This would be worthwhile commenting on, and testing if possible.

We agree that the reviewer that this point is certainly worth testing. Thus, we examine PreQual image quality metrics (IQMs) for all datasets across the lifespan, comparing distributions at different age ranges (Supplemental Figure SF.F1). We do not find that lower quality data (lower CNR, higher chi-squared, higher motion) are consistently found in aging populations. Furthermore, we do not find centile scores to be strongly correlated with IQMs (Supplemental Figure SF.F2). We have aggregated these results and analyses in the newly added supplementary section titled “Data Quality”:

Data Quality

As mentioned in the main manuscript, we conducted a thorough quality control process that involved visualizing each scan and performing quantitative outlier removal (see Methods: Data Selection). The final results of this process can be found in Supplemental Table S1. Notably, infant datasets tend to have many more scans that are not included in the final participant pool for creating the brain charts. We attribute this effect largely to TractSeg, which was trained on an adult population. As infant data are more out-of-distribution compared to other datasets that the TractSeg models were not trained on, many infant scans showed poor tract reconstruction.

Furthermore, we conducted an analysis to assess both image quality across datasets and how image quality affects downstream centile score estimates. Specifically, we visualized distributions for the PreQual image quality metrics (IQMs) of mean relative displacement, the chi-squared error metric in shells used for diffusion tensor fitting, contrast-to-noise ratio (CNR) as calculated by EDDY for the main shell used for tensor fitting for scans included in creating the brain charts (Supplemental Figure SF.F1). We do not observe IQMs to be influenced by age at time of scan, as distributions are not consistently higher or lower at any given age range. In other words, IQM distribution appeared to be dataset-dependent rather than age-dependent.

For assessing correlation of estimated centile scores with IQMs, we computed Spearman’s correlation coefficients between centile scores and each IQM (Supplemental Figure SF.F2). All correlations were either low or non-existent, with nearly all correlations is below 0.15 and none above 0.19. These results suggest that image quality of the dMRI data does not largely affect the estimated centile scores.

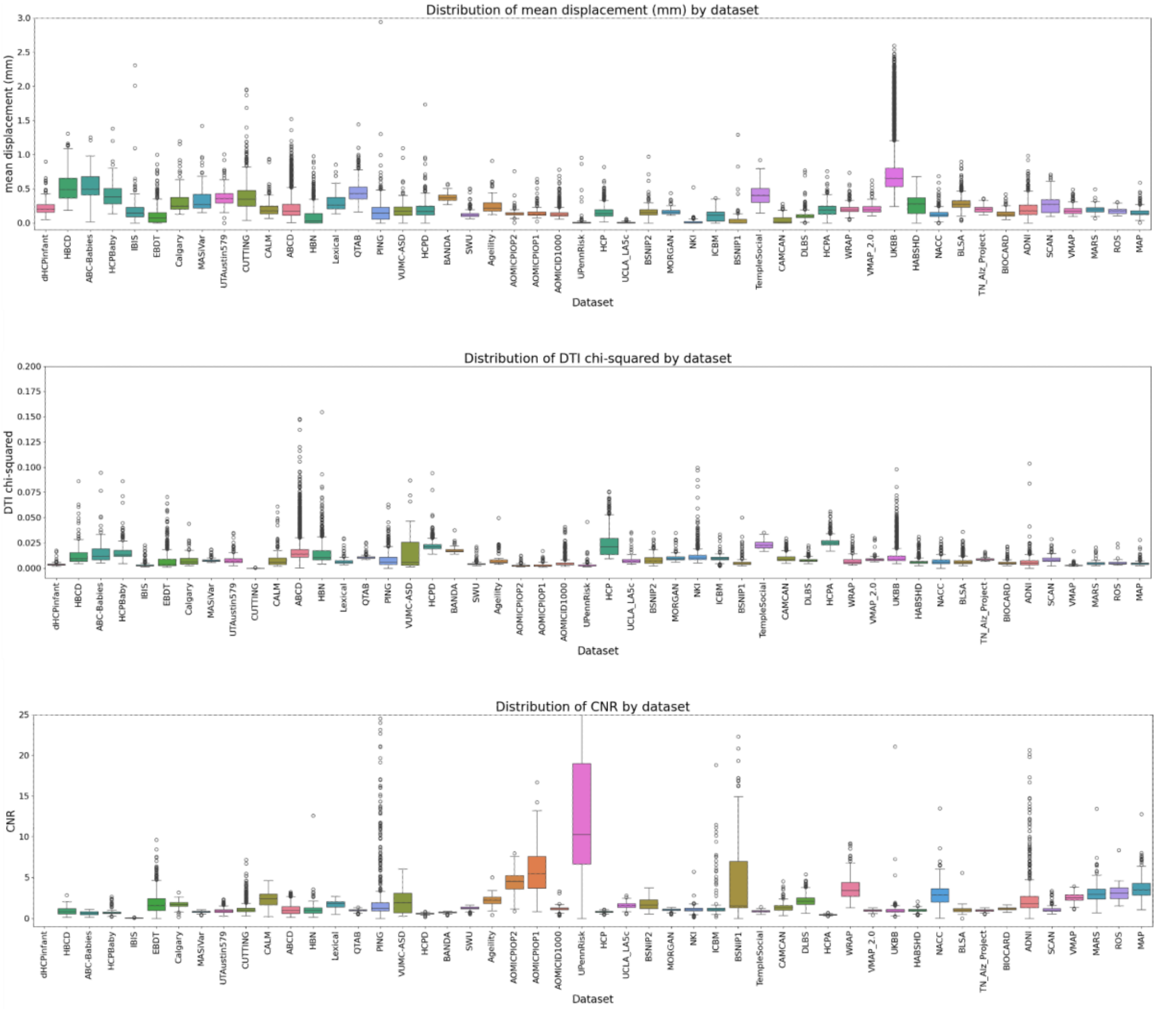

Supplemental Figure SF.F1. Distribution of PreQual image quality metrics by dataset. We do not observe age at time of scans to largely influence image quality metrics (IQMs) for dMRI preprocessing, as IQM distributions are not consistently higher or lower at any age range. Plotted are (top) motion as mean relative displacement, (middle) chi-squared error metric in the shells used for the diffusion tensor imaging fit, and (bottom) contrast to noise ratio (CNR) as calculated by eddy for the main shell used for the tensor fitting. Datasets are ordered by mean age of participants, with the youngest ages on the left and oldest on the right.

Further, our own recent work on both microstructure and macrostructure suggests that modern preprocessing pipelines can effectively mitigate artifacts (motion, in particular) to the point where biases are not detectable with current analysis techniques.[102]

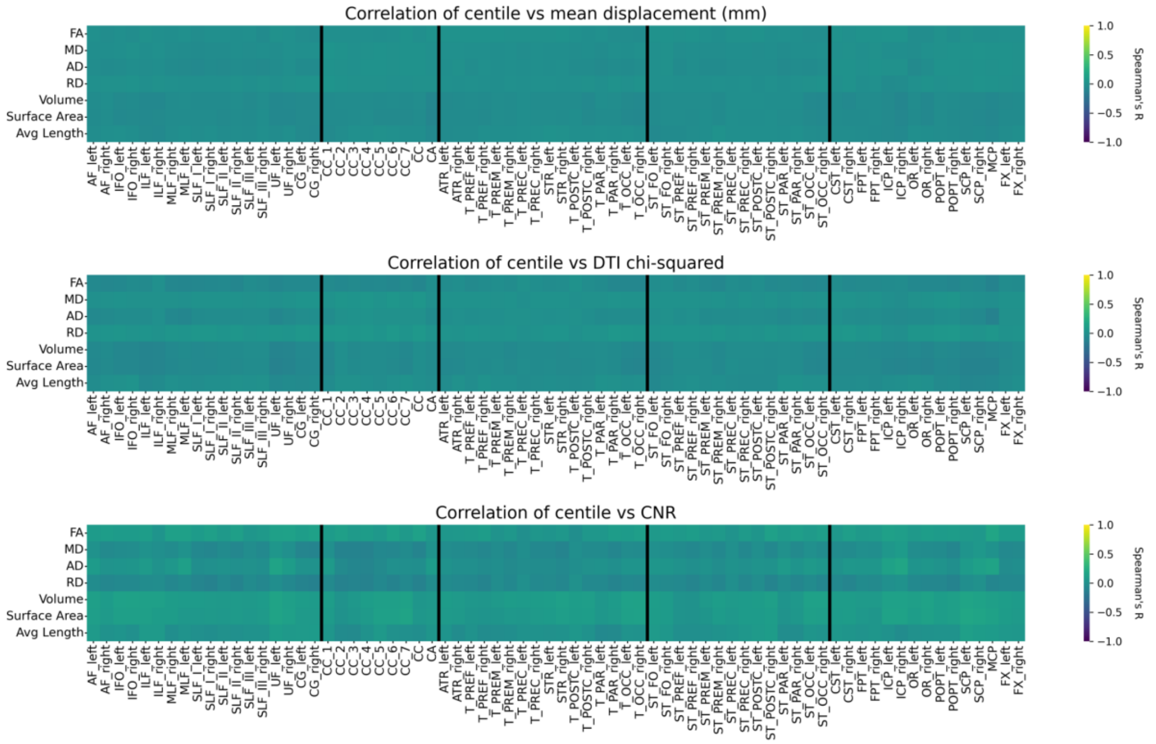

Supplemental Figure SF.F2. Correlation of PreQual image quality metrics with centile scores. We do not observe image quality metrics (IQMs) for dMRI preprocessing to be correlated with observed centile scores, as the absolute value of nearly all correlations is below 0.15, with none above 0.19. Plotted are Spearman's correlation coefficients with (top) motion as mean relative displacement, (middle) chi-squared error metric in the shells used for the diffusion tensor imaging fit, and (bottom) contrast to noise ratio (CNR) as calculated by eddy for the main shell used for the tensor fitting.

R3.C3: The examples given for atypical brains are primarily diseases of aging (MCI, Alzheimers), while epilepsy may span a wider age range. Useful other examples are provided in Figure 5D, but I would appreciate some idea of the age range of participants in these samples. Does the centile scoring work across the age range? Does it perform equally well in young vs old participants? Males vs females? It would be really useful to understand how consistent/fair/useful these predictions are across populations.

We have made two key clarifications in the manuscript to address this.

First, for clinical age ranges: We agree that providing the age ranges for our clinical cohorts is helpful for context. We have added a new panel to Figure 4A that visually displays the age ranges of the diagnostic groups included in our study, situating them alongside typical diagnostic age ranges from the literature

Second, consistency across age and sex: regarding the consistency and fairness of centile scoring, this is a core strength and a primary goal of the GAMLSS normative modeling framework. By design, the centile scores are adjusted for age and sex (and derived directly from age- and sex-adjusted distributions). As defined in our Methods, age (as a non-linear function) and sex are included as fixed effects for both the location and scale of the distribution. A 50th centile score, for example, is inherently specific, meaning "median for a person of this specific age and sex." This ensures that the scores are, by design, appropriate and comparable across all ages and for both males and females. To empirically confirm the stability of these models across the lifespan, we performed a leave-one-study-out (LOSO) bootstrapping analysis (see R2.C7). The coefficient of variation (COV) is very low (below 0.5%) across the vast majority of the lifespan for all our sex-stratified models. This is strong evidence that our models are robust and generate consistent centile scores for participants, whether generated from young or old cohorts.

R3.C4: Why was longitudinal data excluded? Many of these datasets contain longitudinal data, and it seems that it would greatly enhance the predictive power of these curves, not only by increasing the total number of datapoints, but also by increasing the power to model individual trajectories. Further, cross-sectional models can underestimate true change: https://www.pnas.org/doi/10.1073/pnas.2216798120

We agree with the reviewer that this is a critical methodological point. The reviewer is correct that longitudinal data provides the most precise measure of individual change, and as Di Biase et al. demonstrates, cross-sectional models can indeed underestimate true longitudinal trajectories. Our decision to use a cross-sectional approach was a deliberate methodological choice, made after weighing the significant trade-offs, especially given our primary goal of creating a robust, generalizable reference chart with a feasible out-of-sample (OOS) alignment tool.

Incorporating longitudinal data (e.g., via individual-level random effects) presented two major, unsolved challenges for a tool intended for this purpose. First was model Stability: while many of our 50 datasets contained longitudinal scans, the vast majority of our 35,120 participants had only cross-sectional data. More importantly, most longitudinal subjects had too few timepoints (e.g., 2-3) to stably estimate individual-level random effects. This is particularly problematic for a GAMLSS model, where inaccurate variance estimates (due to unstable random effects) would corrupt the centile calculations, which are the primary output of the chart. Second was Out-of-Sample (OOS) Alignment: this was the primary motivator for our cross-sectional analysis. Our current cross-sectional model estimates 2 parameters (location and scale offsets) for a new study. A longitudinal model with individual-level random effects would require estimating 2N parameters for N new subjects. More critically, it is mathematically and conceptually unclear how one would estimate a new, unseen subject's random effect from a single scan - which is the most common use case for a normative chart. This would require developing novel, complex methods (e.g., integrating out the random effect) that are a significant research project in their own right.

Given these constraints, we chose the robust and feasible cross-sectional approach, following the precedent of Bethlehem et al., to ensure a stable model and a usable OOS tool.

As you noted from our previous discussion (R1.C6), we have explicitly acknowledged this in our limitations section and added the full justification you see here to the supplementary material ("Considerations for Cross-sectional vs Longitudinal Brain Charts") .

<u>Considerations for Cross-sectional vs Longitudinal Brain Charts</u>

Previous work has shown that cross-sectional normative models can be inaccurate in predicting measured longitudinal trajectories[7], as longitudinal models can better separate true developmental change from generational differences that exist in the data and cross-sectional data may be unrepresentative of individuals. Although many of our datasets contained longitudinal data, there were several challenges we encountered and aspects about the normative models we needed to consider for longitudinal brain charts[103]. First, despite the subset of datasets with participant data at multiple time points, for the majority of participants in the study we only have cross-sectional data available. Incorporating longitudinal data would require estimation of random effects for each participant, introducing significant computational complexity in the model. We found that we did not have enough data points to stably estimate individual-level random effects. Furthermore, having to estimate these individual-level random effects would greatly decrease the stability and increase complexity of the out-of-sample centile scoring process, as alignment with N participants would require estimation of $2N$ terms instead of only 2 terms with the cross-sectional models. These constraints and considerations led us to develop a robust cross-sectional approach that maintains generalizability and computational feasibility. We acknowledge the potential benefits of longitudinal modeling and suggest this as an important avenue for future research.

R3.C5: The pipelines used for processing here are designed for adults (except infant freesurfer), and can have known issues in infants, young children, or elderly participants. Can the authors please comment on data quality across the lifespan in this dataset?

We thank the reviewer for this important consideration. We have added a new "Data Quality" section to the Supplementary Information to address this directly , but we will elaborate on our rationale here. We view this challenge as two separate components: (1) dMRI preprocessing and (2) tractography segmentation.

Preprocessing (PreQual):

Our preprocessing pipeline, PreQual, is not based on age-specific biological models but on the fundamental physics of MR imaging. It corrects for universal artifacts like motion, eddy-current distortions, and susceptibility-induced distortions. These correction principles are valid for any dMRI scan, even beyond human models and in systems outside the brain. The primary challenge, is that artifacts like motion are more prevalent in infant and elderly populations. This is precisely why we implemented a multi-stage quality control process. As shown in Supplemental Table SB.T1 , we do have a higher prevalence of infant data that did not pass QA, but still have large, quality, samples across all ages (indicated by the image quality metric (IQM) distributions in Supplemental Figure SF.F1). Supplemental Figure SF.F2 demonstrates that centile scores are not correlated with these IQMs, attesting to our pipeline's robustness.

Segmentation (TractSeg):

The reviewer is correct that TractSeg was trained on a young adult population. While this is a limitation, TractSeg has been shown to be highly robust and has been widely applied in studies across the entire lifespan, from infancy to aging[30,104–112], and has been shown to be robust for individuals with a variety of brain pathologies[113–124]. In our study, we did observe a higher rate of tract reconstruction failure in the infant datasets, as detailed in Supplemental Table SB.T1. These scans were appropriately excluded by our QA process. While the development of population-specific tractography atlases and tools is an important avenue for future research [125], the combination of a robust tool (TractSeg) and strict QA gives us confidence in the resulting segmentations.

We believe that the data informing our charts are of high quality and not systematically biased by age-related acquisition challenges.

R3.C6: Bethlehem et al.'s brain charts include white matter volume, so statements like "previous efforts did not extend to the study of WM" should be changed. Note that this doesn't negate the importance of this work – I just suggest rewording these statements for accuracy.

We agree with the reviewer that our previous statement is misleading, and thus have modified it to be more appropriate:

> … However, in the context of WM, these previous efforts were predominantly limited to global volumetric analyses and did not provide the tract-specific, pathway-level phenotypes needed to localize deviations and to relate white-matter architecture to circuit function and dysfunction.

R3.C7: Introduction, 2nd paragraph, "WM specific traits may be even more critical for clinical studies" – in what way? I'm not sure what this sentence means.

We agree with the reviewer that the claim is too far-reaching and an over-generalization. Thus, we have removed this sentence from the manuscript.

R3.C8: Introduction, 3rd paragraph: "…largely due to methodological challenges of dMRI". I think this does not really follow from the first part of the sentence. There are many ways to quantify WM without dMRI. I would revise this.

Thank you for this comment. We have amended the text to improve the logical flow of the paragraph:

R3.C9: p. 9, "AD generally peaked later than other features, suggesting prolonged maturation of myelin-sensitive properties" AD is generally associated with axonal change, rather than myelin. Can the authors clarify what they mean here?

Again, this may be an over-generalization (and a challenge of interpreting these sensitive measures that lack specificity). Other features, such as RD, would be more suitable for assessing myelination. To improve the manuscript based on this suggestion, we have altered Figure 4 to assess myelination in the context of RD.

R3.C10: "scans were excluded if key demographic information – … cognitive status – was missing." What does this mean and how was it assessed?

Thank you for the opportunity to improve clarity regarding the exclusion of data. For any given dataset, we may have imaging data present for participants, even though demographic information of age, sex, or diagnosis is absent from the dataset metadata information. In these instances, we remove the participants from the analysis. While we cannot comment on why these variables are missing, given that they were released by 3rd parties, we have rewritten this section for better clarity:

> Participant scans were excluded if key demographic information - age, sex, or cognitive status - was missing or not reported for that participant.

R3.C11: I noted in the online docker code that out-of-sample data requires at least some typical controls to be used. This is understandable, but would be useful to mention in the paper, since I know there are many researchers out there with clinical datasets looking for readily available control comparisons.

We have added the following text to the manuscript:

> We note that as the brain charts were constructed with typically aging and developing individuals, aligning out-of-sample datasets requires some typically aging and developing individuals in the out-of-sample dataset as well (See Supplementary Material: "Out-of-sample Alignment Analysis").

R3.C12: I have reviewed the code and the online documentation. I did not install and try running the code due to limitations of the computer I'm currently using. The documentation online is excellent, and provides clear instructions for installing and running the code to complete analysis. It also provides a tutorial dataset, which appears to be a useful resource for those learning how to use this code.

Thank you for the kind assessment of our efforts, we appreciate your comment and agree that making tools open source is an important part of scientific reproducibility.

References

[1] Yakolev PI, Lecours A-R. The myelogenetic cycles of regional maturation of the brain, pp 3-70. Oxford: Blackwell. In: Minkowski A, editor. Regional development of the brain in early life, Oxford; 1967, p. 3–70.

[2] Flechsig P. Developmental (myelogenetic) localisation of the cerebral cortex in the human subject. The Lancet 1901;158:1027–30.

[3] de Faria O, Pivonkova H, Varga B, Timmler S, Evans KA, Káradóttir RT. Periods of synchronized myelin changes shape brain function and plasticity. Nat Neurosci 2021;24:1508–21. https://doi.org/10.1038/S41593-021-00917-2.

[4] Solmi M, Radua J, Olivola M, Croce E, Soardo L, Salazar de Pablo G, et al. Age at onset of mental disorders worldwide: large-scale meta-analysis of 192 epidemiological studies. Mol Psychiatry 2022;27:281–95. https://doi.org/10.1038/S41380-021-01161-7;SUBJMETA.

[5] Li M, Santpere G, Kawasawa YI, Evgrafov O V., Gulden FO, Pochareddy S, et al. Integrative functional genomic analysis of human brain development and neuropsychiatric risks. Science (1979) 2018;362. https://doi.org/10.1126/SCIENCE.AAT7615.

[6] Hendrickson MA, Pitt MB. Three Areas Where Our Growth Chart Conversations Fall Short-Room to Grow. JAMA Pediatr 2022;176:123–4. https://doi.org/10.1001/JAMAPEDIATRICS.2021.4330.

[7] Di Biase MA, Tian YE, Bethlehem RAI, Seidlitz J, Alexander-Bloch AF, Thomas Yeo BT, et al. Mapping human brain charts cross-sectionally and longitudinally. Proc Natl Acad Sci U S A 2023;120:e2216798120. https://doi.org/10.1073/PNAS.2216798120/SUPPL_FILE/PNAS.2216798120.SAPP.PDF.

[8] Grummer-Strawn LM, Reinold C, Krebs NF. Use of World Health Organization and CDC growth charts for children aged 0-59 months in the United States. MMWR Recomm Report 2010.

[9] Zhu AH, Nir TM, Javid S, Villalón-Reina JE, Rodrigue AL, Strike LT, et al. Lifespan reference curves for harmonizing multi-site regional brain white matter metrics from diffusion MRI. Scientific Data 2025 12:1 2025;12:1–18. https://doi.org/10.1038/s41597-025-05028-2.

[10] Jahanshad N, Kochunov P V., Sprooten E, Mandl RC, Nichols TE, Almasy L, et al. Multi-site genetic analysis of diffusion images and voxelwise heritability analysis: A pilot project of the ENIGMA–DTI working group. Neuroimage 2013;81:455–69. https://doi.org/10.1016/J.NEUROIMAGE.2013.04.061.

[11] Villalón-Reina JE, Zhu AH, Benavidez S, Moreau CA, Feng Y, Chattopadhyay T, et al. Lifespan Normative Modeling of Brain Microstructure. BioRxiv 2024:2024.12.15.628527. https://doi.org/10.1101/2024.12.15.628527.

[12] Storsve AB, Fjell AM, Yendiki A, Walhovd KB. Longitudinal Changes in White Matter Tract Integrity across the Adult Lifespan and Its Relation to Cortical Thinning. PLoS One 2016;11:e0156770. https://doi.org/10.1371/journal.pone.0156770.

[13] Giorgio A, Santelli L, Tomassini V, Bosnell R, Smith S, De Stefano N, et al. Age-related changes in grey and white matter structure throughout adulthood. Neuroimage 2010;51:943–51. https://doi.org/10.1016/j.neuroimage.2010.03.004.
[14] Beck D, de Lange A-MG, Maximov II, Richard G, Andreassen OA, Nordvik JE, et al. White matter microstructure across the adult lifespan: A mixed longitudinal and cross-sectional study using advanced diffusion models and brain-age prediction. Neuroimage 2021;224:117441. https://doi.org/10.1016/j.neuroimage.2020.117441.
[15] Henriques RN, Henson R, Correia MM. Unique information from common diffusion MRI models about white-matter differences across the human adult lifespan. Imaging Neuroscience 2023;1:1–25. https://doi.org/10.1162/imag_a_00051.
[16] Toschi N, Gisbert RA, Passamonti L, Canals S, De Santis S. Multishell diffusion imaging reveals sex-specific trajectories of early white matter degeneration in normal aging. Neurobiol Aging 2020;86:191–200. https://doi.org/10.1016/j.neurobiolaging.2019.11.014.
[17] Lebel C, Gee M, Camicioli R, Wieler M, Martin W, Beaulieu C. Diffusion tensor imaging of white matter tract evolution over the lifespan. Neuroimage 2012;60:340–52. https://doi.org/10.1016/j.neuroimage.2011.11.094.
[18] Slater DA, Melie-Garcia L, Preisig M, Kherif F, Lutti A, Draganski B. Evolution of white matter tract microstructure across the life span. Hum Brain Mapp 2019;40:2252–68. https://doi.org/10.1002/hbm.24522.
[19] Yeatman JD, Wandell BA, Mezer AA. Lifespan maturation and degeneration of human brain white matter. Nat Commun 2014;5:4932. https://doi.org/10.1038/ncomms5932.
[20] Groves AR, Smith SM, Fjell AM, Tamnes CK, Walhovd KB, Douaud G, et al. Benefits of multi-modal fusion analysis on a large-scale dataset: Life-span patterns of inter-subject variability in cortical morphometry and white matter microstructure. Neuroimage 2012;63:365–80. https://doi.org/10.1016/j.neuroimage.2012.06.038.
[21] Hoagey DA, Rieck JR, Rodrigue KM, Kennedy KM. Joint contributions of cortical morphometry and white matter microstructure in healthy brain aging: A partial least squares correlation analysis. Hum Brain Mapp 2019;40:5315–29. https://doi.org/10.1002/hbm.24774.
[22] Kochunov P, Glahn DC, Lancaster J, Thompson PM, Kochunov V, Rogers B, et al. Fractional anisotropy of cerebral white matter and thickness of cortical gray matter across the lifespan. Neuroimage 2011;58:41–9. https://doi.org/10.1016/j.neuroimage.2011.05.050.
[23] Bender AR, Raz N. Normal-appearing cerebral white matter in healthy adults: mean change over 2 years and individual differences in change. Neurobiol Aging 2015;36:1834–48. https://doi.org/10.1016/j.neurobiolaging.2015.02.001.
[24] Sexton CE, Walhovd KB, Storsve AB, Tamnes CK, Westlye LT, Johansen-Berg H, et al. Accelerated Changes in White Matter Microstructure during Aging: A Longitudinal Diffusion Tensor Imaging Study. The Journal of Neuroscience 2014;34:15425–36. https://doi.org/10.1523/JNEUROSCI.0203-14.2014.
[25] Sala S, Agosta F, Pagani E, Copetti M, Comi G, Filippi M. Microstructural changes and atrophy in brain white matter tracts with aging. Neurobiol Aging 2012;33:488-498.e2. https://doi.org/10.1016/j.neurobiolaging.2010.04.027.
[26] Westlye LT, Walhovd KB, Dale AM, Bjornerud A, Due-Tonnessen P, Engvig A, et al. Life-Span Changes of the Human Brain White Matter: Diffusion Tensor Imaging (DTI) and Volumetry. Cerebral Cortex 2010;20:2055–68. https://doi.org/10.1093/cercor/bhp280.

[27] Billiet T, Vandenbulcke M, Mädler B, Peeters R, Dhollander T, Zhang H, et al. Age-related microstructural differences quantified using myelin water imaging and advanced diffusion MRI. Neurobiol Aging 2015;36:2107–21. https://doi.org/10.1016/j.neurobiolaging.2015.02.029.

[28] Molloy CJ, Nugent S, Bokde ALW. Alterations in Diffusion Measures of White Matter Integrity Associated with Healthy Aging. The Journals of Gerontology: Series A 2021;76:945–54. https://doi.org/10.1093/gerona/glz289.

[29] Ardekani S, Kumar A, Bartzokis G, Sinha U. Exploratory voxel-based analysis of diffusion indices and hemispheric asymmetry in normal aging. Magn Reson Imaging 2007;25:154–67. https://doi.org/10.1016/j.mri.2006.09.045.

[30] Schilling KG, Chad JA, Chamberland M, Nozais V, Rheault F, Archer D, et al. White matter tract microstructure, macrostructure, and associated cortical gray matter morphology across the lifespan. Imaging Neuroscience 2023;1:1–24. https://doi.org/10.1162/IMAG_A_00050/118326/WHITE-MATTER-TRACT-MICROSTRUCTURE-MACROSTRUCTURE.

[31] Conte S, Zimmerman D, Richards JE. White matter trajectories over the lifespan. PLoS One 2024;19. https://doi.org/10.1371/JOURNAL.PONE.0301520.

[32] Schilling KG, Tax CMW, Rheault F, Landman BA, Anderson AW, Descoteaux M, et al. Prevalence of white matter pathways coming into a single white matter voxel orientation: The bottleneck issue in tractography. Hum Brain Mapp 2022;43:1196–213. https://doi.org/10.1002/HBM.25697;PAGE:STRING:ARTICLE/CHAPTER.

[33] Maier-Hein KH, Neher PF, Houde JC, Côté MA, Garyfallidis E, Zhong J, et al. The challenge of mapping the human connectome based on diffusion tractography. Nature Communications 2017 8:1 2017;8:1–13. https://doi.org/10.1038/s41467-017-01285-x.

[34] Schilling KG, Rheault F, Petit L, Hansen CB, Nath V, Yeh FC, et al. Tractography dissection variability: What happens when 42 groups dissect 14 white matter bundles on the same dataset? Neuroimage 2021;243:118502. https://doi.org/10.1016/J.NEUROIMAGE.2021.118502.

[35] Bhagwat N, Barry A, Dickie EW, Brown ST, Devenyi GA, Hatano K, et al. Understanding the impact of preprocessing pipelines on neuroimaging cortical surface analyses. Gigascience 2021;10:giaa155. https://doi.org/10.1093/GIGASCIENCE/GIAA155.

[36] Bethlehem RAI, Seidlitz J, White SR, Vogel JW, Anderson KM, Adamson C, et al. Brain charts for the human lifespan. Nature 2022 604:7906 2022;604:525–33. https://doi.org/10.1038/s41586-022-04554-y.

[37] Courchesne E, Chisum HJ, Townsend J, Cowles A, Covington J, Egaas B, et al. Normal brain development and aging: Quantitative analysis at in vivo MR imaging in healthy volunteers. Radiology 2000;216:672–82. https://doi.org/10.1148/RADIOLOGY.216.3.R00AU37672.

[38] Cai LY, Yang Q, Hansen CB, Nath V, Ramadass K, Johnson GW, et al. PreQual: An automated pipeline for integrated preprocessing and quality assurance of diffusion weighted MRI images. Magn Reson Med 2021;86:456–70. https://doi.org/10.1002/mrm.28678.

[39] Tournier JD, Smith R, Raffelt D, Tabbara R, Dhollander T, Pietsch M, et al. MRtrix3: A fast, flexible and open software framework for medical image processing and

visualisation. Neuroimage 2019;202:116137. https://doi.org/10.1016/J.NEUROIMAGE.2019.116137.
[40] Jenkinson M, Beckmann CF, Behrens TEJ, Woolrich MW, Smith SM. FSL. Neuroimage 2012;62:782–90. https://doi.org/10.1016/J.NEUROIMAGE.2011.09.015.
[41] Schilling KG, Blaber J, Huo Y, Newton A, Hansen C, Nath V, et al. Synthesized b0 for diffusion distortion correction (Synb0-DisCo). Magn Reson Imaging 2019;64:62–70. https://doi.org/10.1016/J.MRI.2019.05.008.
[42] Tang L, Zhou XJ. Diffusion MRI of cancer: From low to high b-values. Journal of Magnetic Resonance Imaging 2019;49:23–40. https://doi.org/10.1002/JMRI.26293.
[43] Novikov DS, Fieremans E, Jespersen SN, Kiselev VG. Quantifying brain microstructure with diffusion MRI: Theory and parameter estimation. NMR Biomed 2019;32. https://doi.org/10.1002/NBM.3998,.
[44] Theaud G, Houde JC, Boré A, Rheault F, Morency F, Descoteaux M. TractoFlow: A robust, efficient and reproducible diffusion MRI pipeline leveraging Nextflow & Singularity. Neuroimage 2020;218:116889. https://doi.org/10.1016/J.NEUROIMAGE.2020.116889.
[45] Wasserthal J, Neher P, Maier-Hein KH. TractSeg - Fast and accurate white matter tract segmentation. Neuroimage 2018;183:239–53. https://doi.org/10.1016/J.NEUROIMAGE.2018.07.070.
[46] Fischl B. FreeSurfer. Neuroimage 2012;62:774–81. https://doi.org/10.1016/j.neuroimage.2012.01.021.
[47] Zöllei L, Iglesias JE, Ou Y, Grant PE, Fischl B. Infant FreeSurfer: An automated segmentation and surface extraction pipeline for T1-weighted neuroimaging data of infants 0–2 years. Neuroimage 2020;218:116946. https://doi.org/10.1016/J.NEUROIMAGE.2020.116946.
[48] Veraart J, Christiaens D, Dai E, Edwards LJ, Golkov V, Mohammadi S, et al. A data-driven variability assessment of brain diffusion MRI preprocessing pipelines. Proceedings of the Joint Annual ISMRM-ESMRMB Meeting, 2022.
[49] Dubos J, Park SK, Vlasova R, Prieto JC, Styner M. Dmriprep: open-source diffusion MRI quality control framework with graphical user interface. Https://DoiOrg/101117/122654470 2023;12464:759–63. https://doi.org/10.1117/12.2654470.
[50] Irfanoglu MO, Nayak A, Jenkins J, Pierpaoli C. TORTOISEv3: Improvements and New Features of the NIH Diffusion MRI Processing Pipeline. 25th Annual Meeting of The International Society for Magnetic Resonance in Medicine, Honolulu: 2017.
[51] Yeh FC. DSI Studio: an integrated tractography platform and fiber data hub for accelerating brain research. Nature Methods 2025 22:8 2025;22:1617–9. https://doi.org/10.1038/s41592-025-02762-8.
[52] Leemans A, Jeurissen B, Sijbers J, Jones DK. ExploreDTI: A Graphical Toolbox for Processing, Analyzing, and Visualizing Diffusion MR Data. 17th Annual Meeting of The International Society for Magnetic Resonance in Medicine, Honolulu, HI: 2009.
[53] Oguz I, Farzinfar M, Matsui J, Budin F, Liu Z, Gerig G, et al. DTIPrep: quality control of diffusion-weighted images. Front Neuroinform 2014;8:4. https://doi.org/10.3389/fninf.2014.00004.
[54] Snoek L, van der Miesen MM, Beemsterboer T, van der Leij A, Eigenhuis A, Steven Scholte H. The Amsterdam Open MRI Collection, a set of multimodal MRI datasets for

individual difference analyses. Sci Data 2021;8:85. https://doi.org/10.1038/s41597-021-00870-6.
[55] Huang S, Zhong L, Shi Y. Automated Mapping of Residual Distortion Severity in Diffusion MRI. Computational Diffusion MRI : MICCAI Workshop 2024;14328:58. https://doi.org/10.1007/978-3-031-47292-3_6.
[56] Forkel SJ, Friedrich P, Thiebaut de Schotten M, Howells H. White matter variability, cognition, and disorders: a systematic review. Brain Struct Funct 2021;227:529. https://doi.org/10.1007/S00429-021-02382-W.
[57] Casey BJ, Cannonier T, Conley MI, Cohen AO, Barch DM, Heitzeg MM, et al. The Adolescent Brain Cognitive Development (ABCD) study: Imaging acquisition across 21 sites. Dev Cogn Neurosci 2018;32:43–54. https://doi.org/10.1016/J.DCN.2018.03.001.
[58] Weber CJ, Carrillo MC, Jagust W, Jack CR, Shaw LM, Trojanowski JQ, et al. The Worldwide Alzheimer’s Disease Neuroimaging Initiative: ADNI-3 updates and global perspectives. Alzheimer’s & Dementia: Translational Research & Clinical Interventions 2021;7:e12226. https://doi.org/10.1002/TRC2.12226.
[59] Karayanidis F, Keuken MC, Wong A, Rennie JL, de Hollander G, Cooper PS, et al. The Age-ility Project (Phase 1): Structural and functional imaging and electrophysiological data repository. Neuroimage 2016;124:1137–42. https://doi.org/10.1016/J.NEUROIMAGE.2015.04.047.
[60] Siless V, Hubbard NA, Jones R, Wang J, Lo N, Bauer CCC, et al. Image acquisition and quality assurance in the Boston Adolescent Neuroimaging of Depression and Anxiety study. Neuroimage Clin 2020;26:102242. https://doi.org/10.1016/J.NICL.2020.102242.
[61] Miller MI, Younes L, Ratnanather JT, Brown T, Trinh H, Lee DS, et al. Amygdalar atrophy in symptomatic Alzheimer’s disease based on diffeomorphometry: the BIOCARD cohort. Neurobiol Aging 2015;36:S3–10. https://doi.org/10.1016/J.NEUROBIOLAGING.2014.06.032.
[62] Ferrucci L. The Baltimore Longitudinal Study of Aging (BLSA): A 50-Year-Long Journey and Plans for the Future. The Journals of Gerontology: Series A 2008;63:1416–9. https://doi.org/10.1093/GERONA/63.12.1416.
[63] Kristian Hill S, Reilly JL, Keefe RSE, Gold JM, Bishop JR, Gershon ES, et al. Neuropsychological impairments in schizophrenia and psychotic bipolar disorder: findings from the Bipolar-Schizophrenia Network on Intermediate Phenotypes (B-SNIP) study. Am J Psychiatry 2013;170:1275–84. https://doi.org/10.1176/APPI.AJP.2013.12101298.
[64] Parker DA, Trotti RL, McDowell JE, Keedy SK, Keshavan MS, Pearlson GD, et al. Differentiating biomarker features and familial characteristics of B-SNIP psychosis Biotypes. Translational Psychiatry 2025 15:1 2025;15:1–10. https://doi.org/10.1038/s41398-025-03501-5.
[65] Reynolds J, Long X, Paniukov D, Bagshawe M, Dewey D, Lebel C. Calgary Preschool MRI Dataset 2019. https://doi.org/10.17605/OSF.IO/AXZ5R.
[66] Holmes J, Bryant A, Gathercole SE. Protocol for a transdiagnostic study of children with problems of attention, learning and memory (CALM). BMC Pediatrics 2019 19:1 2019;19:1–11. https://doi.org/10.1186/S12887-018-1385-3.
[67] Shafto MA, Tyler LK, Dixon M, Taylor JR, Rowe JB, Cusack R, et al. The Cambridge Centre for Ageing and Neuroscience (Cam-CAN) study protocol: a cross-sectional, lifespan, multidisciplinary examination of healthy cognitive ageing. BMC Neurol 2014;14. https://doi.org/10.1186/S12883-014-0204-1.

[68] Edwards AD, Rueckert D, Smith SM, Abo Seada S, Alansary A, Almalbis J, et al. The Developing Human Connectome Project Neonatal Data Release. Front Neurosci 2022;16:886772. https://doi.org/10.3389/FNINS.2022.886772/BIBTEX.
[69] Park DC, Hennessee JP, Smith ET, Chan MY, Chen X, Dakanali M, et al. The Dallas Lifespan Brain Study: A Comprehensive Adult Lifespan Data Set of Brain and Cognitive Aging. Scientific Data 2025 12:1 2025;12:1–14. https://doi.org/10.1038/s41597-025-04847-7.
[70] Chen Y, Zhu H, Shen D, An H, Gilmore J, Lin W. Mapping Growth Patterns and Genetic Influences on Early Brain Development in Twins. Med Image Comput Comput Assist Interv 2009;12:232. https://doi.org/10.1007/978-3-642-04271-3_29.
[71] O'Bryant SE, Johnson LA, Barber RC, Braskie MN, Christian B, Hall JR, et al. The Health & Aging Brain among Latino Elders (HABLE) study methods and participant characteristics. Alzheimer's & Dementia: Diagnosis, Assessment & Disease Monitoring 2021;13. https://doi.org/10.1002/dad2.12202.
[72] Nelson CA, Frankeberger J, Chambers CD. An introduction to the HEALthy Brain and Child Development Study (HBCD) study. Dev Cogn Neurosci 2024;69:101441. https://doi.org/10.1016/J.DCN.2024.101441.
[73] Richie-Halford A, Cieslak M, Ai L, Caffarra S, Covitz S, Franco AR, et al. An analysis-ready and quality controlled resource for pediatric brain white-matter research. Scientific Data 2022 9:1 2022;9:1–27. https://doi.org/10.1038/s41597-022-01695-7.
[74] Van Essen DC, Ugurbil K, Auerbach E, Barch D, Behrens TEJ, Bucholz R, et al. The Human Connectome Project: A data acquisition perspective. Neuroimage 2012;62:2222–31. https://doi.org/10.1016/j.neuroimage.2012.02.018.
[75] Bookheimer SY, Salat DH, Terpstra M, Ances BM, Barch DM, Buckner RL, et al. The Lifespan Human Connectome Project in Aging: An overview. Neuroimage 2019;185:335–48. https://doi.org/10.1016/J.NEUROIMAGE.2018.10.009.
[76] Howell BR, Styner MA, Gao W, Yap PT, Wang L, Baluyot K, et al. The UNC/UMN Baby Connectome Project (BCP): An overview of the study design and protocol development. Neuroimage 2019;185:891–905. https://doi.org/10.1016/j.neuroimage.2018.03.049.
[77] Harms MP, Somerville LH, Ances BM, Andersson J, Barch DM, Bastiani M, et al. Extending the Human Connectome Project across ages: Imaging protocols for the Lifespan Development and Aging projects. Neuroimage 2018;183:972–84. https://doi.org/10.1016/j.neuroimage.2018.09.060.
[78] Wolff JJ, Gu H, Gerig G, Elison JT, Styner M, Gouttard S, et al. Differences in white matter fiber tract development present from 6 to 24 months in infants with autism. Am J Psychiatry 2012;169:589–600. https://doi.org/10.1176/APPI.AJP.2011.11091447.
[79] Mazziotta JC, Woods R, Iacoboni M, Sicotte N, Yaden K, Tran M, et al. The myth of the normal, average human brain—The ICBM experience: (1) Subject screening and eligibility. Neuroimage 2009;44:914–22. https://doi.org/10.1016/j.neuroimage.2008.07.062.
[80] Lytle MN, McNorgan C, Booth JR. A longitudinal neuroimaging dataset on multisensory lexical processing in school-aged children. Scientific Data 2019 6:1 2019;6:1–12. https://doi.org/10.1038/s41597-019-0338-5.

[81] Cai LY, Yang Q, Kanakaraj P, Nath V, Newton AT, Edmonson HA, et al. MASiVar: Multisite, multiscanner, and multisubject acquisitions for studying variability in diffusion weighted MRI. Magn Reson Med 2021;86:3304–20. https://doi.org/10.1002/MRM.28926.
[82] Beekly DL, Ramos EM, Lee WW, Deitrich WD, Jacka ME, Wu J, et al. The National Alzheimer's Coordinating Center (NACC) database: The uniform data set. Alzheimer Dis Assoc Disord 2007;21:249–58. https://doi.org/10.1097/WAD.0B013E318142774E.
[83] Nooner KB, Colcombe SJ, Tobe RH, Mennes M, Benedict MM, Moreno AL, et al. The NKI-Rockland sample: A model for accelerating the pace of discovery science in psychiatry. Front Neurosci 2012;6:32787. https://doi.org/10.3389/FNINS.2012.00152/BIBTEX.
[84] Jernigan TL, Brown TT, Hagler DJ, Akshoomoff N, Bartsch H, Newman E, et al. The Pediatric Imaging, Neurocognition, and Genetics (PING) Data Repository. Neuroimage 2015;124:1149. https://doi.org/10.1016/J.NEUROIMAGE.2015.04.057.
[85] Strike LT, Hansell NK, Chuang KH, Miller JL, de Zubicaray GI, Thompson PM, et al. The Queensland Twin Adolescent Brain Project, a longitudinal study of adolescent brain development. Scientific Data 2023 10:1 2023;10:1–18. https://doi.org/10.1038/s41597-023-02038-w.
[86] Bennett DA, Schneider JA, Arvanitakis Z, Wilson RS. OVERVIEW AND FINDINGS FROM THE RELIGIOUS ORDERS STUDY. Curr Alzheimer Res 2012;9:628. https://doi.org/10.2174/156720512801322573.
[87] Bennett DA, Schneider JA, Buchman AS, De Leon CM, Bienias JL, Wilson RS. The Rush Memory and Aging Project: Study Design and Baseline Characteristics of the Study Cohort. Neuroepidemiology 2005;25:163–75. https://doi.org/10.1159/000087446.
[88] Barnes LL, Shah RC, Aggarwal NT, Bennett DA, Schneider JA. The Minority Aging Research Study: Ongoing Efforts to Obtain Brain Donation in African Americans without Dementia. Curr Alzheimer Res 2012;9:734. https://doi.org/10.2174/156720512801322627.
[89] SCAN n.d. https://scan.naccdata.org/ (accessed November 2, 2025).
[90] SLIM n.d. https://doi.org/10.15387/fcp_indi.retro.slim.
[91] Smith D V., Sharp CJ, Dachs A, Wyngaarden J, Sazhin D, Yang Y, et al. Social reward and nonsocial reward processing across the adult lifespan: An interim multi-echo fMRI and diffusion dataset. Data Brief 2024;56:110810. https://doi.org/10.1016/J.DIB.2024.110810).
[92] Social Reward and Nonsocial Reward Processing Across the Adult Lifespan: An Interim Multi-echo fMRI and Diffusion Dataset - OpenNeuro n.d. https://openneuro.org/datasets/ds005123/versions/1.1.3 (accessed January 13, 2025).
[93] UCLA Consortium for Neuropsychiatric Phenomics LA5c Study - OpenNeuro n.d. https://openneuro.org/datasets/ds000030/versions/1.0.0 (accessed January 13, 2025).
[94] Alfaro-Almagro F, Jenkinson M, Bangerter NK, Andersson JLR, Griffanti L, Douaud G, et al. Image processing and Quality Control for the first 10,000 brain imaging datasets from UK Biobank. Neuroimage 2018;166:400–24. https://doi.org/10.1016/J.NEUROIMAGE.2017.10.034.
[95] Cognitive Training - OpenNeuro n.d. https://openneuro.org/datasets/ds002843/versions/1.0.1 (accessed January 13, 2025).
[96] Kable JW, Caulfield MK, Falcone M, McConnell M, Bernardo L, Parthasarathi T, et al. No effect of commercial cognitive training on brain activity, choice behavior, or cognitive

performance. Journal of Neuroscience 2017;37:7390–402. https://doi.org/10.1523/JNEUROSCI.2832-16.2017.

[97] Wang J, Lytle MN, Weiss Y, Yamasaki BL, Booth JR. A longitudinal neuroimaging dataset on language processing in children ages 5, 7, and 9 years old. Sci Data 2022;9. https://doi.org/10.1038/S41597-021-01106-3.

[98] Jefferson AL, Gifford KA, Acosta LMY, Bell SP, Donahue MJ, Davis LT, et al. The Vanderbilt Memory & Aging Project: Study Design and Baseline Cohort Overview. Journal of Alzheimer's Disease 2016;52:539–59. https://doi.org/10.3233/JAD-150914.

[99] Sager MA, Hermann B, La Rue A. Middle-aged children of persons with Alzheimer's disease: APOE genotypes and cognitive function in the Wisconsin Registry for Alzheimer's Prevention. J Geriatr Psychiatry Neurol 2005;18:245–9. https://doi.org/10.1177/0891988705281882.

[100] Ge R, Yu Y, Qi YX, Fan Y nan, Chen S, Gao C, et al. Normative modelling of brain morphometry across the lifespan with CentileBrain: algorithm benchmarking and model optimisation. Lancet Digit Health 2024;6:e211–21. https://doi.org/10.1016/S2589-7500(23)00250-9.

[101] Habes M, Pomponio R, Shou H, Doshi J, Mamourian E, Erus G, et al. The Brain Chart of Aging: Machine-learning analytics reveals links between brain aging, white matter disease, amyloid burden, and cognition in the iSTAGING consortium of 10,216 harmonized MR scans. Alzheimer's and Dementia 2021;17:89–102. https://doi.org/10.1002/ALZ.12178,.

[102] Schilling KG, Ramadass K, Sairanen V, Kim ME, Rheault F, Newlin N, et al. Head Motion in Diffusion Magnetic Resonance Imaging: Quantification, Mitigation, and Structural Associations in Large, Cross-Sectional Datasets Across the Lifespan. Hum Brain Mapp 2025;46. https://doi.org/10.1002/HBM.70143.

[103] Cole TJ. Sample size and sample composition for constructing growth reference centiles. Stat Methods Med Res 2020;30:488. https://doi.org/10.1177/0962280220958438.

[104] Sutter EN, Guerrero-Gonzalez J, Casey CP, Dean DC, de Abreu e Gouvea A, Peyton C, et al. White-Matter Connectivity and General Movements in Infants with Perinatal Brain Injury. Brain Sci 2025;15:341. https://doi.org/10.3390/BRAINSCI15040341/S1.

[105] Kojima K, Kline JE, Altaye M, Kline-Fath BM, Parikh NA, Allahverdy A, et al. Corpus Callosum Abnormalities at Term-Equivalent Age Are Associated with Language Development at 2 Years' Corrected Age in Infants Born Very Preterm. The Journal of Pediatrics: Clinical Practice 2024;11:200101. https://doi.org/10.1016/J.JPEDCP.2024.200101.

[106] Verschuur AS, Tax CMW, Nijholt IM, van Wezel-Meijler G, Hendson L, Zein H, et al. Diffusion MRI tractography with along-tract profiling reveals subtle neurodevelopmental differences between moderate and late preterm infants. Eur J Radiol 2025;187:112098. https://doi.org/10.1016/j.ejrad.2025.112098.

[107] Schilling KG, Archer D, Yeh F-C, Rheault F, Cai LY, Hansen C, et al. Aging and white matter microstructure and macrostructure: a longitudinal multi-site diffusion MRI study of 1218 participants. Brain Struct Funct 2022;227:2111–25. https://doi.org/10.1007/s00429-022-02503-z.

[108] Tinney EM, Warren AEL, Ai M, Morris TP, O'Brien A, Odom H, et al. Understanding Cognitive Aging Through White Matter: A Fixel-Based Analysis. Hum Brain Mapp 2024;45:e70121. https://doi.org/10.1002/HBM.70121.

[109] Trofimova O, Latypova A, DiDomenicantonio G, Lutti A, de Lange AMG, Kliegel M, et al. Topography of associations between cardiovascular risk factors and myelin loss in the ageing human brain. Communications Biology 2023 6:1 2023;6:1–14. https://doi.org/10.1038/s42003-023-04741-1.
[110] Meisler SL, Gabrieli JDE. Fiber-specific structural properties relate to reading skills in children and adolescents. Elife 2022;11. https://doi.org/10.7554/ELIFE.82088.
[111] Mukherjee M, Hyde C, Barhoun P, Bianco KM, Singh M, Waugh J, et al. White matter organisation of sensorimotor tracts is associated with motor imagery in childhood. Brain Struct Funct 2024;229:1591. https://doi.org/10.1007/S00429-024-02813-4.
[112] Lima Santos JP, Soehner AM, Ladouceur CD, Versace A. The Impact of Insufficient Sleep on White Matter Development in Late Childhood and Early Adolescence. Journal of Adolescent Health 2025;76:220–7. https://doi.org/10.1016/J.JADOHEALTH.2024.10.007.
[113] Moshe YH, Ben Bashat D, Hananis Z, Teicher M, Artzi M. Utilizing the TractSeg Tool for Automatic Corticospinal Tract Segmentation in Patients With Brain Pathology. Technol Cancer Res Treat 2022;21:15330338221131388. https://doi.org/10.1177/15330338221131387.
[114] Tu S, Wang C, Menke RAL, Talbot K, Barnett M, Kiernan MC, et al. Regional callosal integrity and bilaterality of limb weakness in amyotrophic lateral sclerosis. Amyotroph Lateral Scler Frontotemporal Degener 2020;21:396–402. https://doi.org/10.1080/21678421.2020.1733020;REQUESTEDJOURNAL:JOURNAL:IAFD20;WGROUP:STRING:PUBLICATION.
[115] D'Mello AM, Frosch IR, Meisler SL, Grotzinger H, Perrachione TK, Gabrieli JDE. Diminished Repetition Suppression Reveals Selective and Systems-Level Face Processing Differences in ASD. Journal of Neuroscience 2023;43:1952–62. https://doi.org/10.1523/JNEUROSCI.0608-22.2023.
[116] Wasserthal J, Maier-Hein KH, Neher PF, Wolf RC, Northoff G, Waddington JL, et al. White matter microstructure alterations in cortico-striatal networks are associated with parkinsonism in schizophrenia spectrum disorders. European Neuropsychopharmacology 2021;50:64–74. https://doi.org/10.1016/J.EURONEURO.2021.04.007.
[117] Wasserthal J, Maier-Hein KH, Neher PF, Northoff G, Kubera KM, Fritze S, et al. Multiparametric mapping of white matter microstructure in catatonia. Neuropsychopharmacology 2020 45:10 2020;45:1750–7. https://doi.org/10.1038/s41386-020-0691-2.
[118] Zhang P, Gu G, Duan Y, Zhuo Z, Pan C, Zuo P, et al. White matter alterations in pediatric brainstem glioma: An national brain tumor registry of China study. Front Neurosci 2022;16:986873. https://doi.org/10.3389/FNINS.2022.986873/BIBTEX.
[119] Neher P, Hirjak D, Maier-Hein K. Radiomic tractometry reveals tract-specific imaging biomarkers in white matter. Nature Communications 2024 15:1 2024;15:1–11. https://doi.org/10.1038/s41467-023-44591-3.
[120] Anijärv TE, Ossenkoppele R, Smith R, Pichet Binette A, Collij LE, Behjat HH, et al. Hemispheric asymmetry of tau pathology is related to asymmetric amyloid deposition in Alzheimer's Disease. Nature Communications 2025 16:1 2025;16:1–15. https://doi.org/10.1038/s41467-025-63564-2.

[121] Dewenter A, Jacob MA, Cai M, Gesierich B, Hager P, Kopczak A, et al. Disentangling the effects of Alzheimer's and small vessel disease on white matter fibre tracts. Brain 2023;146:678–89. https://doi.org/10.1093/BRAIN/AWAC265.
[122] Andica C, Kamagata K, Uchida W, Takabayashi K, Shimoji K, Kaga H, et al. White matter fiber-specific degeneration in older adults with metabolic syndrome. Mol Metab 2022;62:101527. https://doi.org/10.1016/J.MOLMET.2022.101527.
[123] Hyde C, Fuelscher I, Sciberras E, Efron D, Anderson VA, Silk T. Understanding motor difficulties in children with ADHD: A fixel-based analysis of the corticospinal tract. Prog Neuropsychopharmacol Biol Psychiatry 2021;105:110125. https://doi.org/10.1016/J.PNPBP.2020.110125.
[124] Fuelscher I, Hyde C, Anderson V, Silk TJ. White matter tract signatures of fiber density and morphology in ADHD. Cortex 2021;138:329–40. https://doi.org/10.1016/J.CORTEX.2021.02.015.
[125] Descoteaux M, Schilling KG, Aydogan DB, Beaulieu C, Borra E, Chamberland M, et al. Millennium Pathways for Tractography: 40 grand challenges to shape the future of tractography. Shawna Farquharson 2025;10:23.

Reviewer 1

R1.C1: Thank you for revising your manuscript thoughtfully. I enjoyed reading it again and have one final comment:

In Figure 6 and its accompanying margin text, the authors state that centile scores are used to predict variability in "physiological, behavioral, or functional measurements". However, the only external validation presented in Figure 6C relates to cognitive performance. Cognition represents a specific subclass of behavioural/functional outcomes and is not equivalent to physiological or functional measures more broadly.

This creates a mismatch between the stated scope and the demonstrated example. The authors are encouraged to clarify the terminology used in the margin text and figure captions, explicitly specify the cognitive measure employed (test name or composite), and temper the generality of the claim unless additional non-cognitive physiological or functional variables are shown. As currently presented, Figure 6C should be framed as an illustrative cognition-specific example rather than evidence for broad physiological or functional prediction.

We agree that the wording was broader than the specific cognition-focused example shown in Fig. 6C. We have revised the caption, and accompanying Results/Discussion to make clear that panel C is an illustrative cognition-specific example and avoid implying this example tested physiological/functional measures.

Results:

> …Second, the centile framework enabled associations between WM structure and cognitive performance to be examined, identifying pathway-specific features most strongly linked to clinical variability…

Figure 6 caption:

> …Aligned centile scores serve as standardized metrics to investigate relationships with external variables; here we provide an illustrative cognition-specific example. Executive function (composite score [1]) is significantly correlated with aligned FA centiles in the right SLF III (left plot, N=160), but not the right CST (right plot, N=151) for CN participants, highlighting pathway-specific associations…

Discussion:

> As an illustrative example with Alzheimer's disease cohorts, these aligned centile scores provide a robust foundation for exploring the relationship between WM features and clinical or cognitive outcomes.

Reviewer 2

R2.C1: The authors have addressed my previous comments thoroughly and effectively. I have a few remaining suggestions that I hope will help further refine the manuscript and are necessary to address.

1. Please ensure that all statistical tests performed are clearly stated, effect sizes are reported, and symbols used in the text and figures are explained. For group comparisons, specify the sample composition and sample sizes for each group (e.g., disease vs. controls). This applies to both the main manuscript and the supplementary materials.

We have altered all figure captions to include information regarding the exact statistical tests that were run, as well as the p-value thresholds and information about multiple comparison correction that were used. We have identified all symbols (e.g. significance with "*", boxplot elements) used as well.

Regarding effect sizes, for the diagnostic group centile score deviations, we report the effect sizes as rank-biserial magnitudes in the Supplemental Information section "Effect Sizes of Centile Score Deviations". We also provide the signed distances to the normative 50th centile for each of these comparisons as CSV files (Supporting Data Table SDT.T8). For the development vs aging analyses, we similarly provide supporting CSV files for the identified timepoints of each normative curve as well as the slopes of each identified age range (Supporting Data Tables SDT.T4, SDT.T5).

R2.C2: The main manuscript should note that sex differences in microstructure are generally less pronounced than in macrostructure, though they do exist. Consider referencing the excellent supplementary analyses that provide detailed insights into this point.

Thank you for the suggestion; we have added the following sentence to address this point:

> Furthermore, although sex differences in microstructural measurements are less pronounced than in macrostructure, we still observe significant effects for both (Supplemental Figure SD.F6).

R2.C3: Figure 4 and related results: What is the primary conclusion regarding these alternative hypotheses? If both patterns occur frequently, consider reporting this across all measures and brain regions. Additionally, revise the discussion section to replace the phrase, testing the hypotheses, with language that reflects the main conclusions drawn from your analyses.

We clarified the primary conclusion of the Figure 4D analyses in the Results and Figure 4 caption. Specifically, macrostructural trajectories are consistent with gain predicts loss, whereas timing-based milestone relationships do not support a last in first out pattern.

Results:

Because the charts yield tract-specific milestones, they also enable quantitative assessment of links between maturation and later-life change (Figure 4d). Under a retrogenesis framework [2], we evaluated two tract-level hypotheses derived from centile trajectories. First (based on relative timings), the last in first out hypothesis predicts that later maturing pathways show earlier onset of decline [3]; operationally, we related each tract's age at feature maturity to the age at which decline begins (illustrated for RD). We did not find evidence supporting last in first out in these timing estimates; instead, later maturing pathways tended to show later onset of decline. Second (based on relative rates of change), gain predicts loss predicts that pathways with larger developmental gains show steeper aging-related losses [4]; operationally, we related adolescent growth rates to later life decline rates (illustrated for tract volume). Macrostructural trajectories were consistent with gain predicts loss, with faster adolescent volumetric expansion associated with steeper volumetric decline in aging (parallel regressions for additional WM features shown in **Supplemental Figure SD.F9**). Taken together, these results indicate that centile trajectories capture systematic, feature-dependent couplings between maturation and later-life change.

Figure 4 caption:

Neurobiological hypothesis tests derived from tract milestone timings and rates. Left (timing): positive relationship between age at feature maturity and age at decline onset (RD shown). Right (rates): gain-predicts-loss - greater adolescent growth in macrostructure predicts steeper decline with aging (slope–slope relation; volume shown).

We also added Supplemental Figure SD.F9 to report the corresponding regressions across all WM features:

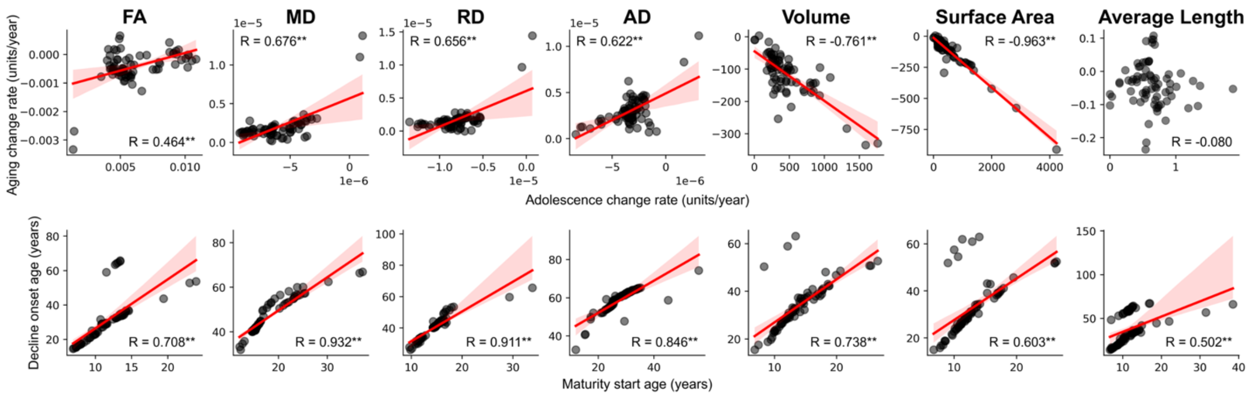

Supplemental Figure SD.F9. White matter charts can be used to test several theories that relate development to aging. These theories include: (1) hypotheses related to the rates of development versus rates of decline (top row), such as the classic gain-predicts-loss theory (i.e., regions which develop most rapidly will decline most rapidly), and the opposing slowest-peak-first-out theory (i.e., regions that develop at the slowest rate are most vulnerable to aging and deteriorate more rapidly); and (2) hypotheses related to developmental timing (bottom row), including the first-in-last-out theory (i.e., brain regions which develop first are the last to degenerate). Microstructural features (FA/MD/AD/RD) show statistically significant relationships between development and aging rates, providing support for the slowest-peak-first-out hypothesis (e.g., pathways with the slowest change in FA during development experienced the fastest decline in aging). Conversely, macrostructural features support the classic gain-predicts-loss hypothesis (e.g., pathways that develop at the fastest rate also decline at the fastest rate). However, our results on developmental timing (bottom row) do not confirm a first-in-last-out theory; rather, they suggest that tracts that reach maturity first are also those that begin to decline first. We note, however, that testing these timing models is complicated by the challenge of defining discrete developmental time points across continuous curves, as well as the ambiguity in the literature regarding whether 'first-in' refers to prenatal emergence versus lifespan maturation. Pearson correlation coefficients are reported for each association, with "**" indicating significance of the association under a least squares linear regression following Bonferroni correction ($p < 0.0035$). For significant associations, regression lines are plotted in red, with 95% confidence intervals shaded pink.

R2.C4: Figure 6: Currently, Figure 6 reads more as an overview of potential applications rather than presenting results. It could serve both purposes. Please revise to present the data in a style consistent with other figures, including details on sample characteristics, statistical tests, and effect sizes. This figure and corresponding text could also highlight that N = 100 is sufficient to estimate centiles for new datasets.

We apologize for the confusion on our end regarding Figure 6, which is intended to provide a demonstrable example of how alignment of out-of-sample datasets is possible and how this alignment process enables investigation of relationships between white matter and other variables of interest (where we have chosen to illustrate this process with cognitive scores). We have changed the figure caption to incorporate the suggested alterations (as well as editorial suggestions):

Figure 6. Applications of Out-of-Sample (OOS) Centile Scoring. Normative white matter brain charts enable standardized centile scoring for new OOS datasets, facilitating harmonized analyses. Exemplar applications are demonstrated using the ADNI dataset (withheld from the models used for this figure for demonstration purposes). A) OOS datasets can be aligned to the reference trajectories using maximum likelihood estimation (MLE) on typically developing/aging participants to estimate study-specific offsets. This process yields a correctly centered centile distribution for OOS controls (N=591 for the mean FA of CC_1). Before and after alignment centile values are shown using a violin/boxplot visualization of median line; interquartile range (IQR); and 1.5*IQR whiskers, with a dotted black line representing the 50th centile. We recommend a sample size of N>100 for alignment based on empirical assessments (see Supplemental Figure SH.F1). B) Aligned centile scores allow for standardized comparisons between diagnostic groups within the OOS dataset, illustrated here comparing typically developing/aging, or cognitively normal (CN, N=591), individuals and those with Alzheimer's disease (AD, N=212). A "*" denotes significance under a Mann-Whitney U test, with $p<0.05$. C) Aligned centile scores serve as standardized metrics to investigate relationships with external variables, such as cognitive performance. The example shows cognitive score (executive function) is significantly correlated with aligned FA centiles in the right SLF III (left plot, N=160), but not the right CST (right plot, N=151) for CN participants, highlighting pathway-specific associations. The statistical test represented is significance of the *centile ~ executive_function* association under a least squares linear regression. Significance ($p<0.025$) of the association is indicated with a red regression line and 95% CIs.

R2.C5: The rationale for tract selection is well justified in the response letter. Consider including this logic in the manuscript or figure legends, noting that the displayed tracts are those most frequently used in ROI-based studies. Figures could also mention the classical directional coloring convention (e.g., blue for inferior-to-superior).

We agree that including such details in the text would improve transparency and readability, and thus have added the following text to manuscript:

> Selection of visualized tracts is based on prevalence of study in the literature [5] (and when displayed in figures are colored to highlight directionality, with red, green, and blue indicating left/right, anterior/posterior, and superior/inferior orientation) [6].

R2.C6: The improvements to shared code and pipelines are excellent. For completeness, please also share the GAMLSS model code. This could for instance be a script for running GAMLSS on one brain area and several metrics of interest and visualizing the results. Please ensure all links are active. I was unable to access the chart plots across all brain areas.

Thank you for noting this. We have now shared the GAMLSS model-fitting code alongside the existing pipelines, including an example script/notebook that runs GAMLSS for a single tract/region across multiple WM metrics and reproduces the corresponding trajectory/centile visualizations.

In addition, we have updated the manuscript links to point to the current Zenodo release (using the Zenodo record/DOI that resolves to the latest version), rather than a prior version-specific link. The Zenodo release also includes the full set of chart plots across all brain areas, provided at the downloadable archive.

R2.C7: Abstract Wording: The phrase "...35,120 diffusion MRI scans from 50 harmonized studies" may be misleading. Consider rephrasing to "50 studies processed using harmonized methods" or similar.

Thank you for the suggestion. We have chosen to keep the original phrasing (in the abstract), because, although we agree with the suggestion, we have had to remove words to meet word-limits criteria for the abstract, so have now kept the more concise "50 harmonized studies". However, we are now clearer in the text for the methods:

> Notably, the 50 included datasets were harmonized through standardized preprocessing, postprocessing, and feature-wise statistical harmonization using generalized additive models for location, scale, and shape (GAMLSS).

R2.C8: Supplemental Figure SE.F2, consider elaborating on expected differences relative to Zhu et al. The authors define DTI tensor values from central and peripheral regions, which explains the lower FA values, which would be good mention.

Thank you for the suggestion. We have added the following text to the Supplemental Figure SE.F2 caption:

> …Compared to our method, which contains information from both central and peripheral WM regions, the TBSS method places a larger focus on central WM, and specifically the central white matter skeleton defined by the highest FA within the sheet-like (or cylinder-like) white matter pathways.

R2.C9: Supplemental Figure SB.F3: Please clarify whether the FreeSurfer mask is shown as an underlay or overlay (the larger image should be the underlay). Indicate whether the visualization represents a single participant or a summary across participants.

We apologize for the ambiguity in the visualization. Both images represent data from a single participant/scan, not aggregate population data. Please note that we have remade Supplemental Figure SB.F3 to better represent the covered regions:

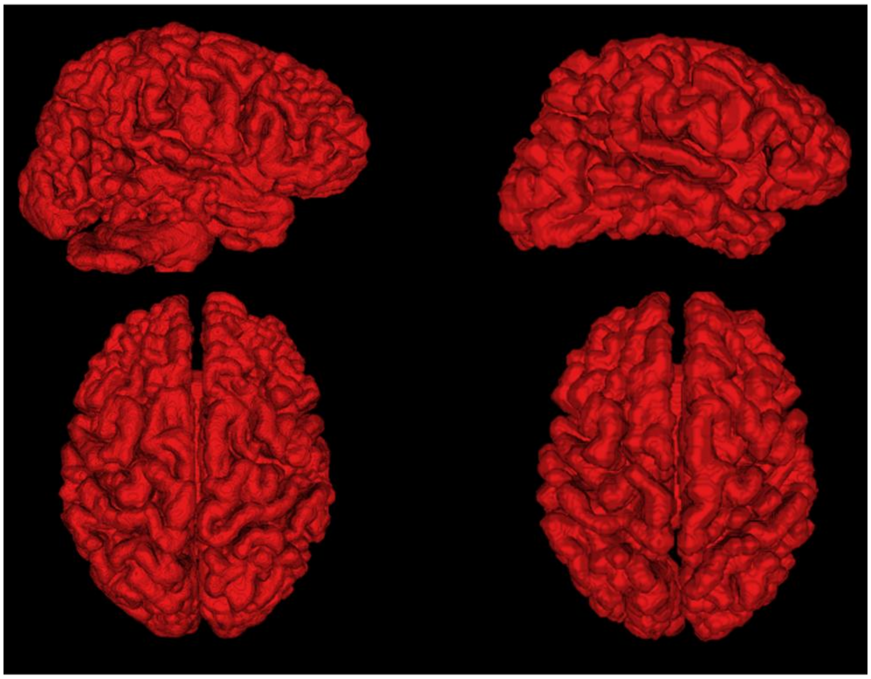

Supplemental Figure SB.F3. Comparison of TractSeg vs FreeSurfer white matter coverage. The overlap in coverage between (left) the aggregate of all TractSeg tracts and (right) the white matter mask from FreeSurfer is substantial. Visualizations of both come from the same scan of a single participant. The most notable difference is that the FreeSurfer mask does not include the cerebellar or brainstem regions.

R2.C10: Supplemental Figure SF.F2: Adjust color bars to better reflect differences (e.g., range from -0.5 to 0.5 or lower bounds). Current settings may obscure relevant variance across brain regions and metrics.

We have altered the colorbar in the figure to the suggested range:

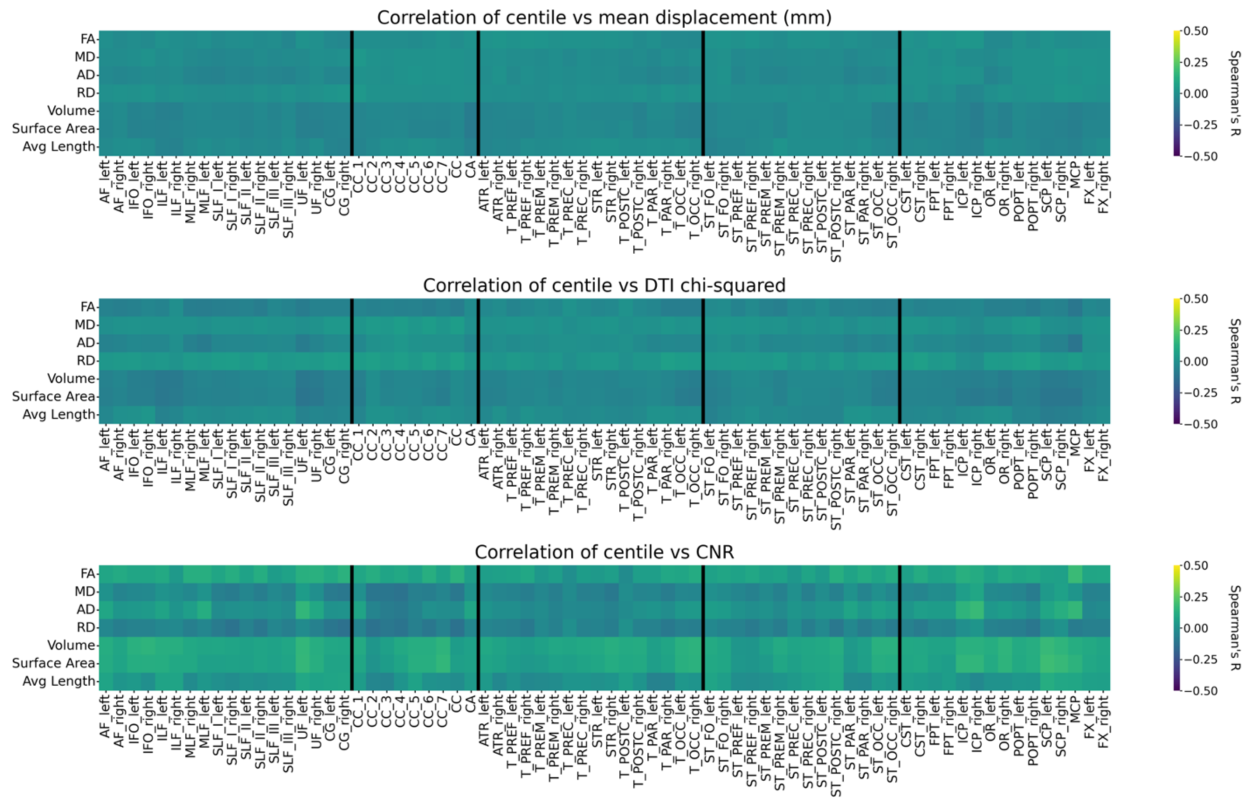

R2.C11: One key advantage of brain charts is their utility in interpreting associations in applied studies. For example, developmental neuroscience often considers stress acceleration/deceleration hypotheses, while adult studies interpret whether “more of metric X” reflects a healthier phenotype. A brief mention of this benefit might be a nice addition for the manuscript.

We agree that this benefit of normative models is important to note, and have added the following sentence to the discussion:

> By providing a normative baseline, a single snapshot in time can be used to determine if an individual exhibits an atypical phenotype relative to their age and sex.

Reviewer 3

R3.C1: This is a resubmission of a manuscript I previously reviewed, developing and showcasing brain growth charts for white matter diffusion metrics across the lifespan. The work was impressive at first submission, and now the authors have done a remarkable job addressing the reviewers’ comments and concerns, including my own. I believe this paper is very strong.

Thank you for your kind assessment of our paper.

R3.C2: One additional question I have concerns Figure 4D – where the authors test the 'last in-first out' hypothesis. The plot for RD shows that early maturity age (e.g., 10-12y) is associated with early decline ages (e.g., 25-35y). This seems to correspond to a 'first in-first out' pattern, rather than a 'last in-first out' pattern. In the 'last in-first out' pattern, I would expect the latest maturing tracts (e.g., 18y, the 'last') to be the first to decline, providing a relationship that is the inverse of that shown here. This graph and the corresponding results should be better explained if they do indeed support a 'last in-first out' hypothesis.

Thank you for highlighting this important point. We have revised this to clarify results: the RD timing plot in Figure 4D shows a positive association between tract maturity age and decline onset age, meaning that earlier-maturing pathways tend to begin declining earlier. This pattern is indeed not consistent with the last-in-first-out prediction (which would imply an inverse relationship). To avoid any ambiguity, we have revised the Results text and the Figure 4 caption (see response to R2.C3) to explicitly state that our timing-based milestone analysis does not support last-in-first-out, and to describe the observed direction of effect (later-maturing pathways tending to show later decline onset). We also provide Supplementary Figure SD.F9, which reports the corresponding milestone-timing and rate-based analyses across all WM features. Finally, we added a brief limitation noting that defining discrete developmental timepoints from continuous trajectories has inherent constraints:

> In our assessments relating developmental and aging milestones for myelination and other properties, we note there are limitations in defining discrete developmental timepoints across continuous curves.

R3.C3: Finally, although I appreciate the linkages with myelin work, and the effort the authors have taken to not equate RD with myelin, I think it would be good to be even more explicit that RD may reflect other changes, e.g., in lines 259-270.

We have edited the text to as the reviewer suggested:

> We emphasize RD as a sensitive, but non-specific, in vivo proxy that can reflect myelination as well as other microstructural factors.

References

[1] S. Mukherjee *et al.*, "Cognitive Domain Harmonization and Cocalibration in Studies of Older Adults," *Neuropsychology*, vol. 37, no. 4, pp. 409–423, Aug. 2022, doi: 10.1037/neu0000835.

[2] B. Reisberg *et al.*, “Retrogenesis: clinical, physiologic, and pathologic mechanisms in brain aging, Alzheimer’s and other dementing processes,” *Eur. Arch. Psychiatry Clin. Neurosci.*, vol. 249 Suppl 3, no. SUPPL. 3, 1999, doi: 10.1007/PL00014170.

[3] D. A. Slater, L. Melie-Garcia, M. Preisig, F. Kherif, A. Lutti, and B. Draganski, “Evolution of white matter tract microstructure across the life span,” *Hum. Brain Mapp.*, vol. 40, no. 7, pp. 2252–2268, May 2019, doi: 10.1002/hbm.24522.

[4] J. D. Yeatman, B. A. Wandell, and A. A. Mezer, “Lifespan maturation and degeneration of human brain white matter,” *Nat. Commun.*, vol. 5, no. 1, p. 4932, Sep. 2014, doi: 10.1038/ncomms5932.

[5] S. J. Forkel, P. Friedrich, M. Thiebaut de Schotten, and H. Howells, “White matter variability, cognition, and disorders: a systematic review,” *Brain Struct. Funct.*, vol. 227, no. 2, p. 529, Mar. 2021, doi: 10.1007/S00429-021-02382-W.

[6] S. Pajevic and C. Pierpaoli, “Color Schemes to Represent the Orientation of Anisotropic Tissues From Diffusion Tensor Data: Application to White Matter Fiber Tract Mapping in the Human Brain,” *Magn. Reson. Med.*, vol. 42, pp. 526–540, Aug. 1999, doi: 10.1002/(SICI)1522-2594(199909)42:3<526::AID-MRM15>3.0.CO;2-J.
